# Efficient Functionalization of Organosulfones via Photoredox Catalysis: Direct Incorporation of *α*-Carbonyl Alkyl Side Chains into *α*-Allyl-*β*-Ketosulfones

**DOI:** 10.3390/molecules29091971

**Published:** 2024-04-25

**Authors:** Hong-Li Huang, Shan Li, Yong-Zheng Lv, Ya-Qian Shi, Tian-Tian Pang, Ru-Fen Zhang, Wenjing Huang, Jianhui Yin, Fei Gao

**Affiliations:** 1 College of Chemistry and Chemical Engineering, Liaocheng University, Liaocheng 252059, China; lishan04118021@163.com (S.L.); 15224388764@163.com (Y.-Z.L.); shiyaqian200009@163.com (Y.-Q.S.); 18853027155@163.com (T.-T.P.); zhangrufen@lcu.edu.cn (R.-F.Z.); 2 Institute of Translation Medicine, Shanghai University, Shanghai 200444, China; hwj@shu.edu.cn (W.H.); yinjianhui@shu.edu.cn (J.Y.)

**Keywords:** functionalized organosulfones, photoredox catalysis, *α*-allyl-*β*-ketosulfones

## Abstract

A novel and efficient method for functionalizing organosulfones has been established, utilizing a visible-light-driven intermolecular radical cascade cyclization of *α*-allyl-*β*-ketosulfones. This process employs *fac*-Ir(ppy)_3_ as the photoredox catalyst and *α*-carbonyl alkyl bromide as the oxidizing agent. Via this approach, the substrates experience intermolecular addition of *α*-carbonyl alkyl radicals to the alkene bonds, initiating a sequence of C-C bond formations that culminate in the production of organosulfone derivatives. Notably, this technique features gentle reaction conditions and an exceptional compatibility with a wide array of functional groups, making it a versatile and valuable addition to the field of organic synthesis.

## 1. Introduction

Organosulfones, exemplified by compounds such as Adociaquinones and Boehringer Ingelheim, serve as versatile components in pharmaceutical molecules, finely modulating drug metabolism and biotransformation, and have been extensively utilized in clinical trials [1,2,3,4,5]. Notably, functionalized organosulfones exhibited diverse reactivity at *α*-methylene/arene positions and facile sulfonyl group removal, enabling their valuable role in synthesizing biologically active compounds, pharmaceuticals, intermediates, and natural products [6,7].

**Figure 1:**
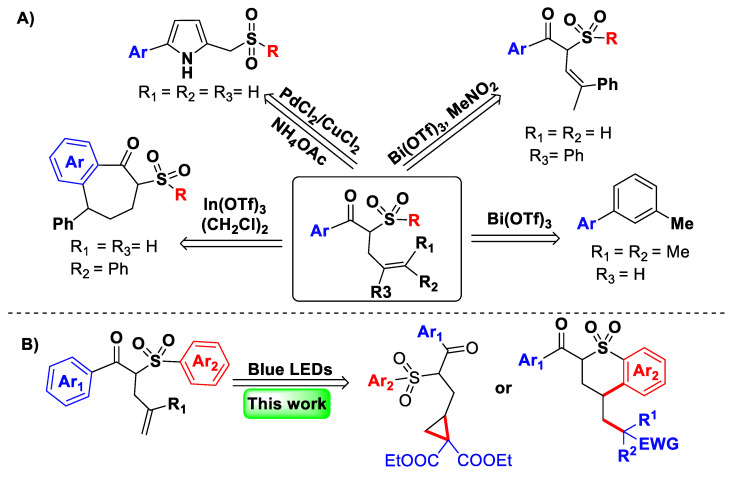
(**A**,**B**) Functionalization of α-benzoyl β-styrylsulfones.

More pioneering efforts have been devoted to developing useful methods for modifying organosulfones [8,9]. Among the functionalized skeletons featuring a distinctive sulfonyl group, *α*-ally-*β*-ketosulfones play a vital role as essential precursors in various organic transformations [10,11], adeptly enabling the functionalization of double bonds (Figure 1). (i) Double bond cyclization [12,13,14]: Numerous cyclization methods of *α*-ally-*β*-ketosulfones have been reported, such as In(OTf)_3_-catalyzed intramolecular hydroarylation of *α*-phenylallyl *β*-ketosulfones [15], Bi(OTf)_3_-mediated cycloisomerization of *γ*-alkynyl arylketones [16], and PdCl_2_/CuCl_2_/NH_4_OAc mediated the domino aerobic Wacker-type aminocyclization of *α*-allyl-*β*-ketosulfones [17]. (ii) *Exo*-olefin isomerization: In 2015, a novel Bi(OTf)_3_-mediated stereoselective *exo*-olefin isomerization of *α*-benzoyl-*β*-styrylsulfones in MeNO_2_ was developed to synthesis α-benzoyl α-cinnamylsulfones [18]. (iii) Aromatization following sulfonation: For instance, Bi(OTf)_3_-mediated intramolecular carbonyl allylation of *β*-ketosulfones has been reported, facilitating the synthesis of substituted benzenes. Additionally, the intermolecular Michael−aldol reaction of *β*-keto sulfones was employed for preparing substituted aryl amines [19].

Compared to metal catalysis, photocatalysis has attracted widespread attention due to its advantages of being environmentally friendly, green, and operating under mild reaction conditions [20,21]. Recently, visible-light-promoted radical cascade reaction of olefins has undoubtedly gained significant momentum [22,23]. In 2021, Tokyuyama used *α*-bromo-*β*-keto esters to accomplish the mild photoredox-catalyzed cyclopropanation of alkenes in an aqueous medium [24]. In addition, intermolecular organophotocatalytic cyclopropannation of unactivated olefins with *α*-bromomalonates was reported, displaying broad functional group tolerance and furnishing highly substituted cyclopropanes [25]. After further comparison of reports from the literature, we reported a novel visible-light-driven intermolecular radical cascade reaction of *α*-allyl-*β*-ketosulfones in the presence of *α*-carbonyl alkyl bromide as the oxidant for investigating valuable functionalized organosulfones (Figure 1B).

## 2. Results and Discussion

The optimization of reaction conditions involved exposing a blend of *α*-allyl-*β*-ketosulfones, *α*-bromo diethyl malonate **2a**, base, and photocatalyst to blue LED irradiation, with the reaction taking place in the presence of N_2_ as the atmosphere (Table 1). In our initial exploration of radical addition reactions, we employed *α*-allyl-*β*-ketosulfones (**1a**) as the substrate, alongside 2,6-lutidine as the base and *fac*-Ir(ppy)_3_ as the photocatalyst, with all experiments conducted in a DMF solvent. Encouragingly, the desired ternary ring product **3a** was obtained in 16% isolated yield (entry 1); meanwhile, **3a′** was observed as a byproduct. Subsequent screening of diverse solvents confirmed chlorobenzene as the optimal choice, such as 1,4-dioxane, trichloromethane, DMSO, toluene, acetone, and *o*-dichlorobenzene (entries 2–8). Further experimentation focusing on the base confirmed 2,6-lutidine as the most effective medium for this transformation (entries 9–13). Notably, additional added LiBF_4_ gave **3a** in enhanced yield (36%) (entries 14–15) [22]. Intriguingly, the attempt to increase the amount of base boosted the yield, use of 5.0 equiv. 2,6-lutidine afforded **3a** in the higher yield of 48% (entries 16–18). And the reaction exhibited greater efficacy when carried out in an inert gas environment, in contrast to the outcomes observed under ambient air conditions (entry 19). Remarkably, control experiments provided solid evidence supporting the crucial role of either visible light or the photocatalyst in facilitating the desired transformation (entries 20–21).

**Scheme 1:**
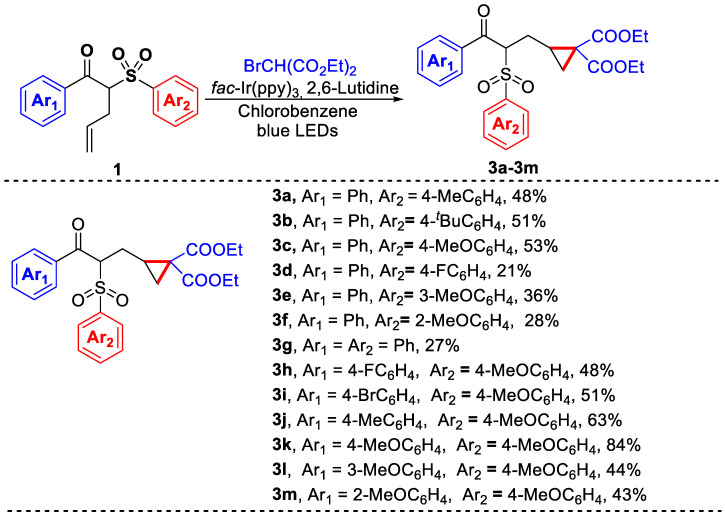
Scope of substrate **1** for synthesizing tetracyclic products **3**. Reaction conditions: **1a** (0.2 mmol, 0.4 M in chlorobenzene), **2a** (2.0 equiv.), LiBF_4_ (2.0 equiv.), base (5.0 equiv.), *fac*-Ir(ppy)_3_ (0.005 mmol), under N_2_ atmosphere irradiated using blue LEDs (5W) at room temperature for 53 h, isolated yield, the dr value of **3** is 2.0:1.

After obtaining the optimized reaction conditions, we proceeded to investigate the range of *α*-allyl-*β*-ketosulfones **1**. As shown in Scheme 1, a diverse set of *α*-allyl-*β*-ketosulfones **1**, spanning a wide range of structural variations, underwent facile transformation to afford the desired products **3**. First, the efficiency of the reaction is significantly influenced by the substitution effect on the Ar_2_ group of skeleton **1**. In cases where Ar_2_ is substituted with electron-donating groups like *tert*-butyl and methoxyl, the yield of product **3b**–**3c** (51–53%) remained unaffected. Nevertheless, the skeleton **1** resulted in the synthesis of the target product (**3d**), achieving a yield of 21%. Regarding the *α*-allyl-*β*-ketosulfone (**1e**–**1g**), it was noted that their reactivity was decreased compared with the *para*-substituted counterparts. Subsquently, altering the Ar_1_ group to incorporate various substituents proved to be compatible. A series of *α*-allyl-*β*-ketosulfones **1** along with 4-MeOC_6_H_4_ substituents on ring Ar_2_, such as 4-F (**1h**) and 4-Cl (**1i**), 4-methyl (**1j**), 4-methoxy (**1k**), 3-methoxy (**1l**), and 2-methoxy (**1m**) substituents on ring Ar_1_ were all employed as the substrates. The result could be attributed to the electron-withdrawing nature of the substituents on Ar_2_, which reduces its reactivity towards oxidation by the catalyst, consequently impacting the formation of the carbocation. The results revealed strong electron-donating groups significantly contributed to improving the reaction’s efficiency. Specifically, the targeted product **3k**, featuring two MeO- groups, was successfully obtained in up to 84% yield.

During the optimization of the ternary ring formation process, we unexpectedly observed the hexacyclic product **4a**. After this, by optimizing the reaction conditions specifically for the synthesis of **4a**, we achieved a pleasing yield of 67% (See SI, Table S1). Furthermore, it is worth noting that the product **4a** yield remained positive even when the reaction was scaled up to a gram level (64% yield). Having obtained the optimized conditions, we conducted a comprehensive exploration of the substrate scope for this transformation, as summarized in Table 2. The Ar_2_ group with diverse substitutes, such as 4-*^t^*Bu (**1b**), 4-MeO (**1c**), 4-Br (**1n**), Ph (**1g**), 2-MeC_6_H_4_- (**1o**), or 3-MeC_6_H_4_- (**1p**), proceeded cyclization reaction smoothly with moderate efficiency (48–43%, respectively, **4b**–**4g**). Notably, the reaction favored electron-donating substituents and exhibited a preference for *ortho*-substitution over *meta*- and *para*-substitution. Following that, we assessed the effect of various substituents on the Ar_1_ group (**4h**–**4j**). The findings suggested that electron-donating groups were more advantageous for this reaction as opposed to electron-withdrawing groups. Then, a range of *α*-allyl-*β*-ketosulfones bearing varied substituents could undergo a remarkably efficient cyclization reaction, leading to the formation of the desired hexacyclic products (**4k**–**4q**) with acceptable to moderate yields.

**Scheme 2:**
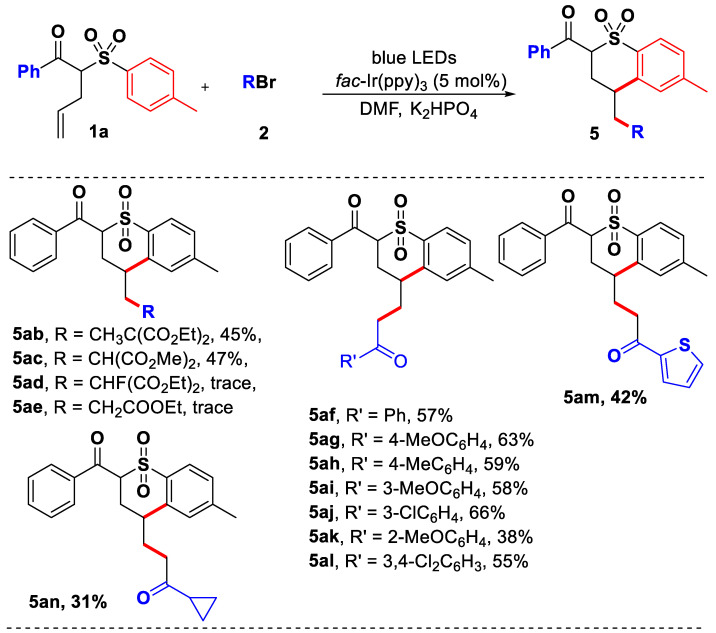
Scope of substrate 2 for hexacyclic products **5**. Reaction conditions: **1a** (0.2 mmol, 0.1 M in dry DMF), **2a** (2.0 equiv.), base (2.0 equiv.), fac-Ir(ppy)_3_ (0.005 mmol), under N_2_ atmosphere irradiated using blue LEDs (5W) at room temperature for 24 h, isolated yield, dr = 1.5:1.

Finally, to expand the applicability of this protocol, we turn our attention to the scope of bromide **2**. As described in Scheme 2, α-bromoalkyl ester **2b** and **2c** proved to be suitable reaction partners, yielding the corresponding products **5ab**–**5ac** in 45–47% isolated yields. Regrettably, the application of α-bromoalkyl esters **2d**–**2e** did not yield favorable results in this transformation, indicating their unsuitability for the process. Then, we redirected our focus towards exploring the viability of 2-bromoacetophenones in this transformation. To our delight, a broad range of 2-bromoacetophenones **5af**–**5al**, featuring with electron-donating or electron-withdrawing substituents at the ortho/meta/para-positions of the benzene ring, all proved to be highly capable partners in successfully achieving this transformation. In this case of 2-(bromoacetyl)thiophene **2m**, the cyclization product **5am** was isolated in 42% yield. As anticipated, this transformation was effectively extended to aliphatic bromide **2n**, yielding the desired cyclization product **5an**.

**Scheme 3:**
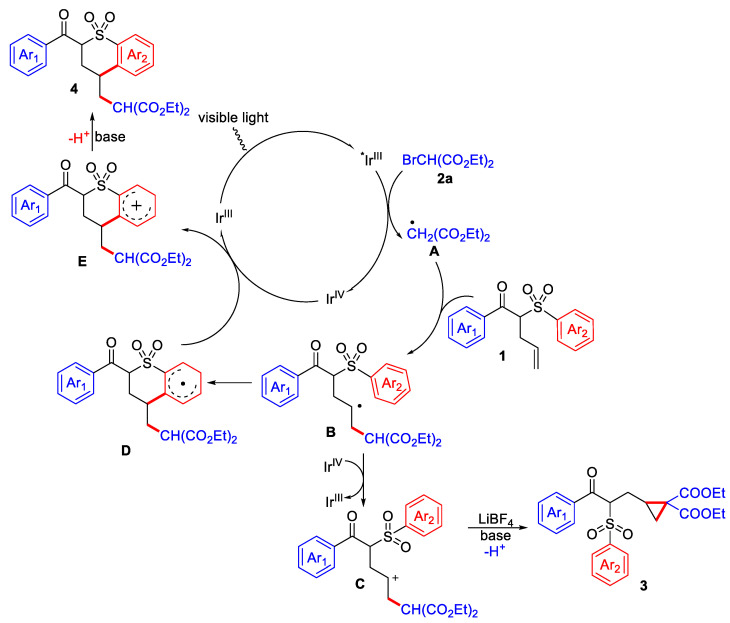
Proposed mechanism.

Based on the afore mentioned result, a plausible mechanism was postulated and detailed in Scheme 3 [26,27]. Upon visible light irradiation, the photocatalyst was energized to *fac-[Ir^III^(ppy)_3_], which underwent rapid reduction via α-bromo diethyl malonate **2a** to yield the corresponding radical specie **A**. Subsequent intermolecular addition to the C-C double bond of compound **1** generated the radical intermediate **B**, followed by oxidation by *fac*-[Ir^IV^(ppy)_3_] to yield the cationic intermediate **C**. Under certain reaction conditions, using 2,6-lutidine as a base and chlorobenzene as the solvent, intermediate **C** underwent an efficient intramolecular cyclization process, leading to the synthesis of the tricyclic compound **3**. On the other hand, when employing potassium dihydrogen phosphate (K_2_HPO_4_) as the base and dimethylformamide (DMF) as the solvent, intermediate **B** directly underwent a radical intramolecular cyclization to give the stable intermediate **D**. Intermediate **D** was oxidized by the photocatalyst Ir^IV^ to produce cationic intermediate **E**, following regeneration of the photocatalyst. Finally, the intermediate **E** favored the loss of a proton on aromatic ring Ar_2_ to form the corresponding hexacyclic product **4**.

## 3. Materials and Methods

### 3.1. General Information

Unless otherwise noted, all reactions were run under a nitrogen atmosphere and were monitored using TLC and visualized using a UV lamp (254 nm)/or via treatment with a solution of 10 g phosphomolybdic acid and 100 mL EtOH followed by heating. All reagents were used as received from commercial sources without further purification. Compounds **2a**–**2n** were purchased from Aladdin Reagent Co. (Shanghai, China). Silica gel (200–300 mesh) and silica gel GF_254_ (10–40 μm) were used for column chromatography (CC) and to prepare thin-layer chromatography (PTLC), respectively. Solvents were dried and purified according to the procedure from “Purification of Laboratory Chemicals book”. ^1^H NMR and ^13^C NMR spectra were recorded in CDCl_3_ on a Varian 500 MHz instrument. Chemical shifts were denoted in ppm (δ) and calibrated using residual undeuterated solvent (CDCl_3_ (7.26 ppm), or tetramethylsilane (0.00 ppm)) as internal reference for ^1^H NMR and the deuterated solvent (CDCl_3_ (77.00 ppm), or tetramethylsilane (0.00 ppm)) as internal standard for ^13^C NMR. The following abbreviations were used to denote the multiplicities: s = singlet, d = doublet, t = triplet, q = quartet, br = broad, td = triple doublet, dt = double triplet, m = multiplet. The MS data were obtained using the ESI technique and the relative intensity (%) is given in brackets. High-resolution mass spectral analysis (HRMS) data were measured using a Waters G2-xs Q-TOF mass spectrometer by means of the ESI technique.

### 3.2. General Procedure for Preparation of Substrates

Substrates **1a [17,28]**, **1b [28]**, **1c**–**d [17]**, **1g [28]**, **1p** [28], **1q**–**r** [17] are known compounds and the analytical data are consistent with the previous literature.

**2-((3-Methoxyphenyl)sulfonyl)-1-phenylpent-4-en-1-one (1e)**: Colorless solid; mp = 95.6–96.1 °C; **^1^H NMR** (500 MHz, CDCl_3_): δ_H_ 7.91 (d, *J* = 9.0 Hz, 2 H), 7.58 (t, *J* = 7.7 Hz, 1 H), 7.45 (t, *J* = 7.7 Hz, 2 H), 7.40 (t, *J* = 7.7 Hz, 1 H), 7.37 (d, *J* = 7.7 Hz, 1 H), 7.23 (s, 1 H), 7.15–7.13 (m, 1 H), 5.63–5.55 (m, 1 H), 5.13 (t, *J* = 7.3 Hz, 1 H), 5.05 (dd, *J* = 17.0, 1.0 Hz, 1 H), 4.98 (dd, *J* = 10.0, 1.0 Hz, 1 H), 3.80 (s, 3 H), 2.88–2.84 (m, 2 H); **^13^C NMR** (126 MHz, CDCl_3_): δ_c_ 191.6, 159.7, 137.4, 137.0, 134.0, 131.8, 129.9, 128.9, 128.7, 121.8, 120.8, 119.0, 114.1, 69.1, 55.6, 32.1; HRMS (ESI-TOF) *m*/*z* [M + Na]^+^ calcd. for C_18_H_18_NaO_4_S^+^, 353.0818, found 353.0801.

**2-((2-Methoxyphenyl)sulfonyl)-1-phenylpent-4-en-1-one (1f)**: Colorless solid; mp = 96.8–97.2 °C; **^1^H NMR** (500 MHz, CDCl_3_): δ_H_ 7.88 (dd, *J* = 8.3, 1.3 Hz, 2 H), 7.85 (dd, *J* = 8.3, 1.3 Hz, 1 H), 7.56–7.50 (m, 2 H), 7.41 (t, *J* = 7.7 Hz, 2 H), 7.04 (td, *J* = 7.7, 1.0 Hz, 1 H), 7.37 (d, *J* = 7.7 Hz, 1 H), 5.66–5.58 (m, 1 H), 5.53 (dd, *J* = 10.7, 3.7 Hz, 1 H), 5.06 (dd, *J* = 17.0, 1.0 Hz, 1 H), 4.97 (dd, *J* = 10.0, 1.0 Hz, 1 H), 3.88 (s, 3 H), 3.16–3.09 (m, 1 H), 2.78–2.74 (m, 1 H); **^13^C NMR** (126 MHz, CDCl_3_): δ_c_ 191.5, 157.3, 137.4, 136.1, 133.6, 132.4, 131.6, 128.7, 128.5, 125.8, 120.8, 118.8, 112.2, 67.4, 56.1, 31.3; HRMS (ESI-TOF) *m*/*z* [M + Na]^+^ calcd. for C_18_H_18_NaO_4_S^+^, 353.0818, found 353.0804.

**1-(4-Fluorophenyl)-2-((4-methoxyphenyl)sulfonyl)pent-4-en-1-one (1h)**: Colorless solid; mp = 117.5–118.5 °C; **^1^H NMR** (500 MHz, CDCl_3_): δ_H_ 8.00 (dd, *J* = 8.7, 5.3 Hz, 2 H), 7.67 (d, *J* = 8.7 Hz, 2 H), 7.15 (t, *J* = 8.7 Hz, 2 H), 6.97 (d, *J* = 8.7 Hz, 2 H), 5.61–5.52 (m, 1 H), 5.06 (dd, *J* = 11.0, 3.5 Hz, 1 H), 5.04–4.96 (m, 2 H), 3.88 (s, 3 H), 2.85–2.71 (m, 2 H); **^13^C NMR** (126 MHz, CDCl_3_): δ_c_ 190.5, 166.2 (d, *J* = 257.5 Hz), 164.3, 133.6 (d, *J* = 3.2 Hz), 132.0, 131.9 (d, *J* = 2.5 Hz), 131.8, 127.4, 119.0, 115.9 (d, *J* = 22.2 Hz), 114.2, 69.4, 55.7, 32.4; **^19^F NMR** (470 MHz, CDCl_3_): δ_F_ −103.2 (s) ppm; HRMS (ESI-TOF) *m*/*z* [M + Na]^+^ calcd. for C_18_H_17_FNaO_4_S^+^, 371.0724, found 371.0735.

**1-(4-Bromophenyl)-2-((4-methoxyphenyl)sulfonyl)pent-4-en-1-one (1i)**: Colorless solid; mp = 115.4–116.1 °C; **^1^H NMR** (500 MHz, CDCl_3_): δ_H_ 7.82 (d, *J* = 8.7 Hz, 2 H), 7.66 (d, *J* = 8.7 Hz, 2 H), 7.62 (d, *J* = 8.7 Hz, 2 H), 6.97 (d, *J* = 8.7 Hz, 2 H), 5.60–5.52 (m, 1 H), 5.05–5.00 (m, 2 H), 4.97 (d, *J* = 10.0 Hz, 1 H), 3.88 (s, 3 H), 2.85–2.71 (m, 2 H); **^13^C NMR** (126 MHz, CDCl_3_): δ_c_ 191.3, 164.4, 135.9, 132.1, 132.0, 131.8, 130.5, 129.5, 127.3, 119.1, 114.2, 69.5, 55.7, 32.4; HRMS (ESI-TOF) *m*/*z* [M + Na]^+^ calcd. for C_18_H_17_BrNaO_4_S^+^, 430.9923, found 430.9924.

**2-((4-Methoxyphenyl)sulfonyl)-1-(p-tolyl)pent-4-en-1-one (1j)**: Colorless solid; mp = 110.1–110.9 °C; **^1^H NMR** (500 MHz, CDCl_3_): δ_H_ 7.85 (d, *J* = 8.5 Hz, 2 H), 7.68 (d, *J* = 8.5 Hz, 2 H), 7.26 (d, *J* = 8.5 Hz, 2 H), 6.95 (d, *J* = 8.5 Hz, 2 H), 5.61–5.53 (m, 1 H), 5.09 (dd, *J* = 11.0, 3.5 Hz, 1 H), 5.02 (dd, *J* = 17.0, 1.3 Hz, 1 H), 4.95 (dd, *J* = 10.3, 1.3 Hz, 1 H), 3.86 (s, 3 H), 2.85–2.72 (m, 2 H), 2.41 (s, 3 H); **^13^C NMR** (126 MHz, CDCl_3_): δ_c_ 191.5, 164.2, 145.1, 134.7, 132.0, 132.0, 129.4, 129.1, 127.6, 118.7, 114.0, 69.1, 55.6, 32.4, 21.7; HRMS (ESI-TOF) *m*/*z* [M + Na]^+^ calcd. for C_19_H_20_NaO_4_S^+^, 367.0975, found 355.0788.

**1-(4-Methoxyphenyl)-2-((4-methoxyphenyl)sulfonyl)pent-4-en-1-one (1k)**: Colorless solid; mp = 75.1–75.8 °C; **^1^H NMR** (500 MHz, CDCl_3_): δ_H_ 7.94 (d, *J* = 9.0 Hz, 2 H), 7.67 (d, *J* = 9.0 Hz, 2 H), 6.95 (d, *J* = 9.0 Hz, 2 H), 6.93 (d, *J* = 9.0 Hz, 2 H), 5.61–5.53 (m, 1 H), 5.07–5.01 (m, 2 H), 4.95 (dd, *J* = 10.0, 1.0 Hz, 1 H), 3.86 (s, 3 H), 3.85 (s, 3 H), 2.84–2.72 (m, 2 H); **^13^C NMR** (126 MHz, CDCl_3_): δ_c_ 190.1, 164.2, 164.1, 132.1, 131.9, 131.5, 130.2, 127.6, 118.6, 114.0, 113.9, 69.0, 55.6, 55.5, 32.4; HRMS (ESI-TOF) *m*/*z* [M + Na]^+^ calcd. for C_19_H_20_NaO_5_S^+^, 383.0924, found 383.0907.

**1-(3-Methoxyphenyl)-2-((4-methoxyphenyl)sulfonyl)pent-4-en-1-one (1l)**: Colorless solid; mp = 103.2–104.1 °C; **^1^H NMR** (500 MHz, CDCl_3_): δ_H_ 7.69 (d, *J* = 9.0 Hz, 2 H), 7.52 (d, *J* = 7.7 Hz, 1 H), 7.42 (t, *J* = 2.0 Hz, 1 H), 7.37 (t, *J* = 7.7 Hz, 1 H), 7.13 (dd, *J* = 7.7, 2.7 Hz, 1 H), 6.96 (d, *J* = 9.0 Hz, 2 H), 5.62–5.54 (m, 1 H), 5.09 (dd, *J* = 11.0, 3.5 Hz, 1 H), 5.03 (dd, *J* = 17.0, 1.3 Hz, 1 H), 4.97 (dd, *J* = 10.0, 1.3 Hz, 1 H), 3.86 (s, 3 H), 3.84 (s, 3 H), 2.86–2.73 (m, 2 H); **^13^C NMR** (126 MHz, CDCl_3_): δ_c_ 192.0, 164.2, 159.8, 138.4, 132.0, 131.9, 129.7, 127.6, 121.7, 120.5, 118.9, 114.1, 112.9, 69.4, 55.6, 55.4, 32.5; HRMS (ESI-TOF) *m*/*z* [M + Na]^+^ calcd. for C_19_H_20_NaO_5_S^+^, 383.0924, found 383.0917.

**1-(2-Methoxyphenyl)-2-((4-methoxyphenyl)sulfonyl)pent-4-en-1-one (1m)**: Colorless solid; mp = 103.6–104.2 °C; **^1^H NMR** (500 MHz, CDCl_3_): δ_H_ 7.67 (d, *J* = 9.0 Hz, 2 H), 7.59 (dd, *J* = 7.7, 1.7 Hz, 1 H), 7.45 (td, *J* = 7.7, 1.7 Hz, 1 H), 6.97 (t, *J* = 8.0 Hz, 1 H), 6.91 (d, *J* = 9.0 Hz, 2 H), 6.89 (d, *J* = 8.0 Hz, 1 H), 5.75–5.67 (m, 1 H), 5.64 (dd, *J* = 9.0, 5.5 Hz, 1 H), 5.08 (dd, *J* = 17.0, 1.5 Hz, 1 H), 5.00 (dd, *J* = 10.3, 1.5 Hz, 1 H), 3.88 (s, 3 H), 3.85 (s, 3 H), 2.83–2.77 (m, 2 H); **^13^C NMR** (126 MHz, CDCl_3_): δ_c_ 193.6, 163.9, 158.5, 134.7, 132.9, 131.7, 131.2, 128.6, 127.7, 120.9, 118.0, 113.8, 111.7, 72.9, 55.6, 55.5, 32.1; HRMS (ESI-TOF) *m*/*z* [M + Na]^+^ calcd. for C_19_H_20_NaO_5_S^+^, 383.0924, found 383.0913.

**2-((4-Bromophenyl)sulfonyl)-1-phenylpent-4-en-1-one (1n)**: Colorless solid; mp = 104.3–105.4 °C; **^1^H NMR** (500 MHz, CDCl_3_): δ_H_ 7.93 (d, *J* = 8.7 Hz, 2 H), 7.66–7.59 (m, 5 H), 7.47 (t, *J* = 8.7 Hz, 2 H), 5.61–5.53 (m, 1 H), 5.15 (dd, *J* = 11.0, 3.5 Hz, 1 H), 5.04 (d, *J* = 17.0 Hz, 1 H), 4.98 (d, *J* = 10.0 Hz, 1 H), 2.87–2.72 (m, 2 H); **^13^C NMR** (126 MHz, CDCl_3_): δ_c_ 191.8, 136.9, 135.1, 134.1, 132.2, 131.5, 131.3, 129.9, 128.9, 128.8, 119.2, 69.1, 32.4; HRMS (ESI-TOF) *m*/*z* [M + Na]^+^ calcd. for C_17_H_15_BrNaO_3_S^+^, 400.9817, found 400.9818.

**1-Phenyl-2-(o-tolylsulfonyl)pent-4-en-1-one (1o)**: Colorless solid; mp = 81.2–81.9 °C; **^1^H NMR** (500 MHz, CDCl_3_): δ_H_ 7.82 (t, *J* = 7.3 Hz, 3 H), 7.54 (t, *J* = 7.3 Hz, 1 H), 7.45–7.38 (m, 3 H), 7.31–7.27 (m, 1 H), 7.21 (d, *J* = 7.3 Hz, 1 H), 5.65–5.55 (m, 1 H), 5.13 (dd, *J* = 11.0, 3.5 Hz, 1 H), 5.07 (d, *J* = 17.0 Hz, 1 H), 4.99 (d, *J* = 10.0 Hz, 1 H), 3.05–2.99 (m, 1 H), 2.88–2.83 (m, 1 H), 2.61 (s, 3 H); **^13^C NMR** (126 MHz, CDCl_3_): δ_c_ 191.4, 139.1, 137.0, 134.2, 133.8, 132.8, 132.0, 131.8, 129.0, 128.6, 128.6, 126.5, 119.1, 69.2, 31.5, 20.8; HRMS (ESI-TOF) *m*/*z* [M + Na]^+^ calcd. for C_18_H_18_NaO_3_S^+^, 337.0869, found 337.0851.

**1-(4-Bromophenyl)-2-tosylpent-4-en-1-one (1s)**: Colorless solid; mp = 109.3–110.2 °C; **^1^H NMR** (500 MHz, CDCl_3_): δ_H_ 7.81 (d, *J* = 8.3 Hz, 2 H), 7.63–7.61 (m, 4 H), 7.32 (d, *J* = 8.3 Hz, 2 H), 5.60–5.52 (m, 1 H), 5.05–5.00 (m, 2 H), 4.97 (d, *J* = 10.5 Hz, 2 H), 2.86–2.72 (m, 2 H), 2.45 (s, 3 H); **^13^C NMR** (126 MHz, CDCl_3_): δ_c_ 191.1, 145.7, 135.9, 133.0, 132.1, 131.8, 130.5, 129.8, 129.6, 129.5, 119.1, 69.4, 32.3, 21.7; HRMS (ESI-TOF) *m*/*z* [M + Na]^+^ calcd. for C_18_H_17_BrNaO_3_S^+^, 414.9974, found 414.9963.

**2-((4-Fluorophenyl)sulfonyl)-1-(p-tolyl)pent-4-en-1-one (1t)**: Colorless solid; mp = 129.4–130.1 °C; **^1^H NMR** (500 MHz, CDCl_3_): δ_H_ 7.84 (d, *J* = 8.5 Hz, 2 H), 7.78 (dd, *J* = 8.5, 5.0 Hz, 2 H), 7.28 (d, *J* = 8.5 Hz, 2 H), 7.19 (t, *J* = 8.5 Hz, 2 H), 5.62–5.53 (m, 1 H), 5.11 (dd, *J* = 10.7, 3.7 Hz, 1 H), 5.04 (dd, *J* = 11.3, 1.3 Hz, 1 H), 4.98 (dd, *J* = 11.3, 1.3 Hz, 1 H), 2.87–2.71 (m, 2 H), 2.43 (s, 3 H); **^13^C NMR** (126 MHz, CDCl_3_): δ_c_ 191.4, 166.2 (d, *J* = 257.8 Hz), 145.4, 134.5, 132.8 (d, *J* = 9.8 Hz), 132.2 (d, *J* = 3.2 Hz), 131.7, 129.6, 129.2, 119.1, 116.2 (d, *J* = 22.7 Hz), 69.1, 32.5, 21.7; **^19^F NMR** (470 MHz, CDCl_3_): δ_F_ −102.4 (s) ppm; HRMS (ESI-TOF) *m*/*z* [M + Na]^+^ calcd. for C_18_H_17_FNaO_3_S^+^, 355.0775, found 355.0778.

**2-((4-Fluorophenyl)sulfonyl)-1-(4-methoxyphenyl)pent-4-en-1-one (1u)**: Colorless solid; mp = 92.4–93.3 °C; **^1^H NMR** (500 MHz, CDCl_3_): δ_H_ 7.94 (d, *J* = 9.0 Hz, 2 H), 7.78 (dd, *J* = 8.7, 5.0 Hz, 2 H), 7.20 (t, *J* = 8.7 Hz, 2 H), 6.95 (d, *J* = 9.0 Hz, 2 H), 5.61–5.53 (m, 1 H), 5.08 (dd, *J* = 11.0, 3.5 Hz, 1 H), 5.04 (dd, *J* = 17.0, 1.3 Hz, 1 H), 4.98 (dd, *J* = 11.0, 1.3 Hz, 1 H), 3.89 (s, 3 H), 2.86–2.70 (m, 2 H); **^13^C NMR** (126 MHz, CDCl_3_): δ_c_ 189.9, 166.2 (d, *J* = 257.8 Hz), 164.5, 132.8 (d, *J* = 9.7 Hz), 132.2 (d, *J* = 3.2 Hz), 131.8, 131.5, 130.0, 119.0, 116.2 (d, *J* = 22.8 Hz), 114.1, 69.0, 55.6, 32.4; **^19^F NMR** (470 MHz, CDCl_3_): δ_F_ −102.5 (s) ppm; HRMS (ESI-TOF) *m*/*z* [M + Na]^+^ calcd. for C_18_H_17_FNaO_4_S^+^, 371.0724, found 371.0730.

**2-((4-Bromophenyl)sulfonyl)-1-(4-methoxyphenyl)pent-4-en-1-one (1v)**: Colorless solid; mp = 102.4–103.2 °C; **^1^H NMR** (500 MHz, CDCl_3_): δ_H_ 7.93 (d, *J* = 8.7 Hz, 2 H), 7.65 (d, *J* = 8.7 Hz, 2 H), 7.61 (d, *J* = 8.7 Hz, 2 H), 6.94 (d, *J* = 8.7 Hz, 2 H), 5.61–5.53 (m, 1 H), 5.09–5.03 (m, 2 H), 4.98 (d, *J* = 10.0 Hz, 1 H), 3.88 (s, 3 H), 2.85–2.71 (m, 2 H); **^13^C NMR** (126 MHz, CDCl_3_): δ_c_ 189.7, 164.5, 135.2, 132.2, 131.8, 131.5, 131.3, 130.0, 129.8, 119.0, 114.1, 69.0, 55.6, 32.4; HRMS (ESI-TOF) *m*/*z* [M + Na]^+^ calcd. for C_18_H_17_BrNaO_4_S^+^, 430.9923, found 430.9934.

**2-((4-Fluorophenyl)sulfonyl)-1-(3-methoxyphenyl)pent-4-en-1-one (1w)**: Colorless solid; mp = 63.5–64.1 °C; **^1^H NMR** (500 MHz, CDCl_3_): δ_H_ 7.79 (dd, *J* = 8.3, 5.0 Hz, 2 H), 7.51 (d, *J* = 8.3 Hz, 1 H), 7.42 (s, 1 H), 7.39 (t, *J* = 8.3 Hz, 1 H), 7.20 (t, *J* = 8.3 Hz, 2 H), 7.15 (dd, *J* = 8.3, 1.7 Hz, 1 H), 5.62–5.54 (m, 1 H), 5.11 (dd, *J* = 11.0, 3.5 Hz, 1 H), 5.05 (dd, *J* = 17.0, 1.0 Hz, 1 H), 4.99 (d, *J* = 10.0 Hz, 1 H), 3.86 (s, 3 H), 2.88–2.72 (m, 2 H); **^13^C NMR** (126 MHz, CDCl_3_): δ_c_ 191.8, 166.3 (d, *J* = 257.9 Hz), 160.0, 138.3, 132.8 (d, *J* = 9.7 Hz), 132.3, 131.6, 129.8, 121.7, 120.7, 119.2, 116.2 (d, *J* = 22.6 Hz), 113.1, 69.5, 55.5, 32.6; **^19^F NMR** (470 MHz, CDCl_3_): δ_F_ −102.3 (s) ppm; HRMS (ESI-TOF) *m*/*z* [M + Na]^+^ calcd. for C_18_H_17_FNaO_4_S^+^, 371.0724, found 371.0728.

**2-((4-Fluorophenyl)sulfonyl)-1-(2-methoxyphenyl)pent-4-en-1-one (1x)**: Colorless solid; mp = 80.2–81.3 °C; **^1^H NMR** (500 MHz, CDCl_3_): δ_H_ 7.77 (dd, *J* = 8.7, 5.0 Hz, 2 H), 7.60 (dd, *J* = 7.7, 1.7 Hz, 1 H), 7.48 (td, *J* = 7.7, 1.7 Hz, 1 H), 7.14 (t, *J* = 8.7 Hz, 2 H), 6.99 (t, *J* = 7.7 Hz, 1 H), 6.90 (d, *J* = 7.7 Hz, 1 H), 5.75–5.65 (m, 2 H), 5.09 (dd, *J* = 17.0, 1.5 Hz, 1 H), 5.02 (d, *J* = 10.0 Hz, 1 H), 3.89 (s, 3 H), 2.85–2.76 (m, 2 H); **^13^C NMR** (126 MHz, CDCl_3_): δ_c_ 193.2, 165.9 (d, *J* = 257.9 Hz), 158.6, 135.0, 133.3, 132.6, 132.5 (d, *J* = 9.7 Hz), 131.3, 127.5, 121.1, 118.3, 115.9 (d, *J* = 22.8 Hz), 111.9, 73.0, 55.6, 32.2; **^19^F NMR** (470 MHz, CDCl_3_): δ_F_ −103.1 (s) ppm; HRMS (ESI-TOF) *m*/*z* [M + Na]^+^ calcd. for C_18_H_17_FNaO_4_S^+^, 371.0724, found 371.0727.

### 3.3. General Procedure for the Synthesis of Compound **3a**

A 25 mL Schlenk flask was equipped with a magnetic stir bar and was charged with compounds **1a** (0.2 mmol), **2** (0.4 mmol), 2,6-lutidine (107.0 mg, 1.0 mmol), LiBF_4_ (37.5 mg, 0.4 mmol), chlorobenzene (0.5 mL), and fac-Ir(ppy)_3_ (6.6 mg, 0.005 mmol). The Schlenk flask was evacuated and backfilled with N_2_ three times under −78 °C. The mixture was irradiated with blue LEDs for 53 h (monitored using TLC). After the reaction was completed, the reaction mixture was purified using PTLC (petroleum ether/EtOAc) to obtain the desired product **3a**.

### 3.4. General Procedure for Gram Scale Reaction of Compound **4a**

A 100 mL Schlenk flask was equipped with magnetic stir bar and was charged with compounds **1a** (1.256 g, 4.0 mmol, 1.0 equiv.), **2a** (3.824g, 8.0 mmol, 2.0 equiv.), K_2_HPO_4_ (2.784 g, 8.0 mmol, 2.0 equiv.), dry DMF (40 mL), and fac-Ir(ppy)_3_ (132 mg, 0.2 mmol). The Schlenk flask was evacuated and backfilled with N_2_ three times under −78 °C. The mixture was irradiated with blue LEDs for 24 h (monitored using TLC). After the reaction was completed, the reaction mixture was quenched with water (50 mL) and was extracted with EtOAc (50 mL × 4). The organic layer was combined, dried (MgSO_4_), filtered, and concentrated in vacuo. The resulting residue was purified using column chromatography on silica gel (petroleum ether/EtOAc) to obtain the desired product **4a**.

**Diethyl 2-(3-oxo-3-phenyl-2-tosylpropyl)cyclopropane-1,1-dicarboxylate (3a)**: Colorless oil (45.3 mg, 48% yield, dr = 2.0:1). **^1^H NMR** (500 MHz, CDCl_3_): δ_H_ 7.96 (d, *J* = 7.7 Hz, 2.0 H), 7.59 (t, *J* = 7.7 Hz, 3.0 H), 7.49–7.44 (m, 2.0 H), 7.28–7.27 (m, 2.0 H), 5.34 (dd, *J* = 11.5, 3.0 Hz, 0.67 H), 5.19 (dd, *J* = 8.7, 4.7 Hz, 0.33 H), 4.30–3.97 (m, 4.0 H), 2.41 (s, 3.0 H), 2.39–2.33 (m, 0.67 H), 2.30–2.24 (m, 0.33 H), 2.00–1.94 (m, 1.0 H), 1.87–1.80 (m, 0.33 H), 1.64–1.57 (m, 0.67 H), 1.36 (t, *J* = 6.7 Hz, 2.0 H), 1.33–1.27 (m, 2.0 H), 1.20 (q, *J* = 6.7 Hz, 2.0 H), 1.10 (t, *J* = 6.7 Hz, 2.0 H); **^13^C NMR** (126 MHz, CDCl_3_): δ_c_ 192.5, 192.0, 169.4, 169.4, 167.4, 167.4, 145.5, 145.4, 137.2, 136.7, 134.0, 134.0, 133.4, 133.2, 129.6, 129.5, 129.1, 128.9, 128.7, 69.3, 68.5, 61.9, 61.6, 61.6, 61.5, 34.4, 33.9, 28.1, 27.6, 24.7, 24.4, 21.6, 20.5, 20.1, 14.1, 14.0, 13.9, 13.8; **IR**: ῡ = 1725, 1678, 1595, 1447, 1147, 748 cm^−^^1^; HRMS (ESI-TOF) *m*/*z* [M + Na]^+^ calcd. for C_25_H_28_NaO_7_S^+^, 495.1448, found 495.1439.

**Diethyl 2-(2-bromo-5-oxo-5-phenyl-4-tosylpentyl)malonate (3a′)**: Colorless oil. ^1^H NMR (500 MHz, CDCl_3_): δ_H_ 7.97 (d, *J* = 7.5 Hz, 2.0 H), 7.62–7.57 (m, 3.0 H), 7.46 (t, *J* = 7.5 Hz, 2.0 H), 7.30–7.27 (m, 2.0 H), 5.56 (dd, *J* = 11.3, 2.7 Hz, 0.67 H), 5.41 (t, *J* = 6.0 Hz, 0.33 H), 4.22–4.09 (m, 4.0 H), 3.73–3.67 (m, 1.0 H), 3.63 (dd, *J* = 10.0, 5.0 Hz, 0.67 H), 2.68–2.63 (m, 1.0 H), 2.56–2.43 (m, 2.0 H), 2.42 (s, 3.0 H), 2.35–2.29 (m, 0.67 H), 2.21–2.15 (m, 0.33 H), 1.27–1.23 (m, 4.33 H), 1.18 (t, *J* = 7.0 Hz, 2.0 H); **^13^C NMR** (126 MHz, CDCl_3_): δc 191.9, 191.3, 168.5, 168.5, 168.3, 168.1, 145.7, 145.7, 136.8, 136.3, 134.2, 134.0, 133.2, 132.9, 129.8, 129.7, 129.7, 129.5, 129.3, 129.2, 128.7, 68.8, 68.2, 61.8, 61.7, 51.2, 50.5, 50.2, 50.2, 38.1, 37.7, 37.2, 36.7, 21.7, 14.0, 13.9, 13.9; **IR**: ῡ = 1728, 1678, 1595, 1447, 1147, 736 cm^−1^; HRMS (ESI-TOF) *m*/*z* [M + Na]^+^ calcd. for C_25_H_29_BrNaO_7_S^+^, 575.0710, found 575.0701.

**Diethyl 2-(2-((4-(tert-butyl)phenyl)sulfonyl)-3-oxo-3-phenylpropyl)cyclopropane-1,1-dicarboxylate (3b)**: Colorless oil (52.4 mg, 51% yield, dr = 2.0:1). **^1^H NMR** (500 MHz, CDCl_3_): δ_H_ 7.91 (d, *J* = 8.0 Hz, 2.0 H), 7.64–7.60 (m, 2.0 H), 7.59–7.55 (m, 1.0 H), 7.46–7.41 (m, 4.0 H), 5.34 (dd, *J* = 11.5, 3.0 Hz, 0.67 H), 5.18 (dd, *J* = 9.0, 4.5 Hz, 0.33 H), 4.30–3.97 (m, 4.0 H), 2.46–2.40 (m, 0.67 H), 2.34–2.28 (m, 0.33 H), 2.02–1.94 (m, 1.0 H), 1.88–1.82 (m, 0.33 H), 1.65–1.59 (m, 1.0 H), 1.35 (t, *J* = 7.3 Hz, 2.0 H), 1.34–1.31(m, 1.67 H), 1.30 (s, 9.0 H), 1.22–1.18 (m, 2.0 H), 1.09 (t, *J* = 7.3 Hz, 2.0 H); **^13^C NMR** (126 MHz, CDCl_3_): δ_c_ 192.5, 192.0, 169.4, 169.4, 167.5, 167.4, 158.3, 158.2, 137.2, 136.8, 134.0, 134.0, 133.6, 133.3 129.5, 129.4, 129.0, 128.8, 128.7, 125.9, 69.2, 68.4, 61.9, 61.6, 61.5, 35.2, 34.5, 34.0, 31.0, 30.9, 27.8, 27.4, 24.8, 24.5, 20.5, 20.1, 14.1, 14.1, 14.0, 13.9; **IR**: ῡ = 1723, 1682, 1594, 1447, 1153, 734 cm^−^^1^; HRMS (ESI-TOF) *m*/*z* [M + Na]^+^ calcd. for C_28_H_34_NaO_7_S^+^, 537.1917, found 537.1893.

**Diethyl 2-(2-((4-methoxyphenyl)sulfonyl)-3-oxo-3-phenylpropyl)cyclopropane-1,1-dicarboxylate (3c)**: Colorless oil (51.8 mg, 53% yield, dr = 2.0:1). **^1^H NMR** (500 MHz, CDCl_3_): δ_H_ 7.97 (d, *J* = 8.0 Hz, 2.0 H), 7.65–7.59 (m, 3.0 H), 7.50–7.45 (m, 2.0 H), 6.94–6.92 (m, 2.0 H), 5.33 (dd, *J* = 11.5, 3.0 Hz, 0.67 H), 5.19 (dd, *J* = 9.0, 4.5 Hz, 0.33 H), 4.32–3.98 (m, 4.0 H), 3.85 (s, 3.0 H), 2.35–2.23 (m, 1.0 H), 1.99–1.92 (m, 1.0 H), 1.86–1.79 (m, 0.33 H), 1.63–1.57 (m, 1.0 H), 1.36 (t, *J* = 7.3 Hz, 2.0 H), 1.33–1.24 (m, 1.67 H), 1.20 (q, *J* = 7.3 Hz, 2.0 H), 1.10 (t, *J* = 7.3 Hz, 2.0 H); **^13^C NMR** (126 MHz, CDCl_3_): δ_c_ 192.7, 192.2, 169.4, 169.4, 167.5, 167.4, 164.2, 164.2, 137.2, 136.7, 134.1, 134.1, 131.9, 131.9, 129.2, 129.0, 128.7, 127.6, 127.4, 114.1, 69.3, 68.5, 62.0, 61.6, 61.6, 55.7, 34.4, 33.9, 28.2, 27.7, 24.7, 24.4, 20.5, 20.1, 14.1, 14.0, 14.0, 13.9; **IR**: ῡ = 1722, 1680, 1594, 1447, 1145, 736 cm^−^^1^; HRMS (ESI-TOF) *m*/*z* [M + Na]^+^ calcd. for C_25_H_28_NaO_8_S^+^, 511.1397, found 511.1398.

**Diethyl 2-(2-((4-fluorophenyl)sulfonyl)-3-oxo-3-phenylpropyl)cyclopropane-1,1-dicarboxylate (3d)**: Colorless oil (20.1 mg, 21% yield, dr = 2.0:1). **^1^H NMR** (500 MHz, CDCl_3_): δ_H_ 7.96 (d, *J* = 8.0 Hz, 2.0 H), 7.76–7.71 (m, 2.0 H), 7.64–7.60 (m, 1.0 H), 7.50–7.46 (m, 2.0 H), 7.18–7.14 (m, 2.0 H), 5.38 (dd, *J* = 11.5, 3.0 Hz, 0.67 H), 5.22 (dd, *J* = 8.7, 4.7 Hz, 0.33 H), 4.33–3.98 (m, 4.0 H), 2.41–2.32 (m, 0.67 H), 2.27–2.20 (m, 0.33 H), 2.04–1.94 (m, 1.0 H), 1.87–1.81 (m, 0.33 H), 1.65–1.60 (m, 0.67 H), 1.37 (t, *J* = 7.0 Hz, 2.0 H), 1.33–1.25 (m, 2.0 H), 1.21 (m, 2.0 H), 1.10 (t, *J* = 7.0 Hz, 2.0 H); **^13^C NMR** (126 MHz, CDCl_3_): δ_c_ 192.5, 192.0, 169.4, 169.3, 167.5, 167.2, 166.2 (d, *J* = 258.2 Hz), 137.0, 136.5, 134.3, 134.3, 132.7, 132.7, 132.6, 132.6, 132.3 (d, *J* = 3.2 Hz), 132.1 (d, *J* = 3.2 Hz), 129.1, 128.9, 128.9, 116.2 (d, *J* = 22.9 Hz), 69.3, 68.4, 62.1, 61.7, 61.6, 34.5, 34.0, 28.2, 27.7, 24.7, 24.3, 20.5, 20.2, 14.1, 14.1, 14.0, 13.9; **^19^F NMR** (470 MHz, CDCl_3_): δ_F_ −102.1 (s) ppm; **IR**: ῡ = 1722, 1680, 1594, 1447, 1145, 736 cm^−^^1^; HRMS (ESI-TOF) *m*/*z* [M + Na]^+^ calcd. for C_24_H_25_FNaO_7_S^+^, 499.1197, found 499.1189.

**Diethyl 2-(2-((3-methoxyphenyl)sulfonyl)-3-oxo-3-phenylpropyl)cyclopropane-1,1-dicarboxylate (3e)**: Colorless oil (35.1 mg, 36% yield, dr = 2.0:1). **^1^H NMR** (500 MHz, CDCl_3_): δ_H_ 7.93 (d, *J* = 8.0 Hz, 2.0 H), 7.61–7.57 (m, 1.0 H), 7.48–7.43 (m, 2.0 H), 7.39–7.36 (m, 1.0 H), 7.33–7.31 (m, 1.0 H), 7.18 (d, *J* = 8.0 Hz, 1.0 H), 7.11 (d, *J* = 8.0 Hz, 1.0 H), 5.36 (dd, *J* = 11.3, 2.7 Hz, 0.67 H), 5.20 (dd, *J* = 9.0, 5.0 Hz, 0.33 H), 4.30–3.97 (m, 4.0 H), 3.80 (s, 2.0 H), 3.79 (s, 1.0 H), 2.47–2.41 (m, 0.67 H), 2.35–2.30 (m, 0.33 H), 2.04–1.95 (m, 1.0 H), 1.87–1.81 (m, 0.33 H), 1.63–1.58 (m, 0.67 H), 1.37–1.25 (m, 5.0 H), 1.22–1.19 (m, 2.0 H), 1.09 (t, *J* = 7.0 Hz, 2.0 H); **^13^C NMR** (126 MHz, CDCl_3_): δ_c_ 192.2, 191.7, 169.4, 169.4, 167.5, 167.4, 159.7, 137.6, 137.5, 137.1, 136.7, 134.1, 134.1, 130.0, 129.9, 129.0, 128.9, 128.7, 121.7, 121.7, 121.0, 120.8, 113.9, 113.9, 69.3, 68.4, 62.0, 61.7, 61.6, 61.6, 55.7, 55.6, 34.5, 33.9, 27.9, 27.5, 24.8, 24.4, 20.5, 20.2, 14.1, 14.0, 14.0, 13.9; **IR**: ῡ = 1722, 1680, 1594, 1447, 1145, 736 cm^−^^1^; HRMS (ESI-TOF) *m*/*z* [M + Na]^+^ calcd. for C_24_H_25_FNaO_7_S^+^, 499.1197, found 499.1193.

**Diethyl 2-(2-((2-methoxyphenyl)sulfonyl)-3-oxo-3-phenylpropyl)cyclopropane-1,1-dicarboxylate (3f)**: Colorless oil (27.4 mg, 28% yield, dr = 2.0:1). **^1^H NMR** (500 MHz, CDCl_3_): δ_H_ 7.93 (d, *J* = 8.0 Hz, 2.0 H), 7.80 (dd, *J* = 8.0, 1.3 Hz, 1.0 H), 7.57–7.53 (m, 1.0 H), 7.52–7.47 (m, 1.0 H), 7.42 (q, *J* = 8.0 Hz, 2.0 H), 7.04–6.99 (m, 1.0 H), 6.92 (d, *J* = 8.0 Hz, 0.33 H), 6.86 (d, *J* = 8.0 Hz, 0.67 H), 5.77 (dd, *J* = 11.5, 3.0 Hz, 0.67 H), 5.64 (dd, *J* = 8.0, 5.5 Hz, 0.33 H), 4.19–3.97 (m, 4.0 H), 3.93 (s, 1.0 H), 3.91 (s, 2.0 H), 2.71–2.65 (m, 0.67 H), 2.48–2.42 (m, 0.33 H), 2.04–1.99 (m, 0.33 H), 1.93–1.81 (m, 1.0 H), 1.71–1.64 (m, 0.67 H), 1.38–1.25 (m, 5.0 H), 1.20 (t, *J* = 7.3 Hz, 1.0 H), 1.16 (t, *J* = 7.3 Hz, 1.0 H), 1.09 (t, *J* = 7.3 Hz, 2.0 H); **^13^C NMR** (126 MHz, CDCl_3_): δ_c_ 192.3, 191.6, 169.5, 169.5, 167.4, 167.4, 157.5, 157.3, 137.6, 137.1, 136.1, 136.0, 133.7, 133.7, 131.6, 131.3, 128.9, 128.7, 128.5, 126.1, 125.7, 120.8, 120.6, 112.3, 112.1, 67.6, 66.3, 61.7, 61.6, 61.6, 61.5, 56.2, 56.1, 34.6, 33.9, 27.2, 27.0, 25.1, 24.7, 20.7, 20.3, 14.0, 14.0, 14.0, 13.9; **IR**: ῡ = 1722, 1680, 1594, 1447, 1145, 736 cm^−^^1^; HRMS (ESI-TOF) *m*/*z* [M + Na]^+^ calcd. for C_24_H_25_FNaO_7_S^+^, 499.1197, found 499.1192.

**Diethyl 2-(3-oxo-3-phenyl-2-(phenylsulfonyl)propyl)cyclopropane-1,1-dicarboxylate (3g)**: Colorless oil (24.8 mg, 27% yield, dr = 2.0:1). **^1^H NMR** (500 MHz, CDCl_3_): δ_H_ 7.94 (d, *J* = 7.5 Hz, 2.0 H), 7.72 (t, *J* = 7.5 Hz, 2.0 H), 7.62–7.57 (m, 2.0 H), 7.50–7.43 (m, 4.0 H), 5.37 (dd, *J* = 11.5, 3.0 Hz, 0.67 H), 5.21 (dd, *J* = 9.0, 5.0 Hz, 0.33 H), 4.29–3.98 (m, 4.0 H), 2.42–2.36 (m, 0.67 H), 2.34–2.25 (m, 0.33 H), 2.02–1.94 (m, 1.0 H), 1.86–1.80 (m, 0.33 H), 1.63–1.57 (m, 0.67 H), 1.37–1.29 (m, 4.0 H), 1.20 (q, *J* = 7.3 Hz, 2.0 H), 1.09 (t, *J* = 7.3 Hz, 2.0 H); **^13^C NMR** (126 MHz, CDCl_3_): δ_c_ 192.3, 191.9, 169.4, 169.3, 167.4, 167.4, 137.1, 136.7, 136.4, 136.3, 134.3, 134.2, 134.1, 134.1, 129.6, 129.6, 129.1, 128.9, 128.7, 69.3, 68.4, 61.9, 61.6, 61.6, 61.5, 34.4, 33.9, 28.0, 27.6, 24.7, 24.4, 20.5, 20.1, 14.1, 14.0, 13.9, 13.8; **IR**: ῡ = 1720, 1681, 1447, 1137, 911, 731 cm^−^^1^; HRMS (ESI-TOF) *m*/*z* [M + Na]^+^ calcd. for C_24_H_26_NaO_7_S^+^, 481.1291, found 481.1272.

**Diethyl 2-(3-(4-fluorophenyl)-2-((4-methoxyphenyl)sulfonyl)-3-oxopropyl)cyclopropane-1,1-dicarboxylate (3h)**: Colorless oil (48.6 mg, 48% yield, dr = 2.0:1). **^1^H NMR** (500 MHz, CDCl_3_): δ_H_ 8.02–8.00 (m, 2.0 H), 7.61 (t, *J* = 8.5 Hz, 2.0 H), 7.16–7.12 (m, 2.0 H), 6.94–6.92 (m, 2.0 H), 5.27 (dd, *J* = 11.5, 2.7 Hz, 0.67 H), 5.13 (dd, *J* = 9.0, 4.5 Hz, 0.33 H), 4.30–3.98 (m, 4.0 H), 3.85 (s, 3.0 H), 2.36–2.30 (m, 0.67 H), 2.24–2.18 (m, 0.33 H), 1.98–1.89 (m, 1.0 H), 1.84–1.78 (m, 0.33 H), 1.61–1.54 (m, 0.67 H), 1.35 (t, *J* = 7.3 Hz, 2.0 H), 1.32–1.24 (m, 2.0 H), 1.22–1.18 (m, 2.0 H), 1.10 (t, *J* = 7.3 Hz, 2.0 H); **^13^C NMR** (126 MHz, CDCl_3_): δ_c_ 191.0, 190.6, 169.4, 169.3, 167.4, 166.3 (d, *J* = 257.5 Hz), 164.3, 164.2, 133.6 (d, *J* = 2.8 Hz), 133.2 (d, *J* = 2.8 Hz), 132.0, 132.0, 131.8, 131.8, 131.8, 131.8, 127.5, 127.3, 115.9 (d, *J* = 22.2 Hz), 115.9 (d, *J* = 22.2 Hz), 114.1, 114.1, 69.4, 68.5, 62.0, 61.6, 61.6, 61.6, 55.6, 34.4, 33.9, 28.1, 27.7, 24.7, 24.3, 20.5, 20.1, 14.1, 14.0, 13.9, 13.8; **^19^F NMR** (470 MHz, CDCl_3_): δ_F_ −103.0 (s) ppm; **IR**: ῡ = 1720, 1675, 1590, 1492, 1148, 733 cm^−^^1^; HRMS (ESI-TOF) *m*/*z* [M + Na]^+^ calcd. for C_25_H_27_FNaO_7_S^+^, 529.1303, found 529.1297.

**Diethyl 2-(3-(4-bromophenyl)-2-((4-methoxyphenyl)sulfonyl)-3-oxopropyl)cyclopropane-1,1-dicarboxylate (3i)**: Colorless oil (57.8 mg, 51% yield, dr = 2.0:1). **^1^H NMR** (500 MHz, CDCl_3_): δ_H_ 7.83 (d, *J* = 8.5 Hz, 2.0 H), 7.63–7.59 (m, 4.0 H), 6.95–6.92 (m, 2.0 H), 5.26 (dd, *J* = 11.5, 3.0 Hz, 0.67 H), 5.12 (dd, *J* = 8.7, 4.7 Hz, 0.33 H), 4.31–3.99 (m, 4.0 H), 3.86 (s, 3.0 H), 2.39–2.31 (m, 0.67 H), 2.23–2.17 (m, 0.33 H), 2.01–1.89 (m, 1.0 H), 1.85–1.79 (m, 0.33 H), 1.59–1.54 (m, 0.67 H), 1.36 (t, *J* = 7.0 Hz, 2.0 H), 1.34–1.28 (m, 2.0 H), 1.23–1.19 (m, 2.0 H), 1.12 (t, *J* = 7.0 Hz, 2.0 H); **^13^C NMR** (126 MHz, CDCl_3_): δ_c_ 191.8, 191.3, 169.4, 169.4, 167.4, 164.4, 164.3, 135.9, 135.5, 132.1, 132.1, 131.8, 130.6, 130.4, 129.6, 127.5, 127.3, 114.2, 69.6, 68.6, 62.0, 61.7, 61.7, 61.6, 55.7, 34.4, 33.9, 28.1, 27.7, 24.7, 24.3, 20.5, 20.1, 14.1, 14.0, 14.0, 13.9; **IR**: ῡ = 1720, 1680, 1593, 1496, 1136, 731 cm^−^^1^; HRMS (ESI-TOF) *m*/*z* [M + Na]^+^ calcd. for C_25_H_27_BrNaO_8_S^+^, 589.0502, found 589.0512.

**Diethyl 2-(2-((4-methoxyphenyl)sulfonyl)-3-oxo-3-(p-tolyl)propyl)cyclopropane-1,1-dicarboxylate (3j)**: Colorless oil (63.3 mg, 63% yield, dr = 2.0:1). **^1^H NMR** (500 MHz, CDCl_3_): δ_H_ 7.87 (d, *J* = 8.7 Hz, 2.0 H), 7.62 (t, *J* = 8.7 Hz, 2.0 H), 7.28–7.25 (m, 2.0 H), 6.94–6.91 (m, 2.0 H), 5.29 (dd, *J* = 11.5, 3.0 Hz, 0.67 H), 5.15 (dd, *J* = 9.0, 4.5 Hz, 0.33 H), 4.32–3.98 (m, 4.0 H), 3.85 (s, 3.0 H), 2.42 (s, 1.0 H), 2.41 (s, 2.0 H), 2.32–2.22 (m, 1.0 H), 1.98–1.89 (m, 1.0 H), 1.85–1.78 (m, 0.33 H), 1.62–1.57 (m, 0.67 H), 1.36 (t, *J* = 7.3 Hz, 2.0 H), 1.34–1.24 (m, 2.0 H), 1.23–1.18 (m, 2.0 H), 1.10 (t, *J* = 7.3 Hz, 2.0 H); **^13^C NMR** (126 MHz, CDCl_3_): δ_c_ 192.0, 191.6, 169.4, 169.4, 167.5, 167.4, 164.2, 164.1, 145.3, 145.2, 134.8, 134.3, 131.9, 129.4, 129.3, 129.1, 127.7, 127.5, 114.0, 114.0, 69.2, 68.4, 62.0, 61.6, 61.6, 61.5, 55.6, 34.4, 33.9, 28.2, 27.7, 24.7, 24.5, 21.7, 21.7, 20.5, 20.1, 14.1, 14.1, 14.0, 13.8; **IR**: ῡ = 1720, 1675, 1590, 1492, 1148, 733 cm^−^^1^; HRMS (ESI-TOF) *m*/*z* [M + Na]^+^ calcd. for C_26_H_30_NaO_8_S^+^, 525.1554, found 525.1542.

**Diethyl 2-(3-(4-methoxyphenyl)-2-((4-methoxyphenyl)sulfonyl)-3-oxopropyl)cyclopropane-1,1-dicarboxylate (3k)**: Colorless oil (87.1 mg, 84% yield, dr = 2.0:1). **^1^H NMR** (500 MHz, CDCl_3_): δ_H_ 7.96 (d, *J* = 9.3 Hz, 2.0 H), 7.62 (t, *J* = 9.3 Hz, 2.0 H), 6.94–6.91 (m, 4.0 H), 5.25 (dd, *J* = 11.3, 2.7 Hz, 0.67 H), 5.11 (dd, *J* = 9.3, 4.7 Hz, 0.33 H), 4.30–3.97 (m, 4.0 H), 3.88 (s, 1.0 H), 3.87 (s, 2.0 H), 3.84 (s, 3.0 H), 2.33–2.22 (m, 1.0 H), 1.95–1.86 (m, 1.0 H), 1.84–1.78 (m, 0.33 H), 1.63–1.57 (m, 0.67 H), 1.35 (t, *J* = 7.3 Hz, 2.0 H), 1.34–1.24 (m, 2.0 H), 1.23–1.18 (m, 2.0 H), 1.10 (t, *J* = 7.3 Hz, 2.0 H); **^13^C NMR** (126 MHz, CDCl_3_): δ_c_ 190.5, 190.1, 169.4, 169.4, 167.4, 167.4, 164.3, 164.1, 164.1, 131.8, 131.7, 131.5, 130.2, 129.8, 127.7, 114.0, 114.0, 113.9, 69.0, 68.2, 63.1, 62.0, 61.9, 61.6, 61.5, 56.4, 55.6, 55.6, 55.6, 34.4, 33.9, 28.1, 27.7, 24.7, 24.5, 20.5, 20.1, 14.1, 14.0, 13.9, 13.9; **IR**: ῡ = 1720, 1679, 1583, 1318, 1135, 804 cm^−^^1^; HRMS (ESI-TOF) *m*/*z* [M + Na]^+^ calcd. for C_26_H_30_NaO_9_S^+^, 541.1503, found 541.1496.

**Diethyl 2-(3-(3-methoxyphenyl)-2-((4-methoxyphenyl)sulfonyl)-3-oxopropyl)cyclopropane-1,1-dicarboxylate (3l)**: Colorless oil (45.7 mg, 44% yield, dr = 2.0:1). **^1^H NMR** (500 MHz, CDCl_3_): δ_H_ 7.63 (t, *J* = 8.0 Hz, 2.0 H), 7.54 (d, *J* = 8.0 Hz, 1.0 H), 7.44 (s, 1.0 H), 7.39–7.34 (m, 1.0 H), 7.15–7.12 (m, 1.0 H), 6.94–6.91 (m, 2.0 H), 5.31 (dd, *J* = 11.5, 3.0 Hz, 0.67 H), 5.15 (dd, *J* = 9.3, 4.7 Hz, 0.33 H), 4.31–3.98 (m, 4.0 H), 3.85 (s, 6.0 H), 2.37–2.23 (m, 1.0 H), 1.97–1.92 (m, 1.0 H), 1.85–1.78 (m, 0.33 H), 1.63–1.57 (m, 0.67 H), 1.36 (t, *J* = 7.3 Hz, 3.0 H), 1.32–1.24 (m, 2.0 H), 1.23–1.18 (m, 2.0 H), 1.11 (t, *J* = 7.3 Hz, 3.0 H); **^13^C NMR** (126 MHz, CDCl_3_): δ_c_ 192.6, 192.1, 169.4, 169.4, 167.4, 167.4, 164.2, 164.2, 159.8, 159.8, 138.5, 138.1, 131.9, 129.7, 129.7, 127.7, 127.6, 121.9, 121.7, 120.8, 120.7, 114.1, 114.1, 113.0, 112.7, 69.5, 68.6, 62.0, 61.6, 61.6, 61.5, 55.7, 55.6, 55.5, 55.4, 34.4, 33.9, 28.3, 27.8, 24.7, 24.4, 20.5, 20.1, 14.1, 14.0, 13.9, 13.8; **IR**: ῡ = 1720, 1679, 1583, 1318, 1135, 804 cm^−^^1^; HRMS (ESI-TOF) *m*/*z* [M + Na]^+^ calcd. for C_26_H_30_NaO_9_S^+^, 541.1503, found 541.1495.

**Diethyl 2-(3-(2-methoxyphenyl)-2-((4-methoxyphenyl)sulfonyl)-3-oxopropyl)cyclopropane-1,1-dicarboxylate (3m)**: Colorless oil (44.5 mg, 43% yield, dr = 2.0:1). **^1^H NMR** (500 MHz, CDCl_3_): δ_H_ 7.60–7.55 (m, 3.0 H), 7.47–7.42 (m, 1.0 H), 6.97 (t, *J* = 7.5 Hz, 1.0 H), 6.89–6.85 (m, 3.0 H), 5.82 (dd, *J* = 11.5, 3.0 Hz, 0.67 H), 5.67 (dd, *J* = 9.7, 3.7 Hz, 0.33 H), 4.34–4.07 (m, 4.0 H), 3.89 (s, 1.0 H), 3.88 (s, 2.0 H), 3.83 (s, 3.0 H), 2.47–2.41 (m, 0.33 H), 2.34–2.28 (m, 1.0 H), 1.98–1.87 (m, 1.0 H), 1.84–1.78 (m, 1.0 H), 1.42–1.37 (m, 1.67 H), 1.34 (t, *J* = 7.3 Hz, 3.0 H), 1.19 (t, *J* = 7.3 Hz, 3.0 H); **^13^C NMR** (126 MHz, CDCl_3_): δ_c_ 194.0, 193.6, 169.9, 169.7, 167.5, 167.4, 163.9, 163.8, 158.5, 158.4, 134.7, 134.6, 131.5, 131.5, 131.3, 131.2, 128.5, 128.4, 127.9, 127.7, 120.9, 120.8, 113.8, 111.6, 111.6, 73.0, 72.5, 61.8, 61.5, 61.5, 61.4, 55.6, 55.4, 34.5, 34.2, 27.2, 27.0, 25.1, 25.0, 20.9, 20.5, 14.1, 14.1, 14.0, 14.0; **IR**: ῡ = 1720, 1679, 1583, 1318, 1135, 804 cm^−^^1^; HRMS (ESI-TOF) *m*/*z* [M + Na]^+^ calcd. for C_26_H_30_NaO_9_S^+^, 541.1503, found 541.1501.

**Diethyl 2-((2-benzoyl-6-methyl-1,1-dioxidothiochroman-4-yl)methyl)malonate (4a)**: Colorless oil (63.4 mg, 67% yield, dr = 2.0:1; 4.5 mmol scale, 1.413 g, 64% yield). **^1^H NMR** (500 MHz, CDCl_3_): δ_H_ 8.14 (d, *J* = 7.5 Hz, 1.34 H), 8.07 (d, *J* = 7.5 Hz, 0.66 H), 7.81 (d, *J* = 8.5 Hz, 0.33 H), 7.76 (d, *J* = 8.5 Hz, 0.67 H), 7.65–7.59 (m, 1.0 H), 7.54–7.48 (m, 2.0 H), 7.33 (s, 0.33 H), 7.26–7.23 (m, 1.0 H), 7.18 (s, 0.67 H), 5.41 (dd, *J* = 10.5, 3.5 Hz, 0.67 H), 5.19 (dd, *J* = 11.0, 4.5 Hz, 0.33 H), 4.30–4.10 (m, 4.0 H), 3.61 (dd, *J* = 11.0, 5.0 Hz, 0.33 H), 3.46 (t, *J* = 7.3 Hz, 0.67 H), 3.25–3.20 (m, 1.0 H), 3.07–3.00 (m, 0.67 H), 2.79–2.70 (m, 0.67 H), 2.59–2.44 (m, 1.33 H), 2.41 (s, 3.0 H), 2.39–2.23 (m, 1.67 H), 1.31–1.23 (m, 6.0 H); **^13^C NMR** (126 MHz, CDCl_3_): δ_c_ 189.9, 189.8, 169.0, 168.9, 168.6, 144.1, 143.6, 139.7, 139.7, 136.4, 136.3, 135.1, 135.0, 134.3, 134.3, 129.7, 129.6, 129.5, 129.0, 128.7, 128.7, 128.4, 128.3, 124.6, 124.2, 63.6, 61.9, 61.8, 61.4, 49.9, 49.2, 34.7, 34.5, 34.5, 33.7, 30.3, 28.7, 21.7, 21.5, 14.1, 14.0, 14.0, 14.0; **IR**: ῡ = 1726, 1682, 1596, 1447, 1137, 735 cm^−^^1^; HRMS (ESI-TOF) *m*/*z* [M + Na]^+^ calcd. for C_24_H_26_NaO_7_S^+^, 495.1448, found 495.1436.

**Diethyl 2-((2-benzoyl-6-(tert-butyl)-1,1-dioxidothiochroman-4-yl)methyl)malonate (4b)**: Colorless oil (59.6 mg, 61% yield, dr = 2.0:1). **^1^H NMR** (500 MHz, CDCl_3_): δ_H_ 8.16 (d, *J* = 7.3 Hz, 1.34 H), 8.08 (d, *J* = 7.3 Hz, 0.66 H), 7.86 (d, *J* = 7.5 Hz, 0.33 H), 7.80 (d, *J* = 7.5 Hz, 0.67 H), 7.65–7.60 (m, 1.0 H), 7.52 (t, *J* = 7.7 Hz, 2.0 H), 7.49–7.46 (m, 1.33 H), 7.34 (d, *J* = 2.5 Hz, 0.67 H), 5.44 (dd, *J* = 11.3, 4.0 Hz, 0.67 H), 5.19 (dd, *J* = 11.3, 4.0 Hz, 0.33 H), 4.29–4.12 (m, 4.0 H), 3.59 (dd, *J* = 9.3, 5.3 Hz, 0.33 H), 3.44 (t, *J* = 7.3 Hz, 0.67 H), 3.27–3.21 (m, 1.0 H), 3.10–3.04 (m, 0.67 H), 2.83–2.68 (m, 0.67 H), 2.60–2.54 (m, 0.33 H), 2.47–2.42 (m, 0.67 H), 2.40–2.33 (m, 1.67 H), 1.34 (s, 9.0 H), 1.29–1.24 (m, 6.0 H); **^13^C NMR** (126 MHz, CDCl_3_): δ_c_ 189.8, 189.7, 169.0, 169.0, 168.9, 168.6, 157.0, 156.6, 139.3, 139.1, 136.4, 135.3, 135.2, 134.3, 134.2, 129.7, 129.5, 128.7, 128.7, 126.1, 125.6, 124.9, 124.8, 124.4, 124.0, 63.8, 61.9, 61.8, 61.8, 61.6, 50.0, 49.2, 35.3, 35.1, 34.9, 34.8, 34.3, 31.0, 30.9, 30.5, 29.1, 14.0, 14.0, 14.0; **IR**: ῡ = 1725, 1596, 1448, 1304, 1143, 732 cm^−^^1^; HRMS (ESI-TOF) *m*/*z* [M + Na]^+^ calcd. for C_28_H_34_NaO_7_S^+^, 537.1917, found 537.1929.

**Diethyl 2-((2-benzoyl-6-methoxy-1,1-dioxidothiochroman-4-yl)methyl)malonate (4c)**: Colorless oil (59.6 mg, 61% yield, dr = 2.0:1). **^1^H NMR** (500 MHz, CDCl_3_): δ_H_ 8.14 (d, *J* = 7.7 Hz, 1.34 H), 8.06 (d, *J* = 7.7 Hz, 0.66 H), 7.84 (d, *J* = 9.0 Hz, 0.33 H), 7.80 (d, *J* = 9.0 Hz, 0.67 H), 7.65–7.60 (m, 1.0 H), 7.53–7.48 (m, 2.0 H), 7.00 (d, *J* = 2.5 Hz, 0.33 H), 6.93 (dt, *J* = 7.7, 2.3 Hz, 1.0 H), 6.86 (d, *J* = 2.5 Hz, 0.67 H), 5.40 (dd, *J* = 10.3, 4.5 Hz, 0.67 H), 5.21 (dd, *J* = 10.3, 4.5 Hz, 0.33 H), 4.27–4.13 (m, 4.0 H), 3.88 (s, 1.0 H), 3.86 (s, 2.0 H), 3.60 (dd, *J* = 9.5, 5.5 Hz, 0.33 H), 3.47 (t, *J* = 7.3 Hz, 0.67 H), 3.28–3.21 (m, 1.0 H), 3.07–3.01 (m, 0.67 H), 2.74–2.67 (m, 0.67 H), 2.58–2.45 (m, 1.0 H), 2.38–2.28 (m, 1.67 H), 1.29–1.23 (m, 6.0 H); **^13^C NMR** (126 MHz, CDCl_3_): δ_c_ 190.1, 189.9, 169.0, 168.9, 168.9, 168.6, 163.3, 162.7, 142.3, 142.1, 136.4, 136.4, 134.3, 134.2, 129.7, 129.6, 129.5, 128.7, 127.0, 126.4, 114.1, 113.9, 112.9, 112.9, 63.8, 61.9, 61.8, 61.6, 55.6, 55.6, 49.9, 49.2, 34.7, 34.4, 34.1, 33.9, 30.7, 29.2, 14.0, 14.0, 14.0; **IR**: ῡ = 1684, 1595, 1448, 1294, 1134, 742 cm^−^^1^; HRMS (ESI-TOF) *m*/*z* [M + Na]^+^ calcd. for C_25_H_28_NaO_8_S^+^, 511.1397, found 511.1389.

**Diethyl 2-((2-benzoyl-6-bromo-1,1-dioxidothiochroman-4-yl)methyl)malonate (4d)**: Colorless oil (35.4 mg, 33% yield, dr = 2.0:1). **^1^H NMR** (500 MHz, CDCl_3_): δ_H_ 8.12 (d, *J* = 8.5 Hz, 1.34 H), 8.05 (d, *J* = 8.5 Hz, 0.66 H), 7.78 (d, *J* = 8.5 Hz, 0.33 H), 7.74 (d, *J* = 8.5 Hz, 0.67 H), 7.71 (s, 0.33 H), 7.67–7.62 (m, 1.0 H), 7.60–7.56 (m, 1.67 H), 7.54–7.49 (m, 2.0 H), 5.41 (dd, *J* = 10.7, 4.0 Hz, 0.67 H), 5.21 (dd, *J* = 10.7, 4.0 Hz, 0.33 H), 4.29–4.13 (m, 4.0 H), 3.59 (dd, *J* = 9.7, 5.3 Hz, 0.33 H), 3.45 (t, *J* = 7.3 Hz, 0.67 H), 3.29–3.23 (m, 1.0 H), 3.59 (dd, *J* = 9.7, 5.3 Hz, 0.33 H), 3.45 (t, *J* = 7.3 Hz, 0.67 H), 3.29–3.23 (m, 1.0 H), 3.05–2.99 (m, 0.67 H), 2.75–2.68 (m, 0.67 H), 2.61–2.56 (m, 0.33 H), 2.50–2.44 (m, 0.67 H), 2.41–2.24 (m, 1.67 H), 1.31–1.23 (m, 6.0 H); **^13^C NMR** (126 MHz, CDCl_3_): δ_c_ 189.5, 189.5, 168.8, 168.7, 168.5, 141.9, 141.8, 136.7, 136.1, 136.1, 134.5, 134.5, 132.2, 131.5, 131.0, 130.9, 129.6, 129.5, 128.8, 128.4, 127.7, 126.3, 125.9, 63.5, 62.0, 62.0, 61.3, 49.7, 49.0, 34.4, 34.3, 33.7, 30.3, 28.7, 14.1, 14.0, 14.0,14.0; **IR**: ῡ = 1723, 1682, 1581, 1447, 1148, 742 cm^−^^1^; HRMS (ESI-TOF) *m*/*z* [M + Na]^+^ calcd. for C_24_H_25_BrNaO_7_S^+^, 559.0397, found 559.0378.

**Diethyl 2-((2-benzoyl-1,1-dioxidothiochroman-4-yl)methyl)malonate (4e)**: Colorless oil (36.7 mg, 40% yield, dr = 2.0:1). **^1^H NMR** (500 MHz, CDCl_3_): δ_H_ 8.15 (d, *J* = 7.5 Hz, 1.34 H), 8.07 (d, *J* = 7.5 Hz, 0.66 H), 7.93 (d, *J* = 8.0 Hz, 0.33 H), 7.88 (d, *J* = 8.0 Hz, 0.67 H), 7.66–7.59 (m, 1.33 H), 7.57–7.48 (m, 3.0 H), 7.47–7.43 (m, 1.0 H), 7.40 (d, *J* = 8.0 Hz, 0.67 H), 5.44 (dd, *J* = 11.0, 4.0 Hz, 0.67 H), 5.21 (dd, *J* = 11.0, 4.0 Hz, 0.33 H), 4.28–4.12 (m, 4.0 H), 3.60 (dd, *J* = 9.7, 5.3 Hz, 0.33 H), 3.47 (t, *J* = 7.3 Hz, 0.67 H), 3.30–3.25 (m, 1.0 H), 3.09–3.03 (m, 0.67 H), 2.80–2.72 (m, 0.67 H), 2.61–2.56 (m, 0.33 H), 2.49–2.26 (m, 2.33 H), 1.30–1.22 (m, 6.0 H); **^13^C NMR** (126 MHz, CDCl_3_): δ_c_ 189.7, 189.7, 168.9, 168.9, 168.9, 168.6, 139.8, 139.7, 138.0, 138.0, 136.3, 134.4, 134.3, 133.3, 132.9, 129.7, 129.5, 129.5, 129.4, 128.8, 128.2, 128.0, 127.6, 124.6, 124.2, 63.6, 61.9, 61.8, 61.4, 49.9, 49.2, 34.7, 34.6, 34.4, 33.9, 30.3, 28.7, 14.0, 14.0, 14.0; **IR**: ῡ = 1724, 1683, 1595, 1448, 1294, 741 cm^−^^1^; HRMS (ESI-TOF) *m*/*z* [M + Na]^+^ calcd. for C_24_H_26_NaO_7_S^+^, 481.1291, found 481.1277.

**Diethyl 2-((2-benzoyl-8-methyl-1,1-dioxidothiochroman-4-yl)methyl)malonate (4f)**: Colorless oil (32.2 mg, 34% yield, dr = 4.0:1). **^1^H NMR** (500 MHz, CDCl_3_): δ_H_ 8.21 (d, *J* = 8.0 Hz, 1.6 H), 8.12 (d, *J* = 8.0 Hz, 0.4 H), 7.67–7.62 (m, 1.0 H), 7.57–7.50 (m, 2.2 H), 7.37 (t, *J* = 8.0 Hz, 1.0 H), 7.21–7.17 (m, 1.8 H), 5.53 (dd, *J* = 12.7, 2.7 Hz, 0.8 H), 5.17 (dd, *J* = 12.7, 2.7 Hz, 0.2 H), 4.28–4.11 (m, 4.0 H), 3.58–3.55 (m, 0.2 H), 3.43 (t, *J* = 7.5 Hz, 0.8 H), 3.21–3.16 (m, 1.0 H), 3.10–3.03 (m, 1.0 H), 2.70 (s, 0.6 H), 2.68 (s, 2.4 H), 2.41 (t, *J* = 7.5 Hz, 1.6 H), 2.30–2.26 (m, 1.0 H), 2.24–2.18 (m, 0.4 H), 1.31–1.23 (m, 6.0 H); **^13^C NMR** (126 MHz, CDCl_3_): δc 189.4, 169.0, 169.0, 168.6, 139.6, 137.5, 137.0, 136.5, 134.4, 132.1, 132.0, 131.9, 129.9, 129.8, 129.7, 128.7, 128.7, 127.6, 65.0, 62.8, 62.0, 61.9, 61.9, 61.8, 50.1, 49.9, 35.8, 35.4, 34.5, 34.1, 27.5, 20.2, 14.1, 14.0, 14.0; HRMS (ESI-TOF) *m*/*z* [M + Na]^+^ calcd. for C25H28NaO7S+, 495.1448, found 495.1427.

**Diethyl 2-((2-benzoyl-8-methyl-1,1-dioxidothiochroman-4-yl)methyl)malonate (4g)**: Colorless oil (40.7 mg, 43% yield, dr = 2.0:1). **^1^H NMR** (500 MHz, CDCl_3_): δ_H_ 8.20 (d, *J* = 7.7 Hz, 1.34 H), 8.15 (d, *J* = 7.7 Hz, 0.66 H), 7.75 (d, *J* = 7.7 Hz, 0.66 H), 7.68–7.62 (m, 1.34 H), 7.56–7.49 (m, 2.0 H), 7.42–7.39 (m, 0.67 H), 7.34 (d, *J* = 7.7 Hz, 1.0 H), 7.28 (d, *J* = 7.7 Hz, 0.33 H), 5.55 (dd, *J* = 12.3, 3.3 Hz, 0.67 H), 5.42 (dd, *J* = 10.7, 3.3 Hz, 0.33 H), 4.26–4.09 (m, 4.0 H), 3.47–3.44 (m, 1.0 H), 3.37–3.34 (m, 0.67 H), 3.24–3.20 (m, 0.33 H), 3.08–2.97 (m, 1.0 H), 2.78–2.71 (m, 0.33 H), 2.44 (s, 2.0 H), 2.41–2.31 (m, 3.67 H), 1.30–1.20 (m, 6.0 H); **^13^C NMR** (126 MHz, CDCl_3_): δ_c_ 189.8, 189.5, 169.0, 168.9, 168.7, 168.5, 138.8, 138.3, 136.5, 136.5, 135.0, 134.4, 133.8, 129.7, 129.7, 129.6, 129.3, 128.8, 128.7, 128.0, 124.2, 122.2, 62.0, 61.9, 61.9, 61.8, 61.3, 61.2, 50.0, 49.9, 34.5, 34.2, 33.7, 32.1, 31.0, 28.9, 26.9, 21.0, 19.1, 14.0, 14.0, 14.0, 13.9; HRMS (ESI-TOF) *m*/*z* [M + Na]^+^ calcd. for C_25_H_28_NaO_7_S^+^, 495.1448, found 495.1420.

**Diethyl 2-((6-methyl-2-(4-methylbenzoyl)-1,1-dioxidothiochroman-4-yl)methyl)malonate (4h)**: Colorless oil (66.2 mg, 68% yield, dr = 2.0:1). **^1^H NMR** (500 MHz, CDCl_3_): δ_H_ 8.04 (d, *J* = 8.3 Hz, 1.34 H), 7.97 (d, *J* = 8.3 Hz, 0.66 H), 7.81(d, *J* = 8.3 Hz, 0.33 H), 7.76 (d, *J* = 8.3 Hz, 0.67 H), 7.32–7.28 (m, 2.33 H), 7.25–7.22 (m, 1.0 H), 7.18 (s, 0.67 H), 5.38 (dd, *J* = 10.7, 4.0 Hz, 0.67 H), 5.16 (dd, *J* = 10.7, 4.0 Hz, 0.33 H), 4.29–4.12 (m, 4.0 H), 3.61 (dd, *J* = 10.0, 5.0 Hz, 0.33 H), 3.46 (t, *J* = 7.5 Hz, 0.67 H), 3.24–3.20 (m, 1.0 H), 3.06–3.00 (m, 0.67 H), 2.79–2.69 (m, 0.67 H), 2.58–2.53 (m, 0.33 H), 2.49–2.44 (m, 0.67 H), 2.43 (s, 3.0 H), 2.42 (s, 1.0 H), 2.41 (s, 2.0 H), 2.38–2.30 (m, 1.67 H), 1.31–1.23 (m, 6.0 H); **^13^C NMR** (126 MHz, CDCl_3_): δ_c_ 189.2, 169.0, 168.9, 168.7, 145.5, 145.5, 144.0, 143.6, 139.7, 135.2, 135.1, 133.9, 133.9, 129.8, 129.7, 129.7, 129.4, 129.4, 128.9, 128.4, 128.3, 124.6, 124.2, 63.4, 61.9, 61.8, 61.2, 49.9, 49.1, 34.7, 34.5, 34.5, 33.8, 30.2, 28.7, 21.8, 21.7, 21.6, 14.1, 14.0, 14.0, 14.0; **IR**: ῡ = 1725, 1678, 1603, 1447, 1136, 729 cm^−^^1^; HRMS (ESI-TOF) *m*/*z* [M + Na]^+^ calcd. for C_26_H_30_NaO_7_S^+^, 509.1604, found 509.1606.

**Diethyl 2-((2-(4-methoxybenzoyl)-6-methyl-1,1-dioxidothiochroman-4-yl)methyl)malonate (4i)**: Colorless oil (62.5 mg, 62% yield, dr = 2.0:1). **^1^H NMR** (500 MHz, CDCl_3_): δ_H_ 8.13 (d, *J* = 9.0 Hz, 1.34 H), 8.05 (d, *J* = 8.7 Hz, 0.66 H), 7.80 (d, *J* = 8.0 Hz, 0.33 H), 7.75 (d, *J* = 8.0 Hz, 0.67 H), 7.32 (s, 0.33 H), 7.23 (d, *J* = 8.0 Hz, 1.0 H), 7.17 (s, 0.67 H), 6.99–6.94 (m, 2.0 H), 5.34 (dd, *J* = 10.7, 3.3 Hz, 0.67 H), 5.12 (dd, *J* = 11.3, 4.3 Hz, 0.33 H), 4.26–4.11 (m, 4.0 H), 3.88 (s, 2.0 H), 3.86 (s, 1.0 H), 3.61 (dd, *J* = 10.0, 5.0 Hz, 0.33 H), 3.46 (t, *J* = 7.3 Hz, 0.67 H), 3.23–3.17 (m, 1.0 H), 3.05–2.99 (m, 0.67 H), 2.78–2.69 (m, 0.67 H), 2.58–2.53 (m, 0.33 H), 2.48–2.44 (m, 0.67 H), 2.43 (s, 1.0 H), 2.41 (s, 2.0 H), 2.37–2.22 (m, 1.67 H), 1.29–1.22 (m, 6.0 H); **^13^C NMR** (126 MHz, CDCl_3_): δ_c_ 187.8, 187.7, 169.0, 168.9, 168.7, 168.7, 164.5, 164.5, 143.9, 143.5, 139.7, 139.7, 135.3, 135.1, 132.2, 132.1, 129.7, 129.5, 129.4, 128.9, 128.4, 128.2, 124.6, 124.2, 113.9, 113.9, 63.3, 61.8, 61.8, 61.0, 55.5, 49.9, 49.1, 34.5, 33.8, 30.2, 28.6, 21.7, 21.5, 14.0, 14.0, 14.0, 14.0; **IR**: ῡ = 1743, 1721, 1598, 1134, 1023, 687 cm^−^^1^; HRMS (ESI-TOF) *m*/*z* [M + Na]^+^ calcd. for C_26_H_30_NaO_8_S^+^, 525.1554, found 525.1534.

**Diethyl 2-((2-(4-bromobenzoyl)-6-methyl-1,1-dioxidothiochroman-4-yl)methyl)malonate (4j)**: Colorless oil (36.4 mg, 33% yield, dr = 2.0:1). **^1^H NMR** (500 MHz, CDCl_3_): δ_H_ 8.01 (d, *J* = 8.7 Hz, 1.34 H), 7.94 (d, *J* = 8.5 Hz, 0.66 H), 7.80 (d, *J* = 8.0 Hz, 0.33 H), 7.75 (d, *J* = 8.0 Hz, 0.67 H), 7.66 (d, *J* = 8.7 Hz, 1.34 H), 7.64 (d, *J* = 8.7 Hz, 0.66 H), 7.33 (s, 0.33 H), 7.24 (d, *J* = 8.0 Hz, 1.0 H), 7.17 (s, 0.67 H), 5.36 (dd, *J* = 10.7, 3.3 Hz, 0.67 H), 5.12 (dd, *J* = 11.0, 4.5 Hz, 0.33 H), 4.28–4.12 (m, 4.0 H), 3.60 (dd, *J* = 10.0, 5.0 Hz, 0.33 H), 3.46 (t, *J* = 7.3 Hz, 0.67 H), 3.25–3.19 (m, 1.0 H), 3.05–2.99 (m, 0.67 H), 2.79–2.69 (m, 0.67 H), 2.58–2.53 (m, 0.33 H), 2.47–2.41 (m, 3.67 H), 2.36–2.23 (m, 1.67 H), 1.30–1.23 (m, 6.0 H); **^13^C NMR** (126 MHz, CDCl_3_): δ_c_ 189.0, 188.9, 169.0, 168.9, 168.6, 144.2, 143.8, 139.7, 139.6, 135.1, 135.0, 134.9, 132.1, 131.1, 131.0, 130.0, 129.9, 129.8, 129.1, 128.4, 128.4, 124.7, 124.2, 63.8, 61.9, 61.8, 61.5, 50.0, 49.2, 34.7, 34.4, 33.7, 30.1, 28.5, 21.8, 21.6, 14.1, 14.0, 14.0, 14.0; **IR**: ῡ = 1724, 1683, 1583, 1447, 1137, 732 cm^−^^1^; HRMS (ESI-TOF) *m*/*z* [M + Na]^+^ calcd. for C_25_H_27_BrNaO_7_S^+^, 573.0553, found 573.0548.

**Diethyl 2-((6-fluoro-2-(4-methylbenzoyl)-1,1-dioxidothiochroman-4-yl)methyl)malonate (4k)**: Colorless oil (57.1 mg, 58% yield, dr = 2.0:1). **^1^H NMR** (500 MHz, CDCl_3_): δ_H_ 8.01 (d, *J* = 8.3 Hz, 1.34 H), 7.95 (d, *J* = 8.3 Hz, 0.66 H), 7.93–7.87 (m, 1.0 H), 7.32–7.28 (m, 2.0 H), 7.27–7.24 (m, 0.33 H), 7.15–7.11 (m, 1.67 H), 5.37 (dd, *J* = 10.0, 4.0 Hz, 0.67 H), 5.20 (dd, *J* = 10.0, 4.0 Hz, 0.33 H), 4.29–4.13 (m, 4.0 H), 3.59 (dd, *J* = 9.5, 5.0 Hz, 0.33 H), 3.46 (t, *J* = 7.3 Hz, 0.67 H), 3.31–3.23 (m, 1.0 H), 3.05–2.99 (m, 0.67 H), 2.74–2.67 (m, 0.67 H), 2.61–2.56 (m, 0.33 H), 2.49–2.24 (m, 5.33 H), 1.31–1.23 (m, 6.0 H); **^13^C NMR** (126 MHz, CDCl_3_): δ_c_ 189.0, 189.0, 168.8, 168.7, 168.7, 168.5, 165.3 (d, *J* = 255.8 Hz), 164.7 (d, *J* = 255.8 Hz), 145.7, 145.7, 143.5 (d, *J* = 8.2 Hz), 143.4 (d, *J* = 8.2 Hz), 133.8 (d, *J* = 3.3 Hz), 133.8, 133.7, 129.8, 129.7, 129.5, 127.7 (d, *J* = 9.5 Hz), 127.2 (d, *J* = 9.5 Hz), 116.1 (d, *J* = 22.7 Hz), 115.8 (d, *J* = 22.7 Hz), 115.1 (d, *J* = 23.5 Hz), 114.9 (d, *J* = 23.5 Hz), 63.4, 62.0, 61.9, 61.2, 49.7, 49.1, 34.5, 34.5, 34.1, 33.9, 30.4, 28.9, 21.8, 21.7, 14.0, 14.0, 14.0, 14.0; **IR**: ῡ = 1725, 1678, 1605, 1305, 1148, 732 cm^−^^1^; **^19^F NMR** (470 MHz, CDCl_3_): δ_F_ −103.4, −104.2 (s) ppm; HRMS (ESI-TOF) *m*/*z* [M + Na]^+^ calcd. for C_25_H_27_FNaO_7_S^+^, 513.1354, found 513.1349.

**Diethyl 2-((6-fluoro-2-(4-methoxybenzoyl)-1,1-dioxidothiochroman-4-yl)methyl)malonate (4l)**: Colorless oil (56.8 mg, 56% yield, dr = 2.0:1). **^1^H NMR** (500 MHz, CDCl_3_): δ_H_ 8.09 (d, *J* = 8.7 Hz, 1.34 H), 8.02 (d, *J* = 8.7 Hz, 0.66 H), 7.93–7.86 (m, 1.0 H), 7.26–7.23 (m, 0.33 H), 7.14–7.10 (m, 1.67 H), 6.97–6.93 (m, 2.0 H), 5.33 (dd, *J* = 10.3, 4.3 Hz, 0.66 H), 5.16 (dd, *J* = 10.3, 4.3 Hz, 0.34 H), 4.26–4.12 (m, 4.0 H), 3.87 (s, 2.0 H), 3.86 (s, 1.0 H), 3.58 (dd, *J* = 9.7, 5.3 Hz, 0.33 H), 3.47–3.44 (m, 0.67 H), 3.30–3.21 (m, 1.0 H), 3.04–2.98 (m, 0.67 H), 2.73–2.66 (m, 0.67 H), 2.59–2.54 (m, 0.33 H), 2.48–2.22 (m, 2.33 H), 1.29–1.22 (m, 6.0 H); **^13^C NMR** (126 MHz, CDCl_3_): δ_c_ 187.6, 187.5, 168.8, 168.7, 168.7, 168.5, 164.7, 164.6, 165.2 (d, *J* = 255.7 Hz), 164.7 (d, *J* = 255.4 Hz), 143.5 (d, *J* = 8.3 Hz), 143.4 (d, *J* = 9.5 Hz), 134.0 (d, *J* = 3.0 Hz), 133.9 (d, *J* = 2.9 Hz), 132.1, 132.0, 129.3, 129.2, 127.6 (d, *J* = 9.5 Hz), 127.2 (d, *J* = 9.5 Hz), 116.0 (d, *J* = 22.8 Hz), 115.7 (d, *J* = 22.9 Hz), 115.2, 114.8 (d, *J* = 22.9 Hz), 114.0, 114.0, 63.2, 61.9, 61.9, 60.9, 55.5, 49.7, 49.1, 34.6, 34.5, 34.2, 33.9, 30.3, 28.8, 14.0, 14.0, 13.9, 13.9; **IR**: ῡ = 1724, 1672, 1597, 1172, 1026, 731 cm^−^^1^; **^19^F NMR** (470 MHz, CDCl_3_): δ_F_ −103.5, −104.3 (s) ppm; HRMS (ESI-TOF) *m*/*z* [M + Na]^+^ calcd. for C_25_H_27_FNaO_8_S^+^, 529.1303, found 529.1295.

**Diethyl 2-((6-bromo-2-(4-methoxybenzoyl)-1,1-dioxidothiochroman-4-yl)methyl)malonate (4m)**: Colorless oil (62.3 mg, 55% yield, dr = 2.0:1). **^1^H NMR** (500 MHz, CDCl_3_): δ_H_ 8.08 (d, *J* = 8.7 Hz, 1.34 H), 8.01 (d, *J* = 8.7 Hz, 0.66 H), 7.75 (d, *J* = 8.7 Hz, 0.33 H), 7.71 (d, *J* = 8.7 Hz, 0.67 H), 7.68 (s, 0.33 H), 7.57–7.54 (m, 1.67 H), 6.97–6.93 (m, 2.0 H), 5.34 (dd, *J* = 10.0, 3.5 Hz, 0.67 H), 5.14 (dd, *J* = 10.7, 4.7 Hz, 0.33 H), 4.28–4.12 (m, 4.0 H), 3.87 (s, 2.0 H), 3.86 (s, 1.0 H), 3.58 (dd, *J* = 9.7, 4.7 Hz, 0.33 H), 3.45 (t, *J* = 7.5 Hz, 0.67 H), 3.27–3.20 (m, 1.0 H), 3.02–2.96 (m, 0.67 H), 2.73–2.66 (m, 0.67 H), 2.58–2.53 (m, 0.33 H), 2.47–2.41 (m, 0.67 H), 2.38–2.22 (m, 1.67 H), 1.28–1.22 (m, 6.0 H); **^13^C NMR** (126 MHz, CDCl_3_): δ_c_ 187.4, 168.8, 168.7, 168.7, 168.4, 164.6, 164.6, 141.9, 141.9, 136.9, 136.8, 132.2, 132.1, 132.0, 131.3, 130.9, 130.8, 129.2, 129.1, 128.2, 127.5, 126.2, 125.8, 114.0, 114.0, 63.1, 61.9, 61.9, 60.8, 55.5, 49.7, 49.0, 34.4, 34.3, 34.3, 33.8, 30.1, 28.6, 14.0, 14.0, 13.9, 13.9; **IR**: ῡ = 1737, 1674, 1599, 1258, 1138, 840 cm^−^^1^; HRMS (ESI-TOF) *m*/*z* [M + Na]^+^ calcd. for C_25_H_27_BrNaO_8_S^+^, 589.0502, found 589.0521.

**Diethyl 2-((2-(4-fluorobenzoyl)-6-methoxy-1,1-dioxidothiochroman-4-yl)methyl)malonate (4n)**: Colorless oil (58.9 mg, 58% yield, dr = 2.0:1). **^1^H NMR** (500 MHz, CDCl_3_): δ_H_ 8.18 (dd, *J* = 8.7, 5.3 Hz, 1.34 H), 8.10 (dd, *J* = 8.7, 5.3 Hz, 0.66 H), 7.82 (d, *J* = 8.7 Hz, 0.33 H), 7.78 (d, *J* = 8.7 Hz, 0.67 H), 7.20–7.14 (m, 2.0 H), 7.00 (d, *J* = 1.7 Hz, 0.33H), 6.94–6.91 (m, 1.0 H), 6.85 (d, *J* = 1.7 Hz, 0.67 H), 5.35 (dd, *J* = 10.5, 4.3 Hz, 0.67 H), 5.15 (dd, *J* = 10.5, 4.3 Hz, 0.33 H), 4.27–4.12 (m, 4.0 H), 3.88 (s, 1.0 H), 3.86 (s, 2.0 H), 3.59 (dd, *J* = 9.5, 5.5 Hz, 0.33 H), 3.47 (t, *J* = 7.3 Hz, 0.67 H), 3.24–3.22 (m, 1.0 H), 3.05–2.99 (m, 0.67 H), 2.73–2.66 (m, 0.67 H), 2.57–2.52 (m, 0.33 H), 2.48–2.42 (m, 0.67 H), 2.37–2.27 (m, 1.67 H), 1.30–1.22 (m, 6.0 H); **^13^C NMR** (126 MHz, CDCl_3_): δ_c_ 188.4, 188.3, 169.0, 168.9, 168.9, 168.6, 166.5 (d, *J* = 258.0 Hz), 163.4 (d, *J* = 258.0 Hz), 163.3, 162.8, 142.2, 142.1, 132.9 (d, *J* = 2.9 Hz), 132.8 (d, *J* = 2.9 Hz), 132.5 (d, *J* = 9.6 Hz), 132.4 (d, *J* = 9.6 Hz), 129.6, 129.5, 127.0, 126.4, 115.9 (d, *J* = 22.1 Hz), 114.0 (d, *J* = 30.9 Hz), 112.9 (d, *J* = 5.3 Hz), 63.8, 61.9, 61.9, 61.8, 61.6, 55.6, 55.6, 49.9, 49.2, 34.8, 34.3, 34.2, 33.9, 30.5, 29.0, 14.0, 14.0, 14.0; **IR**: ῡ = 1682,1595, 1448, 1294, 1134, 730 cm^−^^1^; **^19^F NMR** (470 MHz, CDCl_3_): δ_F_ −102.8 (s) ppm; HRMS (ESI-TOF) *m*/*z* [M + Na]^+^ calcd. for C_25_H_27_FNaO_8_S^+^, 529.1303, found 529.1306.

**Diethyl 2-((2-(4-bromobenzoyl)-6-methoxy-1,1-dioxidothiochroman-4-yl)methyl)malonate (4o)**: Colorless oil (33.0 mg, 29% yield, dr = 2.0:1). **^1^H NMR** (500 MHz, CDCl_3_): δ_H_ 8.01 (d, *J* = 8.7 Hz, 1.34 H), 7.93 (d, *J* = 8.7 Hz, 0.66 H), 7.83 (d, *J* = 8.7 Hz, 0.33 H), 7.79 (d, *J* = 8.7 Hz, 0.67 H), 7.67–7.63 (m, 2.0 H), 7.01 (s, 0.33 H), 6.93 (dd, *J* = 8.7, 2.3 Hz, 1.0 H), 6.85 (d, *J* = 2.3 Hz, 0.67 H), 5.34 (dd, *J* = 10.0, 3.5 Hz, 0.67 H), 5.14 (dd, *J* = 10.5, 5.0 Hz, 0.33 H), 4.28–4.11 (m, 4.0 H), 3.89 (s, 1.0 H), 3.87 (s, 2.0 H), 3.60 (dd, *J* = 9.0, 5.5 Hz, 0.33 H), 3.47 (t, *J* = 7.3 Hz, 0.67 H), 3.25–3.23 (m, 1.0 H), 3.06–3.00 (m, 0.67 H), 2.74–2.66 (m, 0.67 H), 2.57–2.52 (m, 0.33 H), 2.49–2.43 (m, 0.67 H), 2.37–2.28 (m, 1.67 H), 1.31–1.22 (m, 6.0 H); **^13^C NMR** (126 MHz, CDCl_3_): δ_c_ 189.1, 189.1, 169.0, 168.9, 168.6, 163.4, 162.8, 142.3, 142.1, 135.1, 135.1, 132.1, 131.1, 131.0, 129.9, 129.6, 129.4, 127.1, 126.5, 114.2, 113.9, 113.0, 112.9, 64.0, 61.9, 61.9, 61.7, 55.7, 55.6, 50.0, 49.2, 34.9, 34.4, 34.1, 33.9, 30.5, 29.0, 14.1, 14.0, 14.0; **IR**: ῡ = 1732, 1664, 1592, 1248, 1131, 848 cm^−^^1^; HRMS (ESI-TOF) *m*/*z* [M + Na]^+^ calcd. for C_25_H_27_BrNaO_8_S^+^, 589.0502, found 589.0515.

**Diethyl 2-((6-fluoro-2-(3-methoxybenzoyl)-1,1-dioxidothiochroman-4-yl)methyl)malonate (4p)**: Colorless oil (29.6 mg, 29% yield, dr = 2.0:1). **^1^H NMR** (500 MHz, CDCl_3_): δ_H_ 7.95–7.88 (m, 1.0 H), 7.73 (d, *J* = 8.0 Hz, 0.67 H), 7.64 (d, *J* = 8.0 Hz, 0.33 H), 7.60 (s, 0.67 H), 7.55 (s, 0.33 H), 7.45–7.40 (m, 1.0 H), 7.27–7.25 (m, 0.67 H), 7.19 (dd, *J* = 8.5, 2.5 Hz, 0.67 H), 7.17–7.12 (m, 1.67 H), 5.37 (dd, *J* = 9.7, 3.7 Hz, 0.67 H), 5.21 (dd, *J* = 10.5, 5.0 Hz, 0.33 H), 4.29–4.13 (m, 4.0 H), 3.86 (s, 2.0 H), 3.85 (s, 1.0 H), 3.59 (dd, *J* = 9.7, 5.3 Hz, 0.33 H), 3.46 (t, *J* = 7.3 Hz, 0.67 H), 3.31–3.25 (m, 1.0 H), 3.06–3.00 (m, 0.67 H), 2.74–2.66 (m, 0.67 H), 2.62–2.57 (m, 0.33 H), 2.50–2.24 (m, 2.33 H), 1.31–1.23 (m, 6.0 H); **^13^C NMR** (126 MHz, CDCl_3_): δ_c_ 189.6, 189.5, 168.8, 168.7, 168.7, 168.5, 165.4 (d, *J* = 255.9 Hz), 164.8 (d, *J* = 255.9 Hz), 159.9, 143.5 (d, *J* = 8.2 Hz), 143.3 (d, *J* = 8.2 Hz), 137.5, 137.4, 133.8 (d, *J* = 3.2 Hz), 133.8 (d, *J* = 3.2 Hz), 129.8, 129.8, 127.8 (d, *J* = 9.6 Hz), 127.3 (d, *J* = 9.6 Hz), 122.5, 122.4, 121.4, 121.2, 116.1 (d, *J* = 22.7 Hz), 115.8 (d, *J* = 22.7 Hz), 115.1 (d, *J* = 23.1 Hz), 114.9 (d, *J* = 23.1 Hz), 113.2, 113.2, 63.7, 62.0, 61.9, 61.6, 55.5, 49.7, 49.1, 34.5, 34.4, 34.0, 33.8, 30.6, 29.1, 14.0, 14.0, 14.0, 14.0; **^19^F NMR** (470 MHz, CDCl_3_): δ_F_ −103.2, −104.1 (s) ppm; **IR**: ῡ = 1724, 1682, 1580, 1146, 1026, 730 cm^−^^1^; HRMS (ESI-TOF) *m*/*z* [M + Na]^+^ calcd. for C_25_H_27_FNaO_8_S^+^, 529.1303, found 529.1291.

**Diethyl 2-((6-fluoro-2-(2-methoxybenzoyl)-1,1-dioxidothiochroman-4-yl)methyl)malonate (4q)**: Colorless oil (54.8 mg, 54% yield, dr = 2.0:1). **^1^H NMR** (500 MHz, CDCl_3_): δ_H_ 7.90–7.83 (m, 1.0 H), 7.68 (dd, *J* = 7.7, 1.7 Hz, 0.33 H), 7.60 (dd, *J* = 7.7, 1.7 Hz, 0.67 H), 7.52 (td, *J* = 7.7, 1.7 Hz, 1.0 H), 7.23 (dd, *J* = 10.0, 1.5 Hz, 0.33 H), 7.14–7.08 (m, 1.67 H), 7.04–6.99 (m, 2.0 H), 5.77 (dd, *J* = 9.0, 3.5 Hz, 0.67 H), 5.60 (dd, *J* = 10.0, 6.0 Hz, 0.33 H), 4.29–4.17 (m, 4.0 H), 3.96 (s, 2.0 H), 3.93 (s, 1.0 H), 3.59 (dd, *J* = 9.3, 5.7 Hz, 0.33 H), 3.49 (dd, *J* = 9.0, 6.0 Hz, 0.67 H), 3.31–3.20 (m, 1.0 H), 2.96–2.90 (m, 0.67 H), 2.72–2.66 (m, 0.67 H), 2.54–2.47 (m, 1.67 H), 2.33–2.22 (m, 1.0 H), 1.32–1.25 (m, 6.0 H); **^13^C NMR** (126 MHz, CDCl_3_): δ_c_ 192.1, 192.1, 168.8, 168.8, 168.6, 165.3 (d, *J* = 255.5 Hz), 164.7 (d, *J* = 255.5 Hz), 158.8, 158.7, 143.9 (d, *J* = 8.4 Hz), 143.3 (d, *J* = 8.4 Hz), 135.1, 134.9, 134.2 (d, *J* = 3.2 Hz), 133.7 (d, *J* = 3.2 Hz), 131.1, 130.9, 127.7 (d, *J* = 9.6 Hz), 127.5, 127.2, 126.9 (d, *J* = 9.6 Hz), 121.2, 116.1 (d, *J* = 22.7 Hz), 115.5 (d, *J* = 22.7 Hz), 114.8 (d, *J* = 22.8 Hz), 114.3 (d, *J* = 22.8 Hz), 111.7, 111.7, 68.0, 65.1, 61.9, 61.9, 55.9, 55.8, 49.5, 49.2, 34.9, 34.0, 33.4, 33.3, 30.8, 28.5, 14.0, 14.0, 14.0, 14.0; **^19^F NMR** (470 MHz, CDCl_3_): δ_F_ −103.7, −104.7 (s) ppm; **IR**: ῡ = 1726, 1597, 1299, 1147, 1020, 730 cm^−^^1^; HRMS (ESI-TOF) *m*/*z* [M + Na]^+^ calcd. for C_25_H_27_FNaO_8_S^+^, 529.1303, found 529.1319.

**Diethyl 2-((2-benzoyl-6-methyl-1,1-dioxidothiochroman-4-yl)methyl)-2-methylmalonate (5ab)**: Colorless oil (43.7 mg, 45% yield, dr = 2.0:1). **^1^H NMR** (500 MHz, CDCl_3_): δ_H_ 8.18 (dd, *J* = 8.3, 1.3 Hz, 1.34 H), 8.06 (dd, *J* = 8.3, 1.3 Hz, 0.66 H), 7.79 (d, *J* = 8.3 Hz, 0.33 H), 7.75 (d, *J* = 8.3 Hz, 0.67 H), 7.65–7.60 (m, 1.0 H), 7.55–7.48 (m, 2.0 H), 7.33 (s, 0.33 H), 7.23 (d, *J* = 8.3 Hz, 1.0 H), 7.12 (s, 0.67 H), 5.59 (dd, *J* = 11.5, 3.7 Hz, 0.67 H), 5.17 (dd, *J* = 11.5, 3.7 Hz, 0.33 H), 4.26–4.00 (m, 4.0 H), 3.38–3.31 (m, 1.0 H), 2.99–2.93 (m, 0.67 H), 2.74–2.67 (m, 0.33 H), 2.60–2.49 (m, 1.0 H), 2.44 (s, 1.0 H), 2.40 (s, 2.0 H), 2.36–2.21 (m, 2.0 H), 1.57 (s, 1.0 H), 1.56 (s, 2.0 H), 1.30–1.24 (m, 3.0 H), 1.23–1.16 (m, 3.0 H); **^13^C NMR** (126 MHz, CDCl_3_): δ_c_190.0, 189.9, 172.2, 172.1, 172.1, 171.9, 144.0, 143.5, 141.3, 140.6, 136.7, 136.4, 135.7, 135.0, 134.2, 133.6, 129.8, 129.7, 129.5, 129.0, 128.7, 128.7, 128.3, 128.1, 124.7, 124.1, 63.9, 61.8, 61.7, 61.5, 53.3, 53.1, 41.7, 40.4, 33.4, 32.7, 31.9, 29.7, 28.3, 21.8, 21.5, 21.1, 20.5, 14.0, 13.9, 13.9, 13.8; **IR**: ῡ = 1723, 1682, 1448, 1108, 911, 728 cm^−^^1^; HRMS (ESI-TOF) *m*/*z* [M + Na]^+^ calcd. for C_26_H_30_NaO_7_S^+^, 509.1604, found 509.1601.

**Dmethyl 2-((2-benzoyl-6-methyl-1,1-dioxidothiochroman-4-yl)methyl)malonate (5ac)**: Colorless oil (41.9 mg, 47% yield, dr = 2.0:1). ^1^H NMR (500 MHz, CDCl_3_): δ_H_ 8.15 (d, *J* = 7.7 Hz, 1.34 H), 8.08 (d, *J* = 7.7 Hz, 0.66 H), 7.82 (d, *J* = 7.7 Hz, 0.33 H), 7.77 (d, *J* = 7.7 Hz, 0.67 H), 7.67–7.61 (m, 1.0 H), 7.55–7.49 (m, 2.0 H), 7.32 (s, 0.33 H), 7.26–7.24 (m, 1.0 H), 7.17 (s, 0.67 H), 5.40 (dd, *J* = 10.7, 4.0 Hz, 0.67 H), 5.19 (dd, *J* = 10.7, 4.0 Hz, 0.33 H), 3.80 (s, 1.0 H), 3.76 (s, 2.0 H), 3.74 (s, 1.0 H), 3.71 (s, 2.0 H), 3.68–3.65 (m, 0.33 H), 3.52 (t, *J* = 7.3 Hz, 0.67 H), 3.25–3.18 (m, 1.0 H), 3.07–2.99 (m, 0.67 H), 2.80–2.73 (m, 0.67 H), 2.58–2.53 (m, 0.33 H), 2.52–2.47 (m, 0.67 H), 2.45 (s, 1.0 H), 2.42 (s, 2.0 H), 2.38–2.28 (m, 1.67 H); ^13^C NMR (126 MHz, CDCl_3_): δ_c_ 189.9 189.8, 169.3, 169.3, 169.0, 144.2, 143.7, 139.6, 136.4, 136.3, 135.1, 135.0, 134.4, 134.3, 129.7, 129.7, 129.6, 129.1, 128.8, 128.8, 128.4, 128.4, 124.7, 124.3, 63.6, 61.5, 52.9, 52.9, 52.9, 52.8, 49.6, 48.8, 34.8, 34.6, 34.5, 33.7, 30.3, 28.8, 21.8, 21.6; HRMS (ESI-TOF) *m*/*z* [M + Na]+ calcd. for C_23_H_24_NaO_7_S^+^, 467.1135, found 467.1119.

**3-(2-Benzoyl-6-methyl-1,1-dioxidothiochroman-4-yl)-1-phenylpropan-1-one (5af)**: Colorless oil (49.3 mg, 57% yield, dr = 1.5:1). **^1^H NMR** (500 MHz, CDCl_3_): δ_H_ 8.14 (d, *J* = 7.7 Hz, 1.2 H), 8.08 (d, *J* = 7.7 Hz, 0.8 H), 7.94 (d, *J* = 7.7 Hz, 0.8 H), 7.90 (d, *J* = 7.7 Hz, 1.2 H), 7.82 (d, *J* = 7.7 Hz, 0.4 H), 7.76 (d, *J* = 7.7 Hz, 0.6 H), 7.64–7.59 (m, 1.0 H), 7.55 (t, *J* = 7.7 Hz, 1.0 H), 7.52–7.48 (m, 2.0 H), 7.46–7.42 (m, 2.0 H), 7.32 (s, 0.4 H), 7.23–7.20 (m, 1.6 H), 5.45 (dd, *J* = 9.7, 3.7 Hz, 0.6 H), 5.24 (dd, *J* = 11.5, 3.7 Hz, 0.4 H), 3.40–3.31 (m, 1.0 H), 3.22–2.97 (m, 3.0 H), 2.56–2.43 (m, 1.2 H), 2.41 (s, 1.2 H), 2.38 (s, 1.8 H), 2.33–2.20 (m, 1.8 H); **^13^C NMR** (126 MHz, CDCl_3_): δ_c_ 199.3, 199.1, 190.2, 190.0, 144.0, 143.6, 140.7, 139.9, 136.5, 136.5, 136.4, 136.3, 135.6, 134.8, 134.2, 133.3, 133.2, 129.6, 129.5, 129.5, 128.7, 128.7, 128.6, 128.6, 128.3, 128.2, 127.9, 127.9, 124.4, 124.1, 63.9, 61.6, 35.5, 35.3, 35.0, 34.2, 30.3, 29.1, 28.9, 28.7, 21.7, 21.5; **IR**: ῡ = 1679, 1596, 1447, 1298, 1133, 729 cm^−^^1^; HRMS (ESI-TOF) *m*/*z* [M + Na]^+^ calcd. for C_26_H_24_NaO_4_S^+^, 455.1288, found 455.1264.

**3-(2-Benzoyl-6-methyl-1,1-dioxidothiochroman-4-yl)-1-(4-methoxyphenyl)propan-1-one (5ag)**: Colorless oil (58.3 mg, 63% yield, dr = 1.5:1). **^1^H NMR** (500 MHz, CDCl_3_): δ_H_ 8.12 (d, *J* = 8.0 Hz, 1.2 H), 8.06 (d, *J* = 8.0 Hz, 0.8 H), 7.89 (d, *J* = 8.0 Hz, 0.8 H), 7.86 (d, *J* = 8.0 Hz, 1.2 H), 7.78 (d, *J* = 8.0 Hz, 0.4 H), 7.72 (d, *J* = 8.0 Hz, 0.6 H), 7.58 (q, *J* = 8.0 Hz, 1.0 H), 7.48–7.42 (m, 2.0 H), 7.30 (s, 0.6 H), 7.20–7.17 (m, 1.4 H), 6.90–6.86 (m, 2.0 H), 5.46 (dd, *J* = 10.0, 3.7 Hz, 0.6 H), 5.25 (dd, *J* = 11.5, 3.7 Hz, 0.4 H), 3.81 (s, 1.2 H), 3.81 (s, 1.8 H), 3.36–3.26 (m, 1.0 H), 3.12–2.91 (m, 2.6 H), 2.84–2.77 (m, 0.4 H), 2.55–2.40 (m, 1.4 H), 2.38 (s, 1.2 H), 2.36 (s, 1.8 H), 2.28–2.17 (m, 1.6 H); **^13^C NMR** (126 MHz, CDCl_3_): δ_c_ 197.9, 197.7, 190.3, 190.2, 163.7, 163.6, 144.0, 143.6, 140.9, 140.1, 136.5, 135.7, 135.0, 134.3, 130.3, 130.3, 129.7, 129.6, 128.8, 128.7, 128.7, 128.5, 128.2, 124.4, 124.0, 113.8, 113.8, 64.0, 61.6, 55.5, 35.6, 35.3, 35.1, 33.9, 30.4, 29.4, 29.0, 28.9, 21.8, 21.6; **IR**: ῡ = 1652, 1595, 1571, 1294, 1134, 741 cm^−^^1^; HRMS (ESI-TOF) *m*/*z* [M + Na]^+^ calcd. for C_27_H_26_NaO_5_S^+^, 485.1393, found 485.1387.

**3-(2-Benzoyl-6-methyl-1,1-dioxidothiochroman-4-yl)-1-(p-tolyl)propan-1-one (5ah)**: Colorless oil (52.8 mg, 59% yield, dr = 1.5:1). **^1^H NMR** (500 MHz, CDCl_3_): δ_H_ 8.14 (d, *J* = 7.7 Hz, 1.2 H), 8.08 (d, *J* = 7.7 Hz, 0.8 H), 7.84–7.75 (m, 3.0 H), 7.64–7.59 (m, 1.0 H), 7.50 (t, *J* = 7.7 Hz, 1.0 H), 7.48 (t, *J* = 7.7 Hz, 1.0 H), 7.32 (s, 0.4 H), 7.24–7.20 (m, 3.6 H), 5.45 (dd, *J* = 9.7, 3.7 Hz, 0.6 H), 5.24 (dd, *J* = 11.5, 3.5 Hz, 0.4 H), 3.39–3.30 (m, 1.0 H), 3.18–2.95 (m, 2.6 H), 2.85–2.80 (m, 0.4 H), 2.56–2.43 (m, 1.4 H), 2.39 (s, 6.0 H), 2.30–2.19 (m, 1.6 H); **^13^C NMR** (126 MHz, CDCl_3_): δ_c_ 198.9, 198.7, 190.2, 190.0, 144.1, 144.0, 144.0, 143.5, 140.7, 139.9, 136.4, 136.3, 135.6, 134.9, 134.2, 134.1, 134.1, 129.6, 129.5, 129.3, 129.3, 128.7, 128.7, 128.6, 128.3, 128.1, 128.1, 128.0, 124.4, 124.0, 63.9, 61.5, 35.4, 35.4, 35.0, 34.0, 30.3, 29.2, 28.9, 28.8, 21.7, 21.6, 21.5; **IR**: ῡ = 1674, 1604, 1448, 1299, 1134, 728 cm^−^^1^; HRMS (ESI-TOF) *m*/*z* [M + Na]^+^ calcd. for C_27_H_26_NaO_4_S^+^, 469.1444, found 469.1420.

**3-(2-Benzoyl-6-methyl-1,1-dioxidothiochroman-4-yl)-1-(3-methoxyphenyl)propan-1-one (5ai)**: Colorless oil (53.8 mg, 58% yield, dr = 1.5:1). **^1^H NMR** (500 MHz, CDCl_3_): δ_H_ 8.14 (d, *J* = 7.7 Hz, 1.2 H), 8.08 (d, *J* = 7.7 Hz, 0.8 H), 7.81 (d, *J* = 7.7 Hz, 0.4 H), 7.76 (d, *J* = 7.7 Hz, 0.6 H), 7.64–7.59 (m, 1.0 H), 7.52–7.47 (m, 3.0 H), 7.42 (s, 0.8 H), 7.36–7.32 (m, 1.6 H), 7.24–7.20 (m, 1.6 H), 7.10 (d, *J* = 7.7 Hz, 1.0 H), 5.44 (dd, *J* = 10.0, 4.0 Hz, 0.6 H), 5.24 (dd, *J* = 11.5, 4.0 Hz, 0.4 H), 3.84 (s, 1.2 H), 3.81 (s, 1.8 H), 3.39–3.30 (m, 1.0 H), 3.20–2.97 (m, 2.6 H), 2.85–2.80 (m, 0.4 H), 2.56–2.43 (m, 1.4 H), 2.41 (s, 1.2 H), 2.39 (s, 1.8 H), 2.31–2.21 (m, 1.6 H); **^13^C NMR** (126 MHz, CDCl_3_): δ_c_ 199.1, 198.9, 190.2, 190.0, 159.8, 144.0, 143.6, 140.7, 139.9, 137.9, 137.9, 136.4, 136.3, 135.6, 134.9, 134.2, 129.6, 129.6, 129.5, 129.5, 128.7, 128.7, 128.6, 128.3, 128.2, 124.5, 124.1, 120.6, 120.5, 119.7, 119.6, 112.3, 112.2, 63.9, 61.6, 55.4, 55.4, 35.7, 35.4, 35.0, 34.4, 30.3, 29.3, 28.9, 28.8, 21.7, 21.5; **IR**: ῡ = 1679, 1596, 1448, 1260, 1135, 731 cm^−^^1^; HRMS (ESI-TOF) *m*/*z* [M + Na]^+^ calcd. for C_27_H_26_NaO_5_S^+^, 485.1393, found 485.1388.

**3-(2-Benzoyl-6-methyl-1,1-dioxidothiochroman-4-yl)-1-(3-chlorophenyl)propan-1-one (5aj)**: Colorless oil (61.8 mg, 66% yield, dr = 1.5:1). **^1^H NMR** (500 MHz, CDCl_3_): δ_H_ 8.13 (d, *J* = 8.0 Hz, 1.2 H), 8.08 (d, *J* = 8.0 Hz, 0.8 H), 7.91 (t, *J* = 1.7 Hz, 0.4 H), 7.87 (t, *J* = 1.7 Hz, 0.6 H), 7.83–7.76 (m, 2.0 H), 7.65–7.60 (m, 1.0 H), 7.53–7.47 (m, 3.0 H), 7.40–7.37 (m, 1.0 H), 7.30 (s, 0.4 H), 7.25–7.20 (m, 1.6 H), 5.43 (dd, *J* = 9.5, 4.0 Hz, 0.6 H), 5.24 (dd, *J* = 11.5, 4.0 Hz, 0.4 H), 3.40–3.32 (m, 1.0 H), 3.18–2.95 (m, 2.6 H), 2.88–2.80 (m, 0.4 H), 2.56–2.43 (m, 1.4 H), 2.42 (s, 1.2 H), 2.40 (s, 1.8 H), 2.34–2.21(m, 1.6 H); **^13^C NMR** (126 MHz, CDCl_3_): δ_c_ 198.0, 197.8, 190.2, 189.9, 144.1, 143.7, 140.6, 139.8, 138.1, 138.1, 136.4, 136.3, 135.6, 135.0, 134.8, 134.3, 133.2, 133.1, 130.0, 130.0, 129.6, 129.5, 129.4, 128.7, 128.7, 128.7, 128.2, 128.2, 128.0, 128.0, 126.1, 126.0, 124.5, 124.2, 63.9, 61.7, 35.6, 35.2, 34.9, 34.3, 30.3, 29.1, 29.0, 28.6, 21.8, 21.6; **IR**: ῡ = 1682, 1596, 1448, 1298, 1134, 729 cm^−^^1^; HRMS (ESI-TOF) *m*/*z* [M + Na]^+^ calcd. for C_26_H_23_ClNaO_4_S^+^, 489.0898, found 489.0885.

**3-(2-Benzoyl-6-methyl-1,1-dioxidothiochroman-4-yl)-1-(2-methoxyphenyl)propan-1-one (5ak)**: Colorless oil (35.3 mg, 38% yield, dr = 1.5:1). **^1^H NMR** (500 MHz, CDCl_3_): δ_H_ 8.17 (d, *J* = 8.0 Hz, 1.2 H), 8.09 (d, *J* = 8.0 Hz, 0.8 H), 7.81 (d, *J* = 8.0 Hz, 0.4 H), 7.76 (d, *J* = 8.0 Hz, 0.6 H), 7.70 (dd, *J* = 8.0, 2.0 Hz, 0.4 H), 7.66–7.60 (m, 1.6 H), 7.54–7.44 (m, 3.0 H), 7.34 (s, 0.4 H), 7.24–7.20 (m, 1.6 H), 7.01–6.93 (m, 2.0 H), 5.45 (dd, *J* = 10.3, 3.7 Hz, 0.6 H), 5.22 (dd, *J* = 11.5, 4.0 Hz, 0.4 H), 3.90 (s, 1.2 H), 3.83 (s, 1.8 H), 3.36–3.25 (m, 1.0 H), 3.24–3.14 (m, 1.0 H), 3.10–2.99 (m, 1.6 H), 2.87–2.80 (m, 0.4 H), 2.57–2.43 (m, 1.4 H), 2.42 (s, 1.2 H), 2.40 (s, 1.8 H), 2.26–2.14 (m, 1.6 H); **^13^C NMR** (126 MHz, CDCl_3_): δ_c_ 201.7, 201.4, 190.1, 190.1, 158.6, 158.5, 143.4, 140.8, 140.4, 136.4, 135.5, 135.0, 134.3, 134.2, 133.7, 133.7, 130.2, 129.7, 129.7, 129.5, 129.5, 128.7, 128.7, 128.6, 128.5, 128.4, 128.0, 127.8, 127.8, 124.3, 124.0, 120.7, 120.6, 111.6, 63.9, 61.5, 55.5, 55.5, 41.2, 39.7, 35.8, 30.3, 29.7, 29.5, 28.7, 21.8, 21.6; **IR**: ῡ = 1678, 1595, 1484, 1298, 1134, 729 cm^−^^1^; HRMS (ESI-TOF) *m*/*z* [M + Na]^+^ calcd. for C_27_H_26_NaO_5_S^+^, 485.1393, found 485.1370.

**3-(2-Benzoyl-6-methyl-1,1-dioxidothiochroman-4-yl)-1-(3,4-dichlorophenyl)propan-1-one (5al)**: Colorless oil (55.3 mg, 55% yield, dr = 1.5:1). **^1^H NMR** (500 MHz, CDCl_3_): δ_H_ 8.13 (d, *J* = 7.3 Hz, 1.2 H), 8.08 (d, *J* = 7.3 Hz, 0.8 H), 8.00 (d *J* = 2.0 Hz, 0.4 H), 7.97 (d, *J* = 2.0 Hz, 0.6 H), 7.82 (d, *J* = 8.0 Hz, 0.4 H), 7.76 (d, *J* = 8.0 Hz, 0.6 H), 7.74–7.71 (m, 1.0 H), 7.65–7.59 (m, 1.0 H), 7.53–7.47 (m, 3.0 H), 7.29 (s, 0.4 H), 7.23 (t, *J* = 8.0 Hz, 1.0 H), 7.19 (s, 0.6 H), 5.41 (dd, *J* = 9.0, 4.0 Hz, 0.6 H), 5.23 (dd, *J* = 11.5, 4.0 Hz, 0.4 H), 3.40–3.33 (m, 1.0 H), 3.14–2.94 (m, 2.6 H), 2.88–2.81 (m, 0.4 H), 2.55–2.44 (m, 1.4 H), 2.42 (s, 1.2 H), 2.40 (s, 1.8 H), 2.33–2.21(m, 1.6 H); **^13^C NMR** (126 MHz, CDCl_3_): δ_c_ 197.0, 196.9, 190.2, 189.9, 144.1, 143.7, 140.5, 139.7, 137.9, 137.8, 136.4, 136.3, 136.1, 136.1, 135.7, 134.7, 134.3, 133.4, 133.4, 130.8, 130.7, 129.9, 129.6, 129.6, 129.4, 128.7, 128.7, 128.7, 128.3, 128.2, 127.1, 127.0, 124.6, 124.2, 63.8, 61.7, 35.5, 35.1, 34.9, 34.2, 30.2, 29.1, 28.9, 28.5, 21.8, 21.6; **IR**: ῡ = 1682, 1596, 1448, 1298, 1133, 726 cm^−^^1^; HRMS (ESI-TOF) *m*/*z* [M + Na]^+^ calcd. for C_26_H_22_Cl_2_NaO_4_S^+^, 523.0508, found 523.0516.

**3-(2-Benzoyl-6-methyl-1,1-dioxidothiochroman-4-yl)-1-(thiophen-2-yl)propan-1-one (5am)**: Colorless oil (36.9 mg, 42% yield, dr = 1.5:1). **^1^H NMR** (500 MHz, CDCl_3_): δ_H_ 8.15 (d, *J* = 8.0 Hz, 1.2 H), 8.10 (d, *J* = 8.0 Hz, 0.8 H), 7.83 (d, *J* = 8.0 Hz, 0.4 H), 7.78 (d, *J* = 8.0 Hz, 0.6 H), 7.70 (dd, *J* = 10.3, 3.7 Hz, 1.0 H), 7.66–7.60 (m, 2.0 H), 7.54–7.49 (m, 2.0 H), 7.33 (s, 0.4 H), 7.25–7.22 (m, 1.0 H), 7.21 (s, 0.6 H), 7.12 (t, *J* = 4.3 Hz, 1.0 H), 5.42 (dd, *J* = 10.5, 4.0 Hz, 0.6 H), 5.23 (dd, *J* = 10.5, 4.0 Hz, 0.4 H), 3.41–3.34 (m, 1.0 H), 3.15–2.95 (m, 2.6 H), 2.90–2.83 (m, 0.4 H), 2.58–2.45 (m, 1.4 H), 2.43 (s, 1.2 H), 2.40 (s, 1.8 H), 2.35–2.23 (m, 1.6 H); **^13^C NMR** (126 MHz, CDCl_3_): δ_c_ 192.2, 191.9, 190.2, 190.0, 144.1, 143.9, 143.8, 143.7, 140.6, 139.8, 136.4, 136.4, 135.6, 134.8, 134.3, 133.9, 133.8, 132.1, 132.0, 129.6, 129.6, 129.5, 128.8, 128.8, 128.7, 128.4, 128.3, 128.2, 124.6, 124.2, 63.9, 61.7, 36.2, 35.3, 35.0, 34.9, 30.3, 29.7, 29.5, 29.1, 21.8, 21.6; **IR**: ῡ = 1658, 1595, 1415, 1297, 1129, 725 cm^−^^1^; HRMS (ESI-TOF) *m*/*z* [M + Na]^+^ calcd. for C_24_H_22_NaO_4_S_2_^+^, 461.0852, found 461.0874.

**3-(2-Benzoyl-6-methyl-1,1-dioxidothiochroman-4-yl)-1-cyclopropylpropan-1-one (5an)**: Colorless oil (24.7 mg, 31% yield, dr = 1.5:1). **^1^H NMR** (500 MHz, CDCl_3_): δ_H_ 8.15 (d, *J* = 7.3 Hz, 1.2 H), 8.09 (d, *J* = 7.3 Hz, 0.8 H), 7.82 (d, *J* = 8.0 Hz, 0.4 H), 7.76 (d, *J* = 8.0 Hz, 0.6 H), 7.66–7.61 (m, 1.0 H), 7.54–7.49 (m, 2.0 H), 7.28 (s, 0.4 H), 7.25–7.21 (m, 1.0 H), 7.17 (s, 0.6 H), 5.40 (dd, *J* = 10.0, 4.0 Hz, 0.6 H), 5.21 (dd, *J* = 11.5, 4.0 Hz, 0.4 H), 3.30–3.21 (m, 1.0 H), 3.00–2.95 (m, 0.4 H), 2.81–2.62 (m, 2.6 H), 2.51–2.45 (m, 0.4 H), 2.43 (s, 1.2 H), 2.41 (s, 1.8 H), 2.39–2.32 (m, 1.6 H), 2.17–2.07 (m, 1.0 H), 1.94–1.89 (m, 1.0 H), 1.03–0.95 (m, 2.0 H), 0.90–0.85 (m, 2.0 H); **^13^C NMR** (126 MHz, CDCl_3_): δ_c_ 210.0, 209.8, 190.2, 190.0, 144.0, 143.5, 140.6, 139.9, 136.4, 136.4, 135.6, 134.9, 134.3, 129.6, 129.6, 129.5, 128.7, 128.7, 128.7, 128.3, 128.2, 124.5, 124.1, 63.9, 61.6, 40.6, 39.2, 35.4, 35.0, 30.3, 29.7, 28.9, 28.5, 21.8, 21.6, 20.7, 20.7, 11.1, 11.0, 11.0, 10.9; **IR**: ῡ = 1682, 1596, 1448, 1300, 1134, 728 cm^−^^1^; HRMS (ESI-TOF) *m*/*z* [M + Na]^+^ calcd. for C_23_H_24_NaO_4_S^+^, 419.1288, found 419.1268.

## 4. Conclusions

In summary, we have developed a photoredox-assisted intermolecular radical cascade cyclization reaction with *α*-carbonyl alkyl bromide **2** for the facile synthesis of diverse organosulfones. This innovative photocatalytic approach has garnered significant interest owing to its straightforward operation, remarkable compatibility with various functional groups, and impressive yields under mild reaction conditions. We are confident that this strategy will discover extensive utility in the realm of organic synthesis.

## Figures and Tables

**Table 1:** Optimization of the reaction conditions *^a^*.

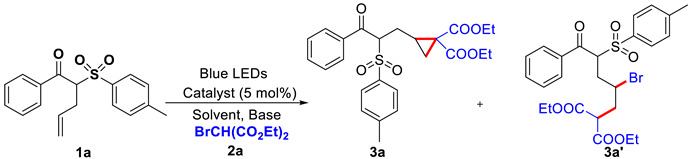						
Entry	Photocatalyst	Base	Additive	Solvent	Yield of (%) *^b^*	
					3a	3a′
1	*fac*-Ir(ppy)_3_	2,6-lutidine	-	DMF	16	32
2	*fac*-Ir(ppy)_3_	2,6-lutidine	-	1,4-Dioxane	23	45
3	*fac*-Ir(ppy)_3_	2,6-lutidine	-	CHCl_3_	25	50
4	*fac*-Ir(ppy)_3_	2,6-lutidine	-	DMSO	21	42
5	*fac*-Ir(ppy)_3_	2,6-lutidine	-	Methylbenzene	26	51
6	*fac*-Ir(ppy)_3_	2,6-lutidine	-	Chlorobenzene	27	54
7	*fac*-Ir(ppy)_3_	2,6-lutidine	-	Acetone	22	44
8	*fac*-Ir(ppy)_3_	2,6-lutidine	-	*o*-DCB	11	22
9	*fac*-Ir(ppy)_3_	K_2_HPO_4_	-	Chlorobenzene	20	40
10 *^c^*	*fac*-Ir(ppy)_3_	-	-	Chlorobenzene	Trace	0
11	*fac*-Ir(ppy)_3_	K_2_CO_3_	-	Chlorobenzene	Trace	0
13	*fac*-Ir(ppy)_3_	KHCO_3_	-	Chlorobenzene	18	36
14 *^d^*	*fac*-Ir(ppy)_3_	2,6-lutidine	-	Chlorobenzene	32	63
15 *^e^*	*fac*-Ir(ppy)_3_	2,6-lutidine	LiBF_4_ (2.0 equiv.)	Chlorobenzene	36	40
16 *^f^*	*fac*-Ir(ppy)_3_	2,6-lutidine	LiBF_4_ (2.0 equiv.)	Chlorobenzene	41	27
17 *^g^*	*fac*-Ir(ppy)_3_	2,6-lutidine	LiBF_4_ (2.0 equiv.)	Chlorobenzene	48	28
18 *^h^*	*fac*-Ir(ppy)_3_	2,6-lutidine	LiBF_4_ (2.0 equiv.)	Chlorobenzene	37	26
19 *^i^*	*fac*-Ir(ppy)_3_	2,6-lutidine	LiBF_4_ (2.0 equiv.)	Chlorobenzene	Trace	0
20 *^j^*	*fac*-Ir(ppy)_3_	2,6-lutidine	LiBF_4_ (2.0 equiv.)	Chlorobenzene	N.R.	0
21 *^k^*	—	2,6-lutidine	LiBF_4_ (2.0 equiv.)	Chlorobenzene	0	0

*^a^* Reaction conditions: **1a** (0.2 mmol, 0.4 M in solvent), **2a** (2.0 equiv.), LiBF_4_ (2.0 equiv.), base (5.0 equiv.), *fac*-Ir(ppy)*_3_* (0.005 mmol), under N_2_ atmosphere irradiated using blue LEDs (5W) at room temperature for 53 h. *^b^* Isolated yield of **3a**. *^c^* Without base. *^d^* chlorobenzene (2.0 mL), 2,6-lutidine (3.0 eq). *^e^* chlorobenzene (2.0 mL), 2,6-lutidine (3.0 eq). *^f^* chlorobenzene (0.5 mL), 2,6-lutidine (3.0 eq). *^g^* chlorobenzene (0.5 mL), 2,6-lutidine (5.0 eq). *^h^* chlorobenzene (0.5 mL), 2,6-lutidine (7.0 eq). *^i^* In the air. *^j^* In the dark. *^k^* Without catalyst.

**Table 2:** Scope of substrate **1** for six-membered ring products **4** *^a,b^*.

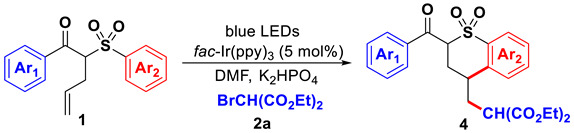		
Entry	Compound 1, Ar_1_ =, Ar_2_ =	Yield (%) *^b^*
1	Ar_1_ = Ph, Ar_2_ = 4-MeC_6_H_4_	**4a**, 67%
2	Ar_1_ = Ph, Ar_2_ = 4-*^t^*BuC_6_H_4_	**4b**, 48%
3	Ar_1_ = Ph, Ar_2_ = 4-MeOC_6_H_4_	**4c**, 61%
4	Ar_1_ = Ph, Ar_2_ = 4-BrC_6_H_4_	**4d**, 35%
5	Ar_1_ = Ph, Ar_2_ = Ph	**4e**, 40%
6	Ar_1_ = Ph, Ar_2_ = 2-MeC_6_H_4_	**4f**, 34%
7	Ar_1_ = Ph, Ar_2_ = 3-MeC_6_H_4_	**4g**, 43%
8	Ar_1_ = 4-MeC_6_H_4_, Ar_2_ = 4-MeC_6_H_4_	**4h**, 68%
9	Ar_1_ = 4-MeOC_6_H_4_, Ar_2_ = 4-MeC_6_H_4_	**4i**, 62%
10	Ar_1_ = 4-BrC_6_H_4_, Ar_2_ = 4-MeC_6_H_4_	**4j**, 33%
11	Ar_1_ = 4-MeC_6_H_4_, Ar_2_ = 4-FC_6_H_4_	**4k**, 58%
12	Ar_1_ = 4-MeOC_6_H_4_, Ar_2_ = 4-FC_6_H_4_	**4l**, 56%
13	Ar_1_ = 4-MeOC_6_H_4_, Ar_2_ = 4-BrC_6_H_4_	**4m**, 55%
14	Ar_1_ = 4-FC_6_H_4_, Ar_2_ = 4-MeOC_6_H_4_	**4n**, 58%
15	Ar_1_ = 4-BrC_6_H_4_, Ar_2_ = 4-MeOC_6_H_4_	**4o**, 29%
16	Ar_1_ = 3-MeOC_6_H_4_, Ar_2_ = 4-FC_6_H_4_	**4p**, 29%
17	Ar_1_ = 2-MeOC_6_H_4_, Ar_2_ = 4-FC_6_H_4_	**4q**, 54%

*^a^* Reaction conditions: **1a** (0.2 mmol, 0.1 M in dry DMF), **2a** (2.0 equiv.), base (2.0 equiv.), fac-Ir(ppy)_3_ (0.005 mmol), under N_2_ atmosphere irradiated using blue LEDs (5W) at room temperature for 24 h. *^b^* Isolated yield, dr = 2.0:1.

## Data Availability

All data generated or analyzed during this study are included in this published article and its Supplementary Information Files.

## Supplementary Materials

The following supporting information can be downloaded at: https://www.mdpi.com/article/10.3390/molecules29091971/s1, Table S1. Optimization of the reaction conditions. References [17,18,28,29,30,31,32,33,34,35] are cited in the Supplementary Materials.
