# Supplementary material for: Efficient Functionalization of Organosulfones via Photoredox Catalysis: Direct Incorporation of α-Carbonyl Alkyl Side Chains into α-Allyl-β-Ketosulfones
Source: Molecules. 2024 Apr 25;29(9):1971. doi: 10.3390/molecules29091971 (PMC11085174; doi:10.3390/molecules29091971)

Supplementary Information
For

# Efficient Functionalization of Organosulfones via Photoredox Catalysis: Direct Incorporation of *α*-Carbonyl Alkyl Side Chains into *α*-Allyl-*β*-Ketosulfones

**Hong-Li Huang [1,*], Shan Li [1], Yong-Zheng Lv [1], Ya-Qian Shi [1], Tian-Tian Pang [1], Ru-Fen Zhang [1], Wenjing Huang [2], Jianhui Yin [2] and Fei Gao [2,*]**

[1] College of Chemistry and Chemical Engineering, Liaocheng University, Liaocheng 252059, China
[2] Institude of Translation Medicine, Shanghai University, Shanghai 200444, China

## Table of Contents

1. General information……………………………………………………………S2

2. Experimental procedures…………………………………………………………..S2

2.1 Synthesis of starting materials…………………………………………………S2

2.2 Optimization data for compound **4a**………………………………………..S3

2.3 Light Source in Detail and Experimental Set-up Photograph........................S5

2.4 TEMPO control experiment for compound **3a**.............................................S5

2.5 TEMPO control experiment for compound **4a**............................................S6

2.6 NBS as bromination reagent for compound **4a**……………………………..S7

3. Copies of NMR Spectra ………………………………………………………......S8

## 1. General information.

Unless otherwise noted, all reactions were performed under a nitrogen atmosphere and were monitored using TLC and visualized using a UV lamp (254 nm) /or via treatment with a solution of 10 g phosphomolybdic acid and 100 mL EtOH followed by heating. All reagents were used as received from commercial sources without further purification. Silica gel (200-300 mesh) and silica gel $GF_{254}$ (10-40 $\mu$m) were used for column chromatography (CC) and prepare thin layer chromatography (PTLC), respectively. Solvents were dried and purified according to the procedure from "Purification of Laboratory Chemicals book". $^1$H NMR and $^{13}$C NMR spectra were recorded in $CDCl_3$ on a Varian 500 MHz instrument. Chemical shifts were denoted in ppm ($\delta$) and calibrated using residual undeuterated solvent ($CDCl_3$ (7.26 ppm), or tetramethylsilane (0.00 ppm)) as internal reference for $^1$H NMR and the deuterated solvent ($CDCl_3$ (77.00 ppm), or tetramethylsilane (0.00 ppm)) as internal standard for $^{13}$C NMR. The following abbreviations were used to denote the multiplicities: s = singlet, d = doublet, t = triplet, q = quartet, br = broad, td = triple doublet, dt = double triplet, m = multiplet. The MS data were obtained with ESI technique, and the relative intensity (%) is given in brackets. High-resolution mass spectral analysis (HRMS) data were measured using a Bruker ApexII mass spectrometer by means of the ESI technique.

## 2. Experimental procedures

### 2.1 Synthesis of starting materials

General Procedure for the Preparation of Sodium Sulfinates[1]:

Benzenesulfinic acid sodium salt (1.0 eq.) was prepared by heating sodium sulfite (1.26 g, 10 mmol, 2.0 eq.), benzenesulphonyl chloride (5 mmol, 1.0 eq.), and sodium bicarbonate (0.84 g, 10 mmol, 2.0 eq.) in 10 mL of water at 70-80 $^{o}$C for 5 h. After cooling to room temperature, water was removed under vacuum and the residue was extracted by ethanol, recrystallization as a white solid, the yield was 60-70%.

Similarly, other sodium arenesulfinates were prepared from their corresponding sulfonyl chlorides.

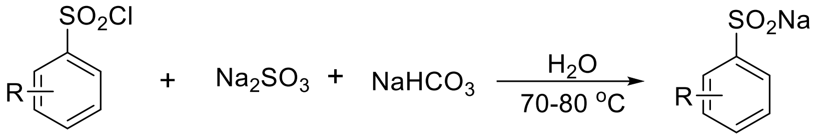


General Procedure for the Preparation of substrates **1a-1x**[2,3]

Sodium sulfinates ($RSO_2Na$, 3.6 mmol, 1.2 eq.) was added to a solution of substituted 2-bromoacetophenones (3.0 mmol, 1.0 eq.) in a cosolvent of 1,4-dioxane and water (20 mL, v/v = 1:1) at rt. The reaction mixture was stirred at reflux for 6 h. After the reaction was completed, the reaction mixture was extracted with $CH_2Cl_2$ (3 × 20 mL). The organic layer was combined, dried (MgSO4), filtered, and evaporated to obtain crude *β*-ketosulfones[2] under reduced pressure. Crude *β*-ketosulfones were recrystallized from petroleum ether/EtOAc in nearly quantitative yields.

$K_2CO_3$ (0.621 g, 4.5 mmol, 1.5 eq.) was added to a solution of *β*-ketosulfones (3.0 mmol, 1.0 eq.) in acetone (30 mL) at rt. The reaction mixture was stirred at rt for 10 min. Allyl halides (3.3 mmol, 1.1 eq.) were added to the reaction mixture at rt. The reaction mixture was stirred at reflux for 14 h. After the reaction was completed, the reaction mixture was extracted with EtOAc (30 mL × 3). The organic layer was combined, dried ($MgSO_4$), filtered, and concentrated in vacuo. The resulting residue was purified by silica gel (petroleum ether/EtOAc = 15:1) obtained **1a-1x.**

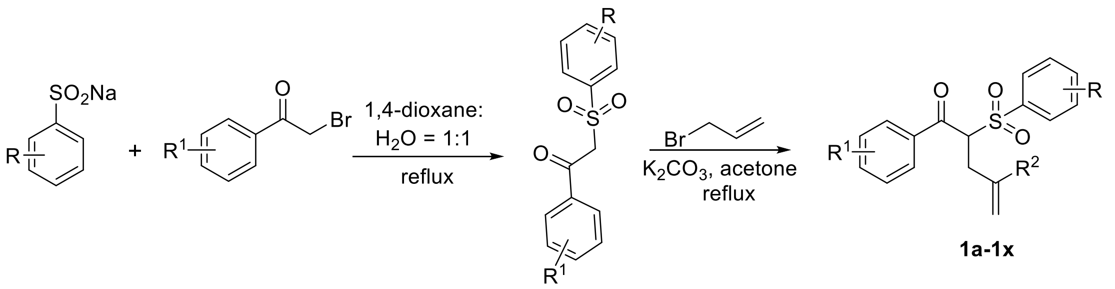


Substrates **1a**,[3a,c] **1b**,[3b] **1c-d**,[3a] **1g**,[3b] **1o-p**[3a] are known compounds and the analytical data are consistent with the previous literature.

**2.2 Optimization data for compound 4a**

**Table S1.** Optimization of the reaction conditions[a,b]

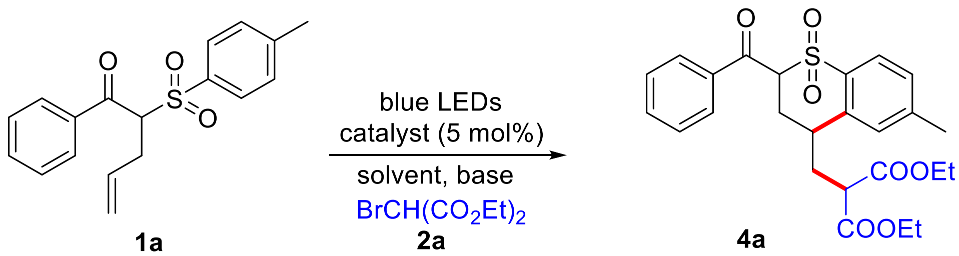


| Entry | Photocatalyst | Base | Solvent | Yield (%) |
|---|---|---|---|---|
| **1** | ***fac*-Ir(ppy)$_3$** | **$K_2HPO_4$** | **DMF** | **67** |
| 2 | (Ir[dF(CF$_3$)ppy]$_2$(dtbpy))PF$_6$ | $K_2HPO_4$ | DMF | 33 |
| 3 | Eosin Y | $K_2HPO_4$ | DMF | N. R. |
| 4 | Ru(bpy)$_3$Cl$_2$ | $K_2HPO_4$ | DMF | 12 |
| 5 | *fac*-Ir(ppy)$_3$ | $K_2HPO_4$ | $CH_3CN$ | 29 |
| 6 | *fac*-Ir(ppy)$_3$ | $K_2HPO_4$ | $CHCl_3$ | 37 |
| 7 | *fac*-Ir(ppy)$_3$ | $K_2HPO_4$ | DMSO | 49 |
| 8 | *fac*-Ir(ppy)$_3$ | $K_2HPO_4$ | 1,4-Dioxane | 46 |
| 9 | *fac*-Ir(ppy)$_3$ | $K_2HPO_4$ | MeOH | 31 |
| 10[c] | *fac*-Ir(ppy)$_3$ | — | DMF | 16 |
| 11 | *fac*-Ir(ppy)$_3$ | $K_2CO_3$ | DMF | 41 |
| 12 | *fac*-Ir(ppy)$_3$ | KOH | DMF | 27 |
| 13 | *fac*-Ir(ppy)$_3$ | 2,6-lutidine | DMF | 35 |
| 14 | *fac*-Ir(ppy)$_3$ | $KHCO_3$ | DMF | 39 |
| 15 | *fac*-Ir(ppy)$_3$ | CsF | DMF | 26 |
| 16[d] | *fac*-Ir(ppy)$_3$ | $K_2HPO_4$ | DMF | N.R. |
| 17[e] | *fac*-Ir(ppy)$_3$ | $K_2HPO_4$ | DMF | trace |
| 18[f] | — | $K_2HPO_4$ | DMF | 0 |

[a]Reaction conditions: **1a** (0.2 mmol, 0.1 M in solvent), **2a** (2.0 equiv.), base (2.0 equiv.), *fac*-Ir(ppy)$_3$ (0.005 mmol), under $N_2$ atmosphere irradiated using blue LEDs (5W) at room temperature for **24** h. [b]Isolated yield of **4a**. [c]Without base. [d]In the dark. [e]In the air. [f]Without catalyst.

## 2.3 Light Source in Detail and Experimental Set-up Photograph

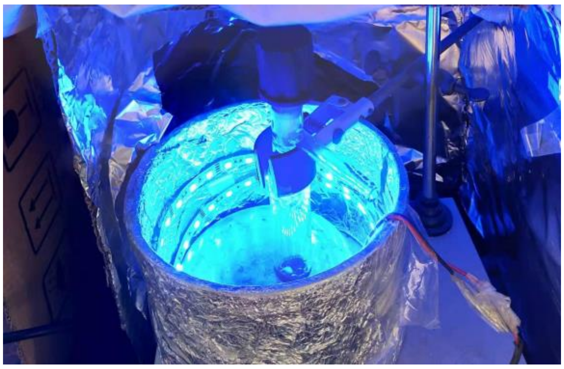

Small scale reaction

The light source used for photochemical experiments was a 5 W blue Leds belt, purchased from JD.COM

Manufacturer: Gree Think. China

Model: 12v3528

Broadband source: λ = 460-470 nm

Material of the irradiation vessel: Schlenk tube

Distance from the light source to the irradiation vessel: 6.0 cm (Not use any filters)

### 2.4 TEMPO control experiment for compound 3a

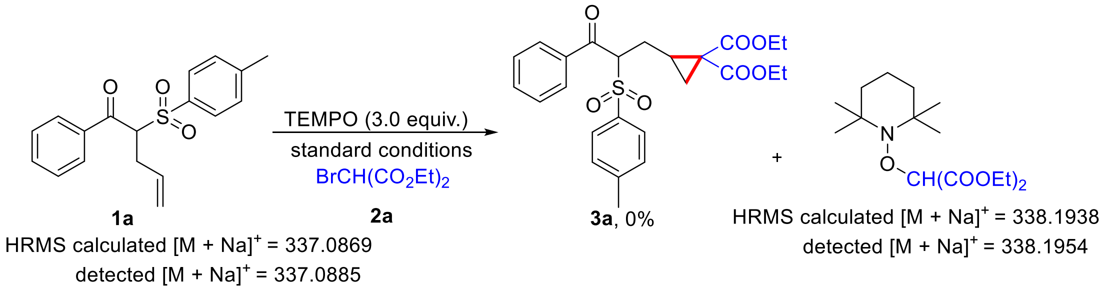


**HRMS data**:

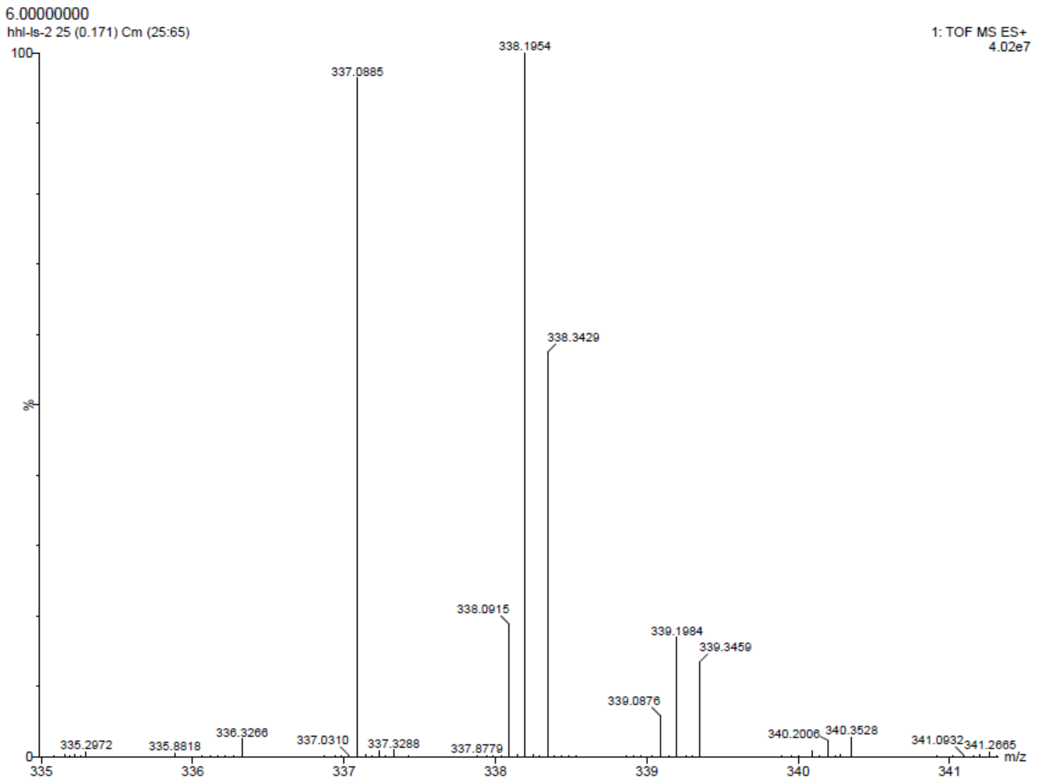


## 2.5 TEMPO control experiment for compound 4a

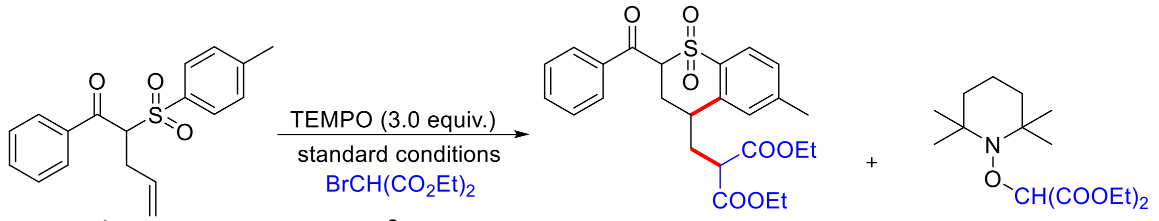


**1a** HRMS calculated $[M + Na]^+$ = 337.0869 detected $[M + Na]^+$ = 337.0889

**2a**

**4a**, 0%

HRMS calculated $[M + Na]^+$ = 338.1938 detected $[M + Na]^+$ = 338.1956

**HRMS data**:

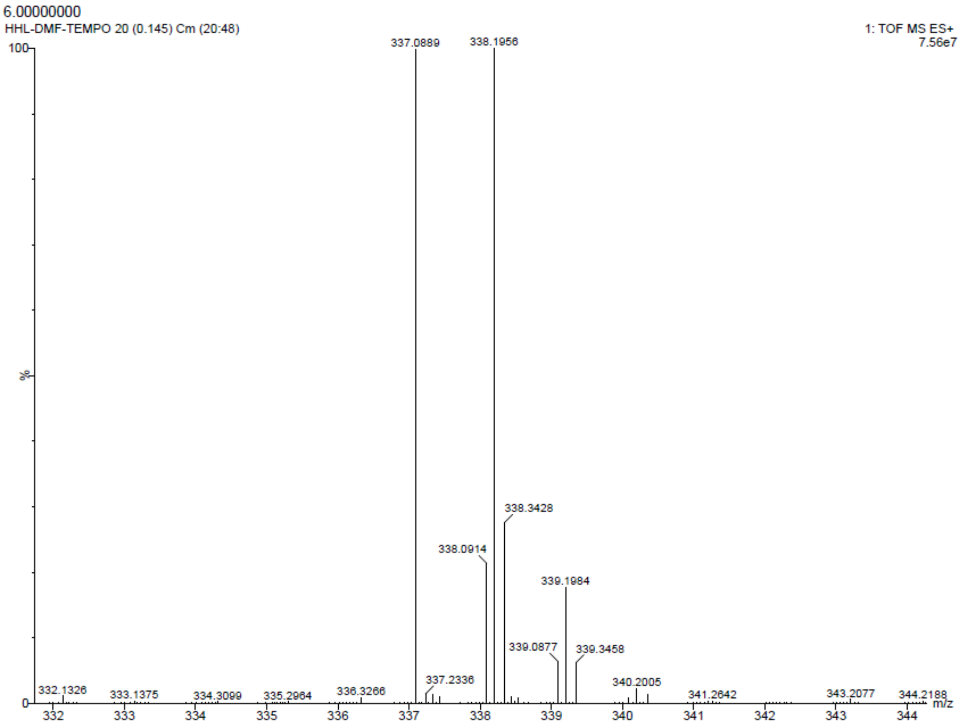


## 2.6 NBS as bromination reagent for compound 4a.

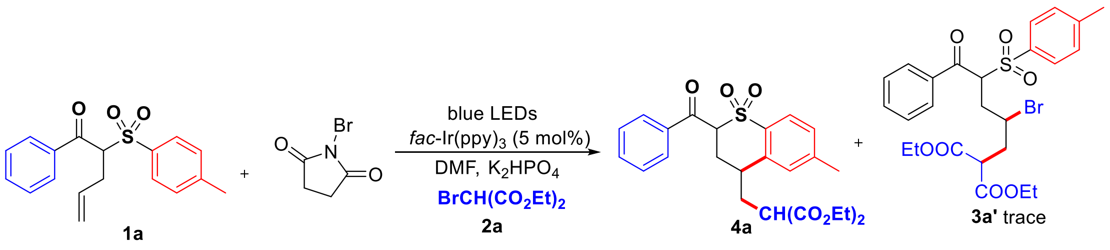


## References

[1]. (a) Fang, Y.; Xu, D.; Yu, Y.; Tang, R.; Dai, S.; Wang, Z.; Zhang, W. Controlled Synthesis of $\beta$-Keto Sulfones and Vinyl Sulfones under Electrochemical Oxidation. Eur. *J. Org. Chem.*, **2022**, *2022*, e202200091; (b) S. Liu, R. Chen, J. Zhang, Copper-Catalyzed Redox Coupling of Nitroarenes with Sodium Sulfinates. *Molecules* **2019**, *24*, 1407-1417.

[2]. (a) Reddy, R. J.; Kumari, A. H.; Kumar, J. J. Recent Advances in the Synthesis and Applications of $\beta$-Keto Sulfones: New Prospects for the Synthesis of $\beta$-Keto Thiosulfones. *Org. Biomol. Chem.*, **2021**, *19*, 3087-3118; (b) Chang, M.-Y.; Chen, H.-Y.; Chen, Y.-H. Synthesis of 2‑Aryl-3-sulfonylchromans via Knoevenagel Condensation and Reduction Protocol. *J. Org. Chem.*, **2017**, *82*, 12631-12639. (c) Chang, M.-Y.; Lai, K.-X.; Chang, Y.-L. In(OTf)$_3$-Catalyzed Intramolecular Hydroarylation of $\alpha$-Phenylallyl

$\beta$-Ketosulfones-Synthesis of Sulfonyl 1-Benzosuberones and 1-Tetralones. RSC Adv., **2020**, *10*, 18231-18244. (d) Hsueh, N.-C.; Hsiao, Y.-T.; Chang, M.-Y. CuI Mediated Synthesis of Sulfonyl Dihydrofurans. *Tetrahedron* **2017**, *73*, 4398-4406. (e) Chang, M.-Y.; Cheng, Y.-C. $Bi(OTf)_3$ Mediated *exo*-Olefin Isomerization of $\alpha$–Benzoyl $\beta$–Styrylsulfones. *Org. Lett.*, **2015**, *17*, 5702-5705.

[3]. (a) Chang, M.-Y.; Cheng, Y.-C.; Lu, Y.-J. One-Pot Access to Sulfonylmethyl Arylpyrroles via the Domino Aerobic Wacker-Type Aminocyclization/1,4-Sulfonyl Migration. *Org. Lett.*, **2014**, *16*, 6252-6255. (b) Chang, M.-Y.; Hsiao, Y.-T. $H_2SO_4$-Mediated Stereocontrolled Annulation of Oxygenated Naphthalenes and 4-Alkenols: One-Pot Synthesis of Tetanthrenes. *J. Org. Chem.* **2017**, *82*, 11594-11602. (c) Chang, M.-Y.; Cheng, Y.-C.; Chan, C.-K. Synthesis of Vinylcyclopropanes by Allylation/ring-closing Metathesis/Claisen Rearrangement. *Tetrahedron* **2014**, *70*, 8908-8913.

## 3. Copies of NMR Spectra

$^1$H NMR (500 MHz, $CDCl_3$) spectrum of compound **1e**

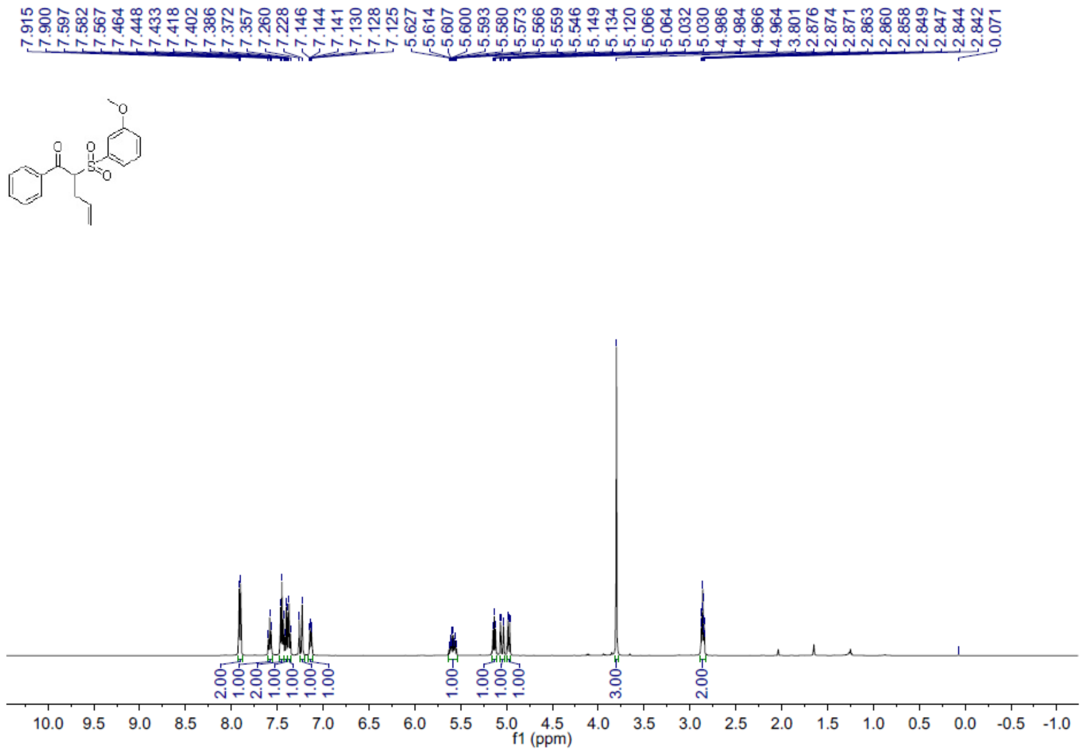


$^{13}C\ \{^{1}H\}$ NMR (126 MHz, $CDCl_3$) spectrum of compound **1e**

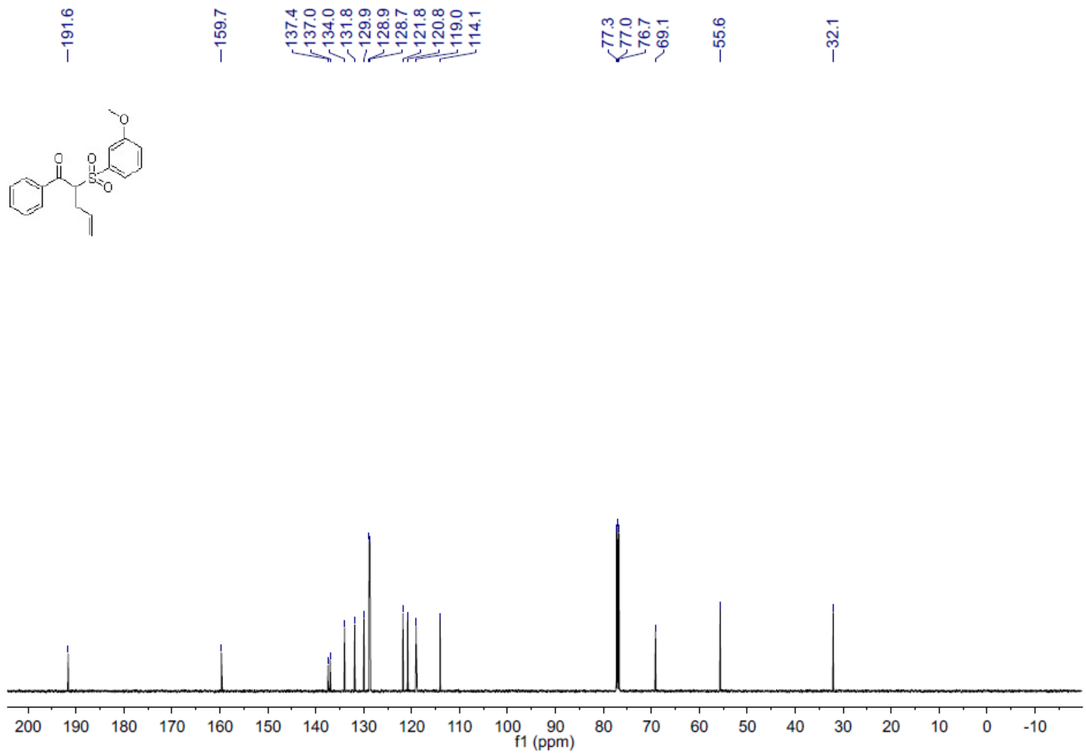

$^{1}H$ NMR (500 MHz, $CDCl_3$) spectrum of compound **1f**

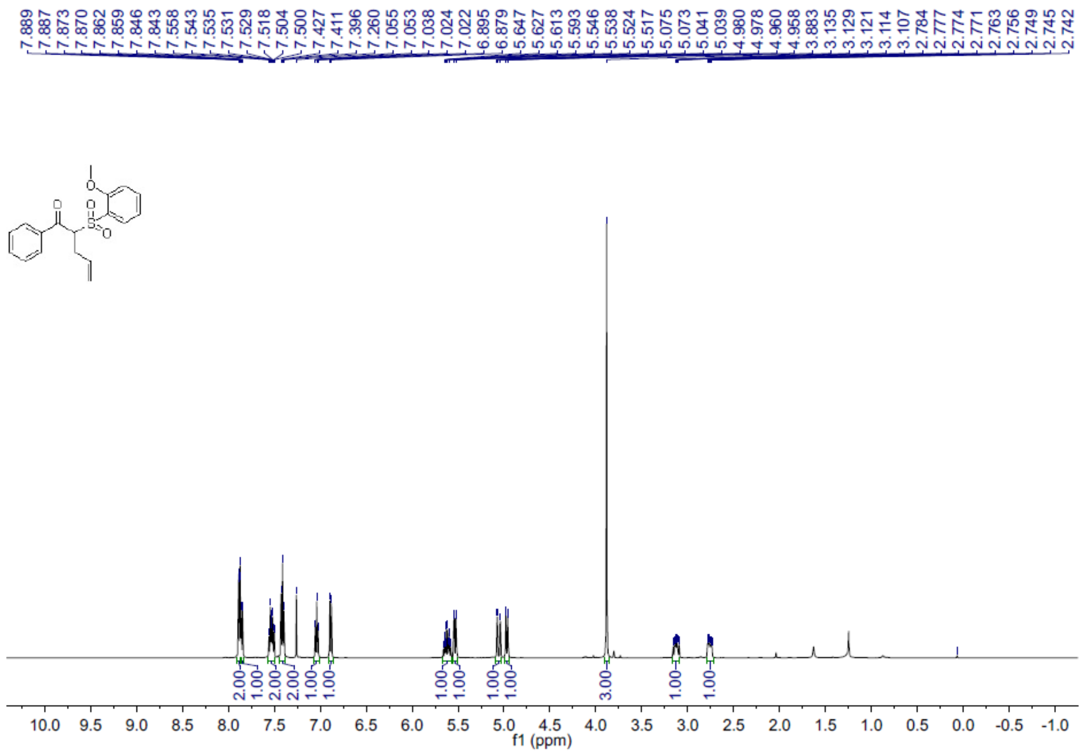


$^{13}C$ {$^{1}H$} NMR (126 MHz, $CDCl_3$) spectrum of compound **1f**

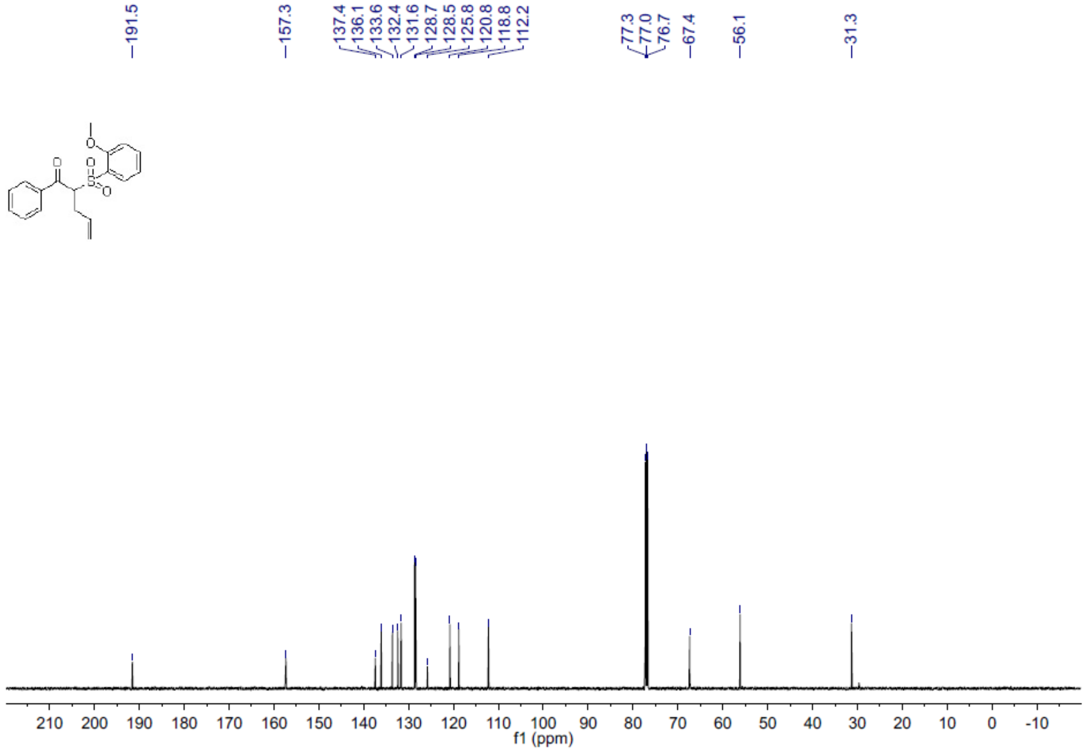

$^{1}$H NMR (500 MHz, $CDCl_3$) spectrum of compound **1h**

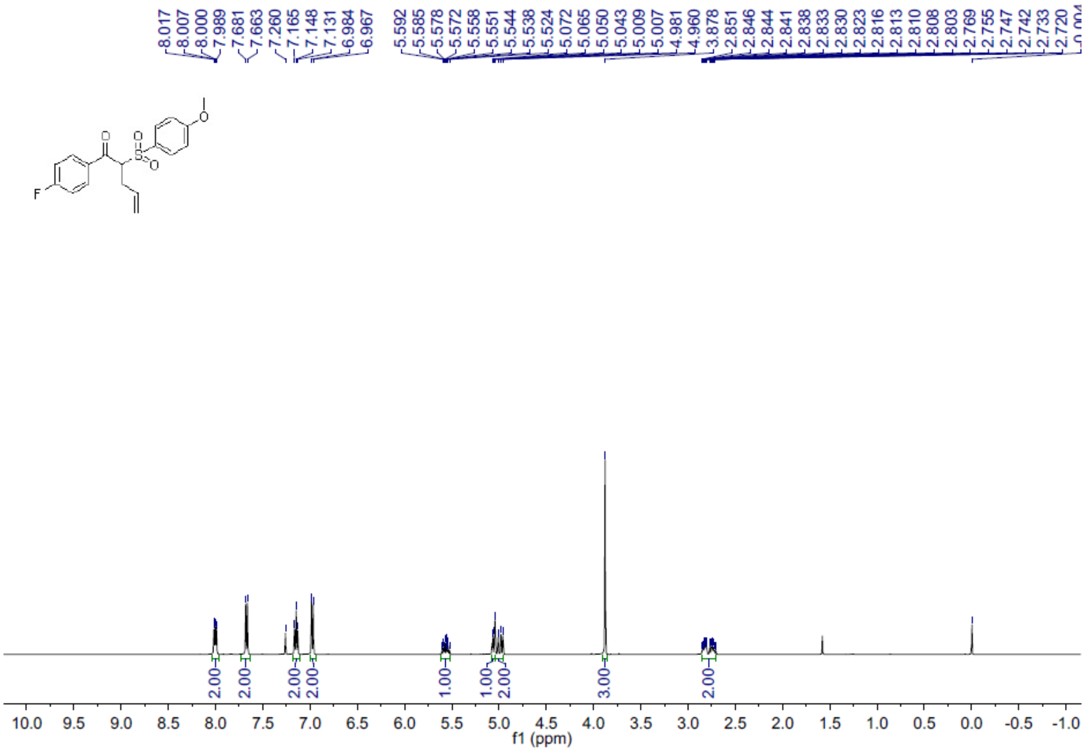


$^{13}$C {$^{1}$H} NMR (126 MHz, $CDCl_3$) spectrum of compound **1h**

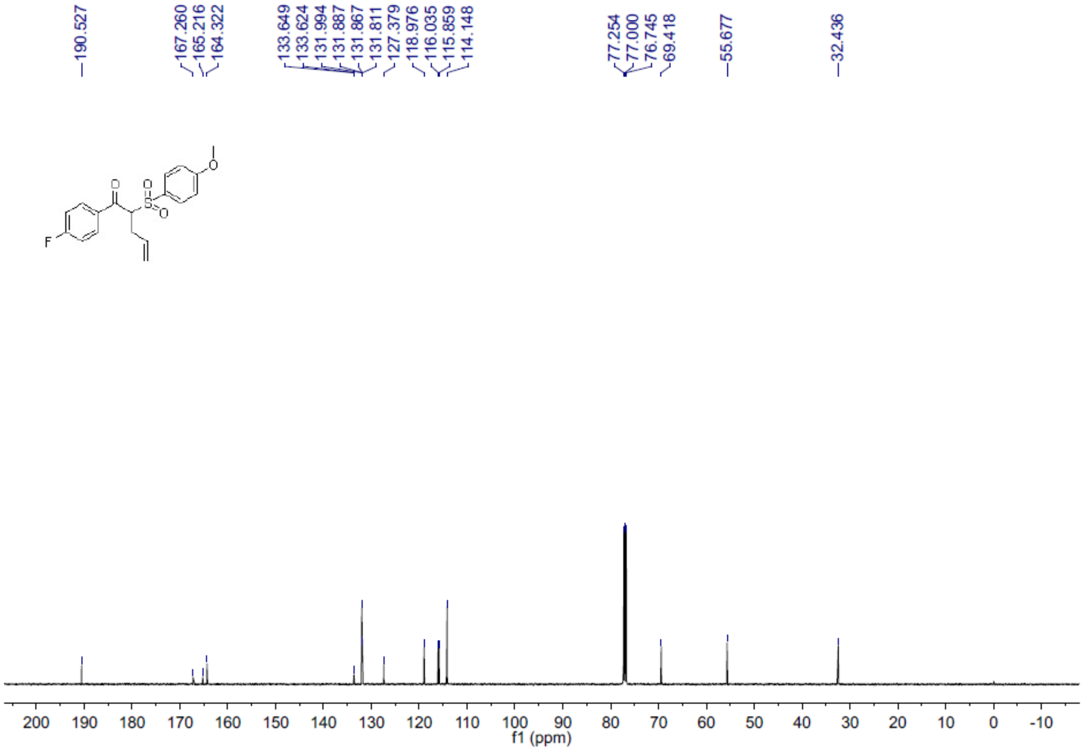

$^{19}$F NMR (470 MHz, $CDCl_3$) spectrum of compound **1h**

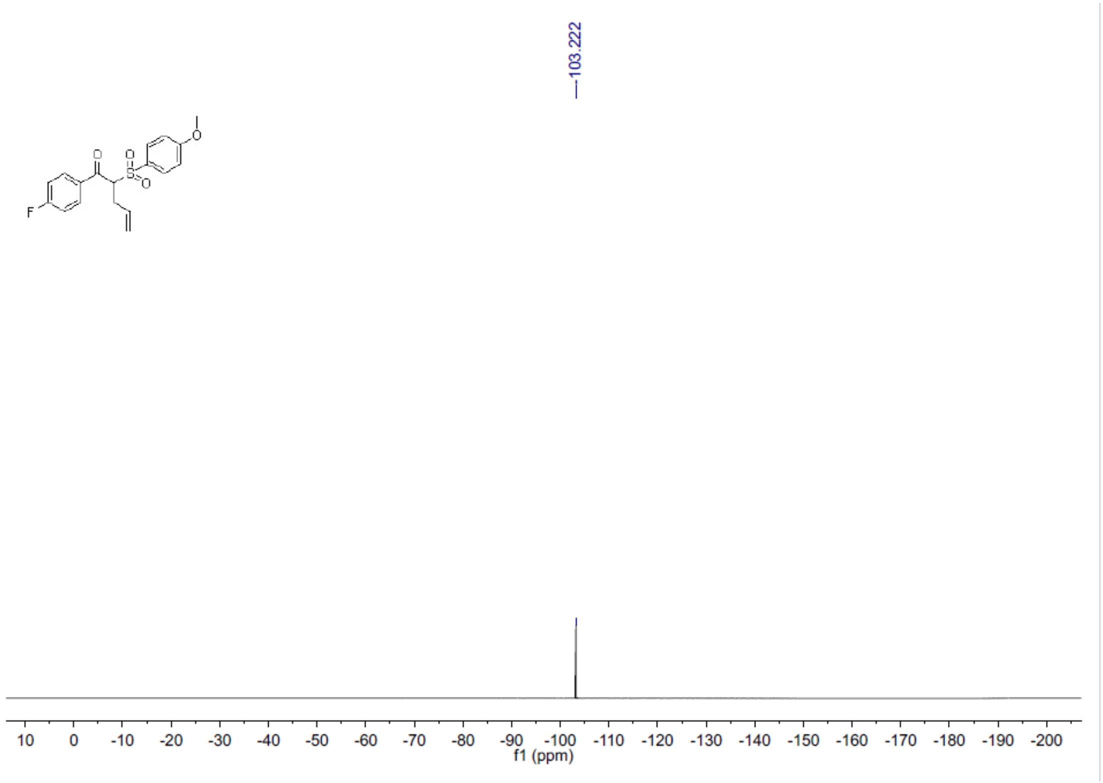


$^{1}$H NMR (500 MHz, $CDCl_3$) spectrum of compound **1i**

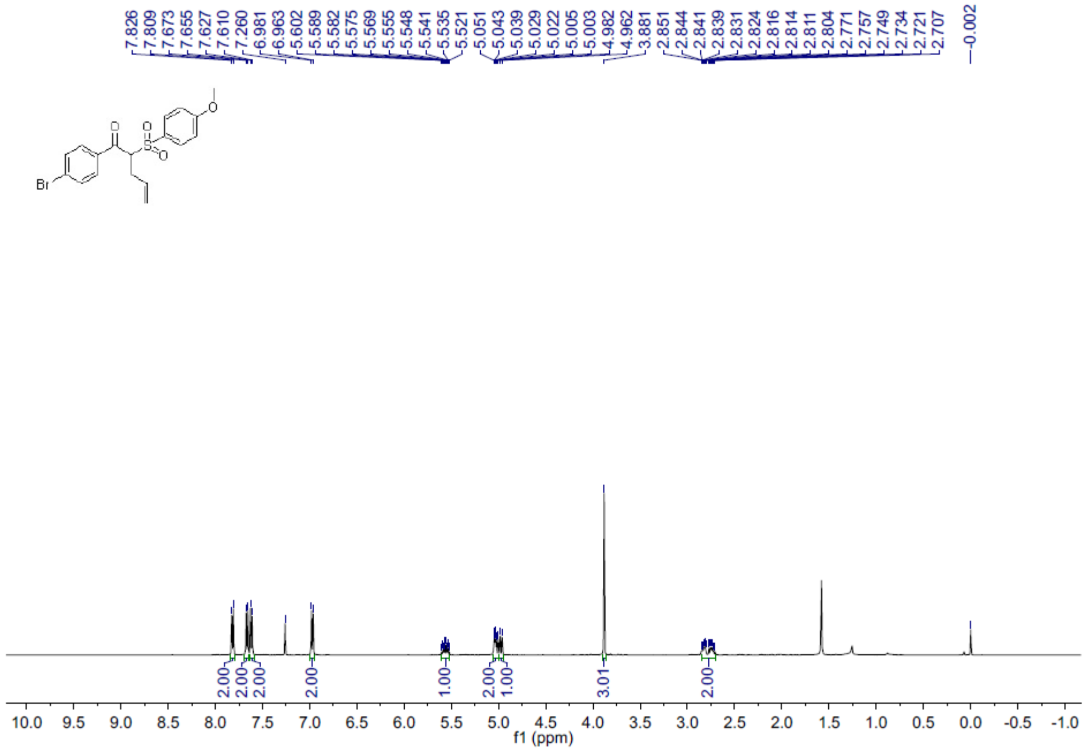

$^{13}C\{^{1}H\}$ NMR (126 MHz, $CDCl_3$) spectrum of compound **1i**

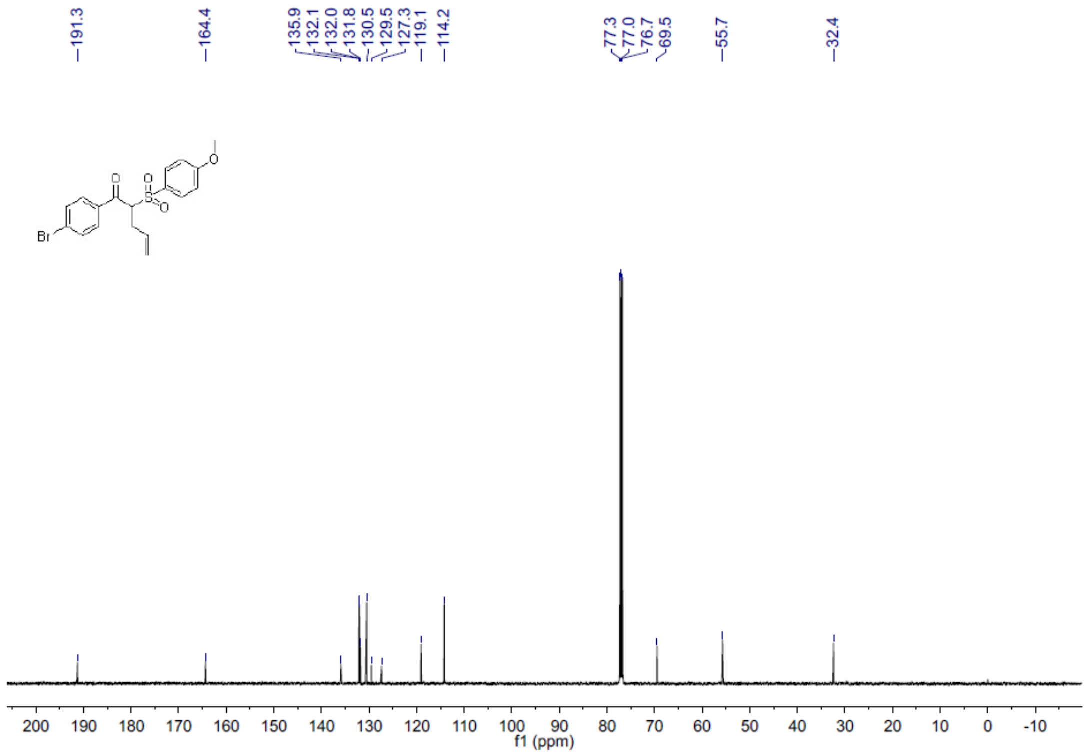


$^{1}H$ NMR (500 MHz, $CDCl_3$) spectrum of compound **1j**

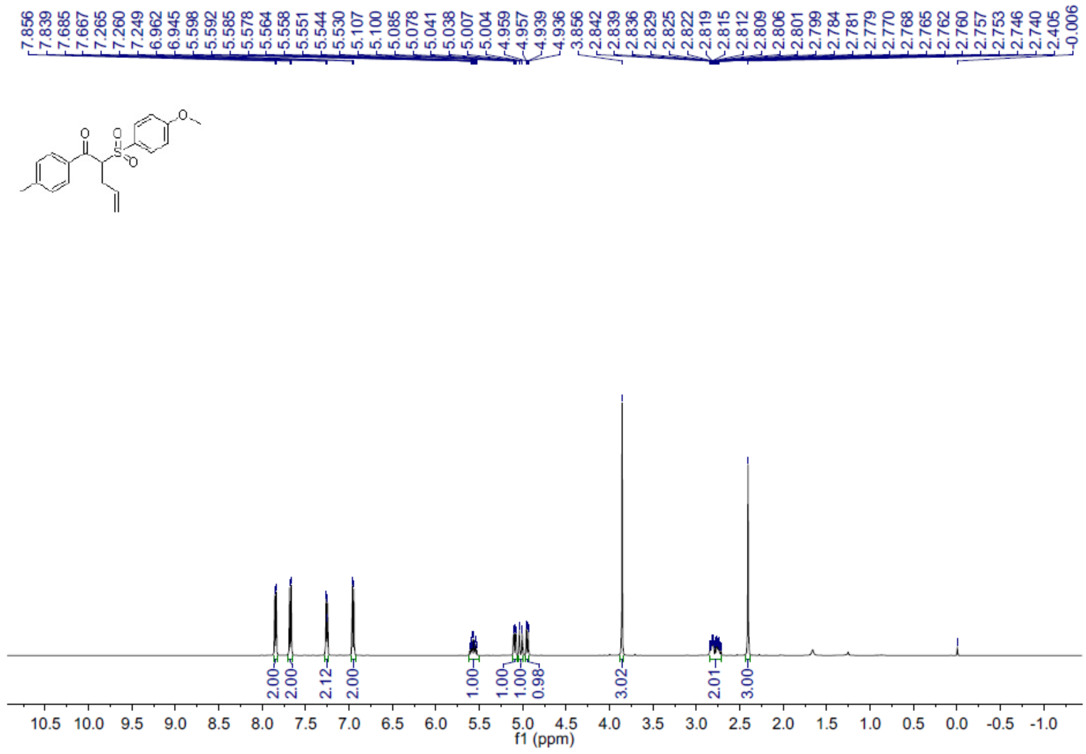

$^{13}C$ {$^{1}H$} NMR (126 MHz, $CDCl_3$) spectrum of compound **1j**

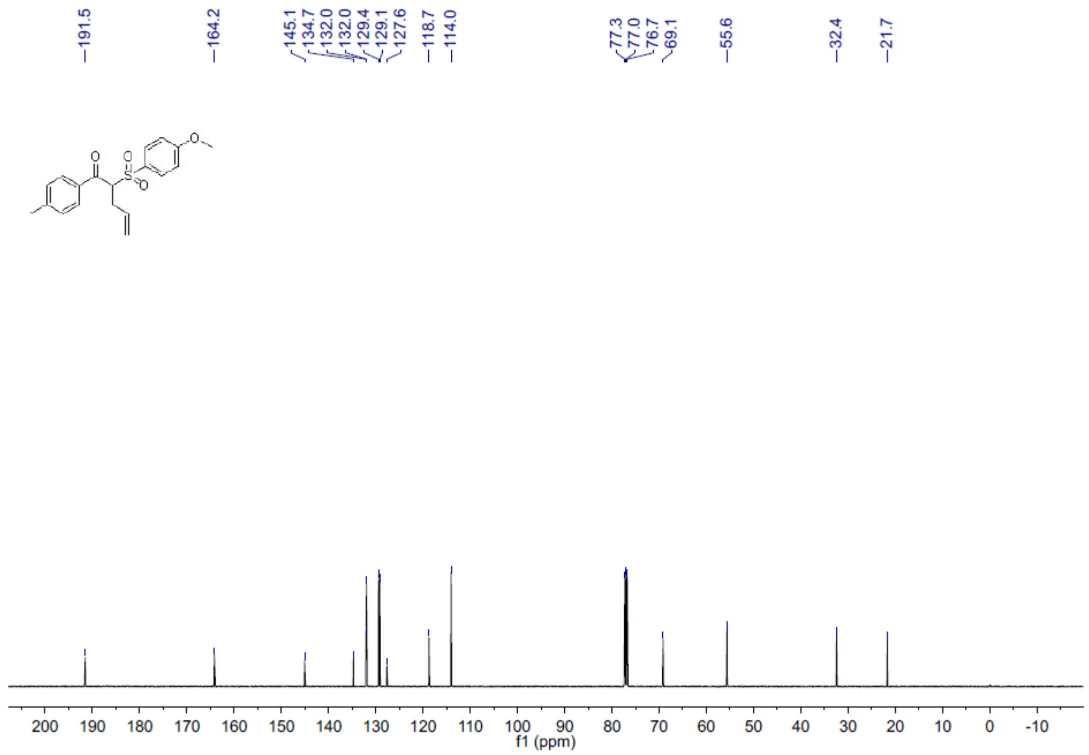


$^{1}H$ NMR (500 MHz, $CDCl_3$) spectrum of compound **1k**

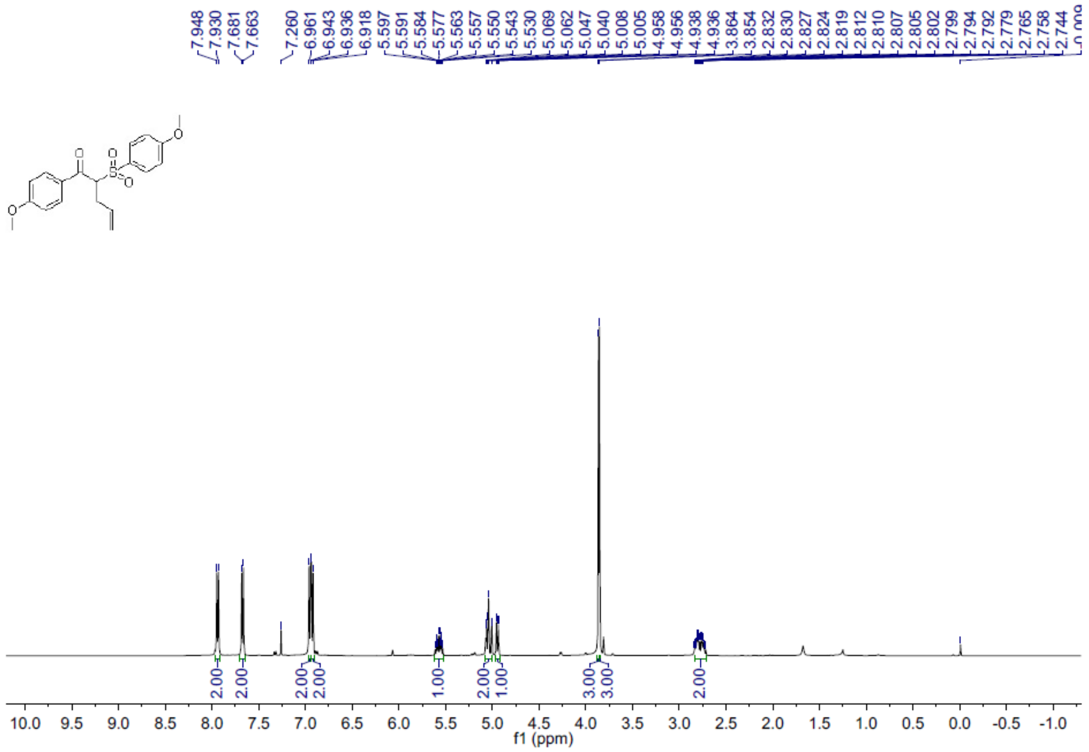

$^{13}C$ {$^{1}H$} NMR (126 MHz, $CDCl_3$) spectrum of compound **1k**

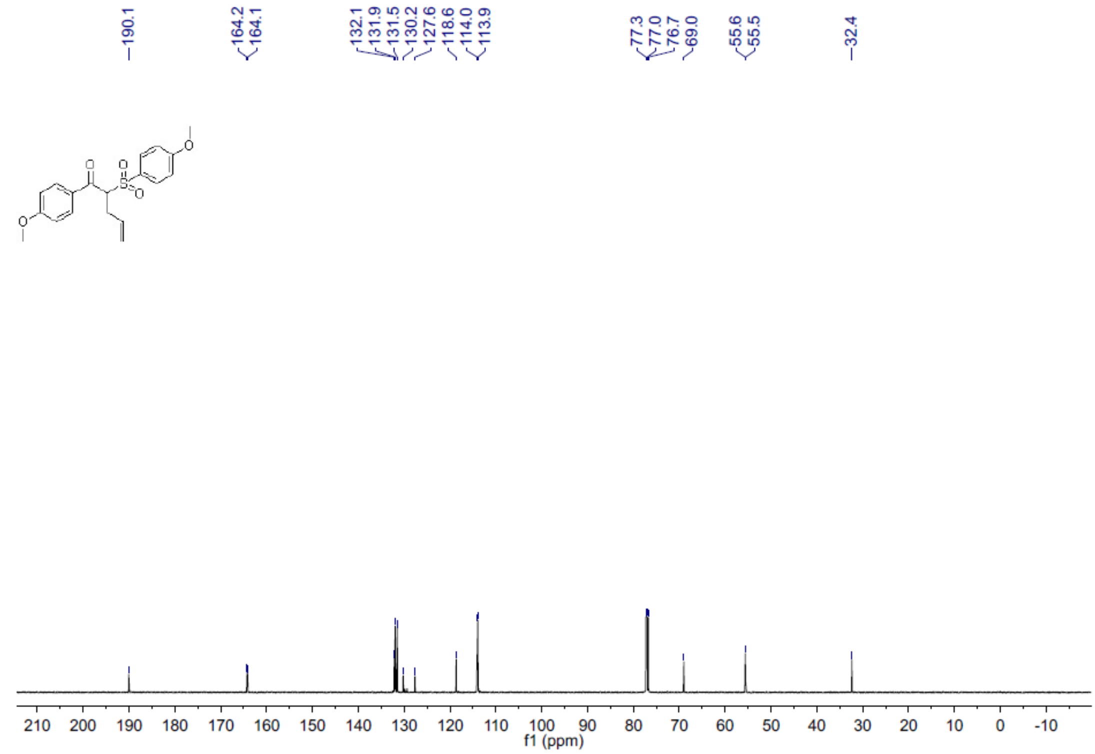


$^{1}H$ NMR (500 MHz, $CDCl_3$) spectrum of compound **1l**

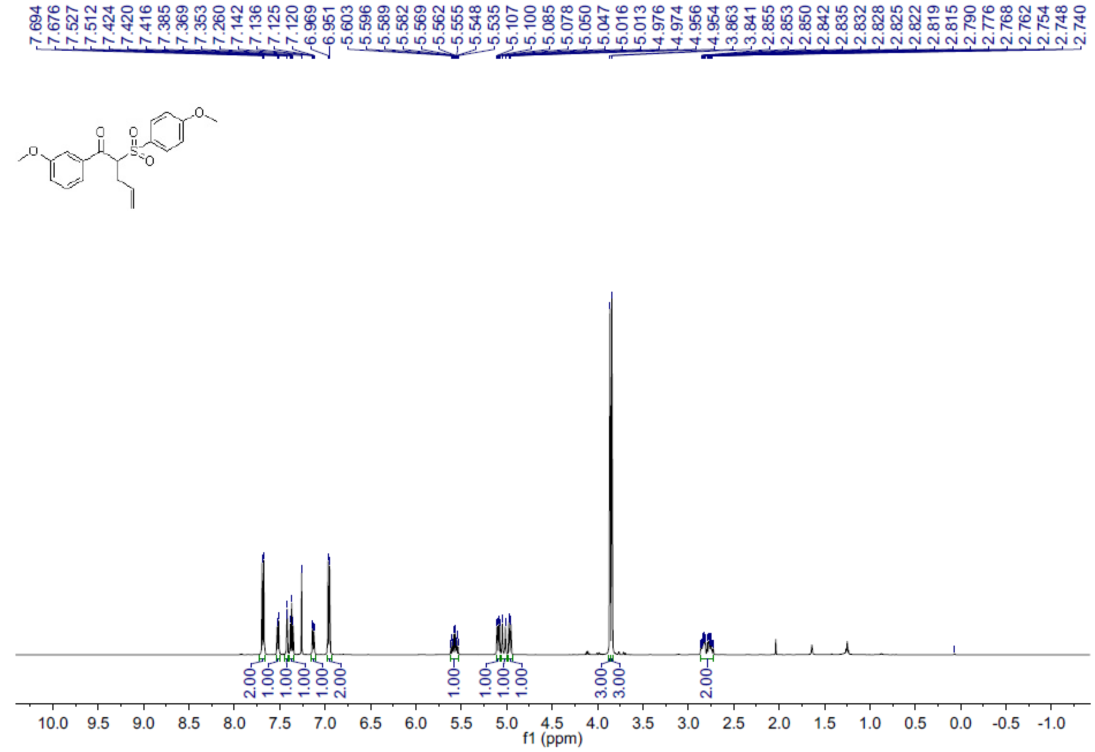

$^{13}C$ {$^{1}H$} NMR (126 MHz, $CDCl_3$) spectrum of compound **1l**

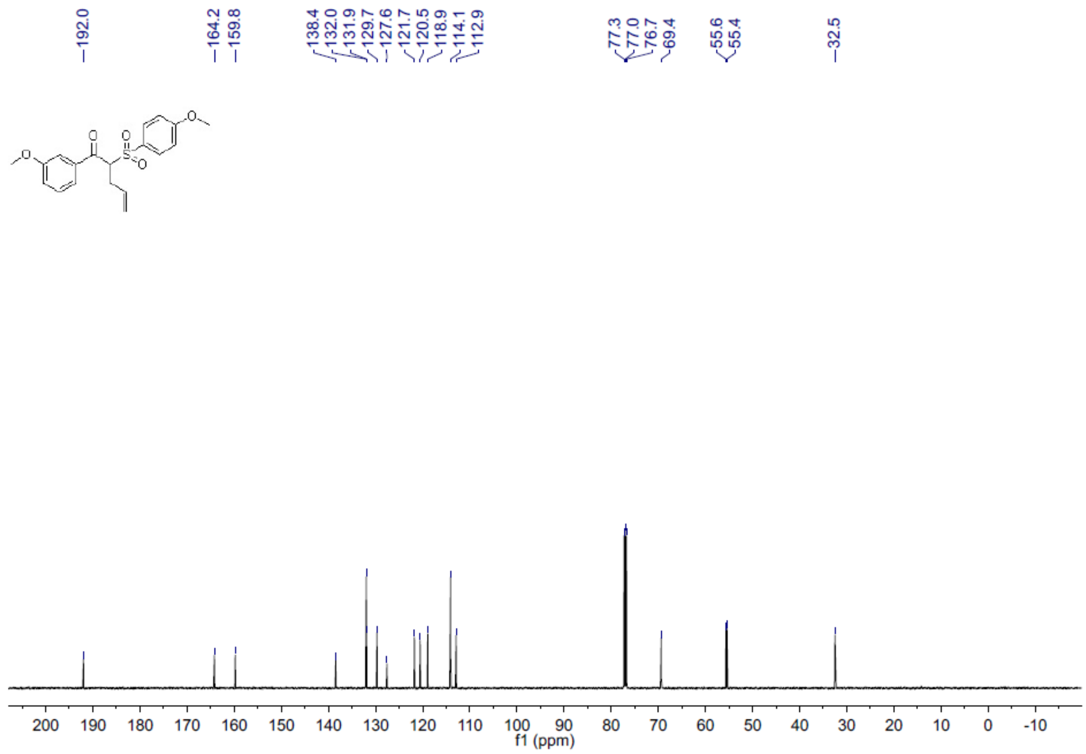


$^{1}H$ NMR (500 MHz, $CDCl_3$) spectrum of compound **1m**

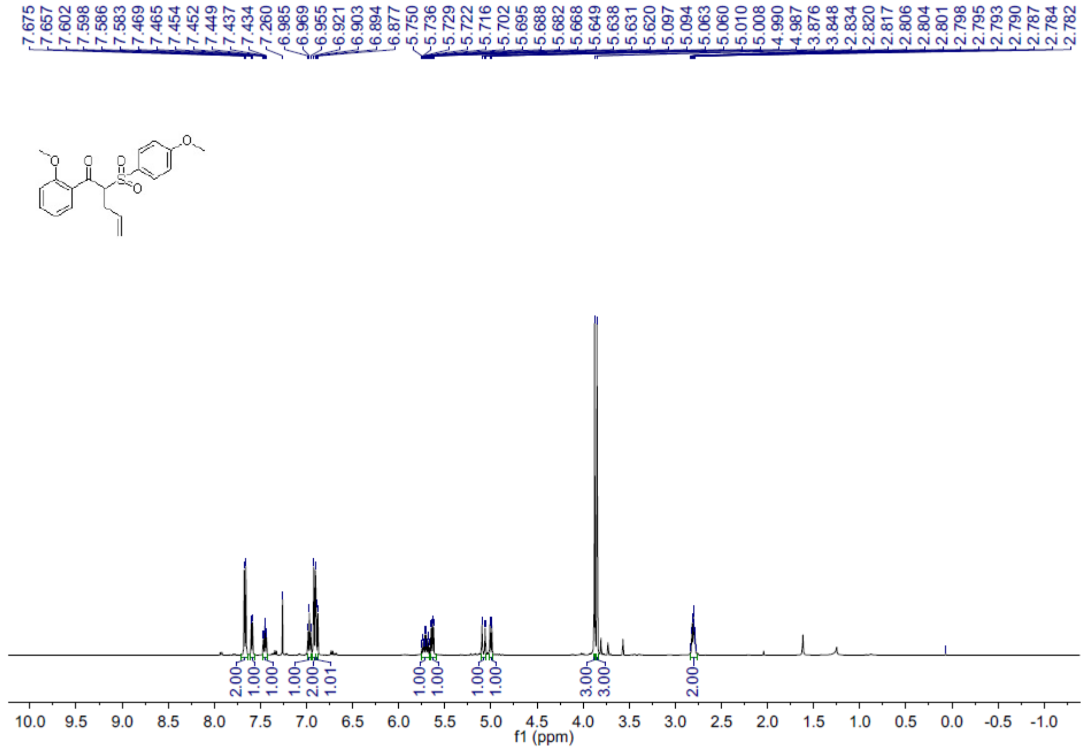

$^{13}C$ {$^{1}H$} NMR (126 MHz, $CDCl_3$) spectrum of compound **1m**

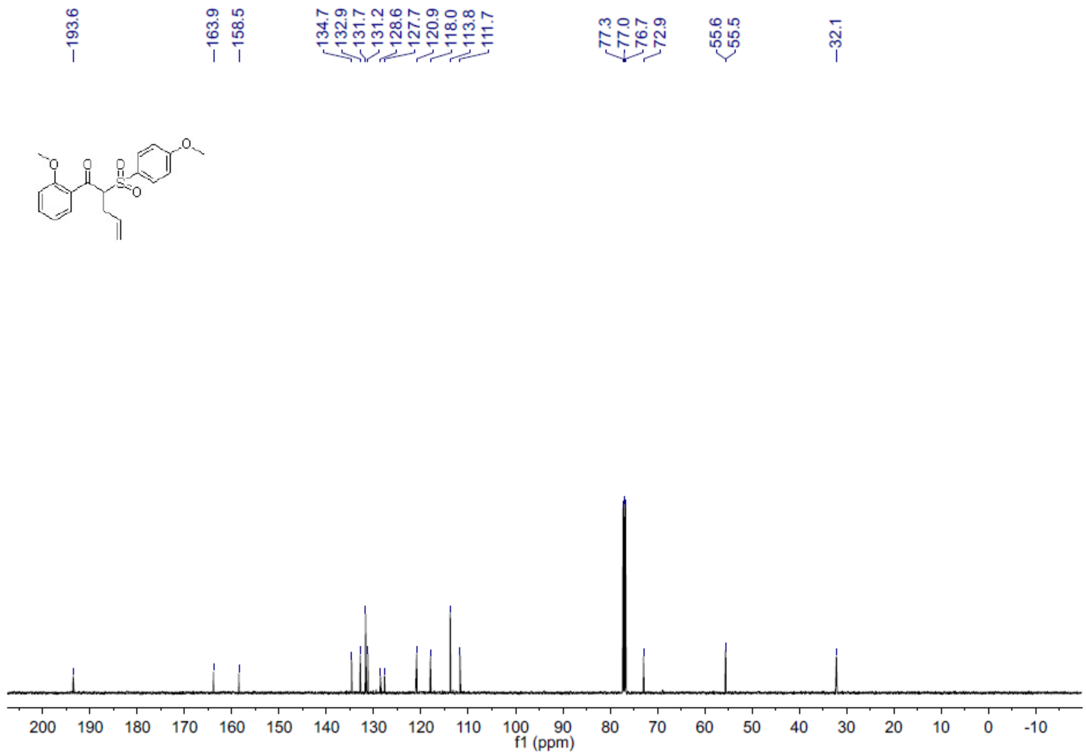


$^{1}H$ NMR (500 MHz, $CDCl_3$) spectrum of compound **1n**

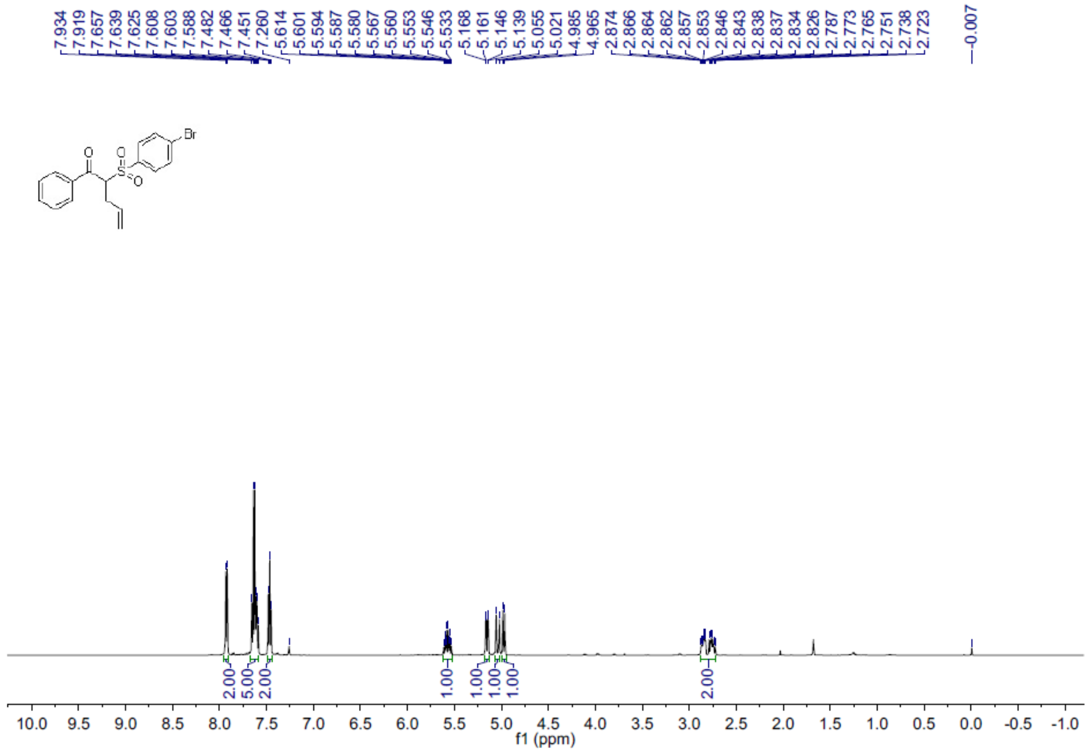

$^{13}C\ \{^{1}H\}$ NMR (126 MHz, $CDCl_3$) spectrum of compound **1n**

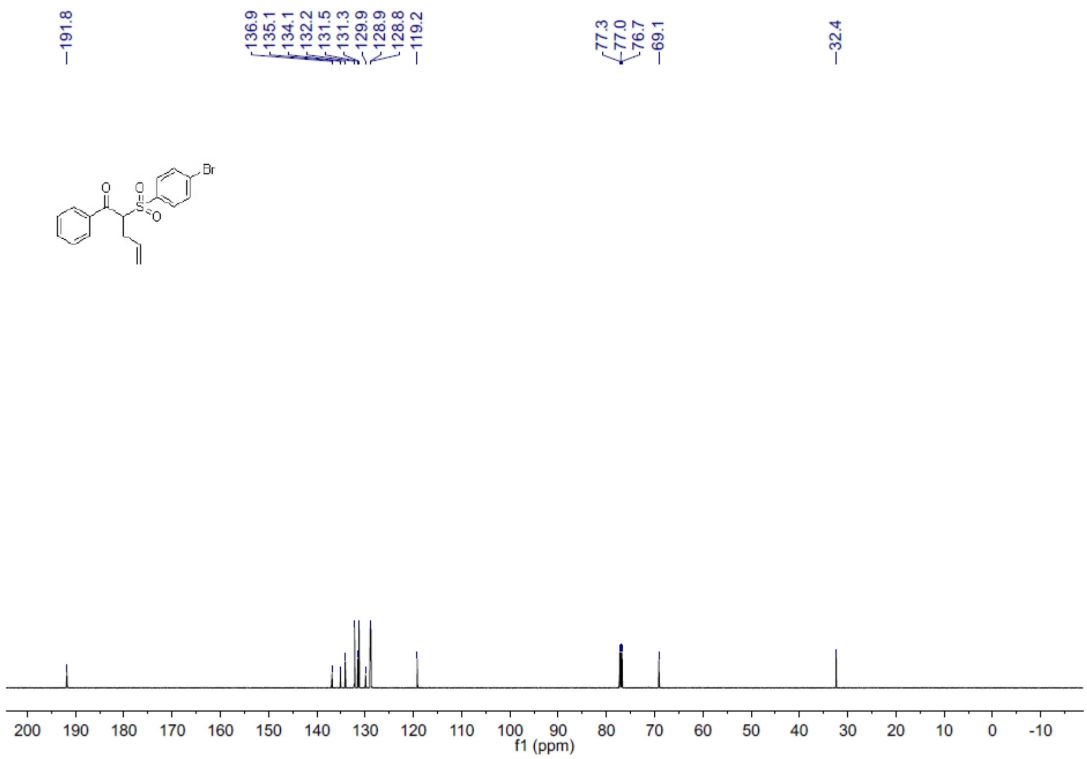

$^{1}H$ NMR (500 MHz, $CDCl_3$) spectrum of compound **1o**

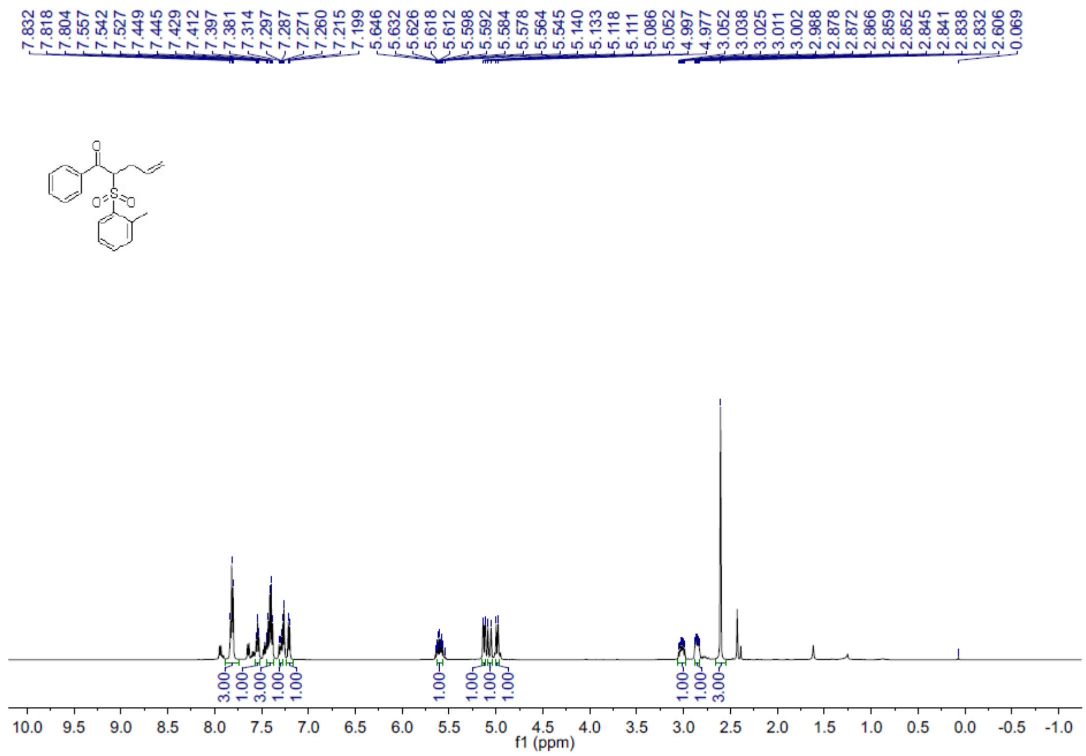

$^{13}C\ \{^{1}H\}$ NMR (126 MHz, $CDCl_3$) spectrum of compound **1o**

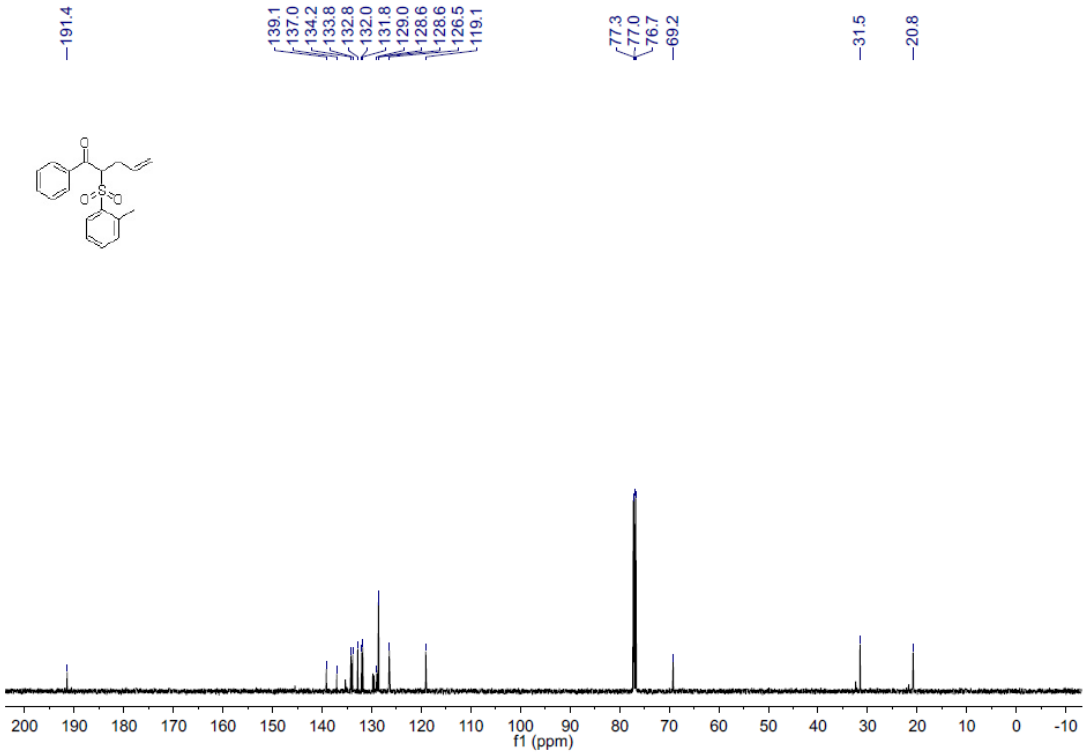


$^{1}H$ NMR (500 MHz, $CDCl_3$) spectrum of compound **1s**

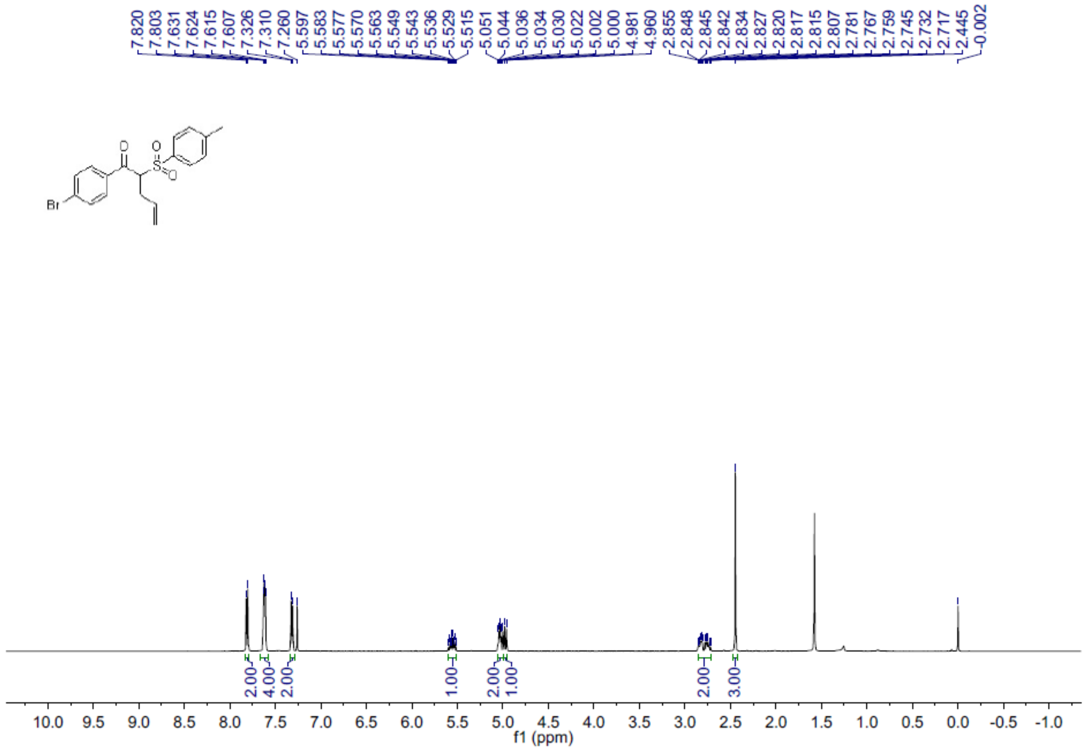

$^{13}C\{^1H\}$ NMR (126 MHz, $CDCl_3$) spectrum of compound **1s**

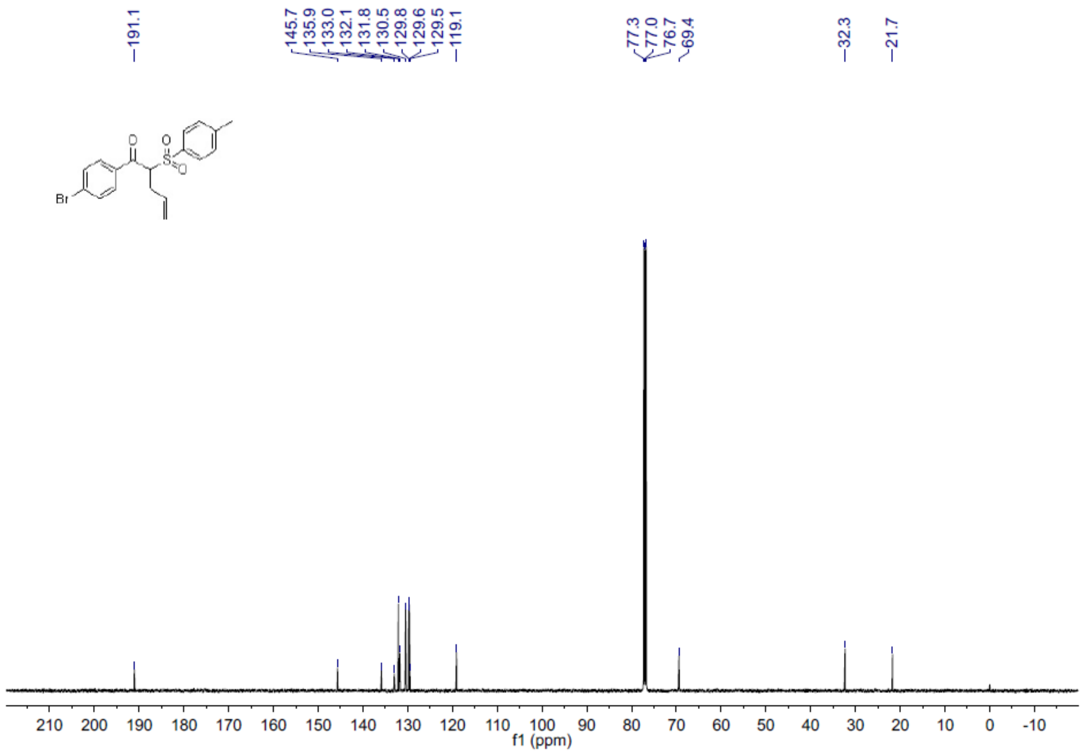

$^1H$ NMR (500 MHz, $CDCl_3$) spectrum of compound **1t**

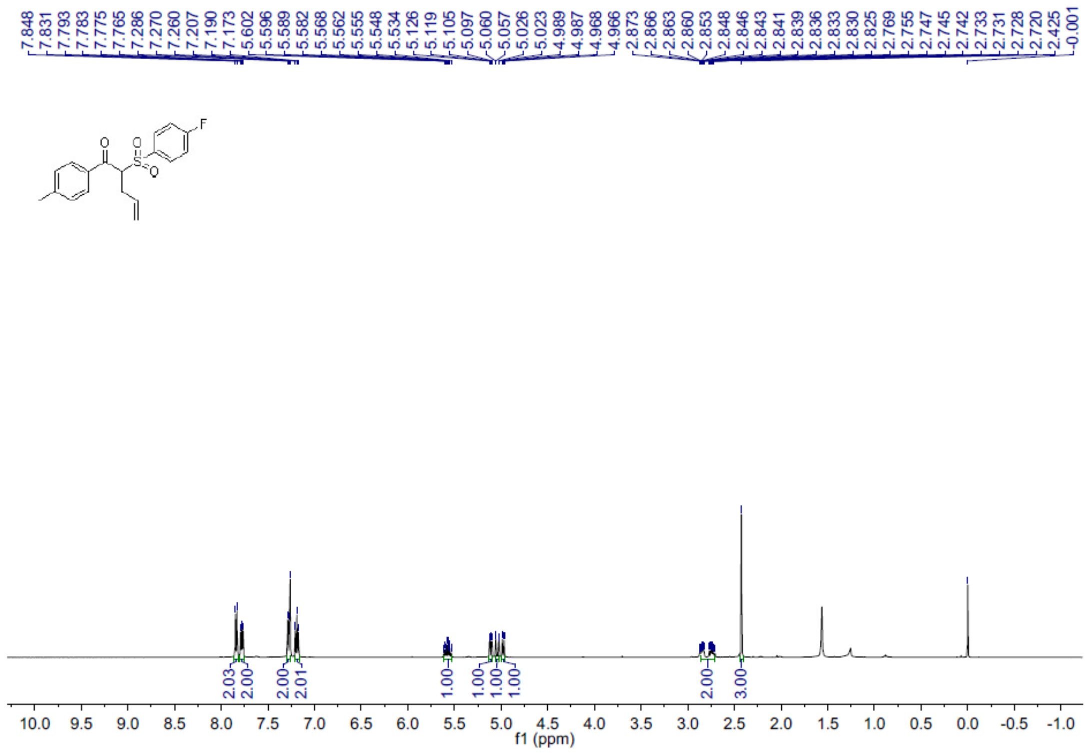

$^{13}C$ {$^{1}H$} NMR (126 MHz, $CDCl_3$) spectrum of compound **1t**

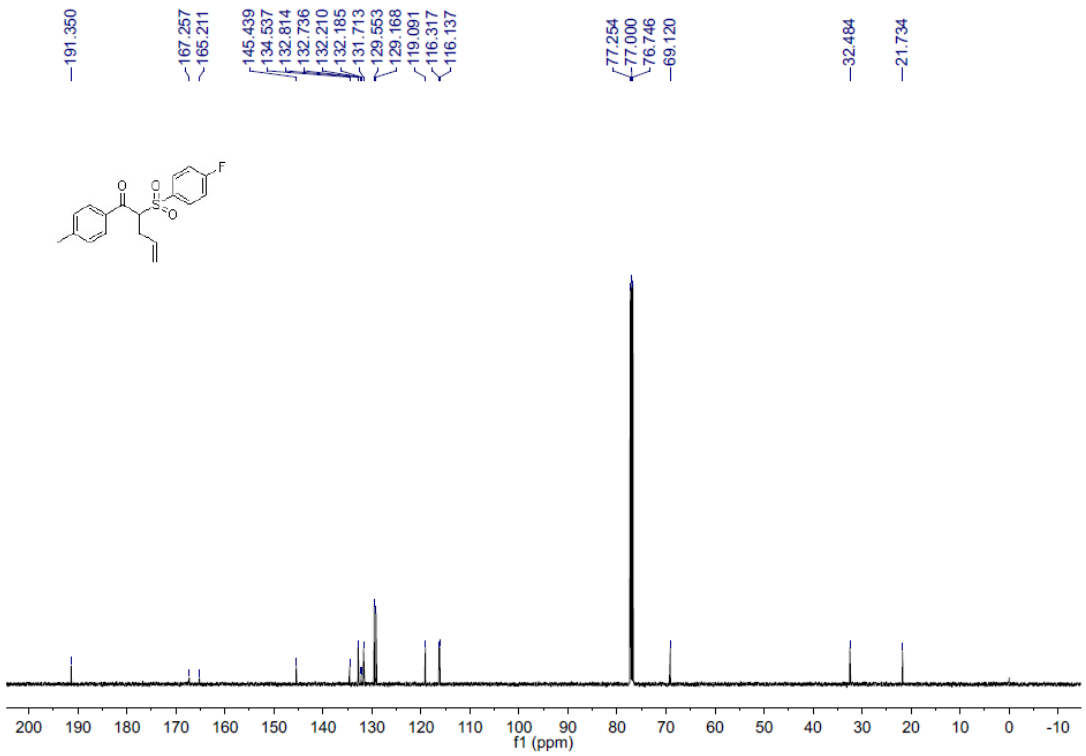


$^{19}F$ NMR (470 MHz, $CDCl_3$) spectrum of compound **1t**

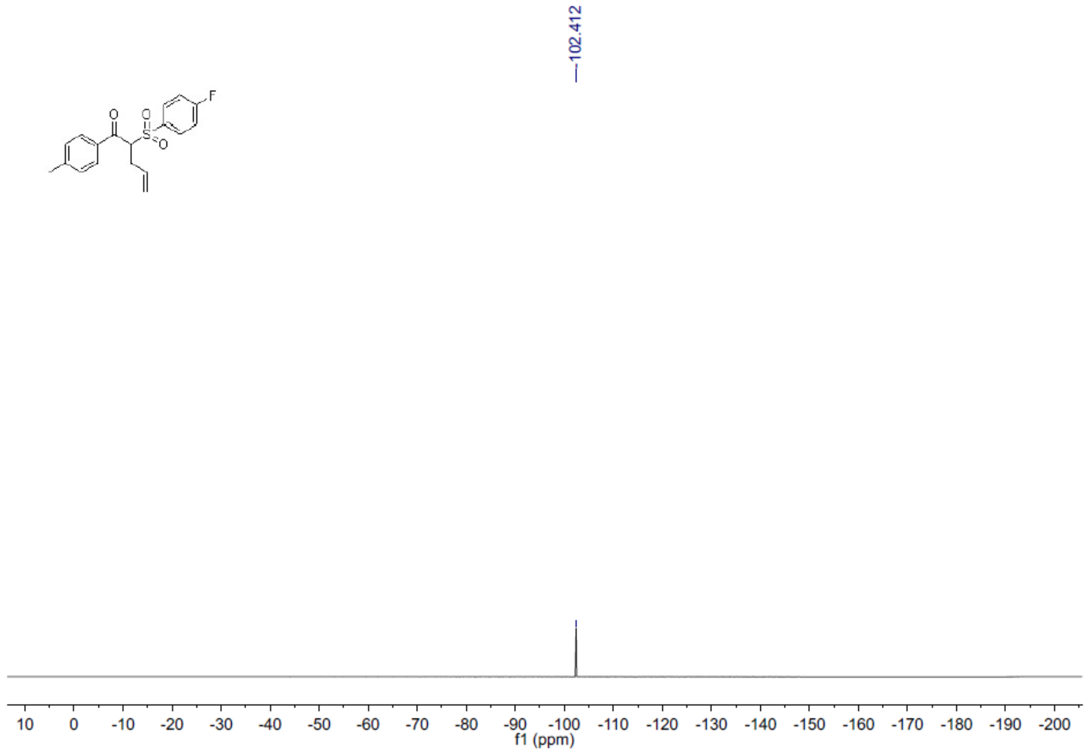

$^{1}H$ NMR (500 MHz, $CDCl_3$) spectrum of compound **1u**

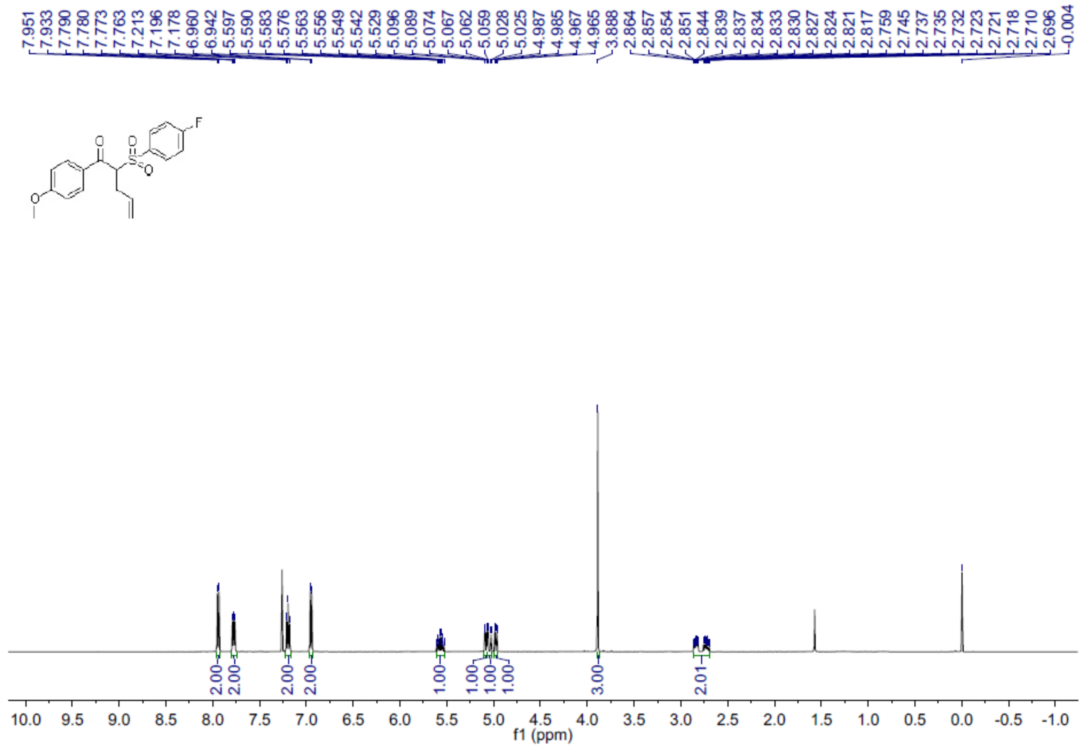


$^{13}C$ {$^{1}H$} NMR (126 MHz, $CDCl_3$) spectrum of compound **1u**

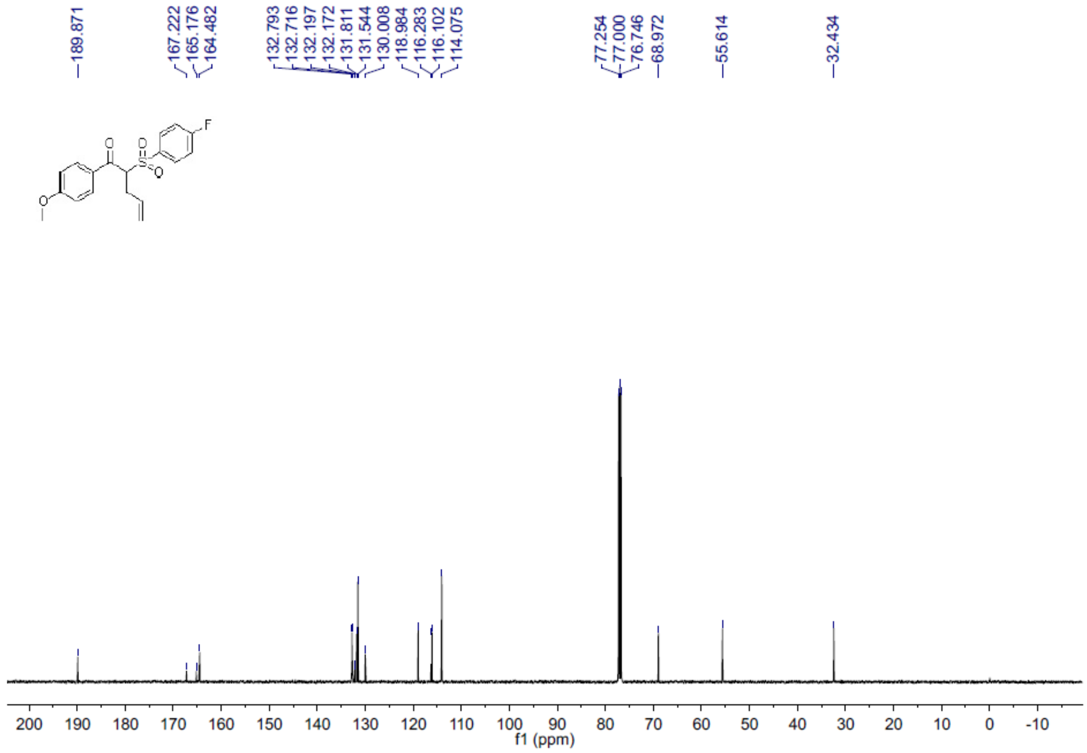

$^{19}$F NMR (470 MHz, $CDCl_3$) spectrum of compound **1u**

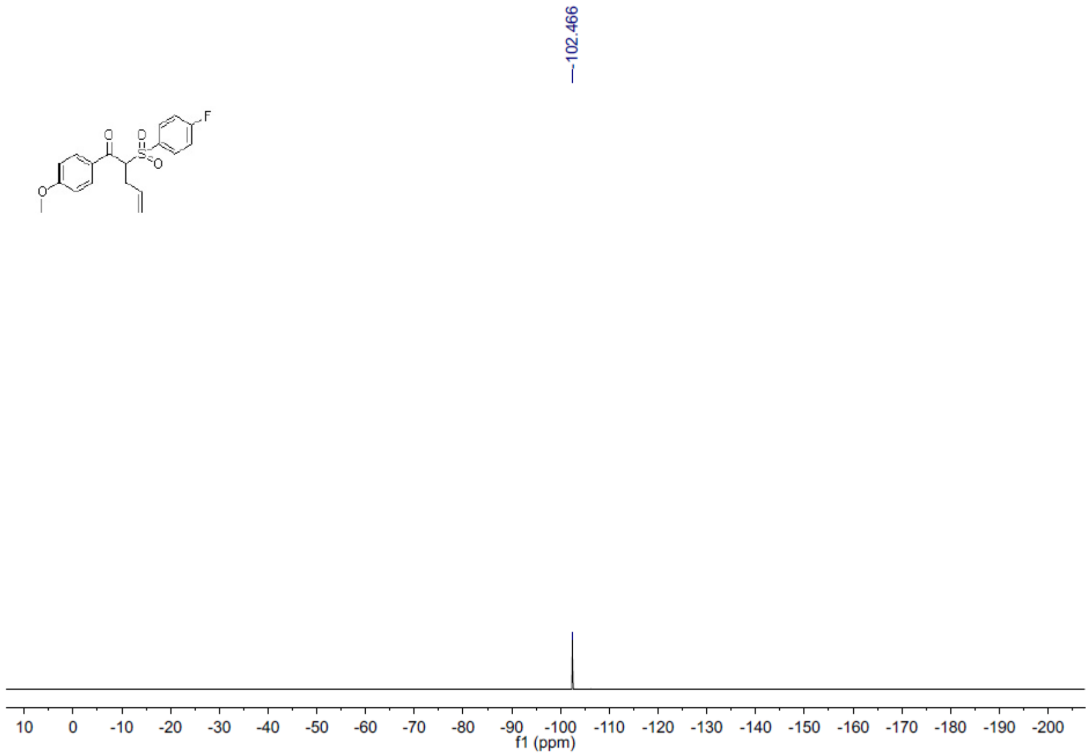

$^{1}$H NMR (500 MHz, $CDCl_3$) spectrum of compound **1v**

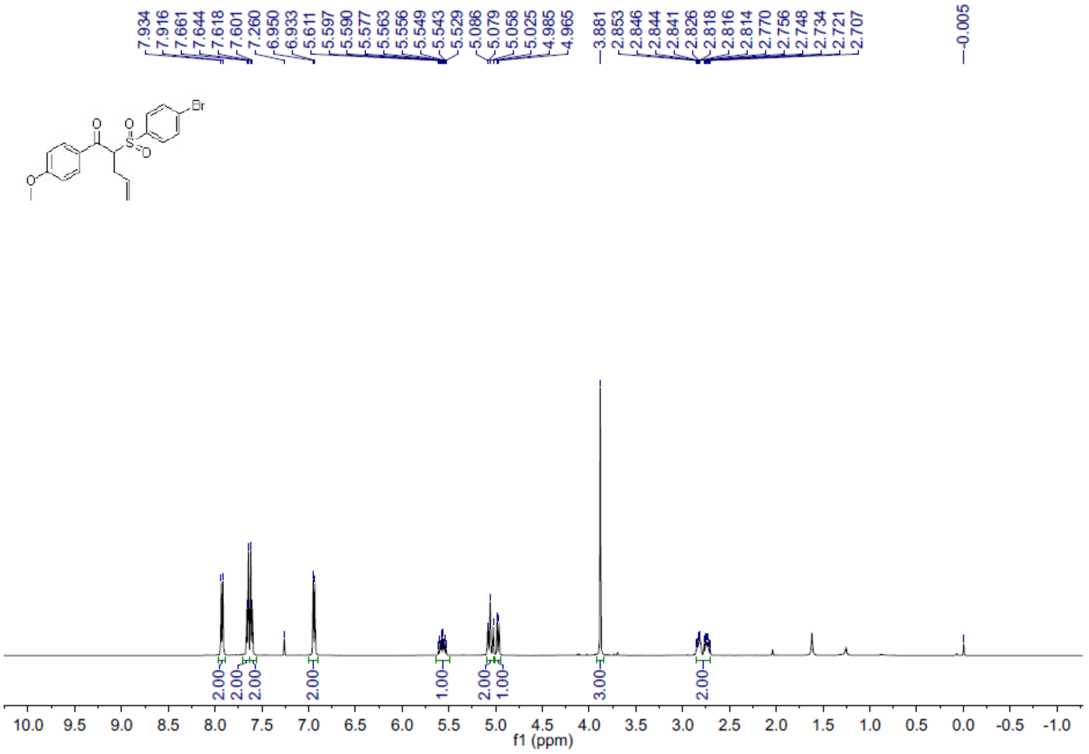

$^{13}C\ \{^1H\}$ NMR (126 MHz, $CDCl_3$) spectrum of compound **1v**

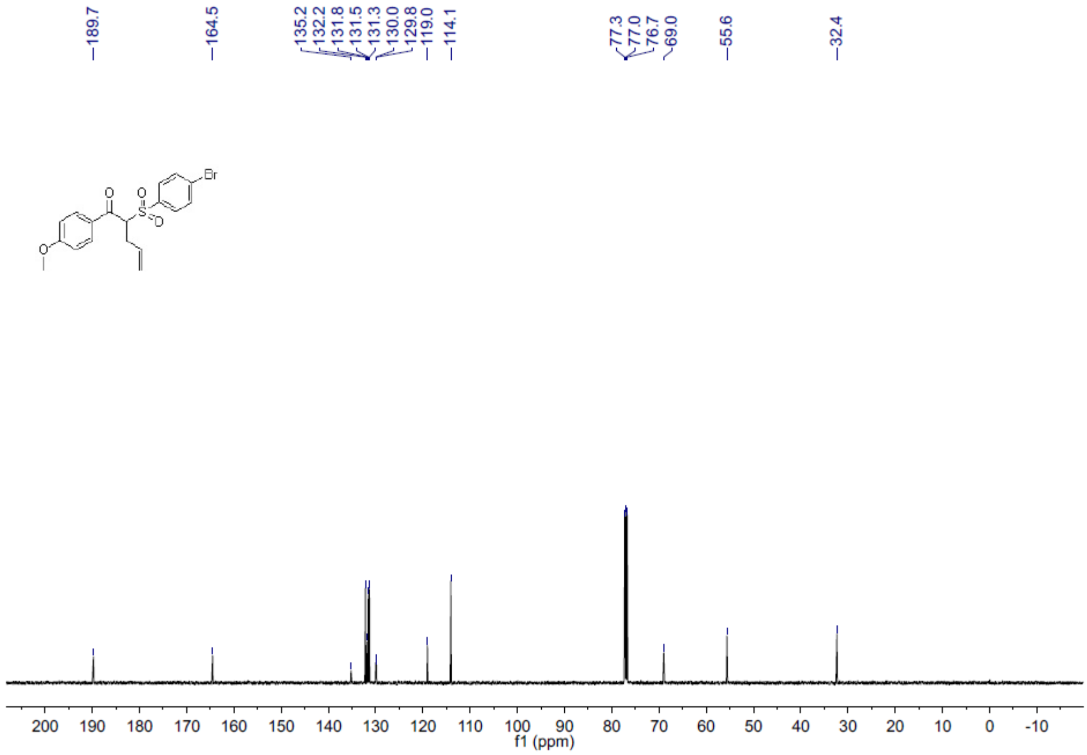


$^1H$ NMR (500 MHz, $CDCl_3$) spectrum of compound **1w**

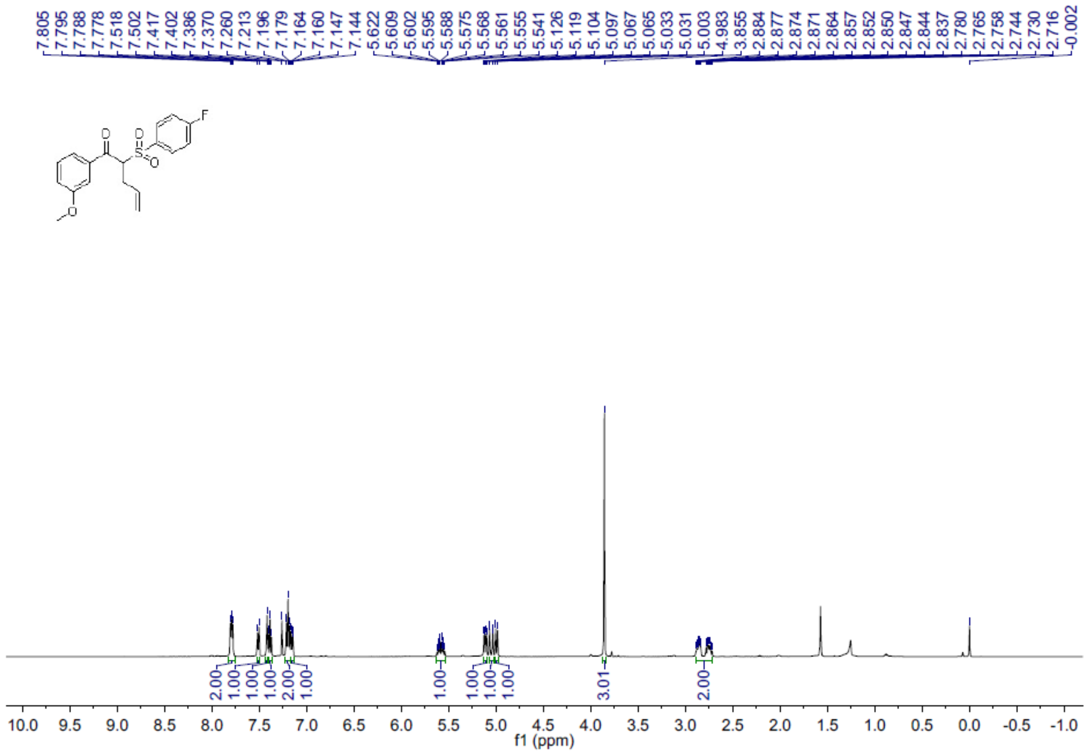

$^{13}C$ {$^{1}H$} NMR (126 MHz, $CDCl_3$) spectrum of compound **1w**

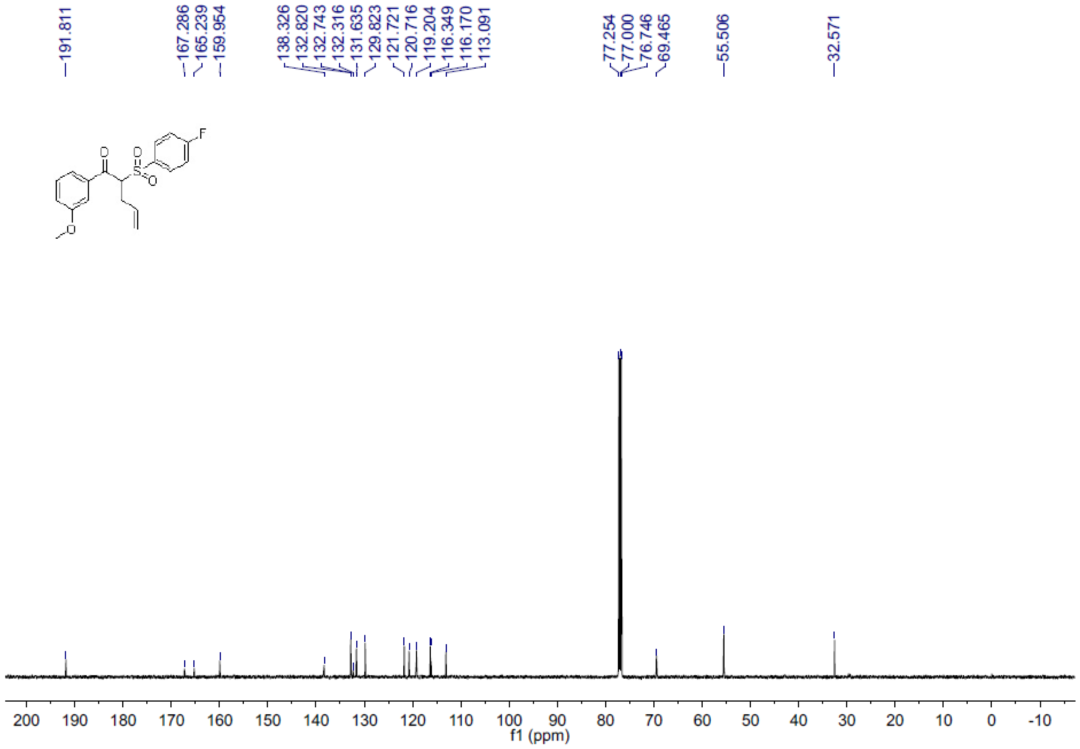


$^{19}F$ NMR (470 MHz, $CDCl_3$) spectrum of compound **1w**

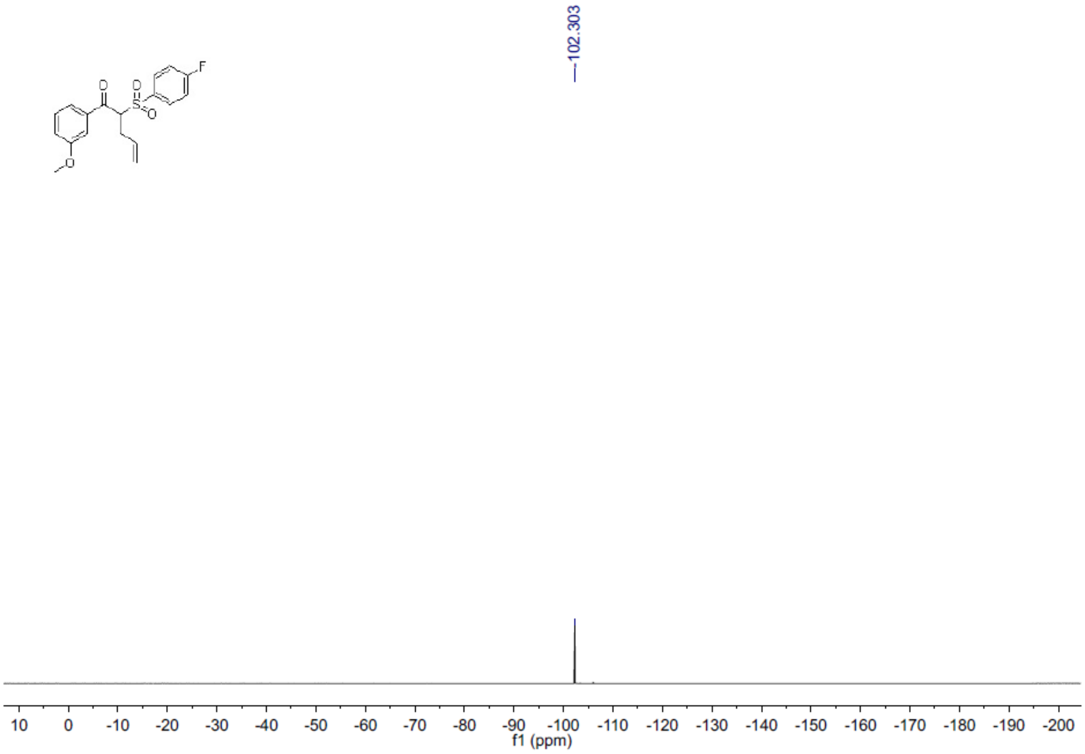

$^{1}$H NMR (500 MHz, $CDCl_3$) spectrum of compound **1x**

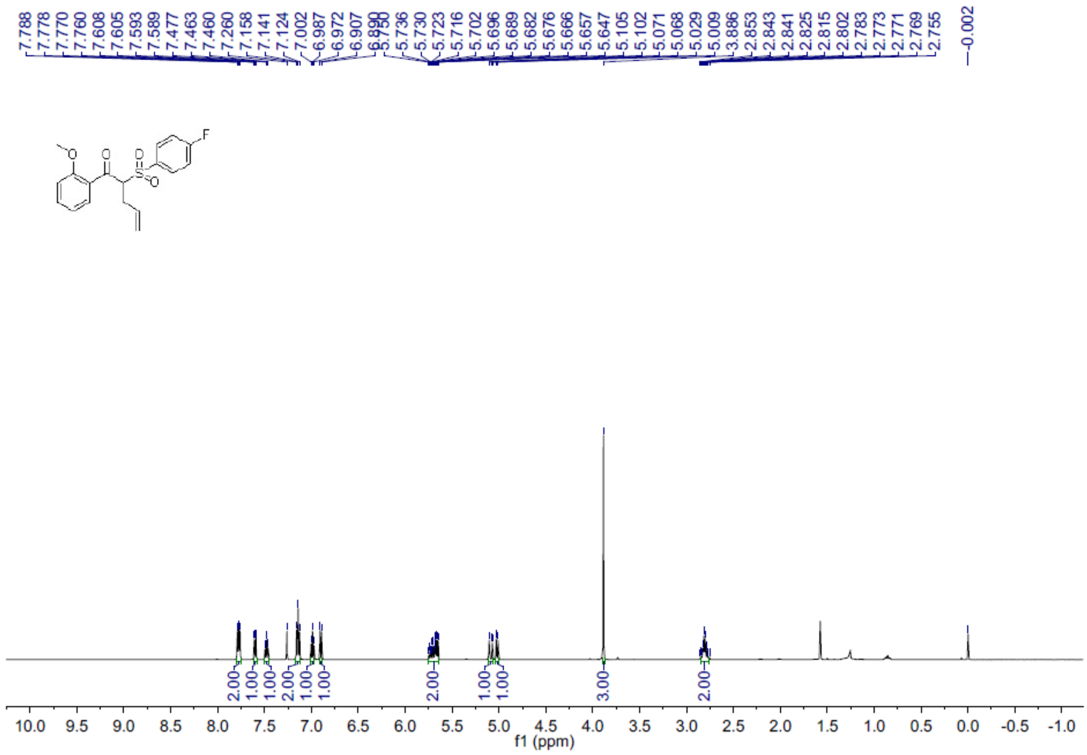


$^{13}$C {$^{1}$H} NMR (126 MHz, $CDCl_3$) spectrum of compound **1x**

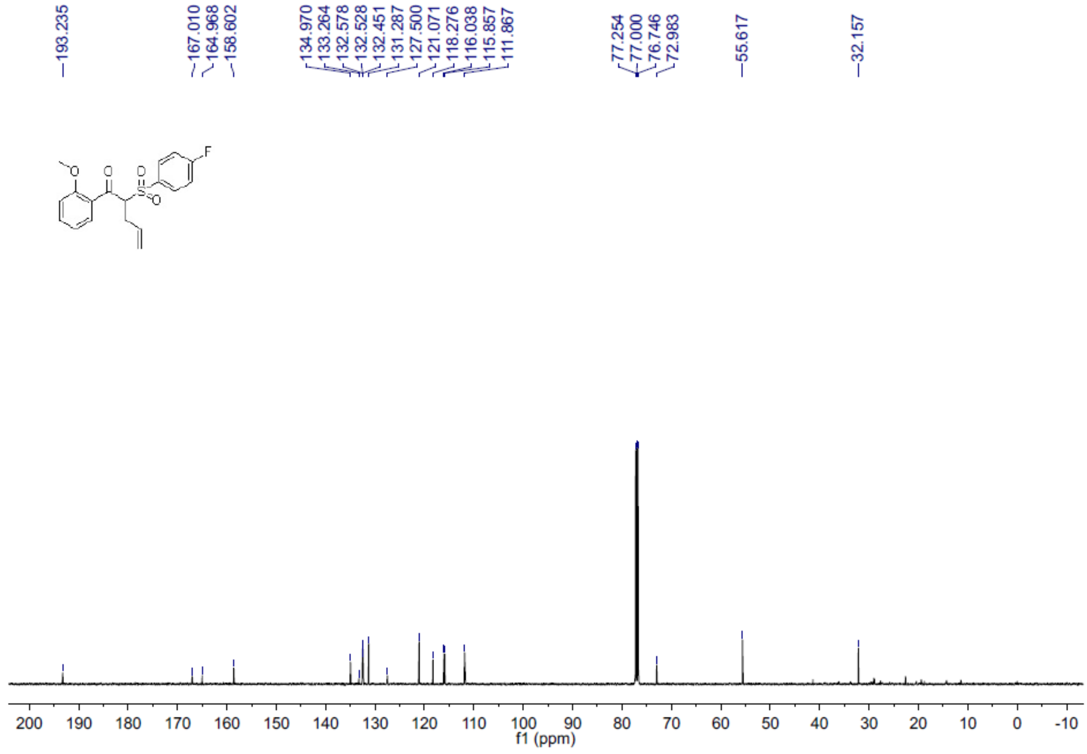

$^{19}$F NMR (470 MHz, CDCl3) spectrum of compound **1x**

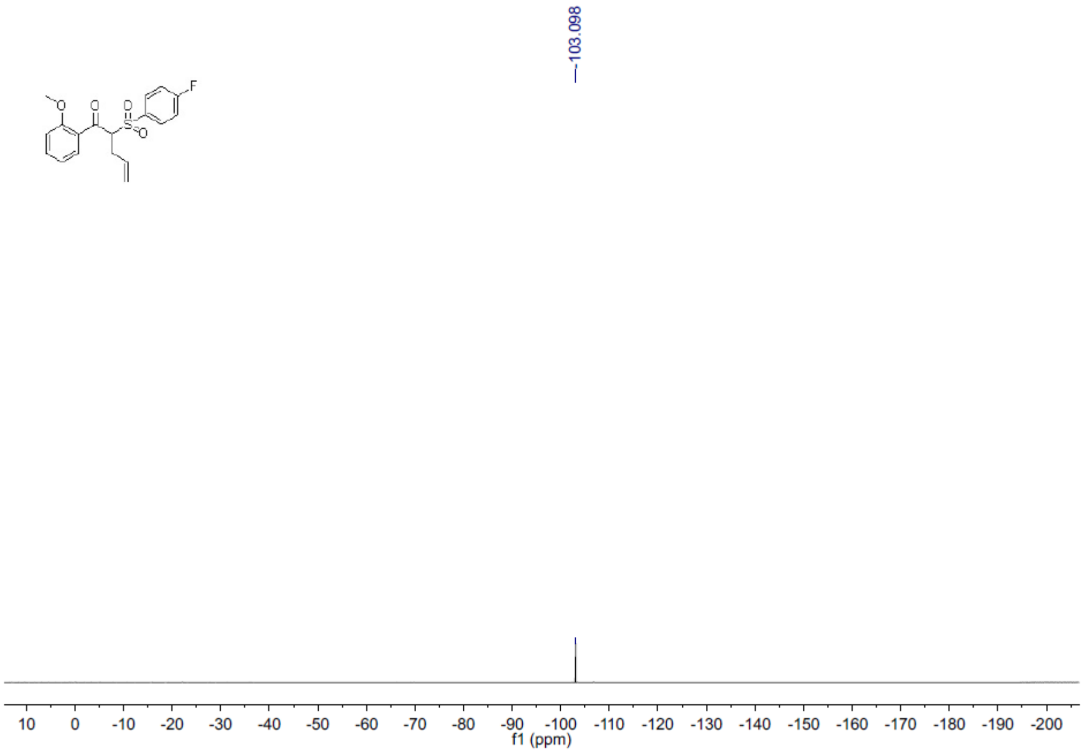


$^{1}$H NMR (500 MHz, CDCl3) spectrum of compound **3a**

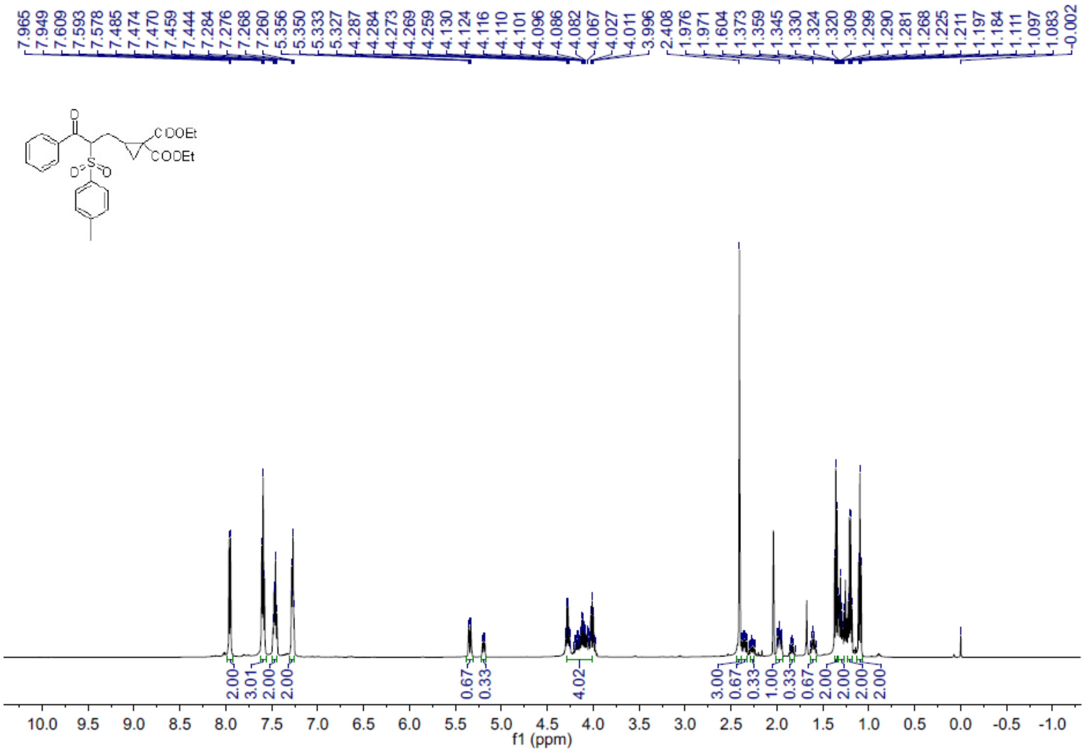

$^{13}C$ {$^{1}H$} NMR (126 MHz, $CDCl_3$) spectrum of compound **3a**

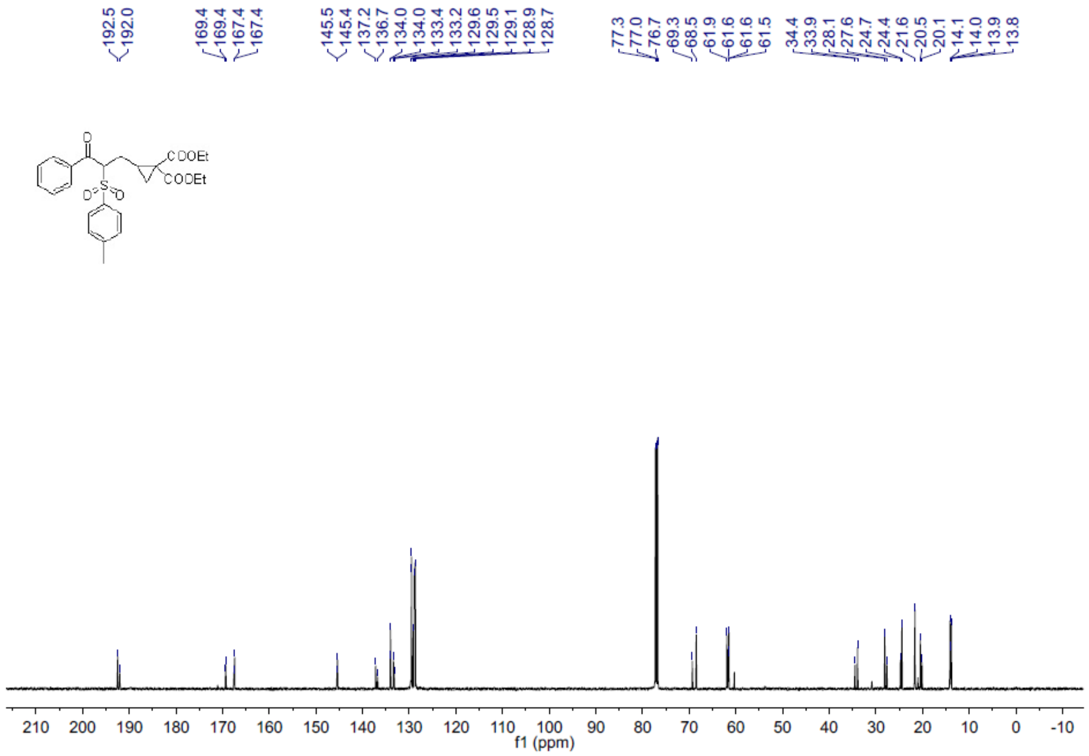


DEPT 135 NMR ($CDCl_3$) spectrum of compound **3a**

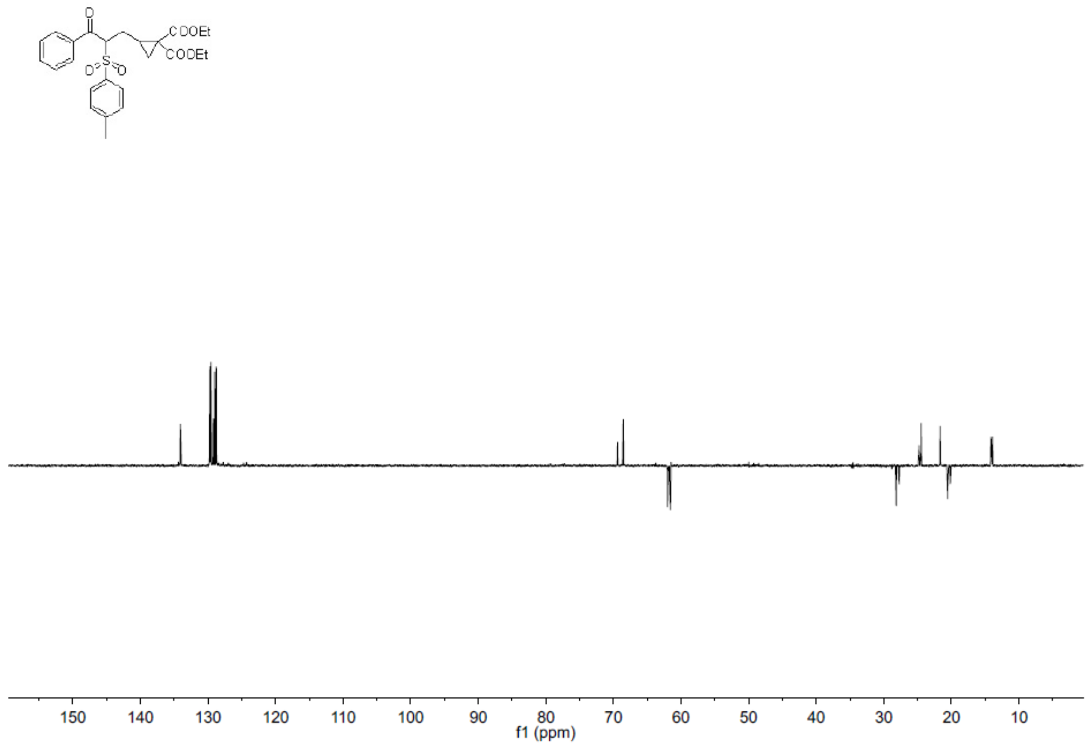

$^{1}$H-$^{1}$H COSY NMR ($CDCl_3$) spectrum of compound **3a**

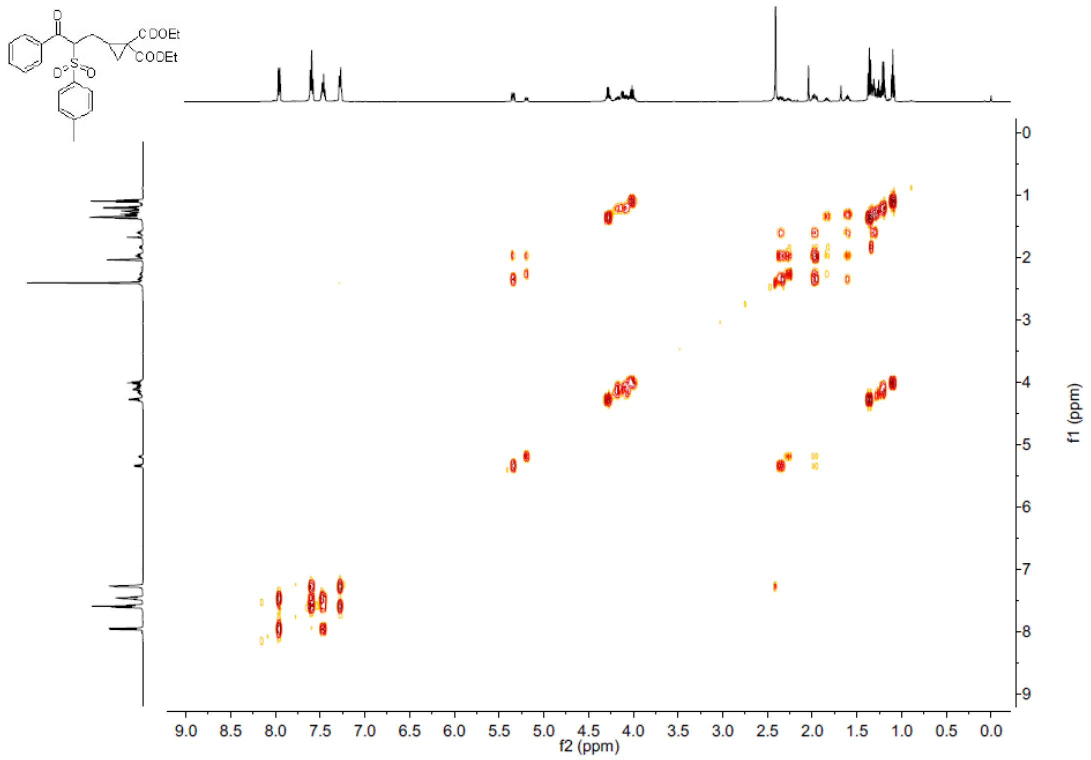


HSQC NMR ($CDCl_3$) spectrum of compound **3a**

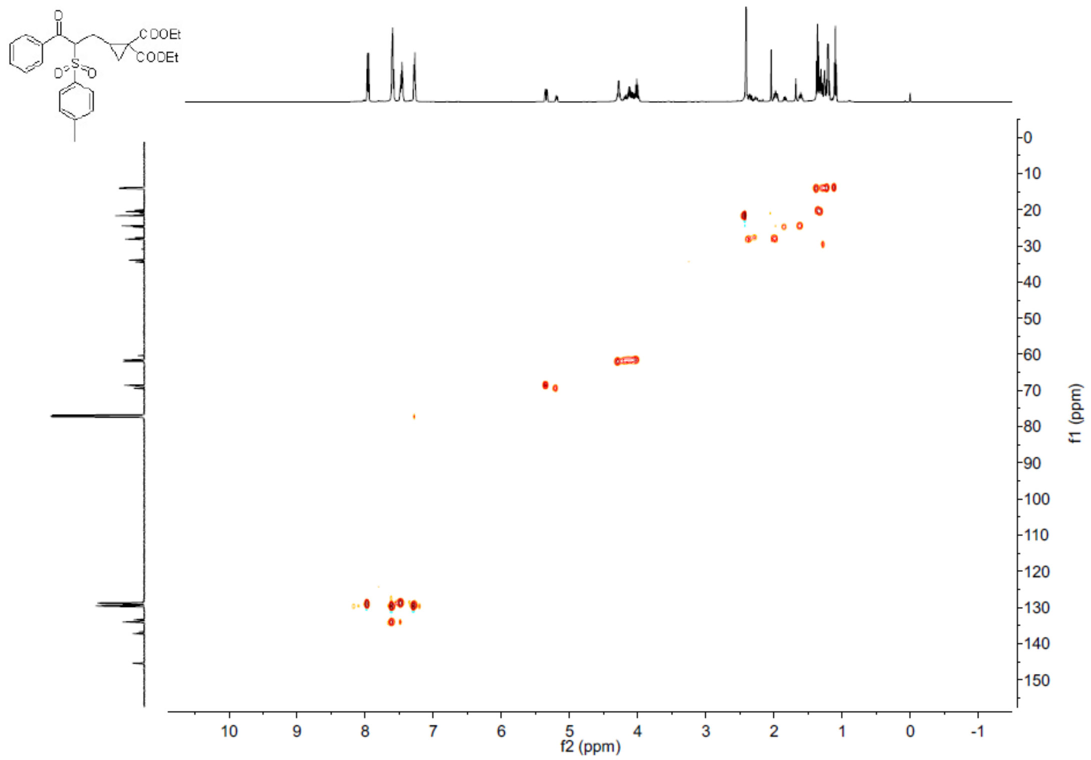

HMBC NMR ($CDCl_3$) spectrum of compound **3a**

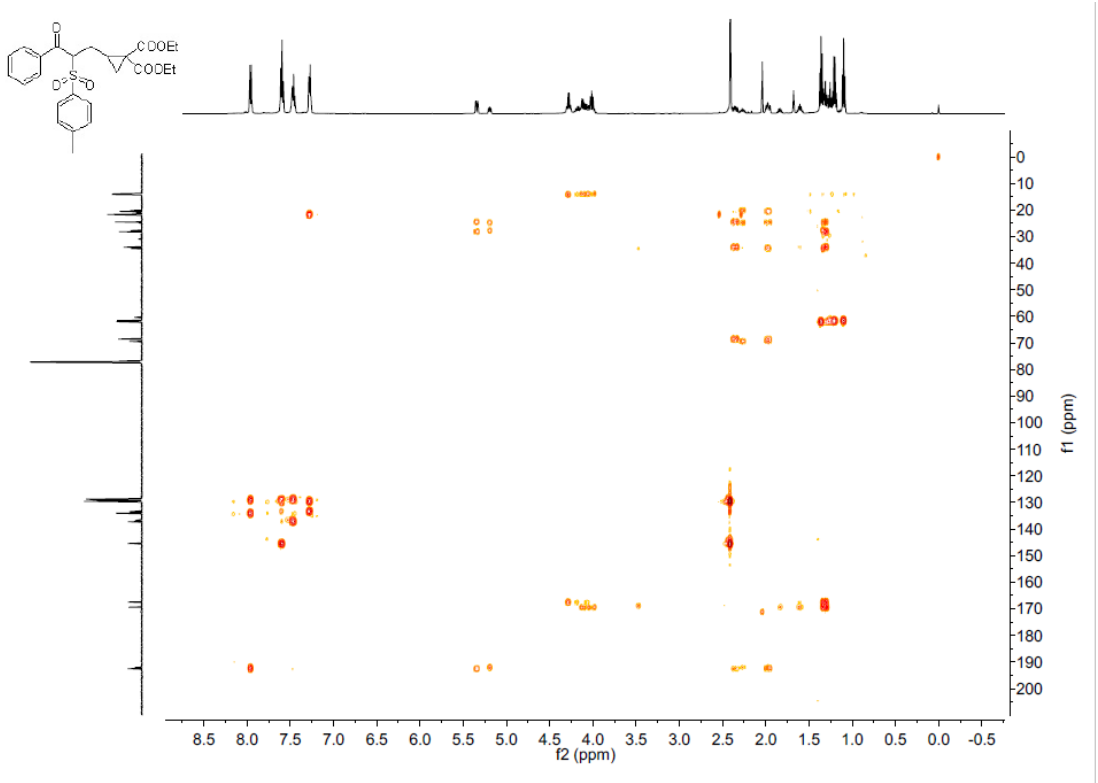


$^{1}$H NMR (500 MHz, $CDCl_3$) spectrum of compound **3a′**

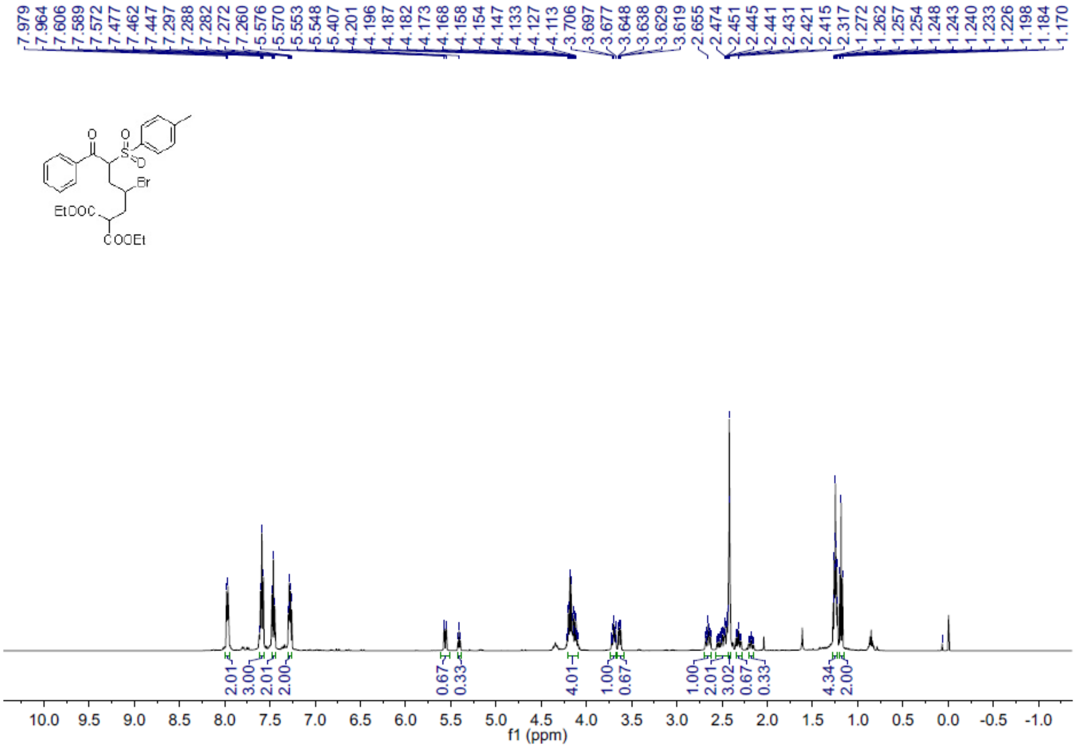

$^{13}$C NMR (126 MHz, $CDCl_3$) spectrum of compound **3a′**

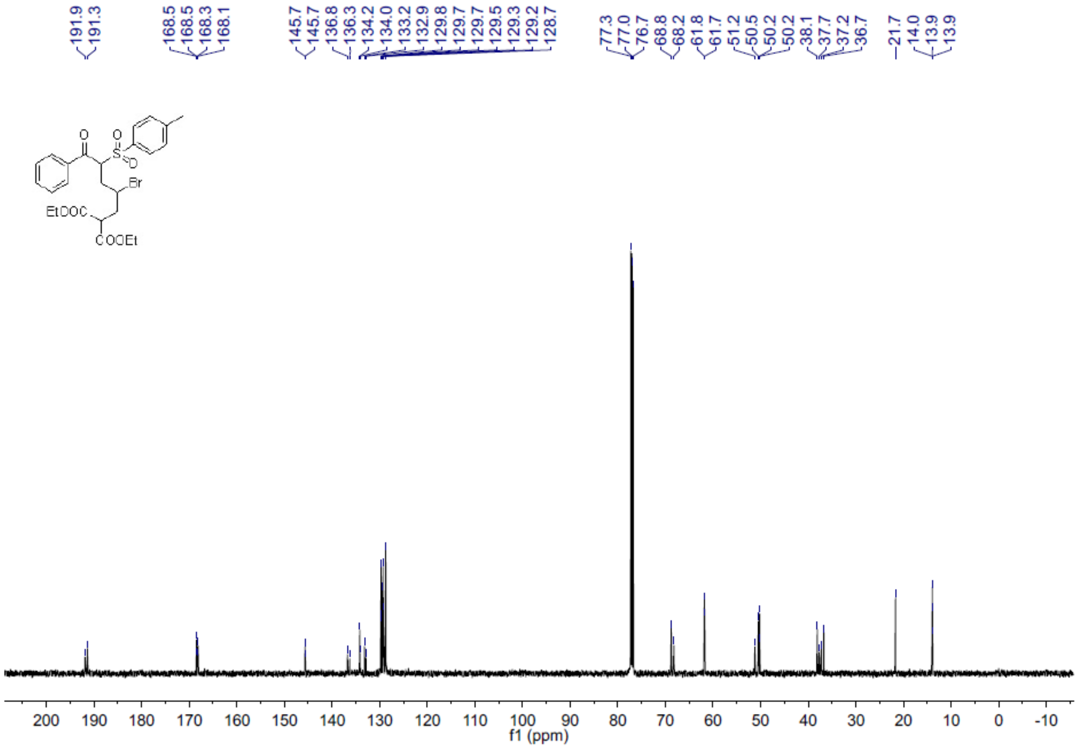


DEPT 135 NMR ($CDCl_3$) spectrum of compound **3a′**

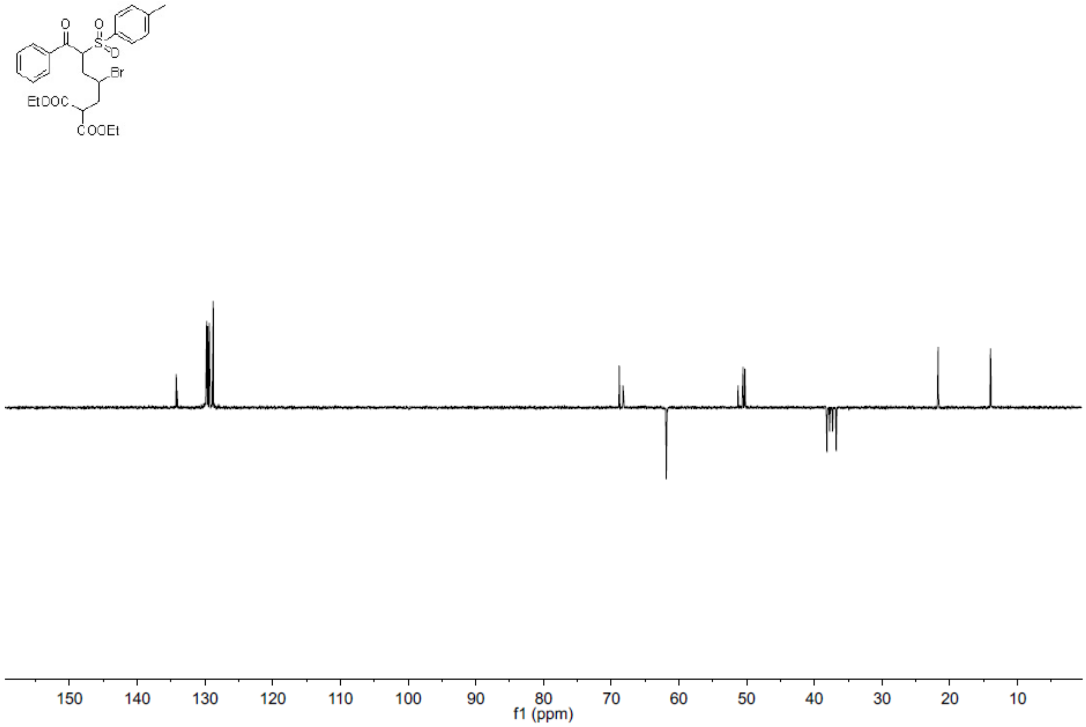

$^{1}$H NMR (500 MHz, $CDCl_3$) spectrum of compound **3b**

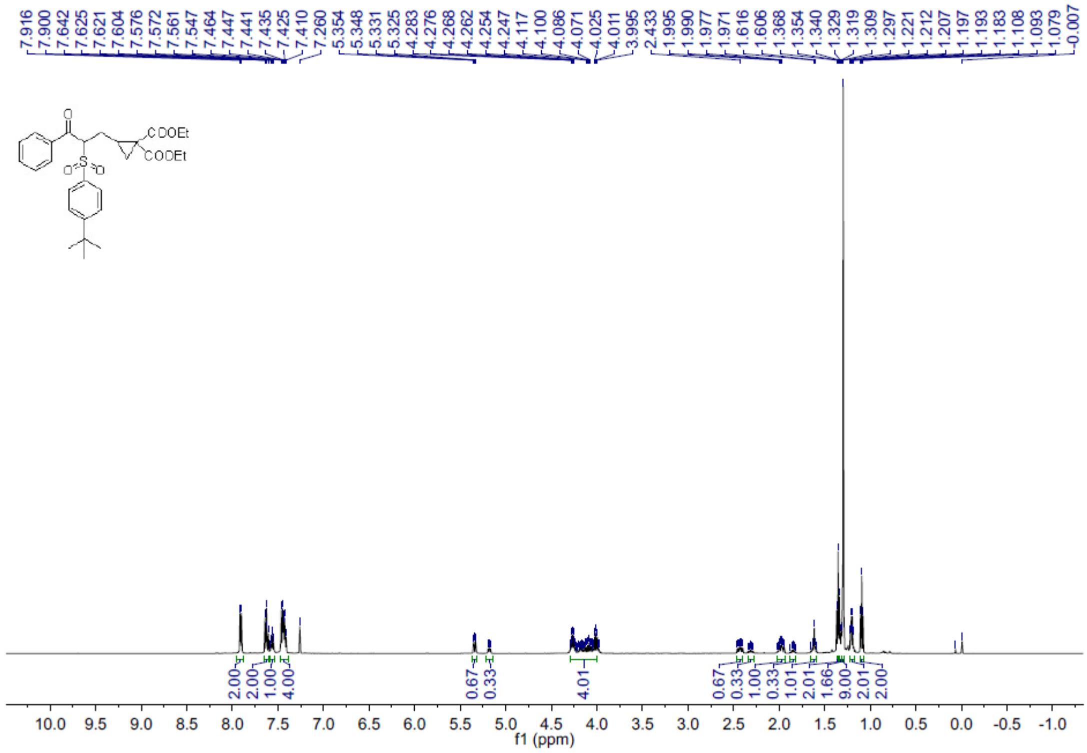


$^{13}$C {$^{1}$H} NMR (126 MHz, $CDCl_3$) spectrum of compound **3b**

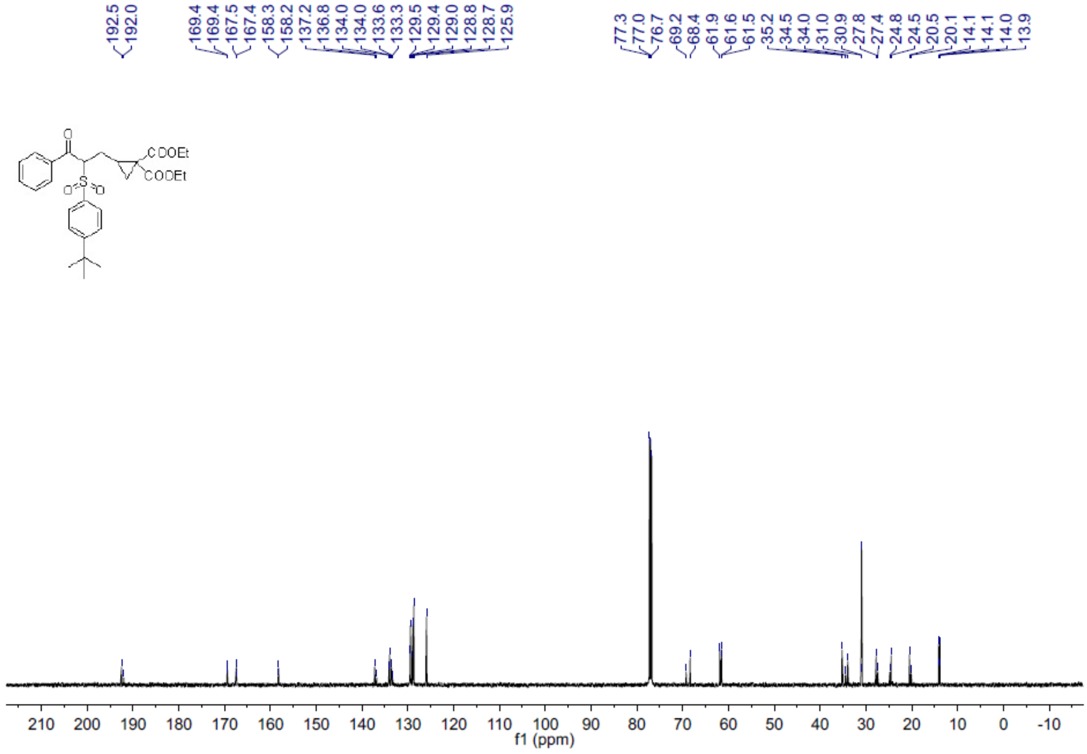

$^{1}H$ NMR (500 MHz, $CDCl_3$) spectrum of compound **3c**

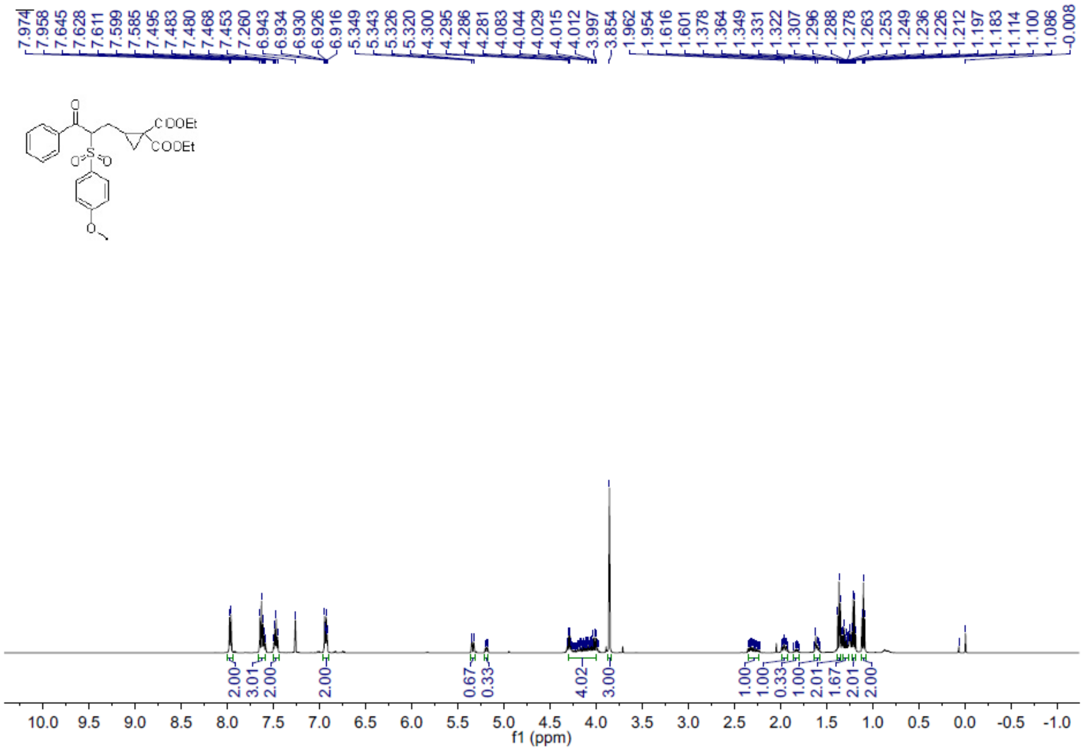

$^{13}C$ {$^{1}H$} NMR (126 MHz, $CDCl_3$) spectrum of compound **3c**

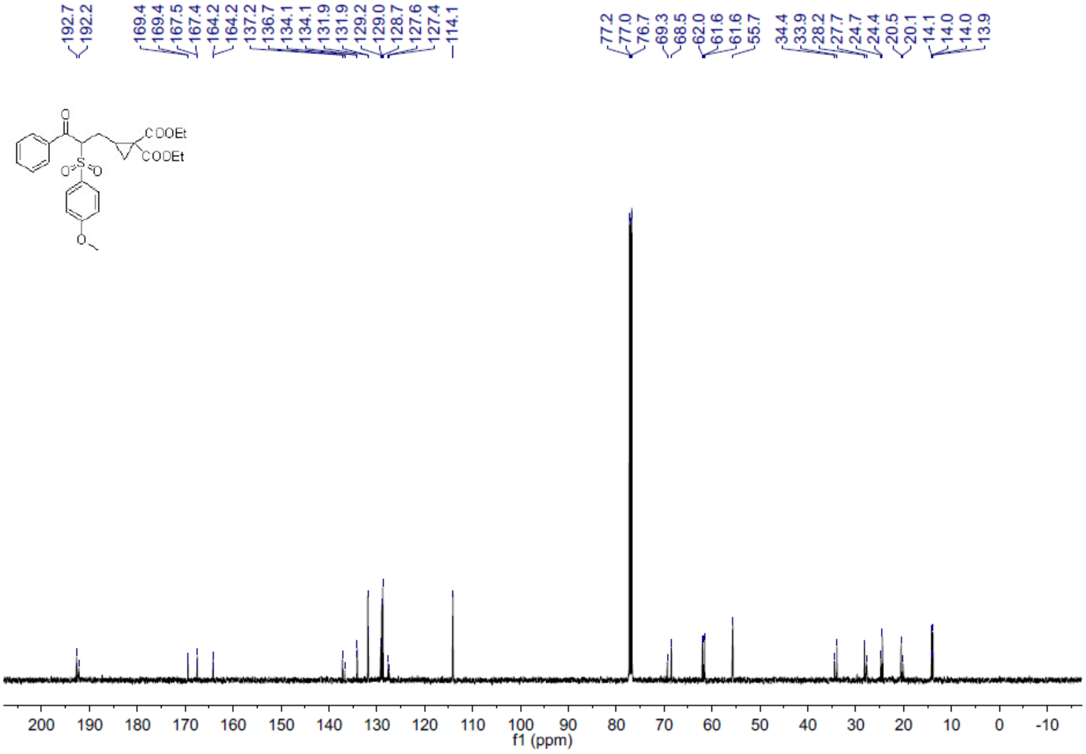

$^{1}$H NMR (500 MHz, CDCl3) spectrum of compound **3d**

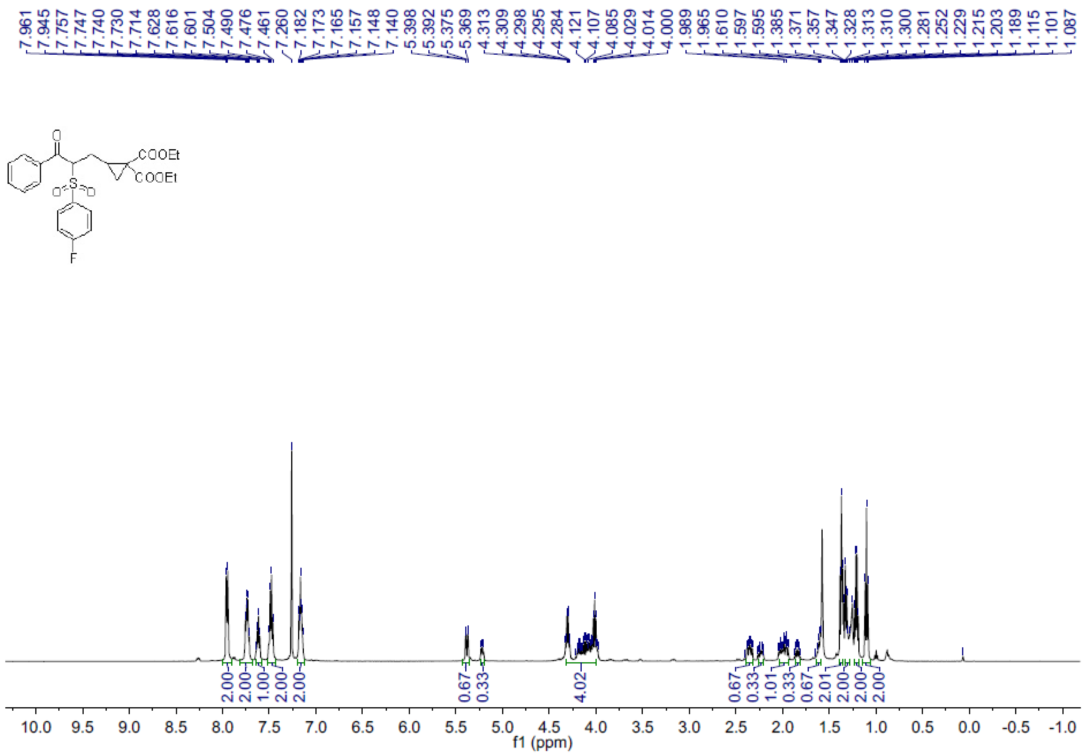


$^{13}$C {$^{1}$H} NMR (126 MHz, CDCl3) spectrum of compound **3d**

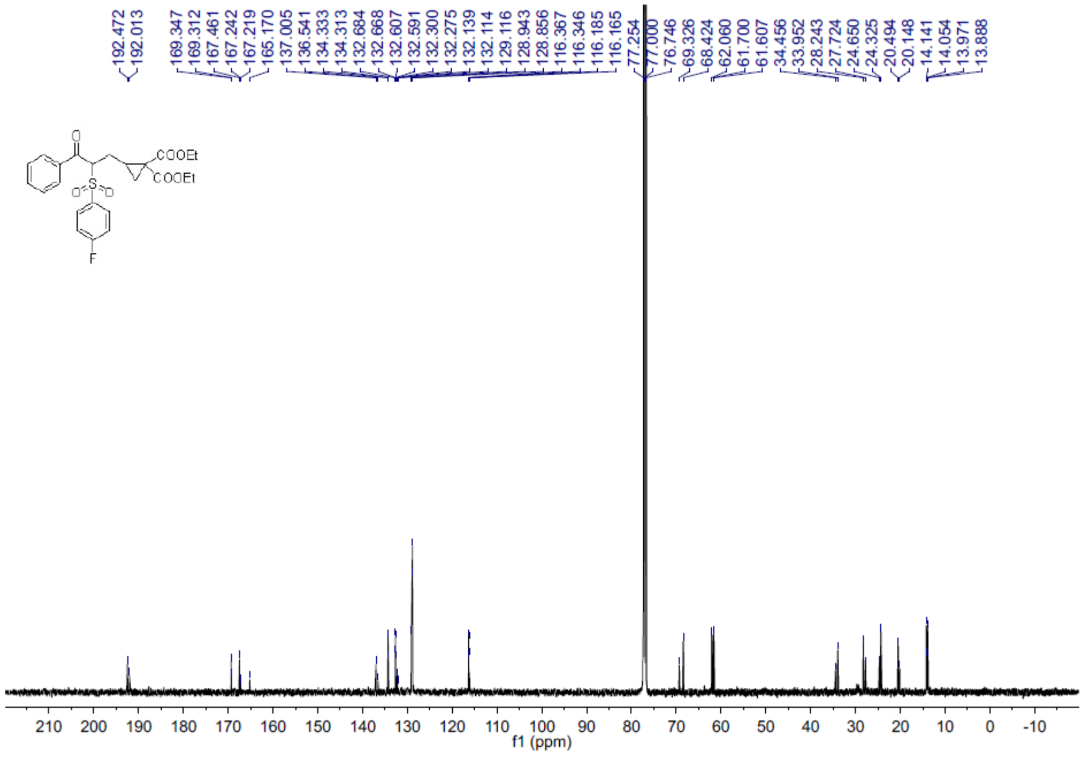

$^{19}$ F NMR (470 MHz, $CDCl_3$) spectrum of compound **3d**

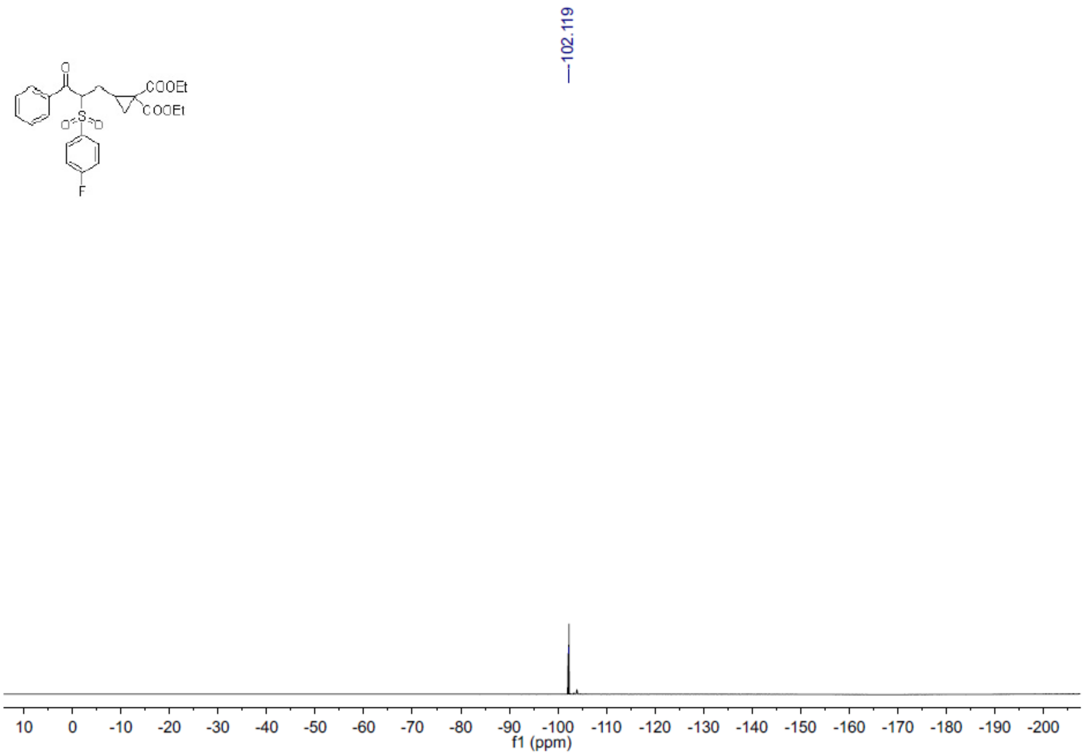


$^{1}$H NMR (500 MHz, $CDCl_3$) spectrum of compound **3e**

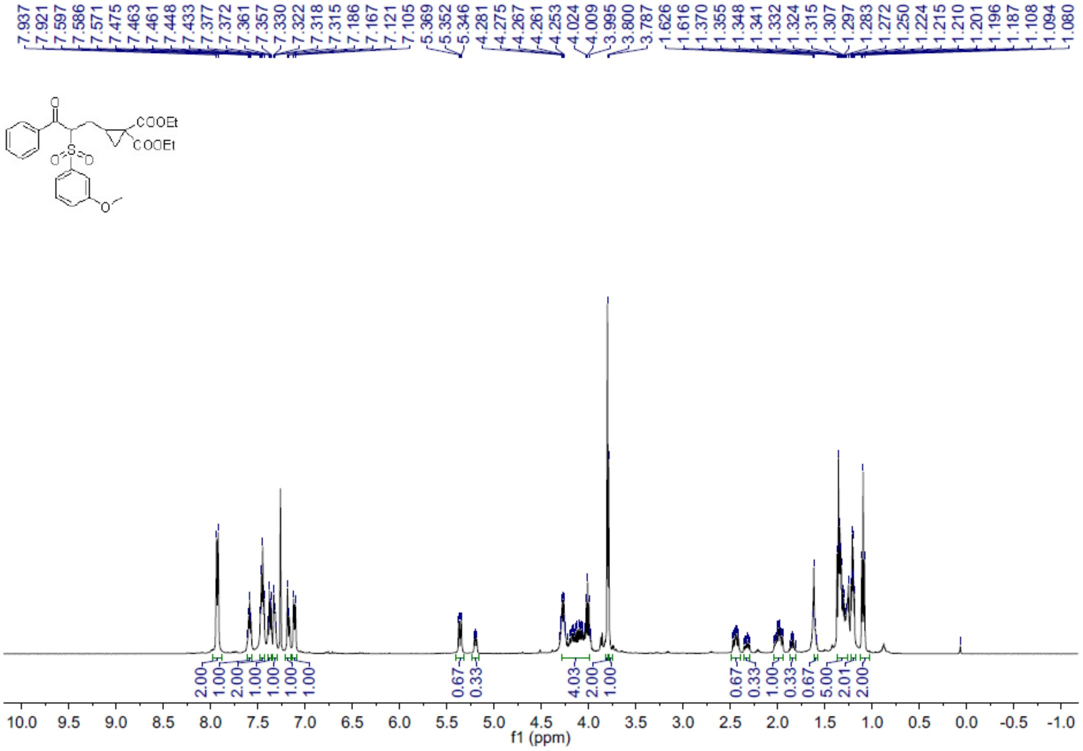

$^{13}C$ {$^{1}H$} NMR (126 MHz, $CDCl_3$) spectrum of compound **3e**

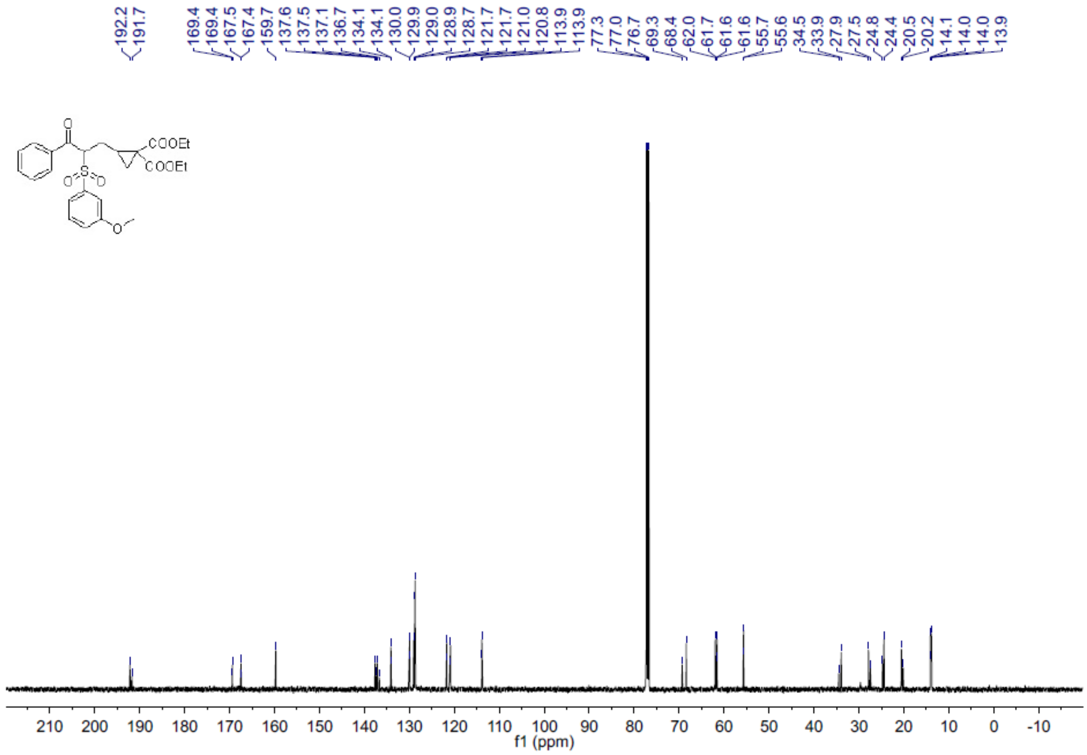


$^{1}H$ NMR (500 MHz, $CDCl_3$) spectrum of compound **3f**

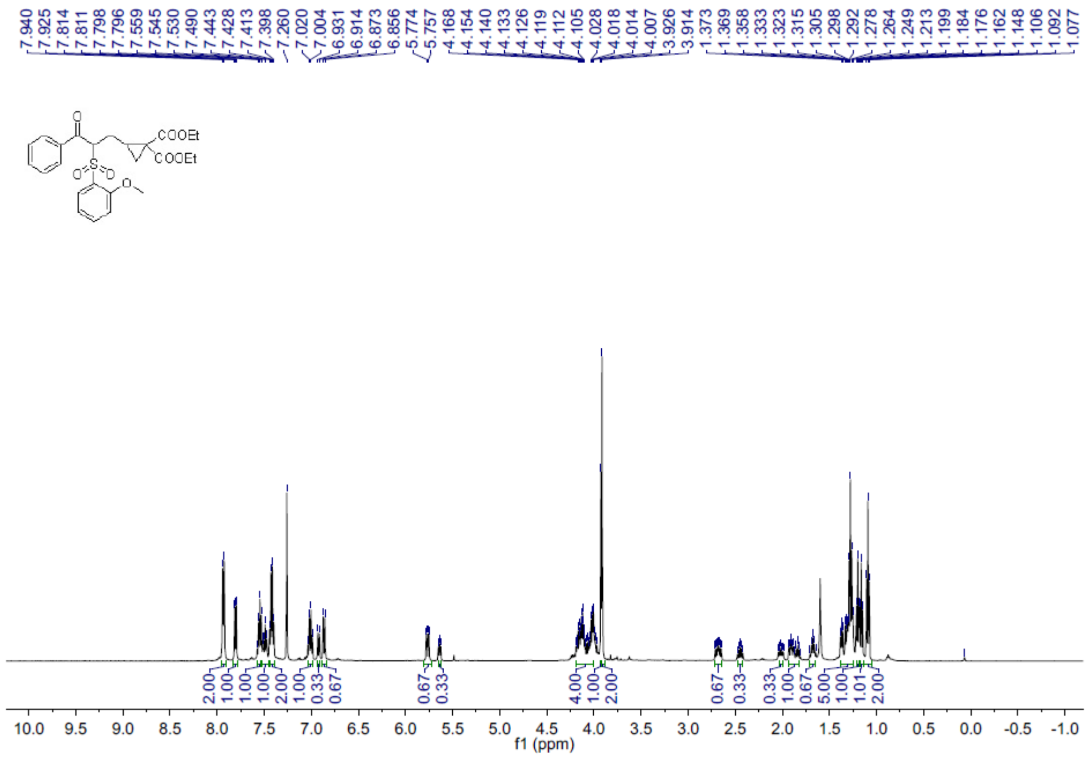

$^{13}C$ {$^{1}H$} NMR (126 MHz, $CDCl_3$) spectrum of compound **3f**

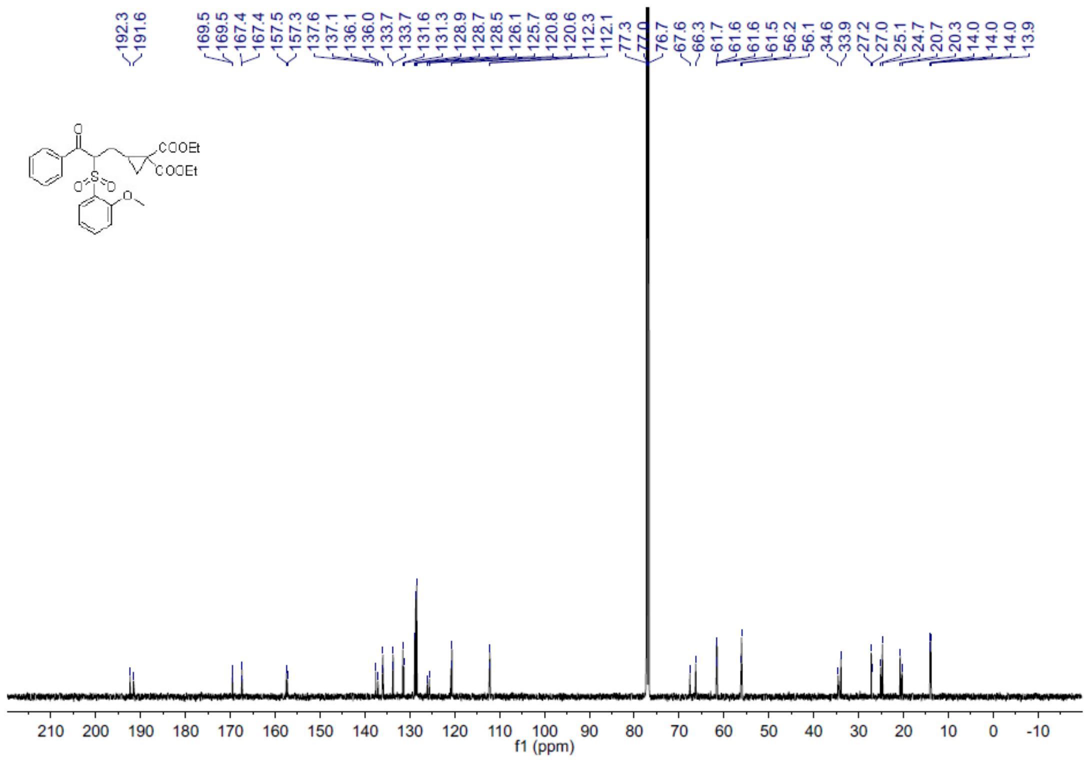

$^{1}H$ NMR (500 MHz, $CDCl_3$) spectrum of compound **3g**

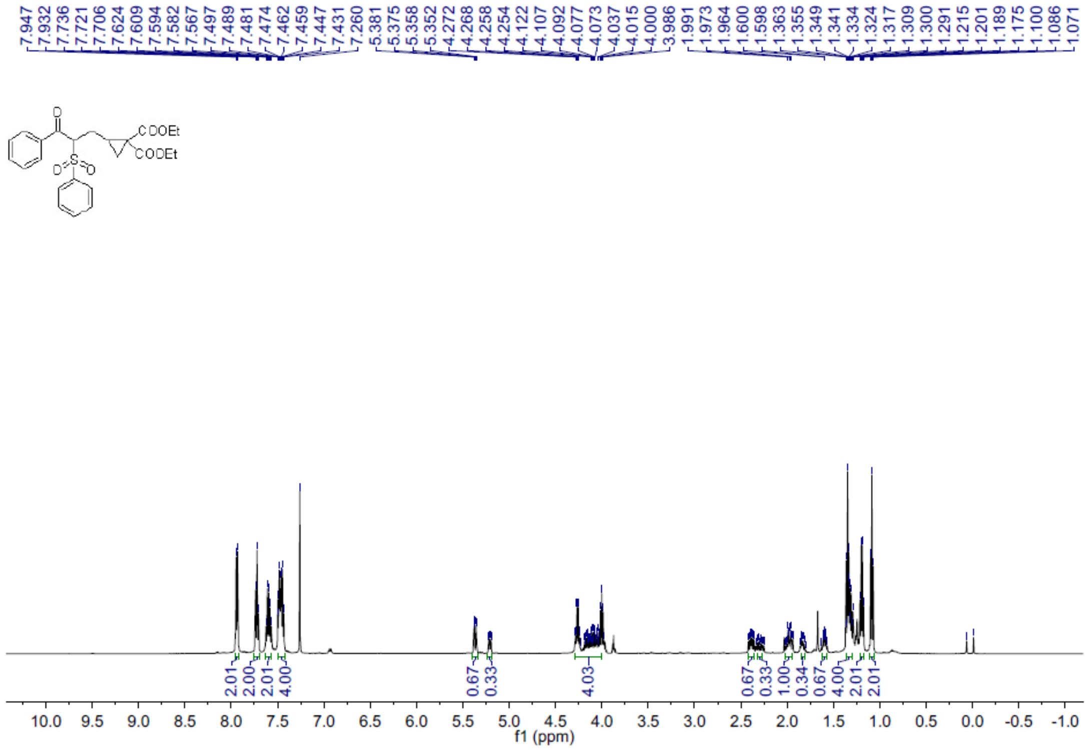

$^{13}$C {$^{1}$H} NMR (126 MHz, $CDCl_3$) spectrum of compound **3g**

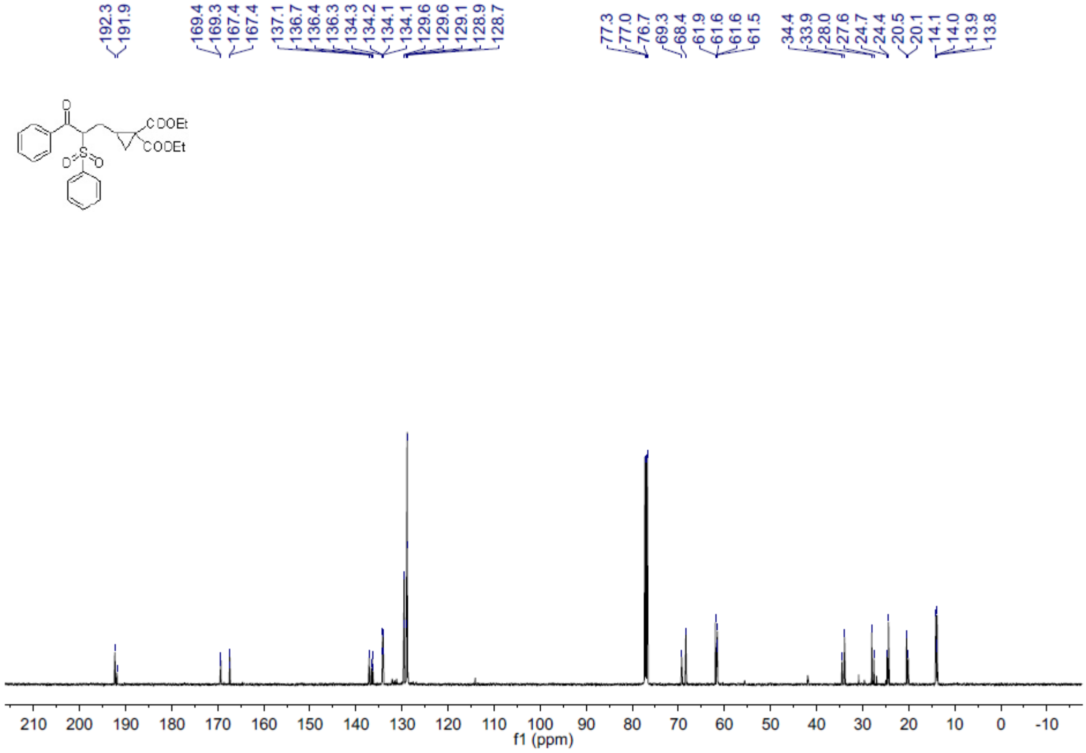


$^{1}$H NMR (500 MHz, $CDCl_3$) spectrum of compound **3h**

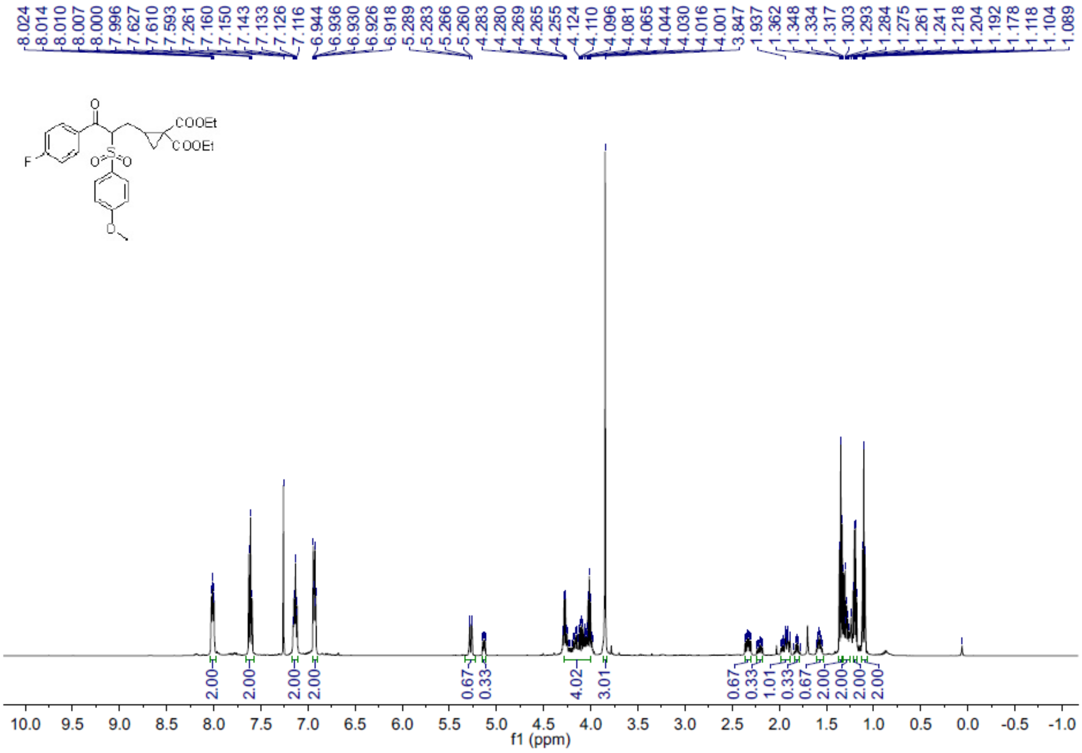

$^{13}C$ {$^{1}H$} NMR (126 MHz, $CDCl_3$) spectrum of compound **3h**

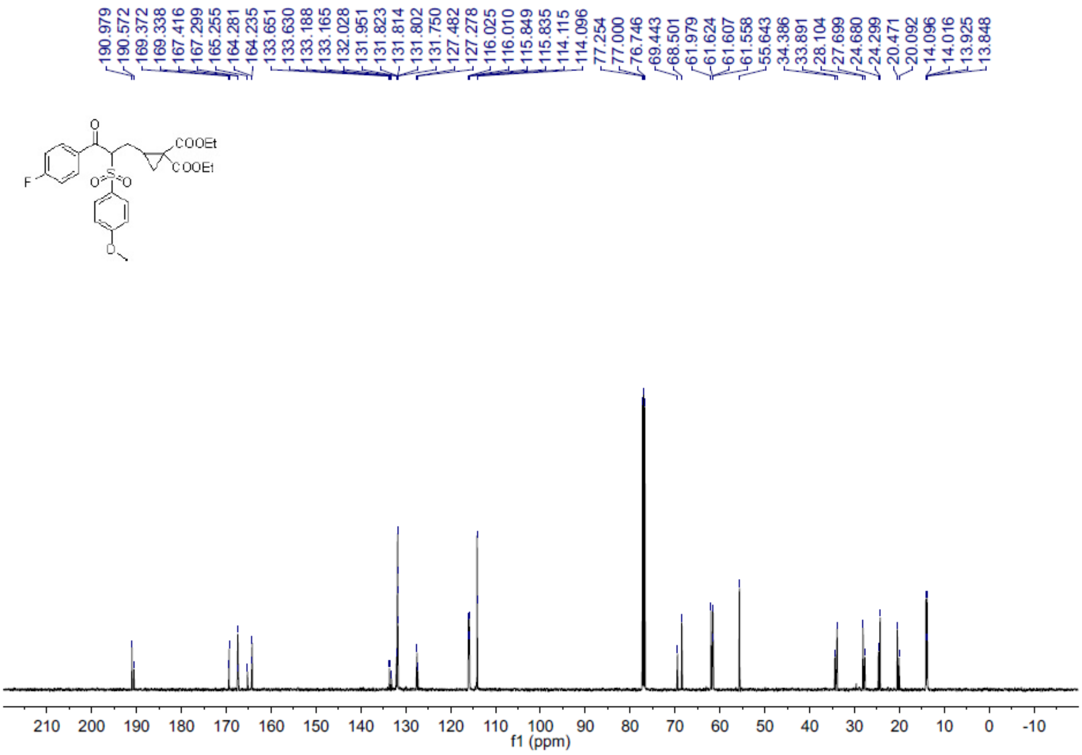


$^{19}F$ NMR (470 MHz, $CDCl_3$) spectrum of compound **3h**

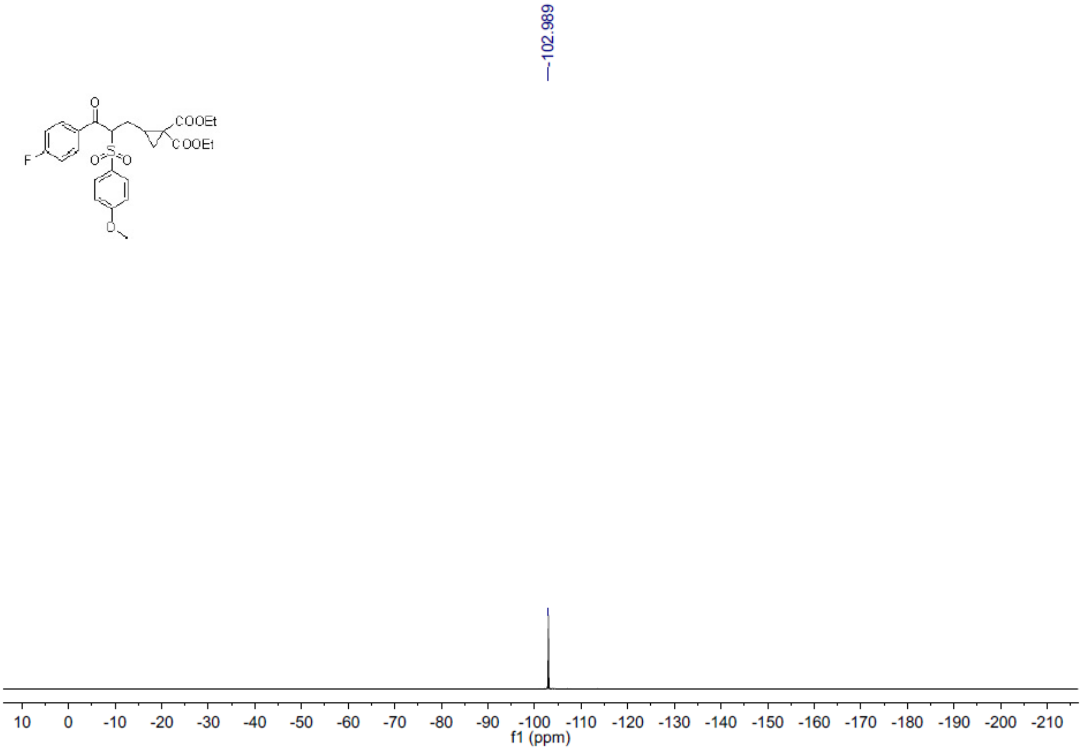

$^{1}$H NMR (500 MHz, $CDCl_3$) spectrum of compound **3i**

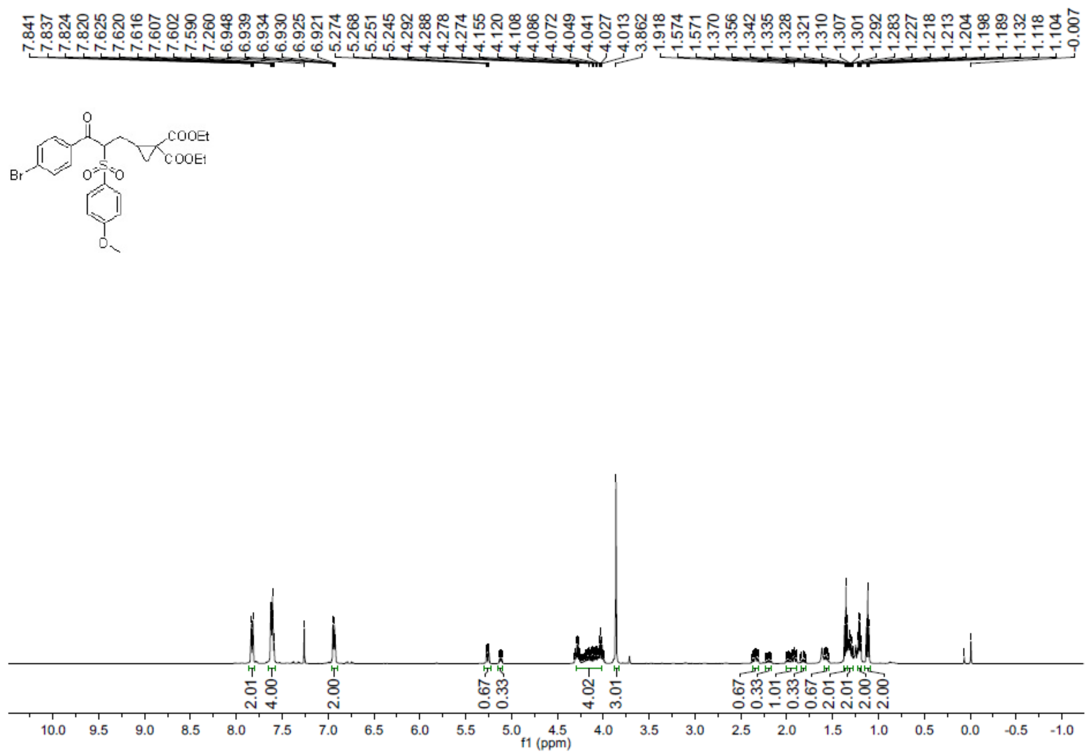

$^{13}$C {$^{1}$H} NMR (126 MHz, $CDCl_3$) spectrum of compound **3i**

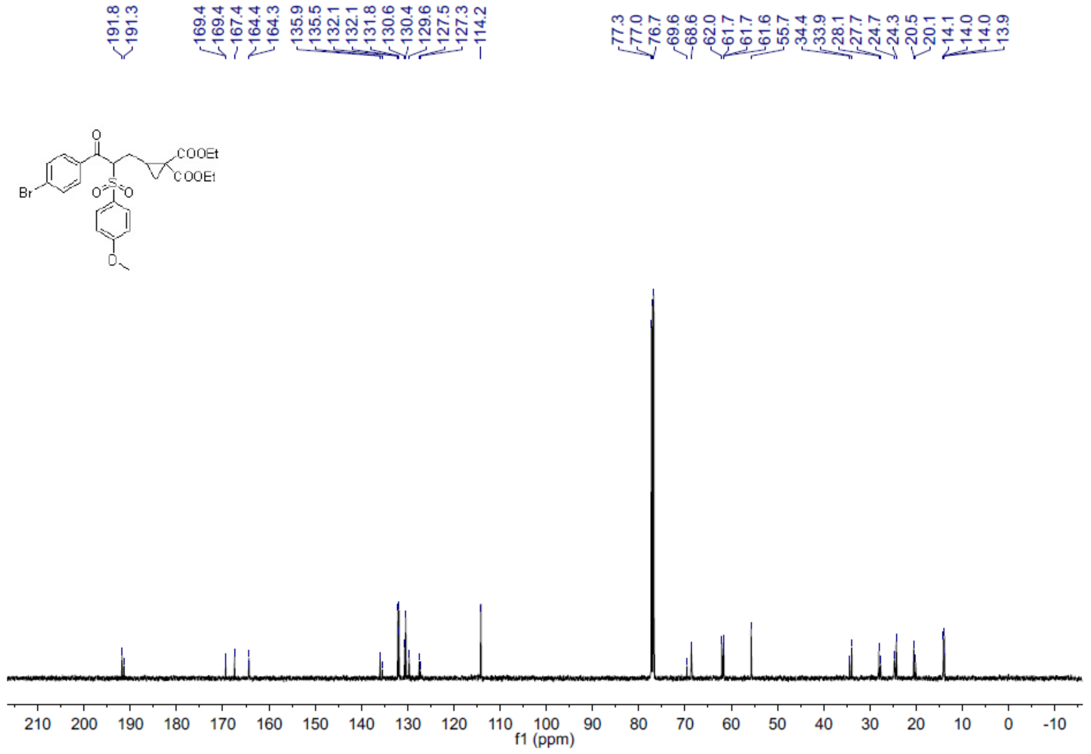

$^{1}$H NMR (500 MHz, $CDCl_3$) spectrum of compound **3j**

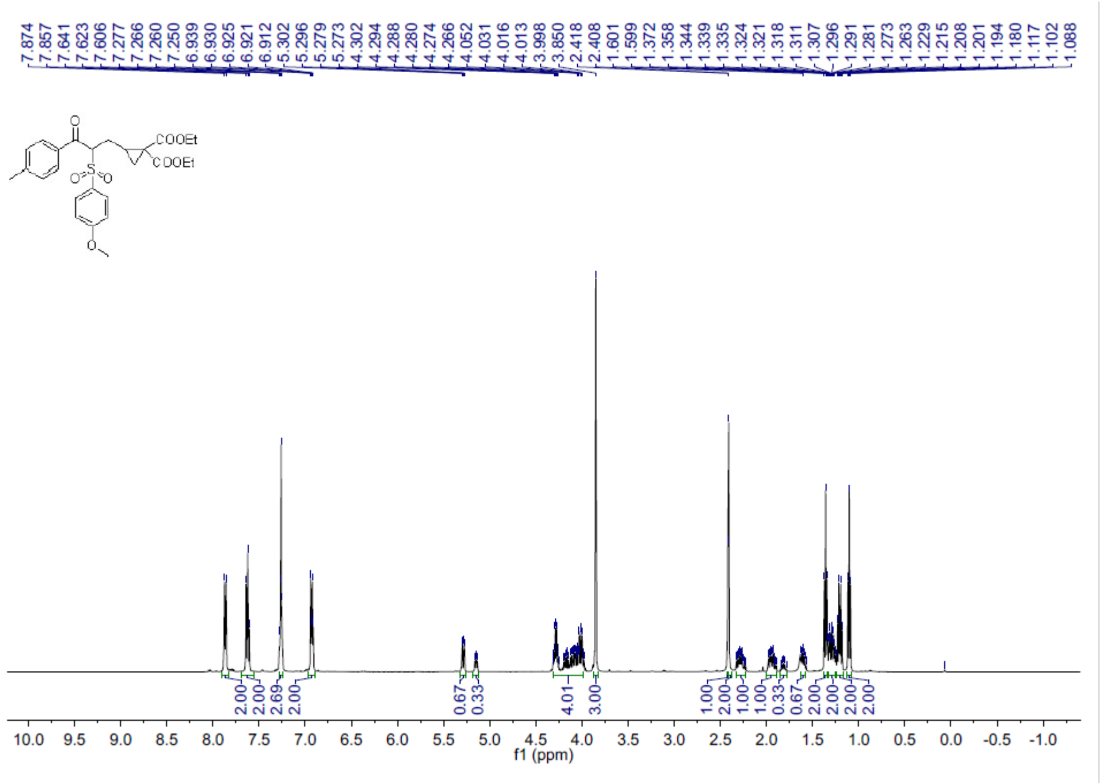


$^{13}$C {$^{1}$H} NMR (126 MHz, $CDCl_3$) spectrum of compound **3j**

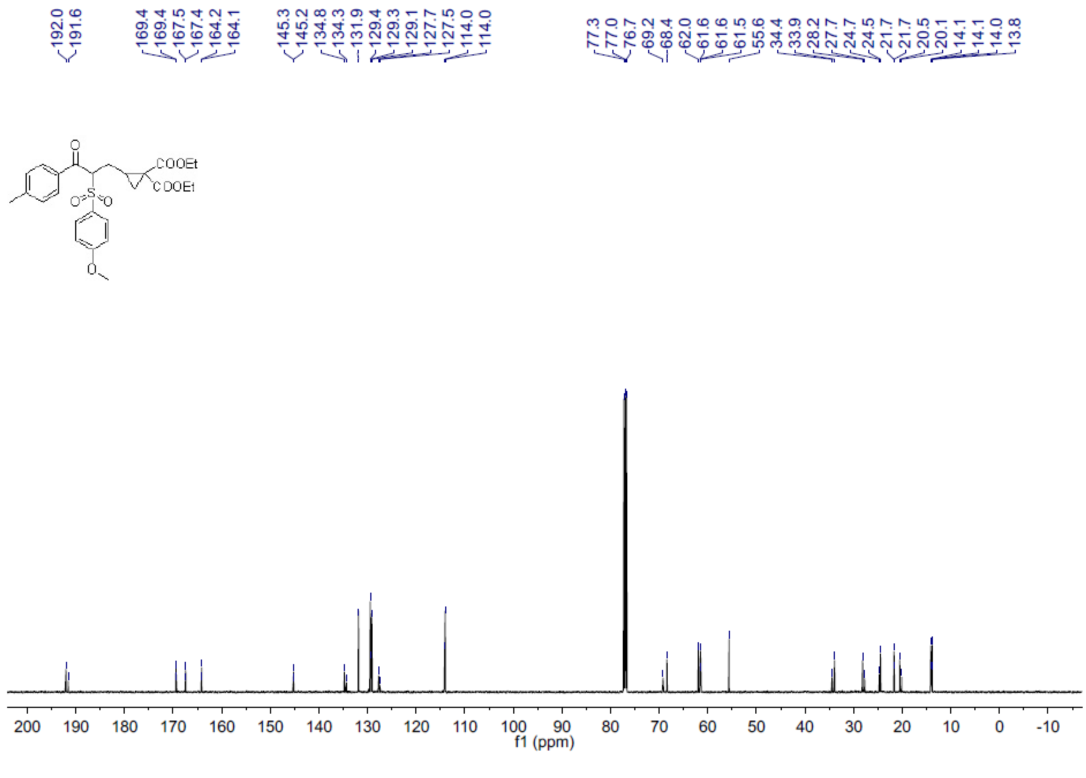

$^{1}$H NMR (500 MHz, $CDCl_3$) spectrum of compound **3k**

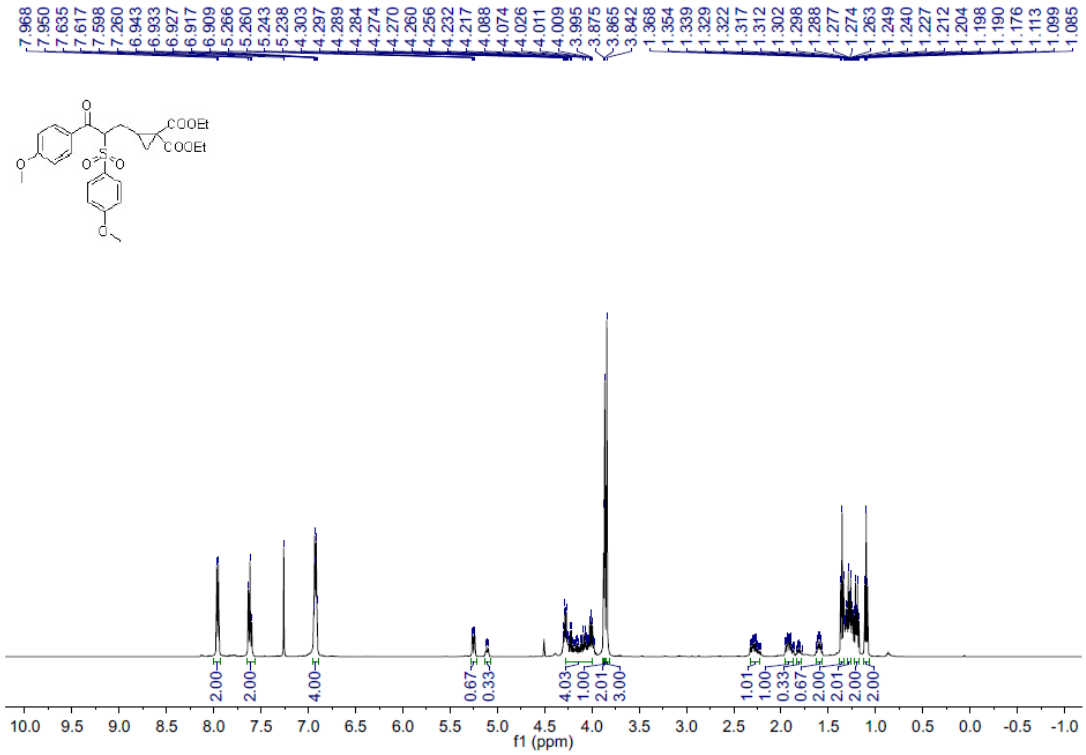


$^{13}$C {$^{1}$H} NMR (126 MHz, $CDCl_3$) spectrum of compound **3k**

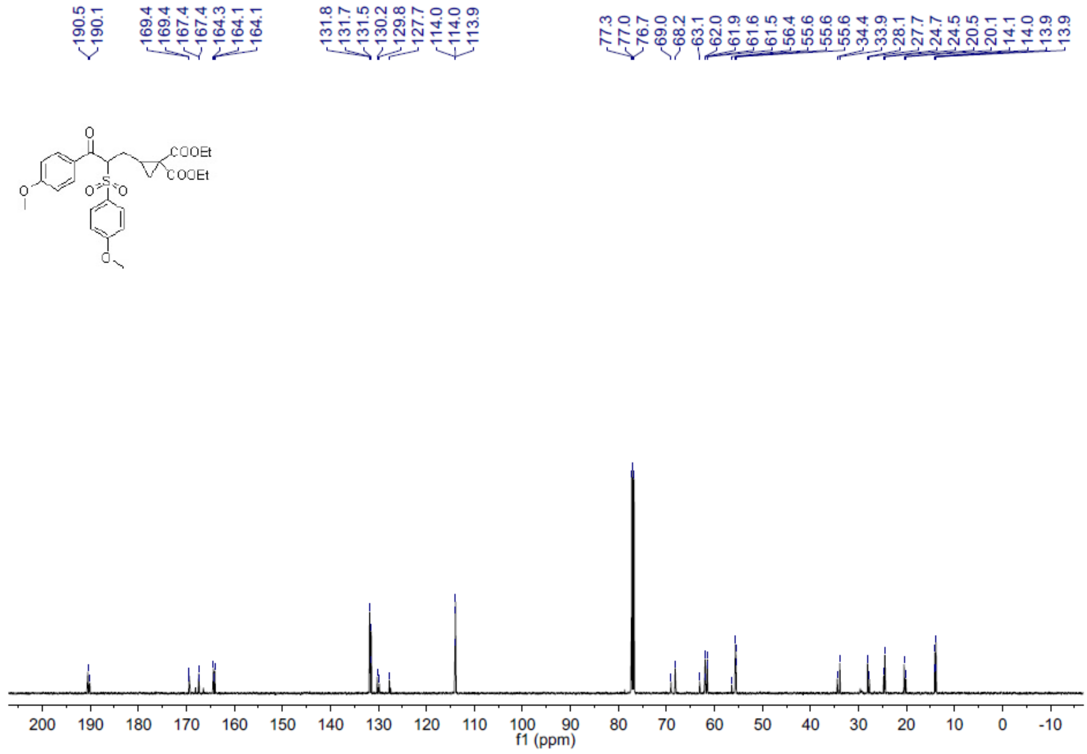

$^{1}$H NMR (500 MHz, $CDCl_3$) spectrum of compound **3l**

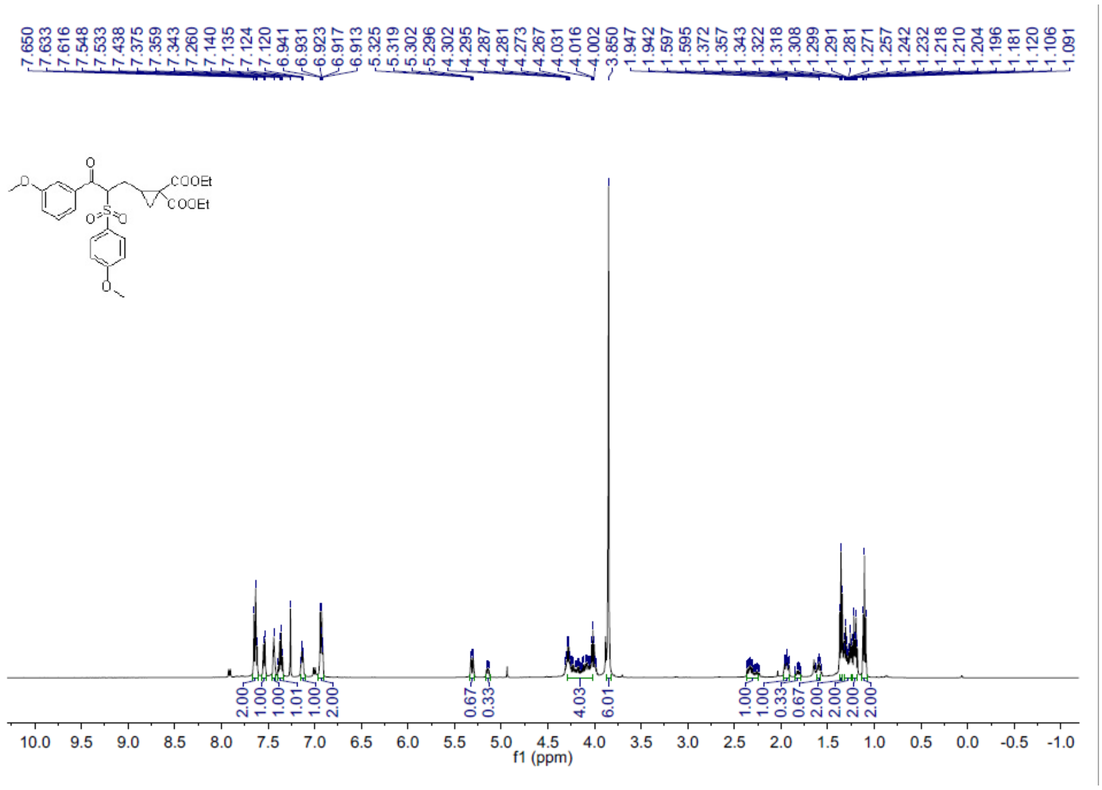


$^{13}$C {$^{1}$H} NMR (126 MHz, $CDCl_3$) spectrum of compound **3l**

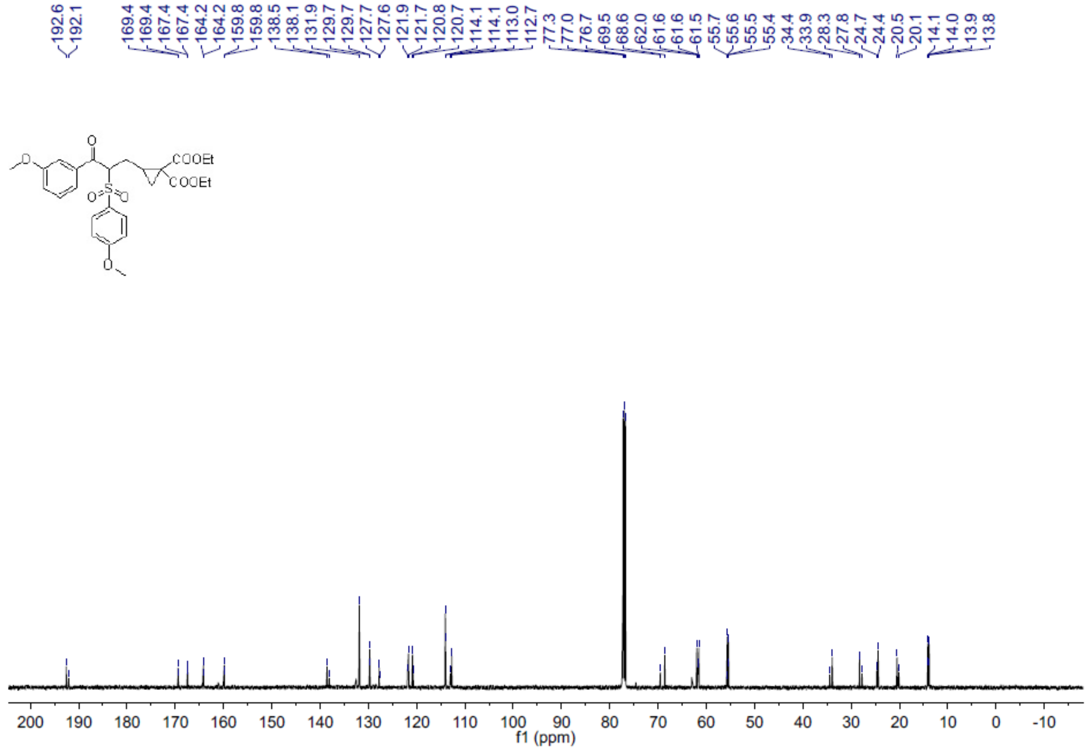

$^{1}$H NMR (500 MHz, $CDCl_3$) spectrum of compound **3m**

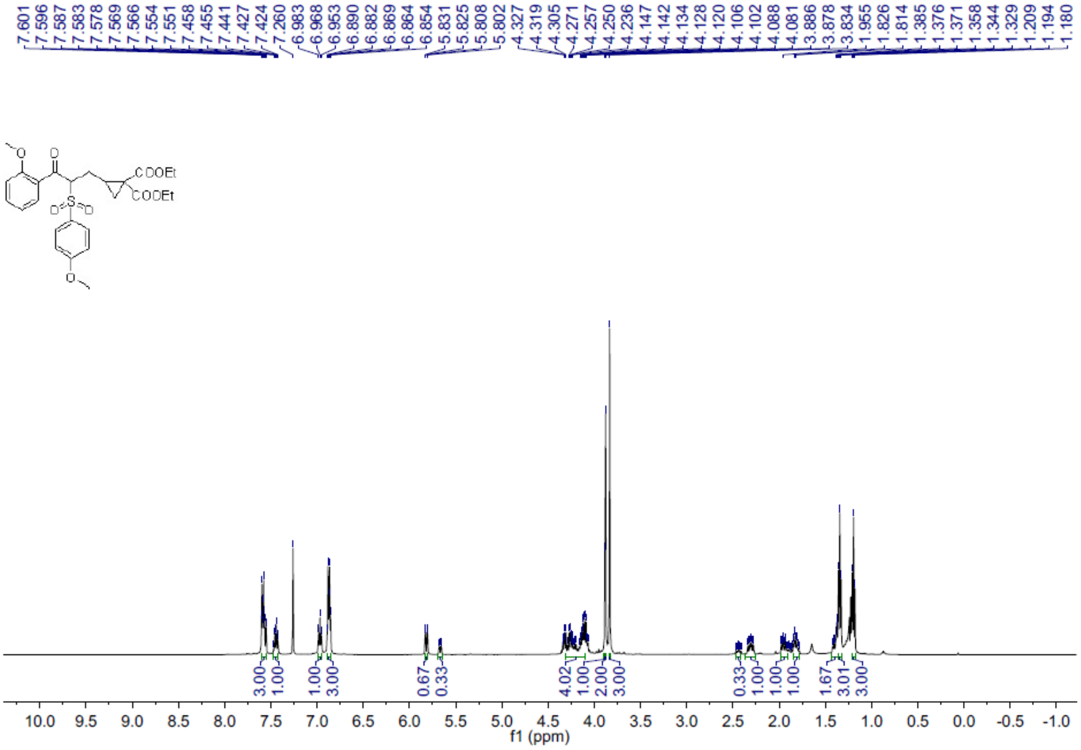


$^{13}$C {$^{1}$H} NMR (126 MHz, $CDCl_3$) spectrum of compound **3m**

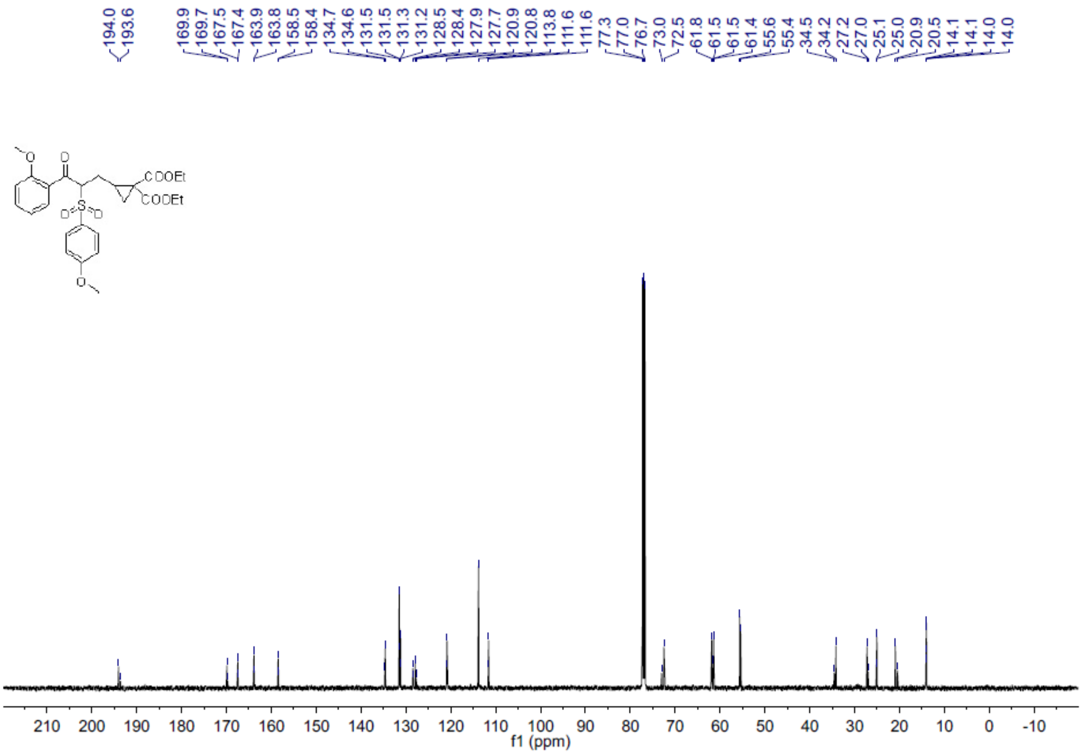

$^{1}H$ NMR (500 MHz, $CDCl_3$) spectrum of compound **4a**

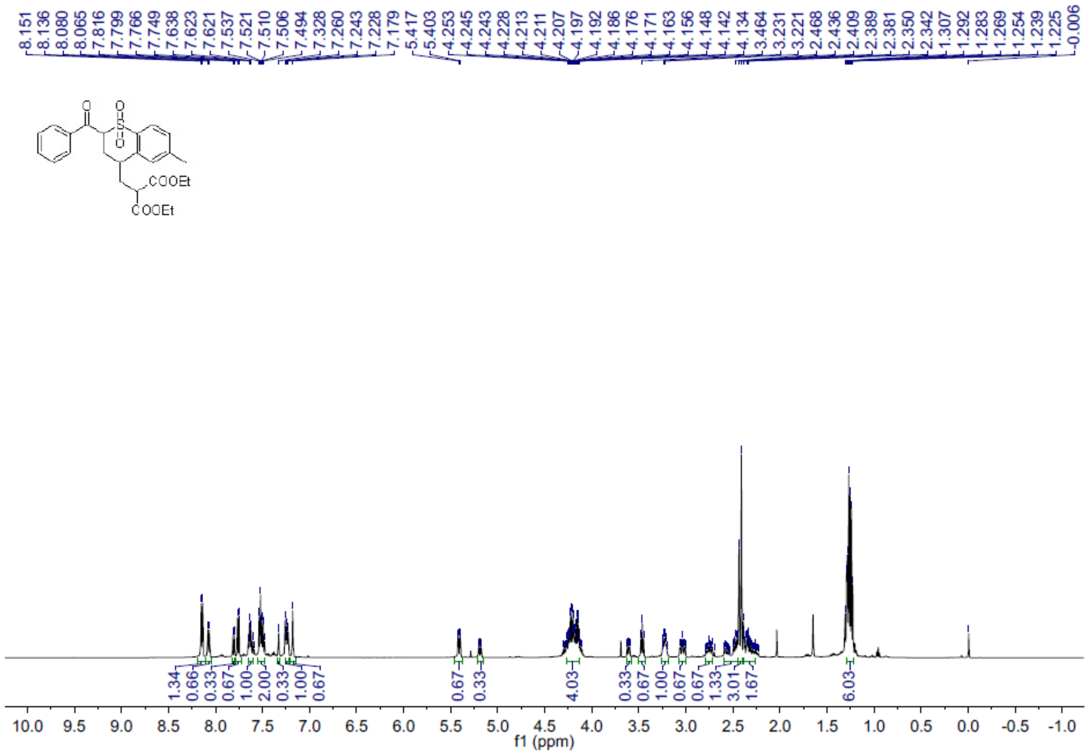


$^{13}C$ {$^{1}H$} NMR (126 MHz, $CDCl_3$) spectrum of compound **4a**

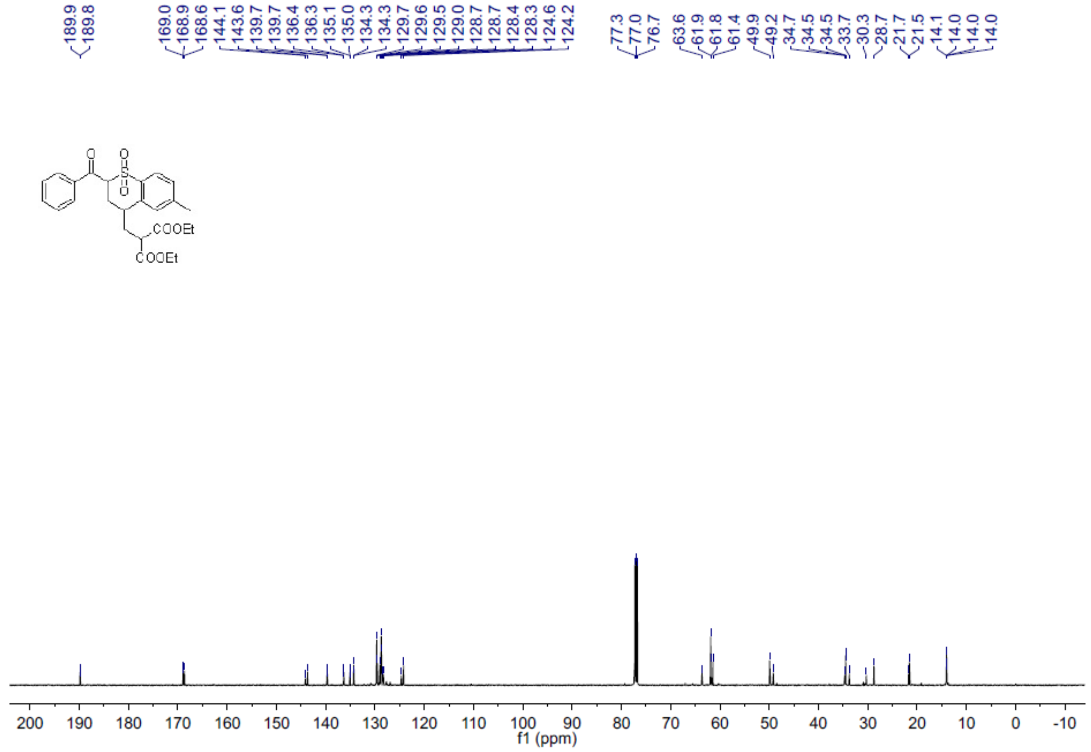

DEPT 135 NMR ($CDCl_3$) spectrum of compound **4a**

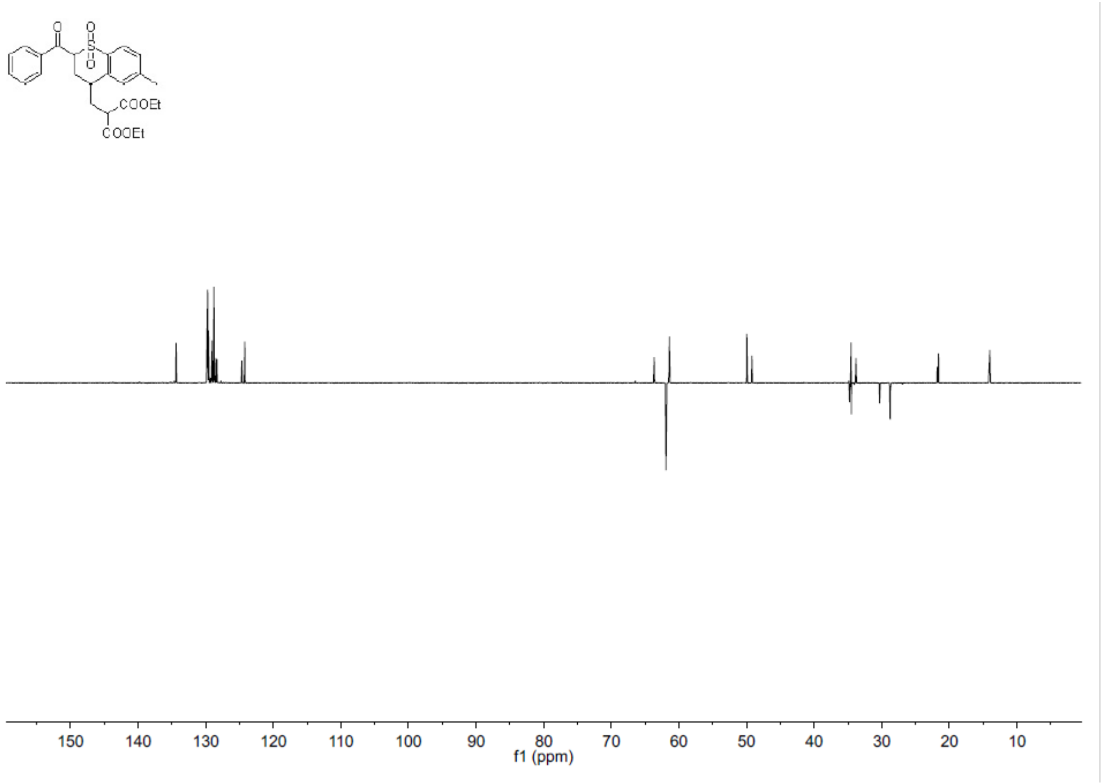


$^{1}$H-$^{1}$H COSY NMR ($CDCl_3$) spectrum of compound **4a**

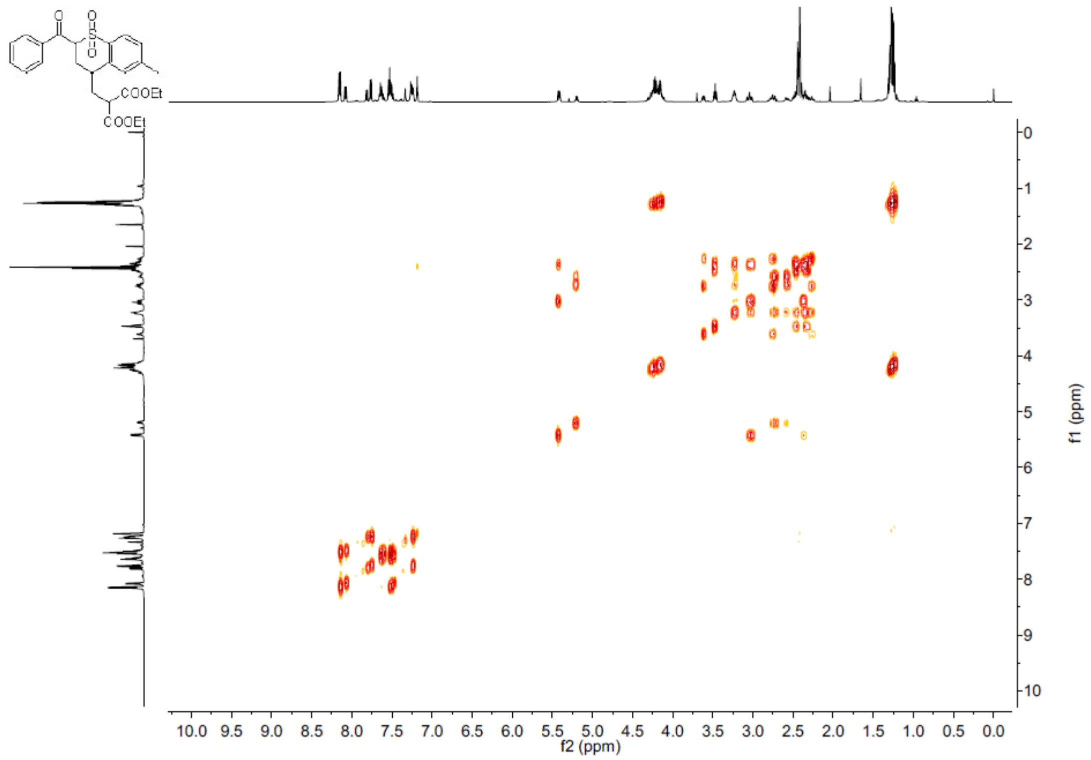

HSQC NMR ($CDCl_3$) spectrum of compound **4a**

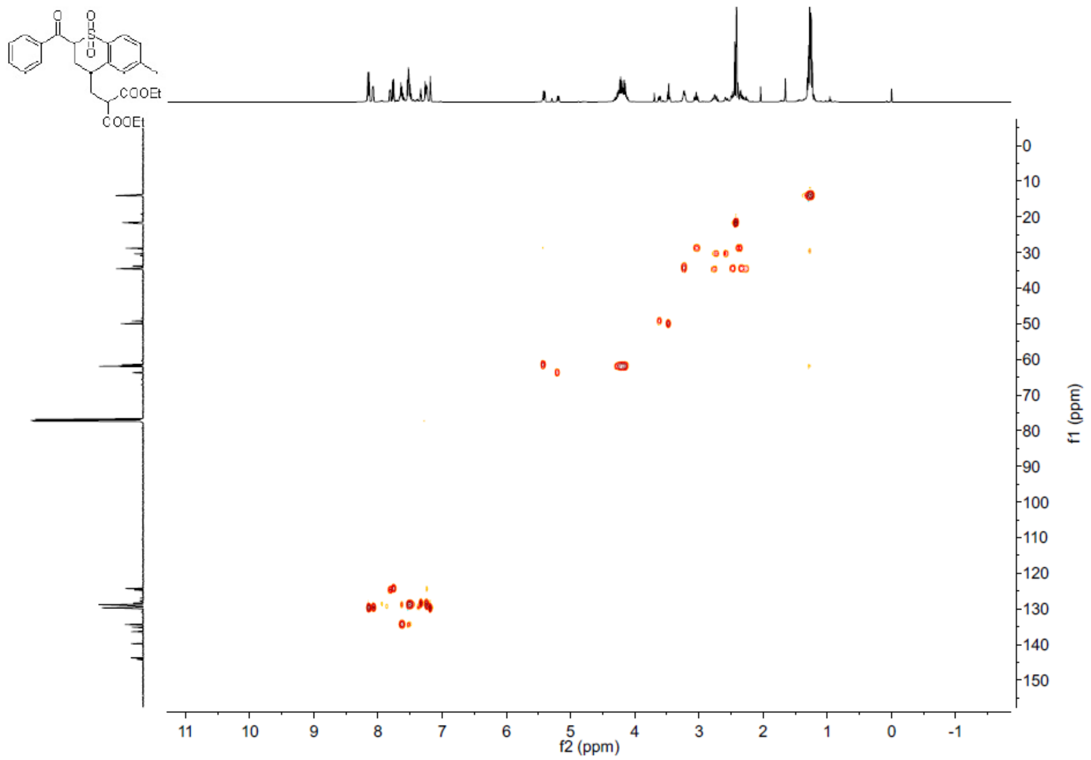


HMBC NMR ($CDCl_3$) spectrum of compound **4a**

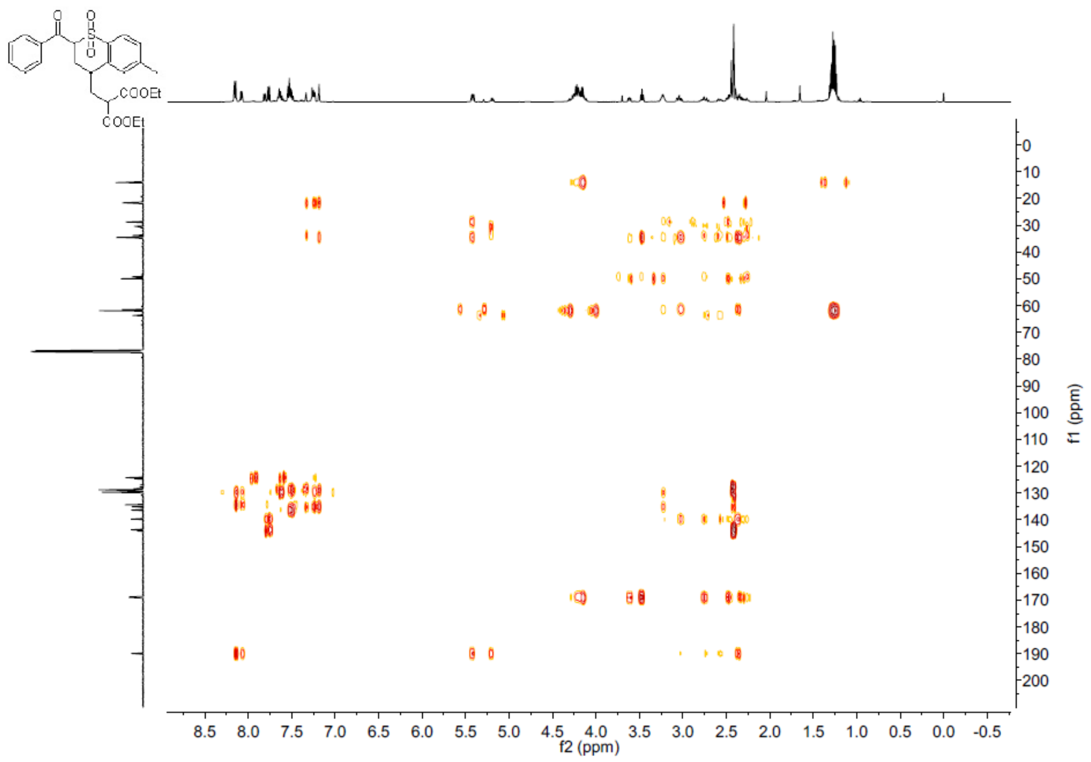

$^{1}H$ NMR (500 MHz, $CDCl_3$) spectrum of compound **4b**

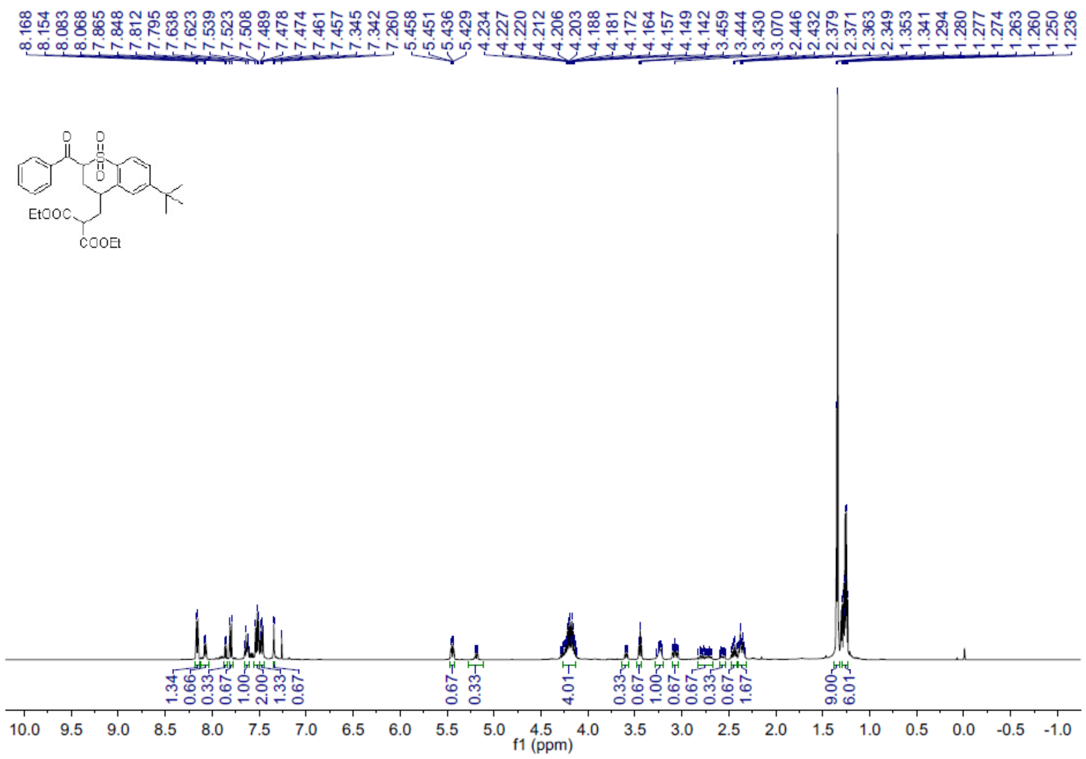

$^{13}C$ {$^{1}H$} NMR (126 MHz, $CDCl_3$) spectrum of compound **4b**

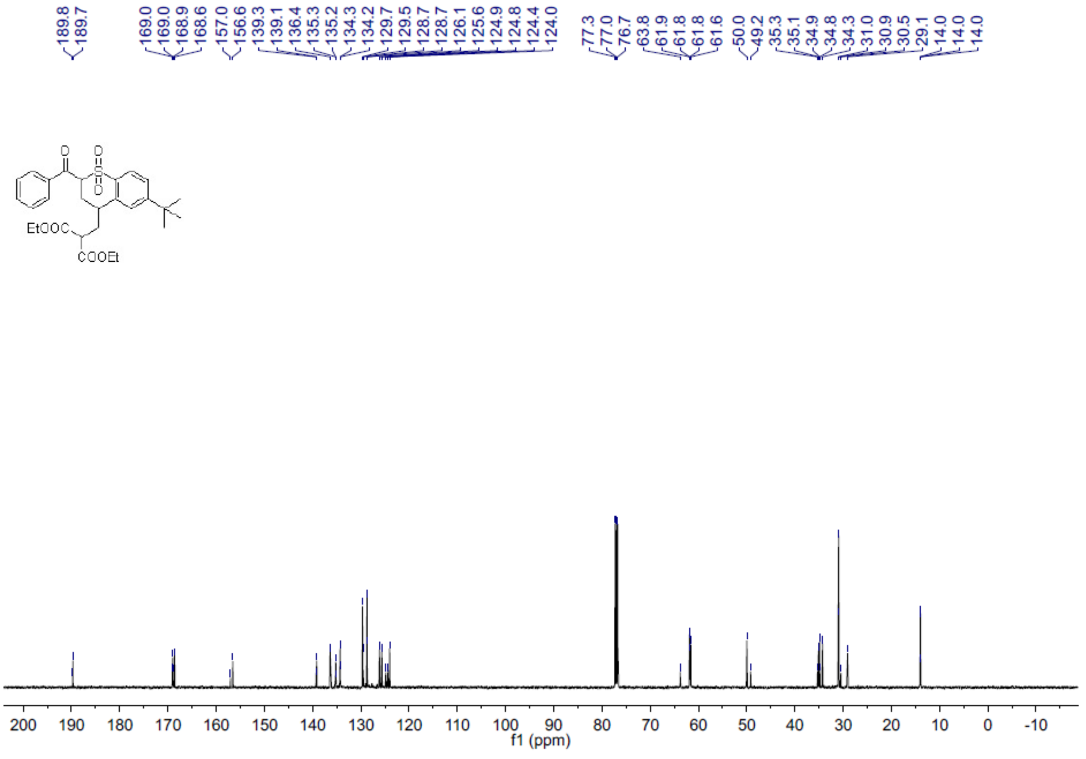

$^{1}H$ NMR (500 MHz, $CDCl_3$) spectrum of compound **4c**

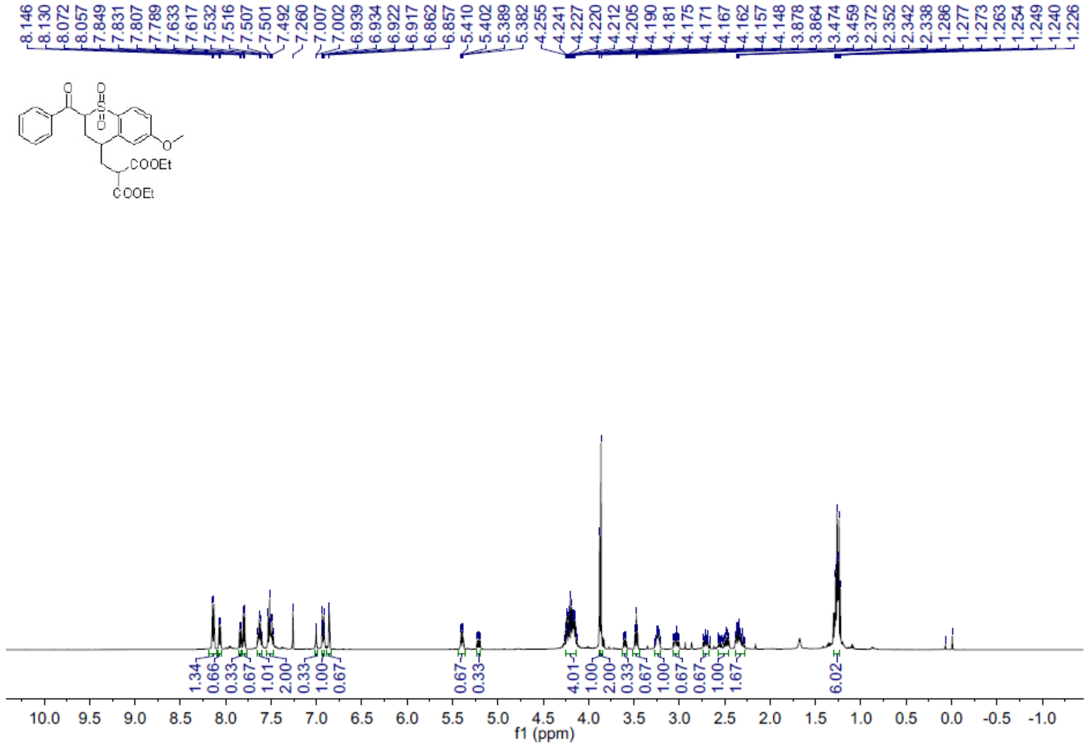


$^{13}C$ {$^{1}H$} NMR (126 MHz, $CDCl_3$) spectrum of compound **4c**

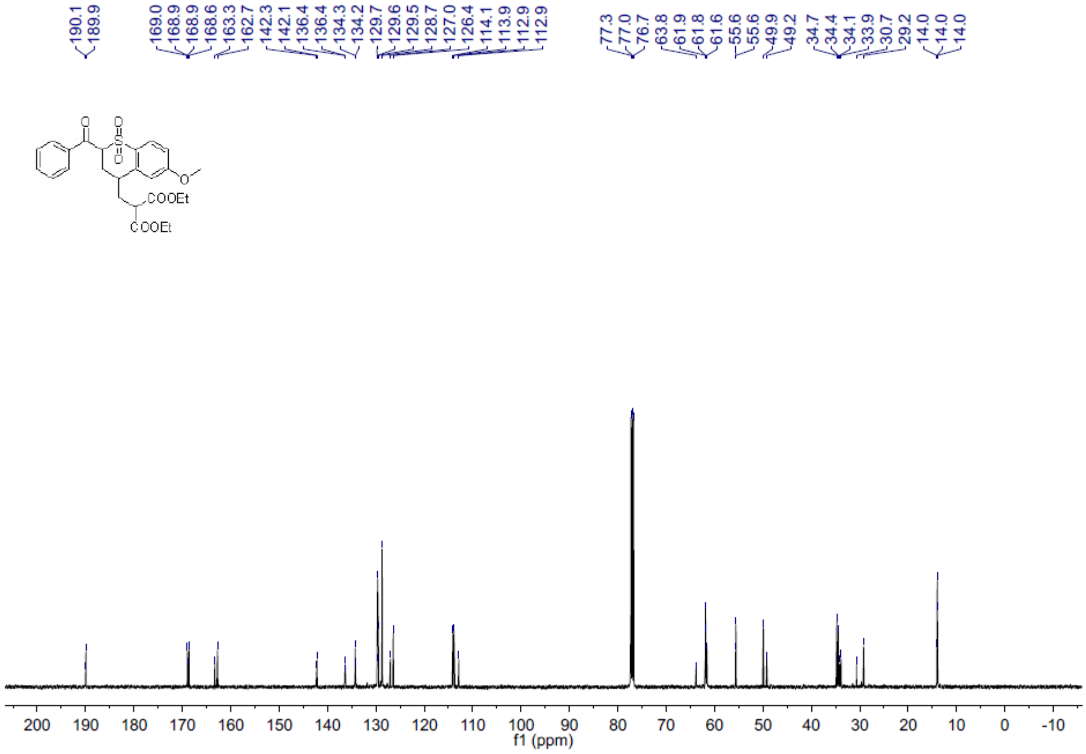

$^{1}$H NMR (500 MHz, $CDCl_3$) spectrum of compound **4d**

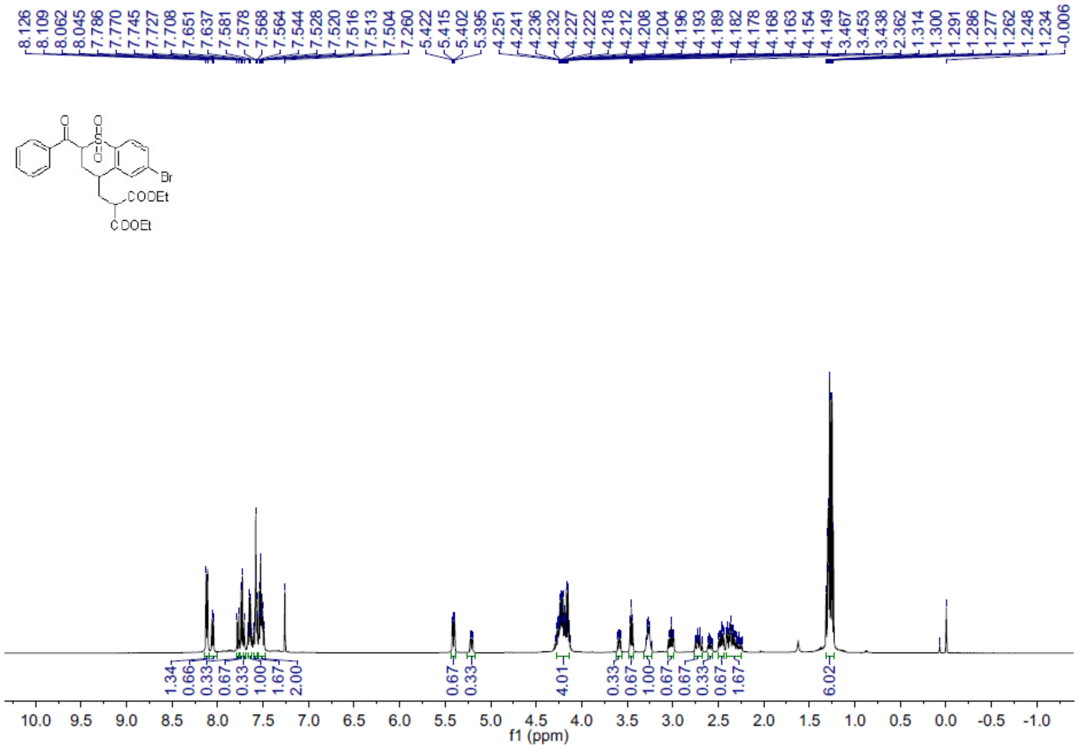


$^{13}$C {$^{1}$H} NMR (126 MHz, $CDCl_3$) spectrum of compound **4d**

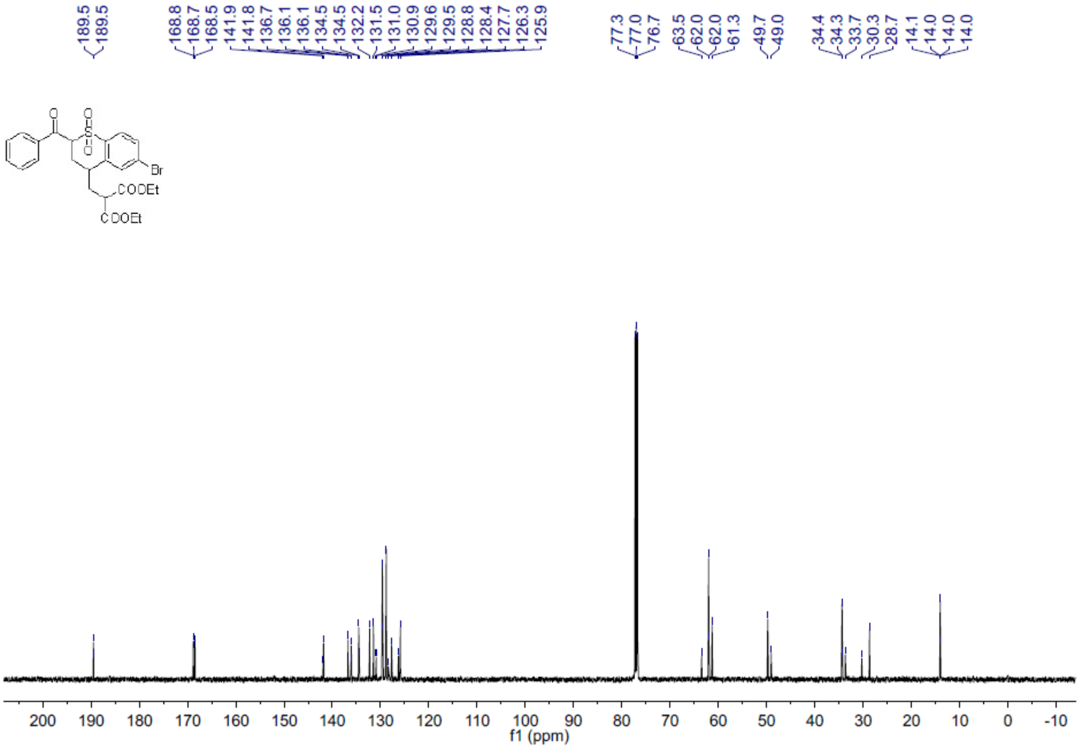

$^{1}$H NMR (500 MHz, CDCl3) spectrum of compound **4e**

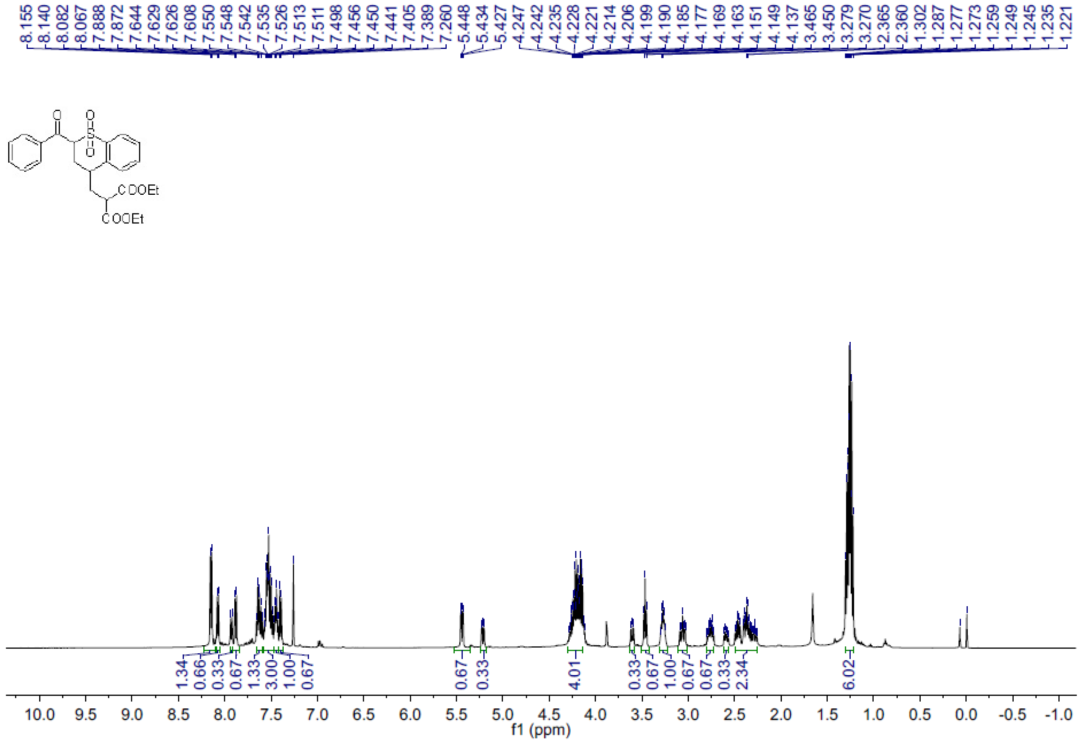


$^{13}$C {$^{1}$H} NMR (126 MHz, CDCl3) spectrum of compound **4e**

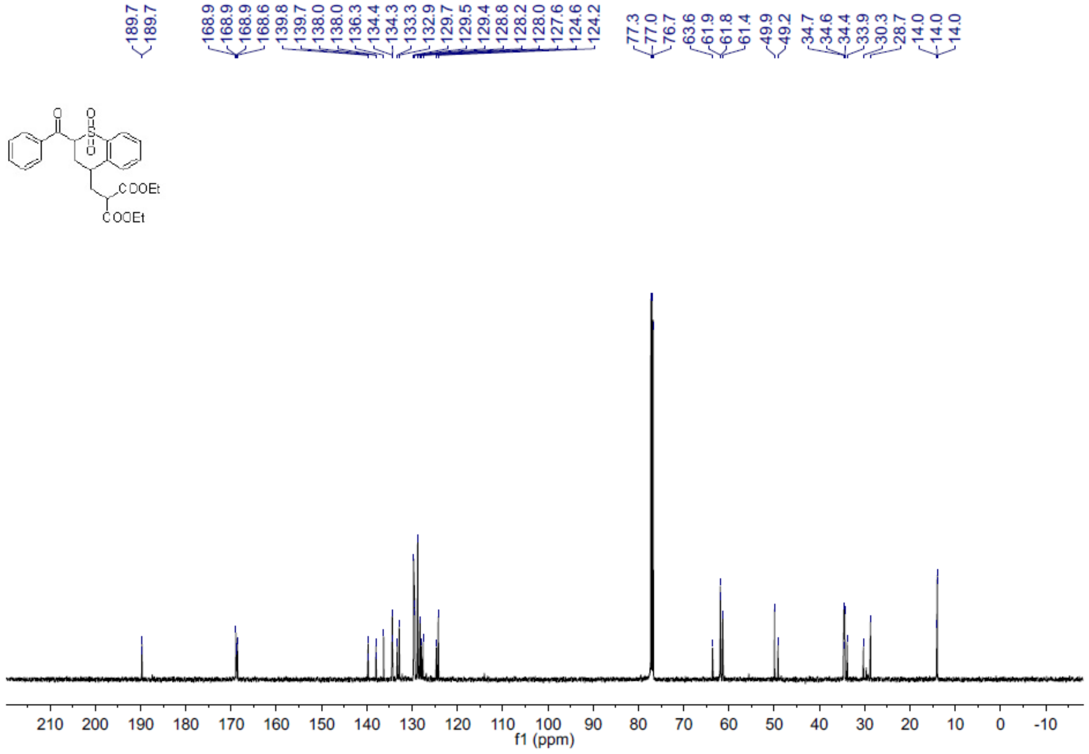

$^{1}$H NMR (500 MHz, $CDCl_3$) spectrum of compound **4f**

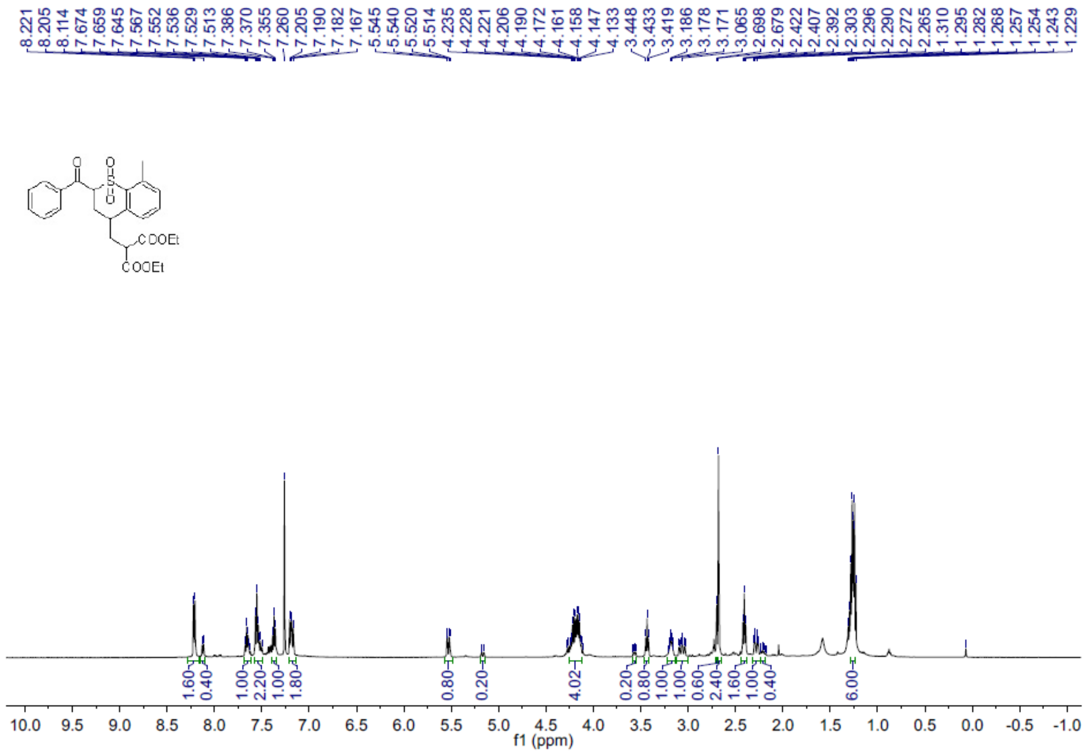


$^{13}$C {$^{1}$H} NMR (126 MHz, $CDCl_3$) spectrum of compound **4f**

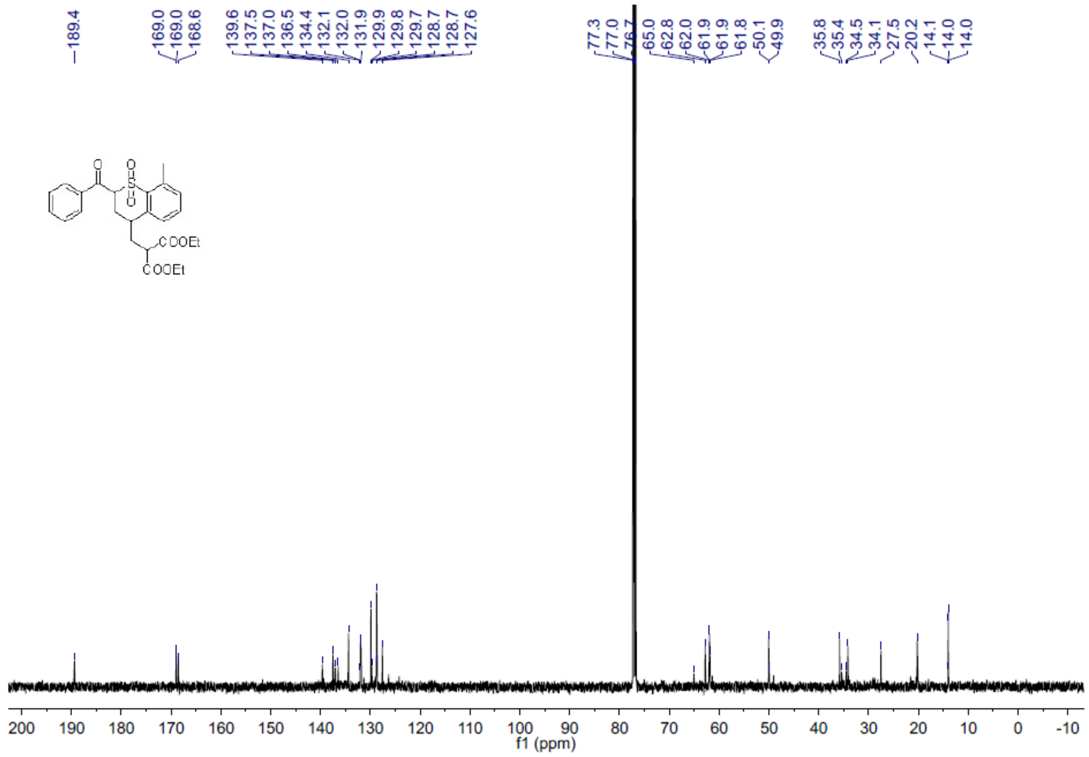

$^{1}H$ NMR (500 MHz, $CDCl_3$) spectrum of compound **4g**

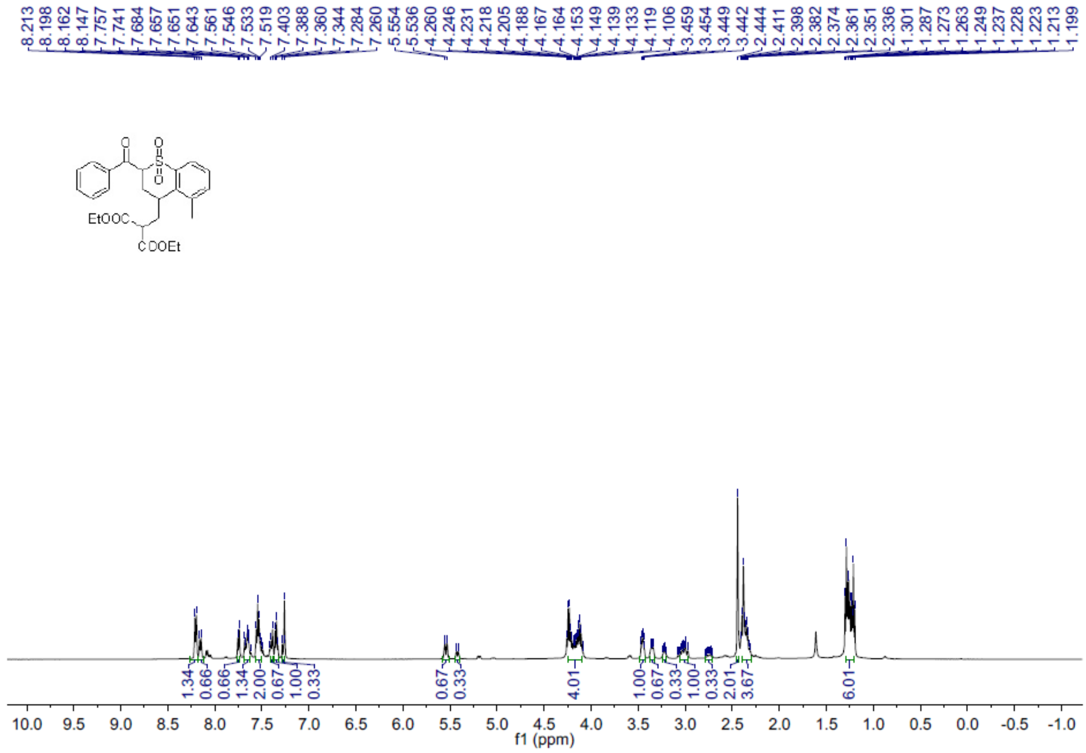

$^{13}C$ {$^{1}H$} NMR (126 MHz, $CDCl_3$) spectrum of compound **4g**

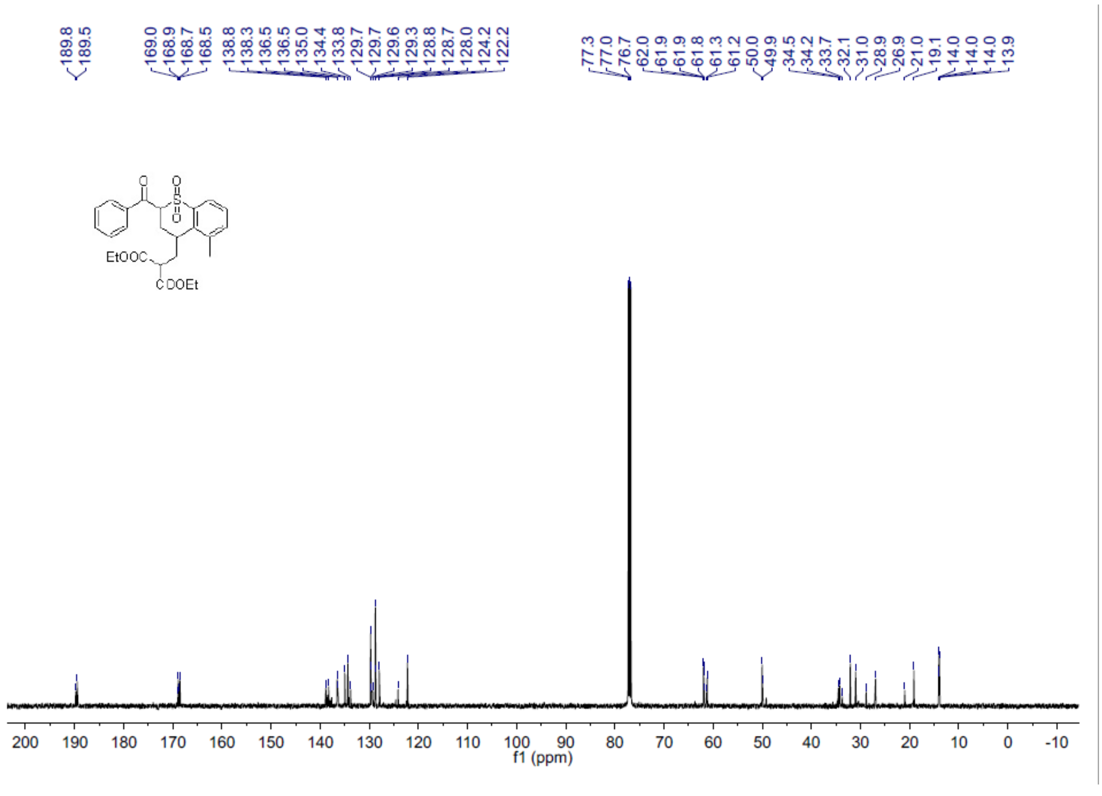

$^{1}$H NMR (500 MHz, $CDCl_3$) spectrum of compound **4h**

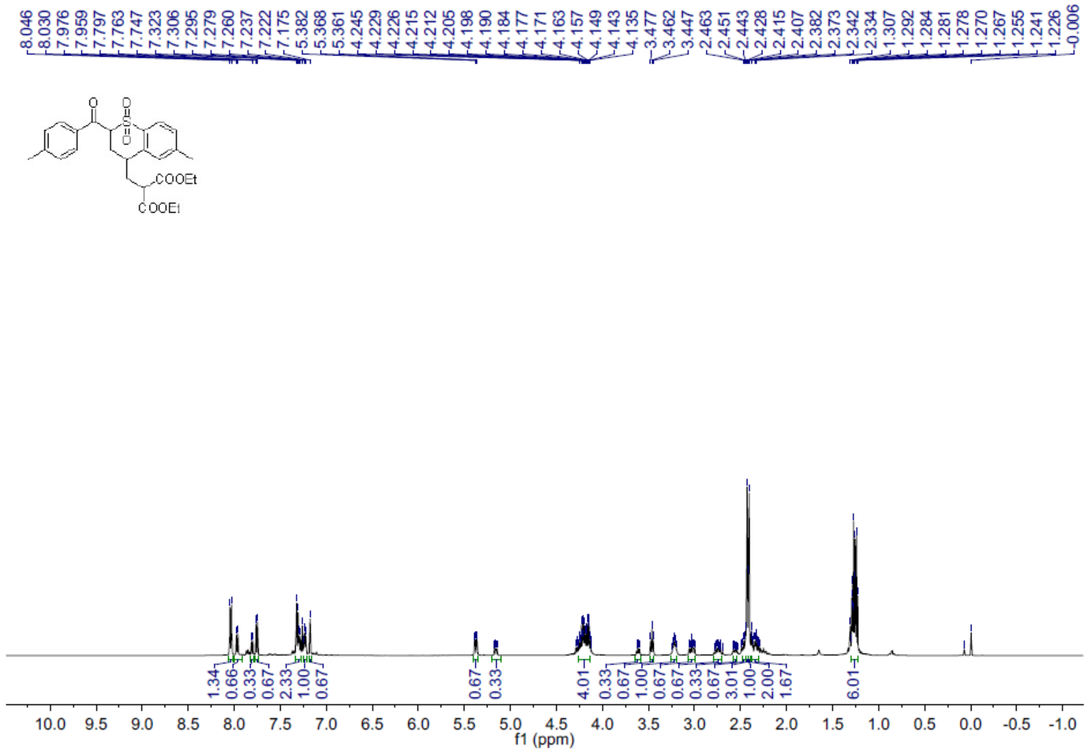


$^{13}$C {$^{1}$H} NMR (126 MHz, $CDCl_3$) spectrum of compound **4h**

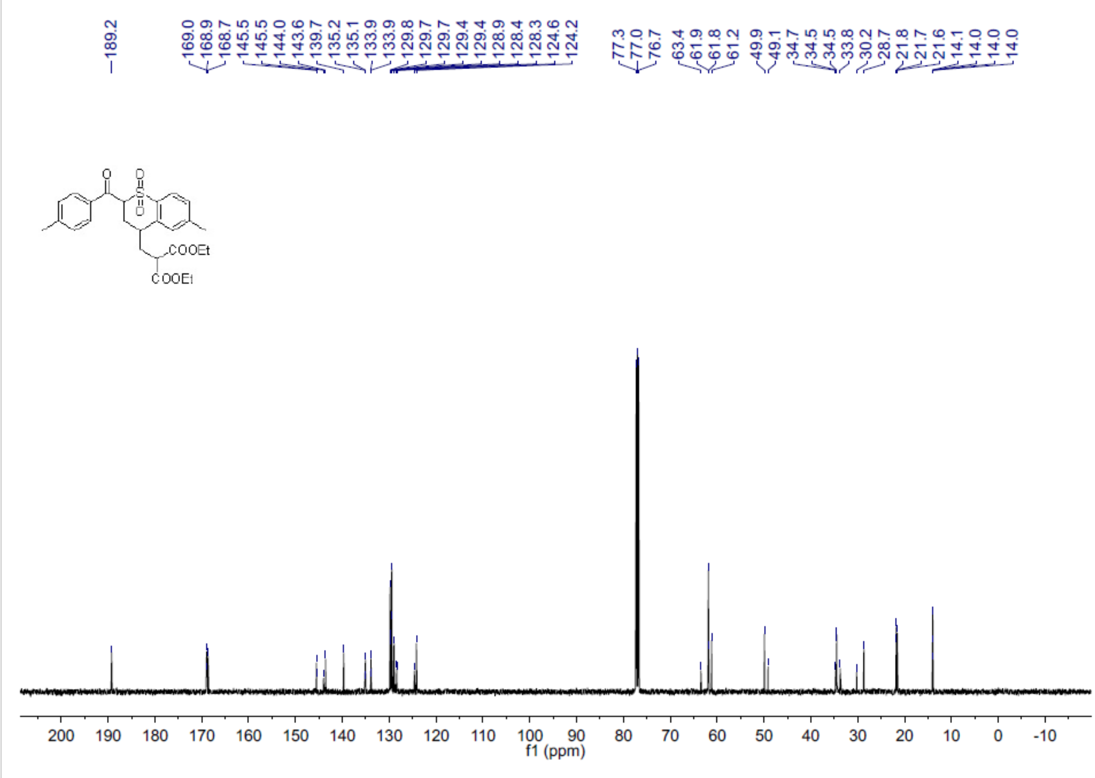

$^{1}$H NMR (500 MHz, $CDCl_3$) spectrum of compound **4i**

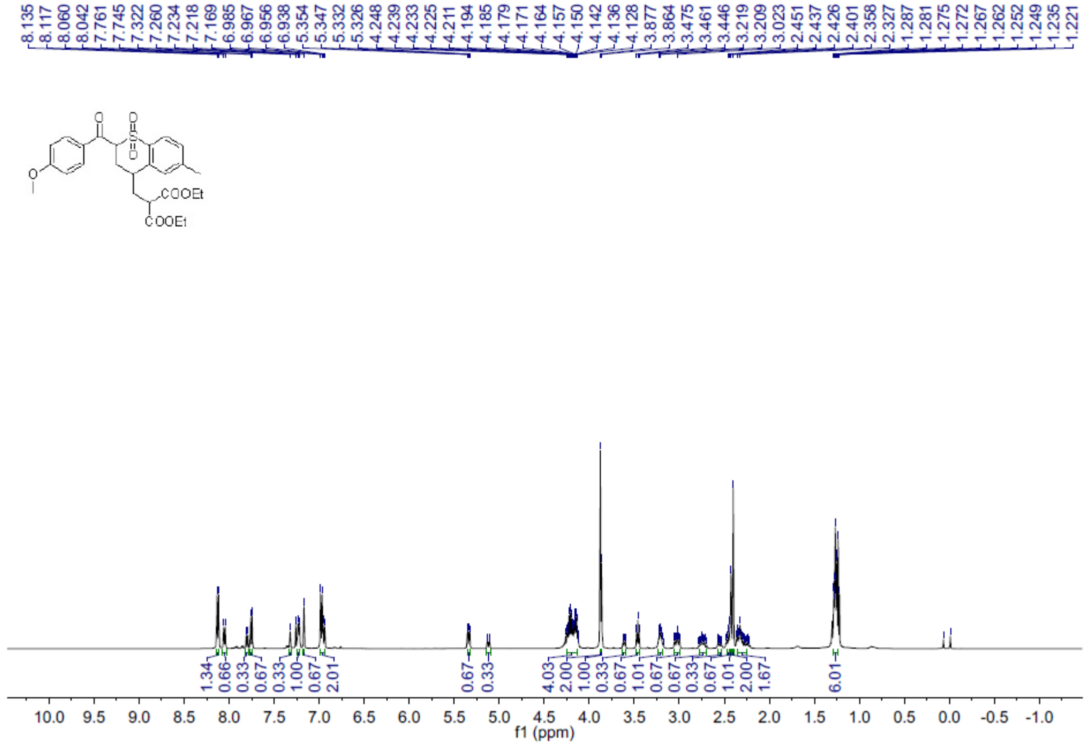

$^{13}$C {$^{1}$H} NMR (126 MHz, $CDCl_3$) spectrum of compound **4i**

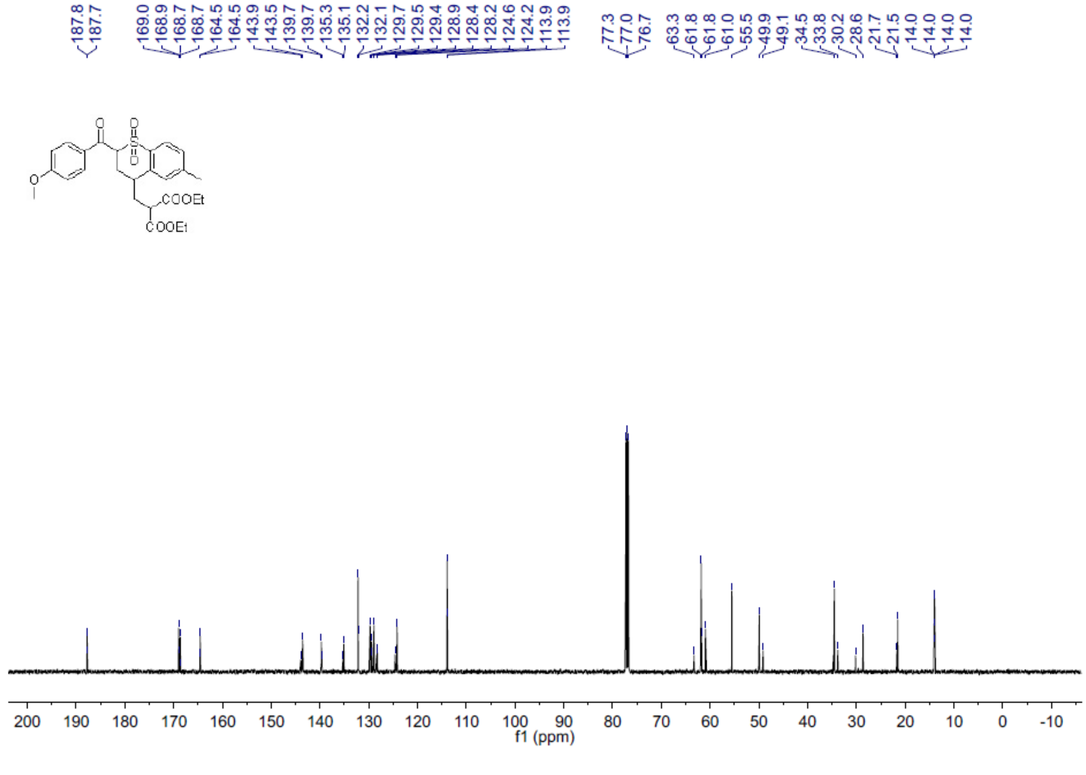

$^{1}H$ NMR (500 MHz, $CDCl_3$) spectrum of compound **4j**

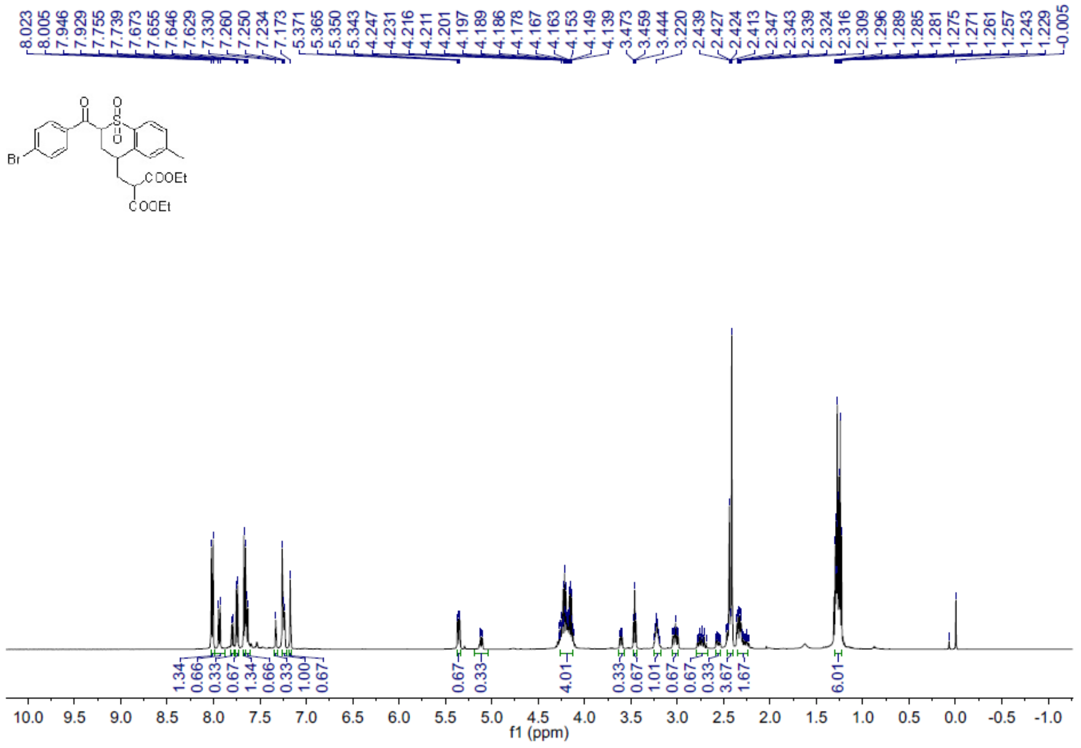

$^{13}C$ {$^{1}H$} NMR (126 MHz, $CDCl_3$) spectrum of compound **4j**

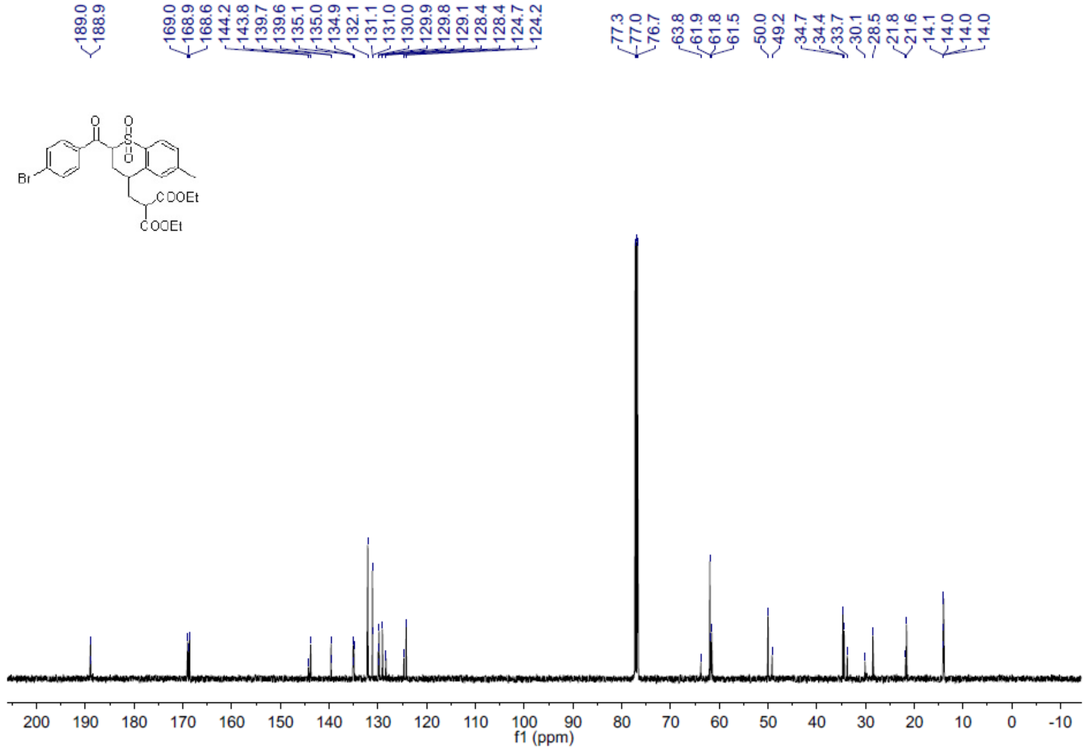

$^{1}$H NMR (500 MHz, CDCl$_3$) spectrum of compound **4k**

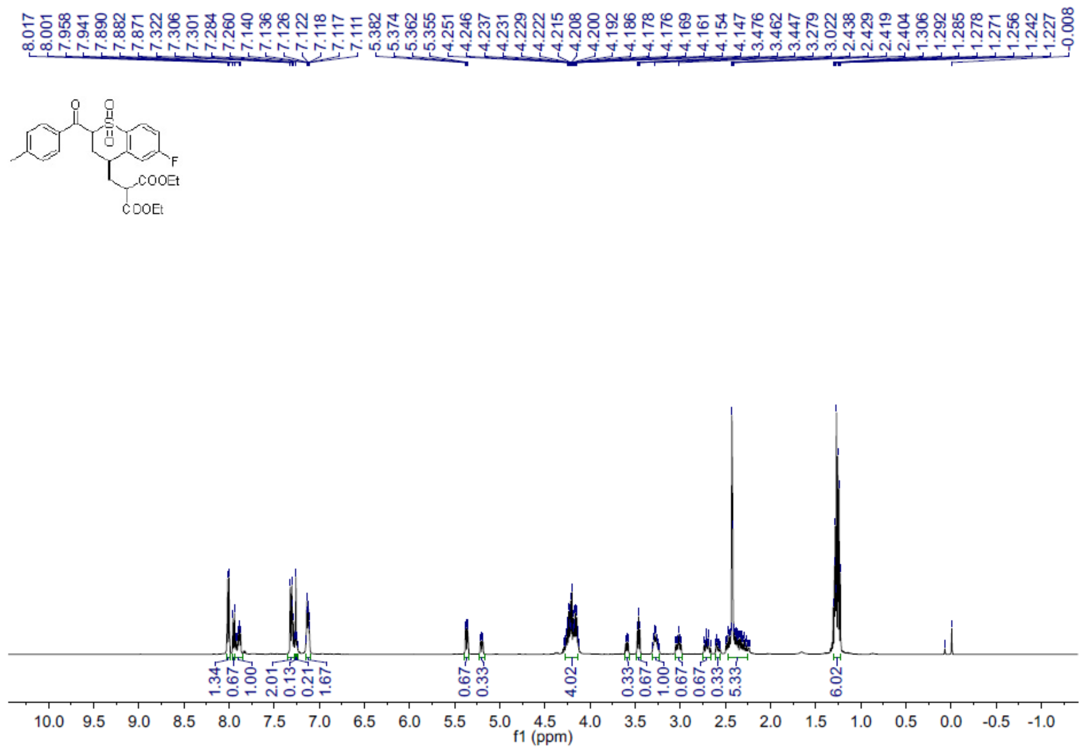


$^{13}$C {$^{1}$H} NMR (126 MHz, CDCl$_3$) spectrum of compound **4k**

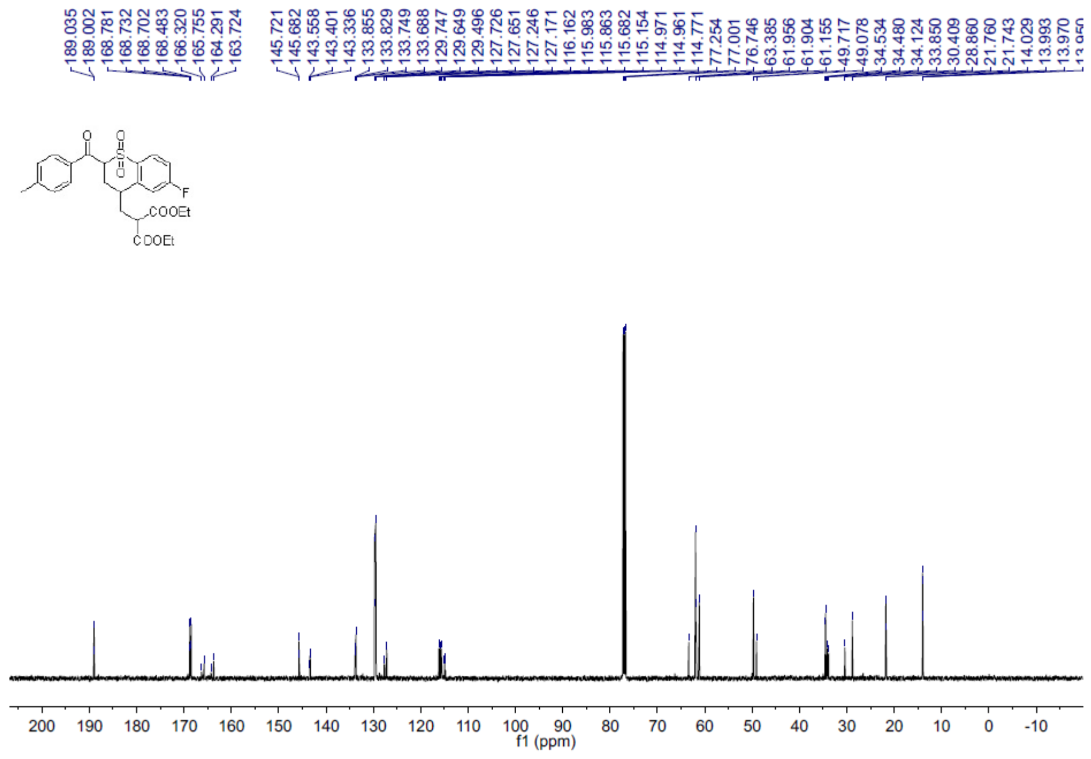

$^{19}$F NMR (470 MHz, $CDCl_3$) spectrum of compound **4k**

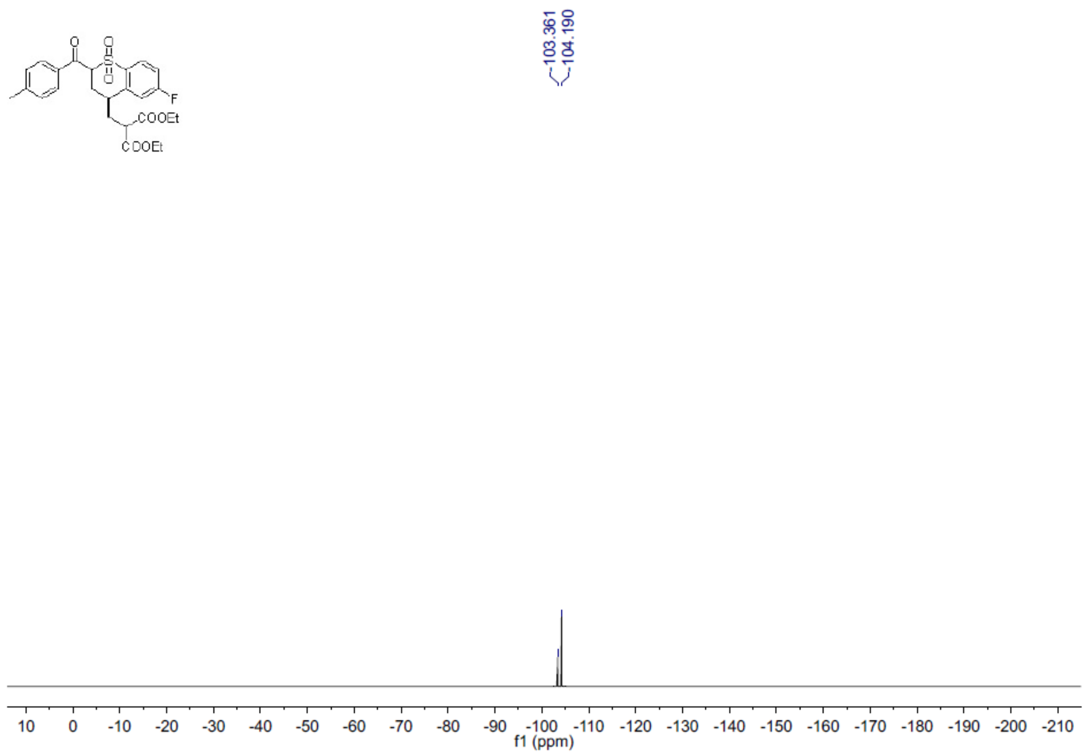


$^{1}$H NMR (500 MHz, $CDCl_3$) spectrum of compound **4l**

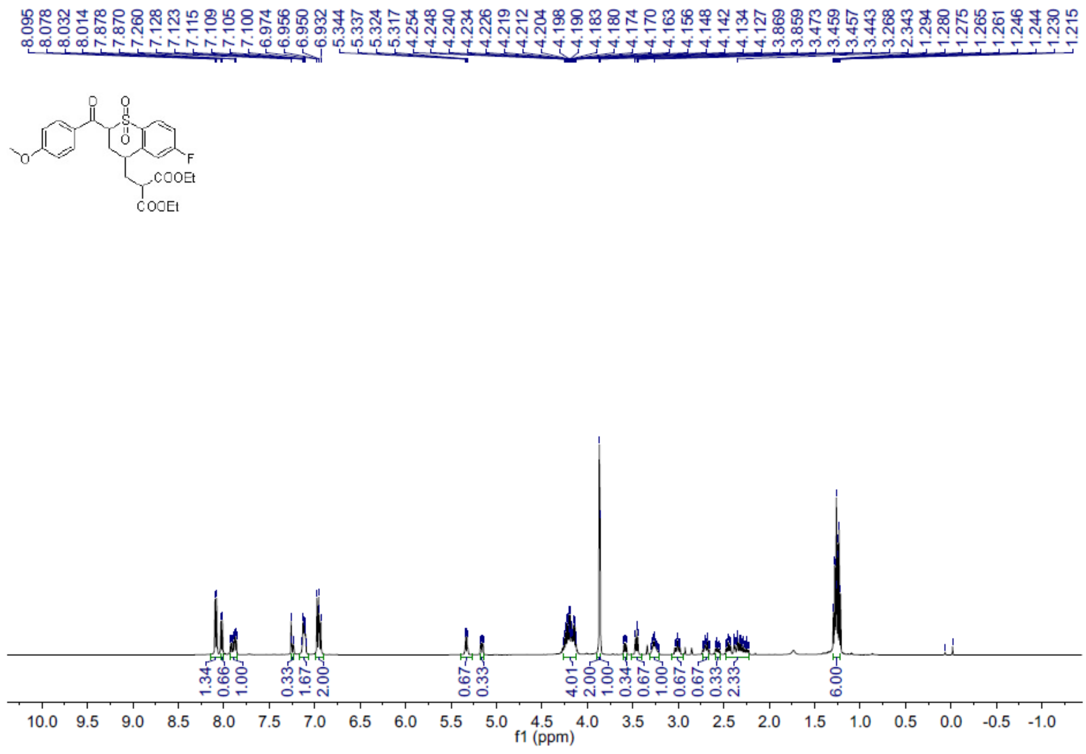

$^{13}C$ {$^{1}H$} NMR (126 MHz, $CDCl_3$) spectrum of compound **4l**

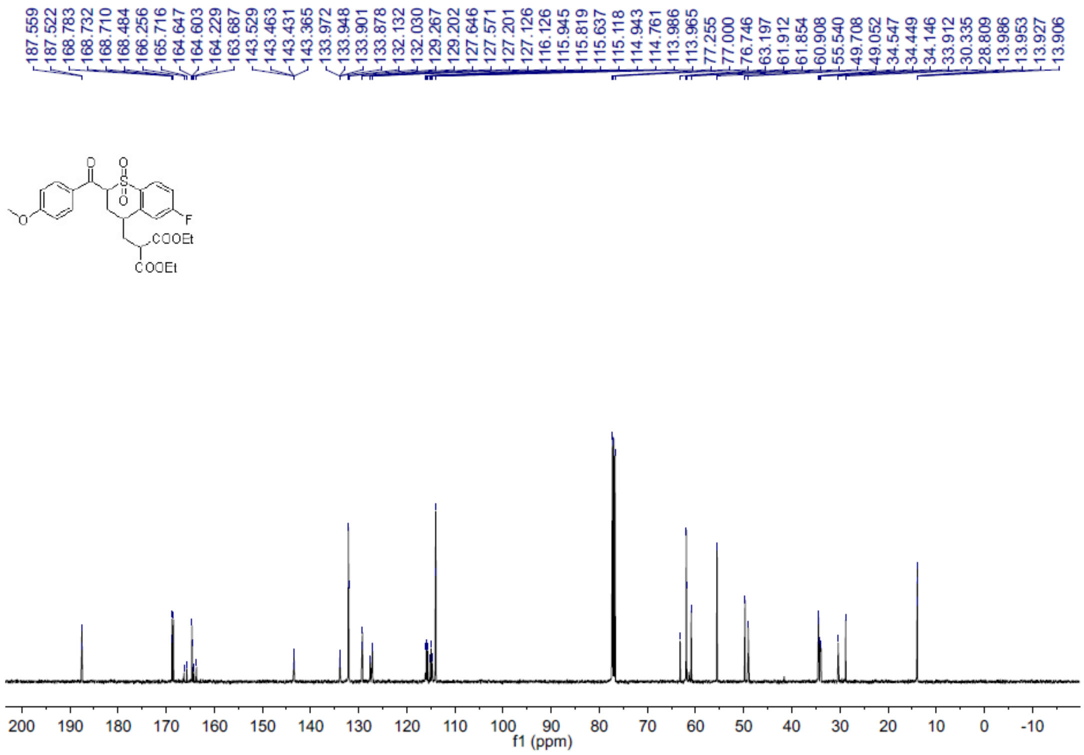


$^{19}F$ NMR (470 MHz, $CDCl_3$) spectrum of compound **4l**

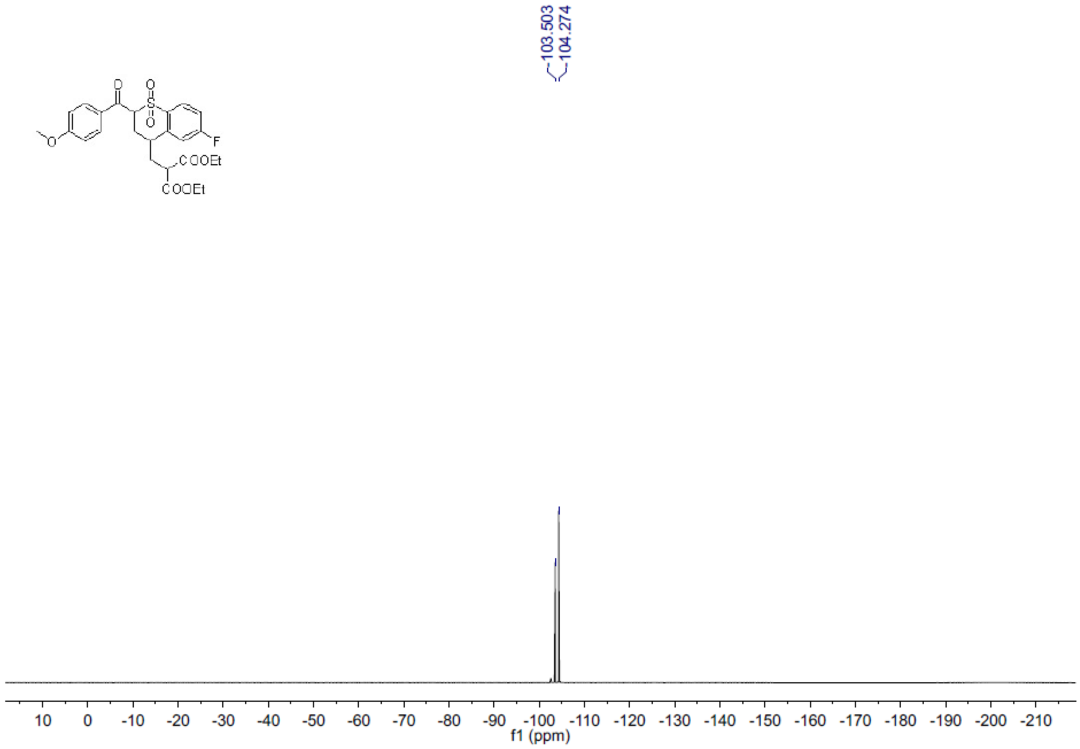

$^{1}$H NMR (500 MHz, $CDCl_3$) spectrum of compound **4m**

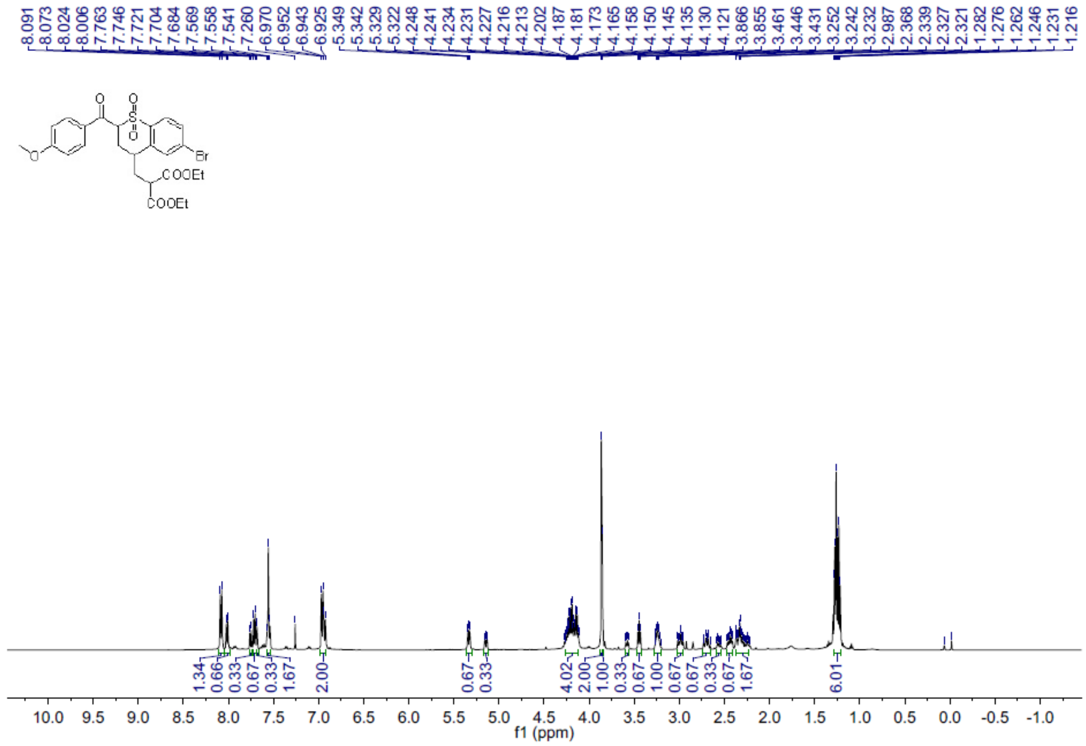


$^{13}$C {$^{1}$H} NMR (126 MHz, $CDCl_3$) spectrum of compound **4m**

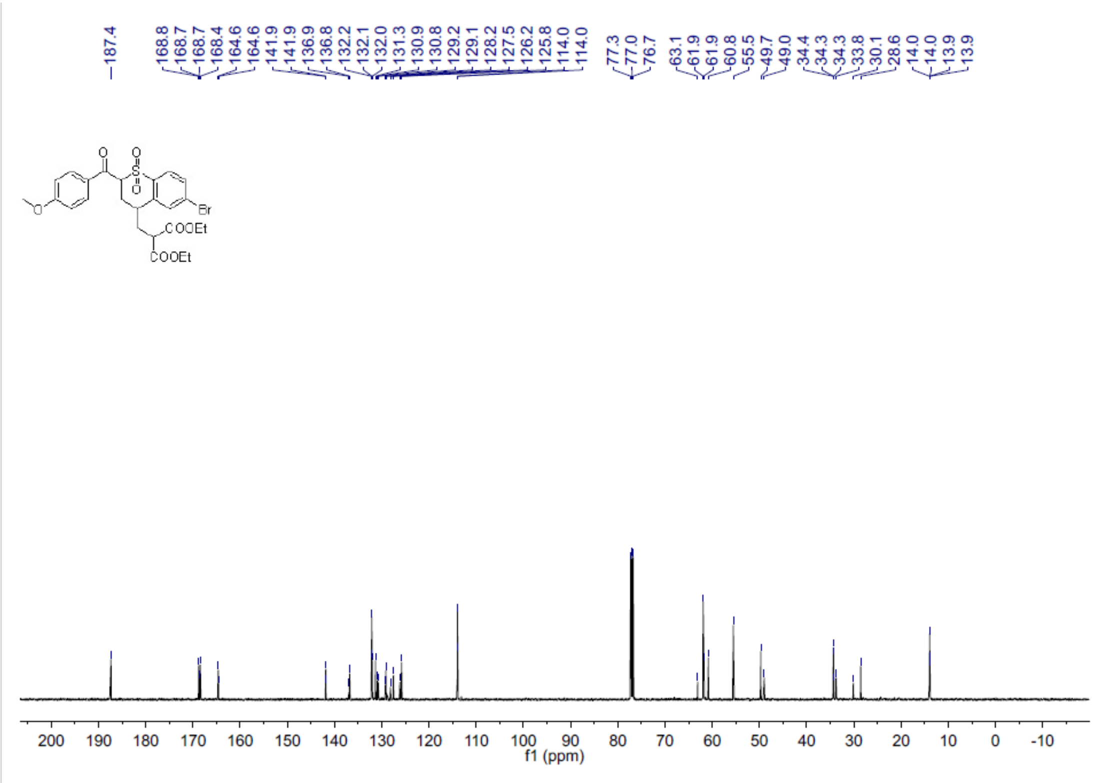

$^1$H NMR (500 MHz, $CDCl_3$) spectrum of compound **4n**

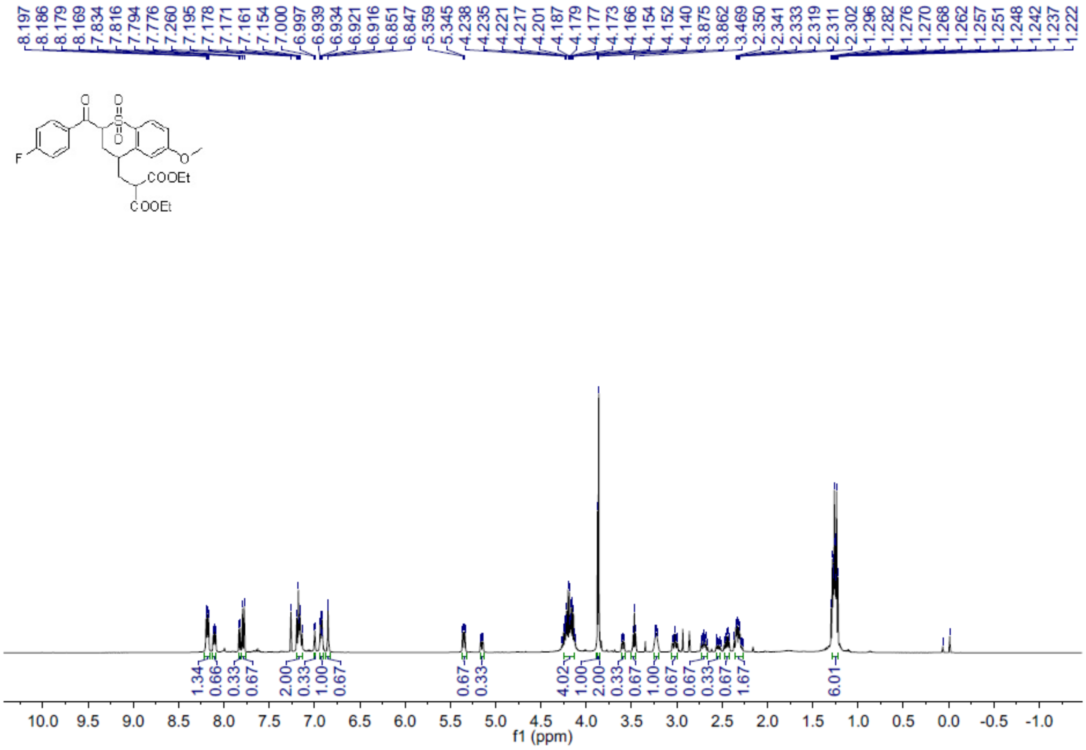


$^{13}$C {$^1$H} NMR (126 MHz, $CDCl_3$) spectrum of compound **4n**

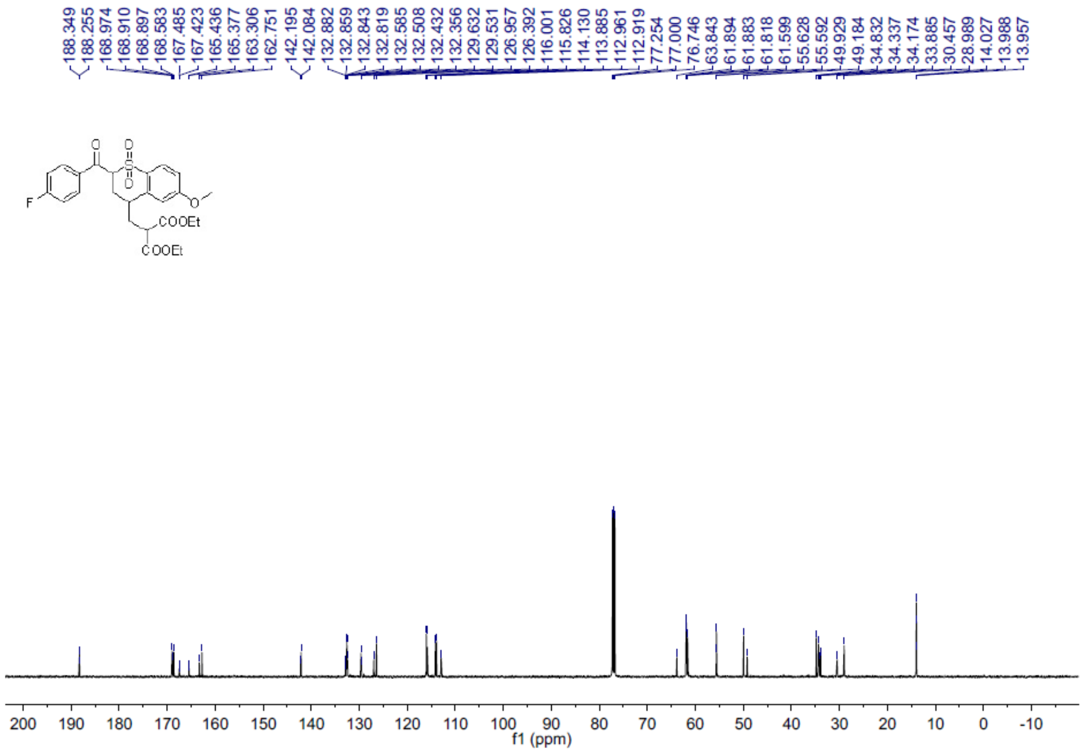

$^{19}$F NMR (470 MHz, $CDCl_3$) spectrum of compound **4n**

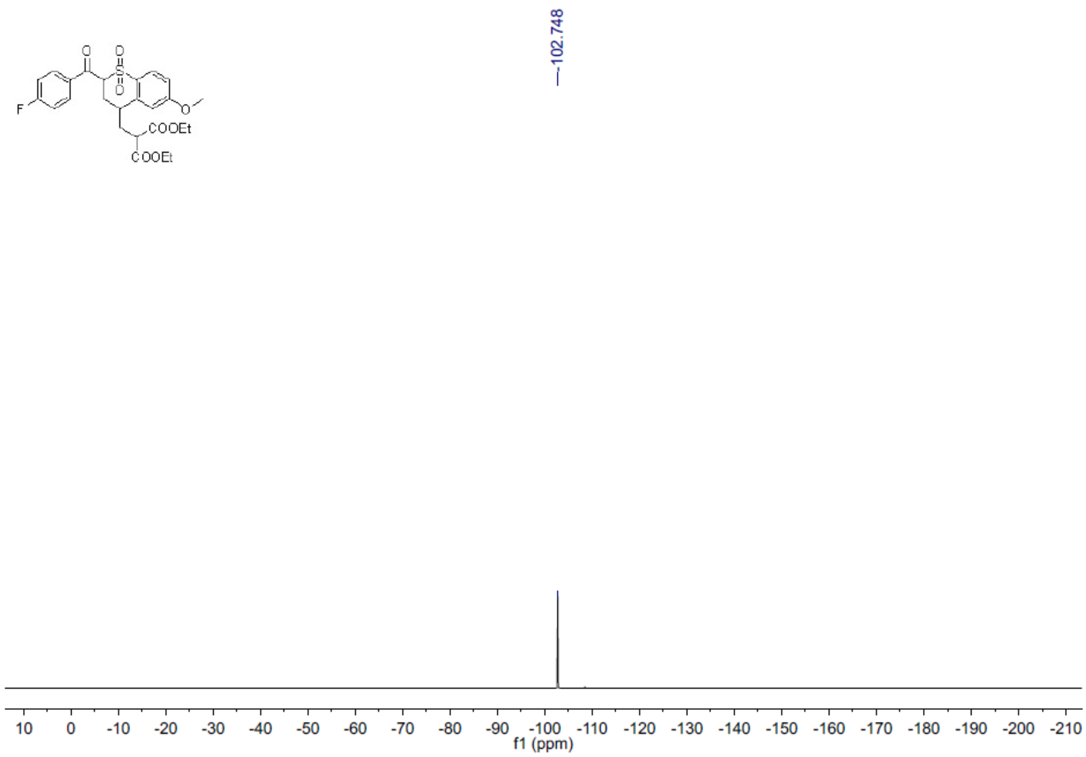


$^{1}$H NMR (500 MHz, $CDCl_3$) spectrum of compound **4o**

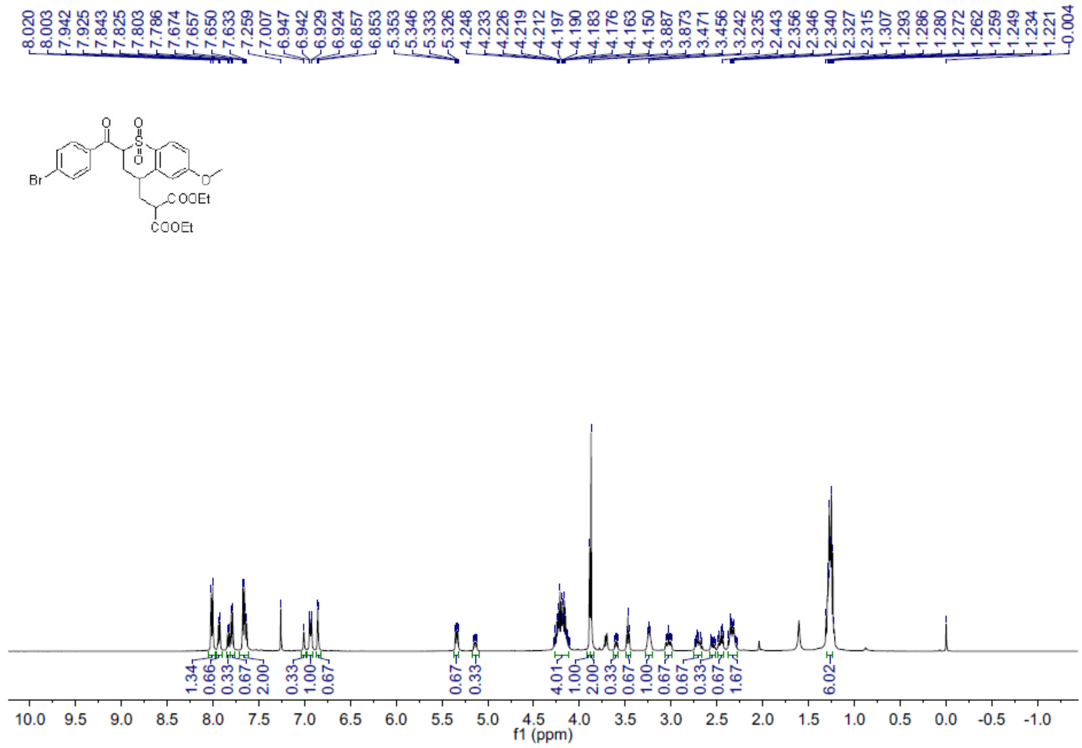

$^{13}C\ \{^{1}H\}$ NMR (126 MHz, $CDCl_3$) spectrum of compound **4o**

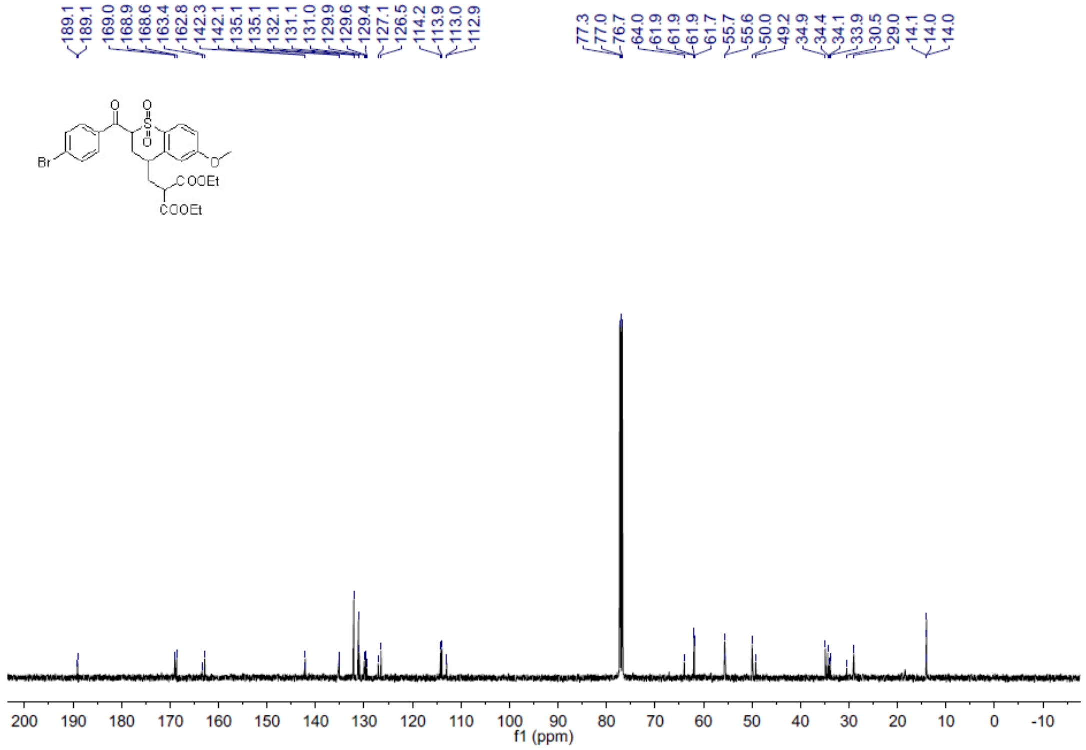

$^{1}H$ NMR (500 MHz, $CDCl_3$) spectrum of compound **4p**

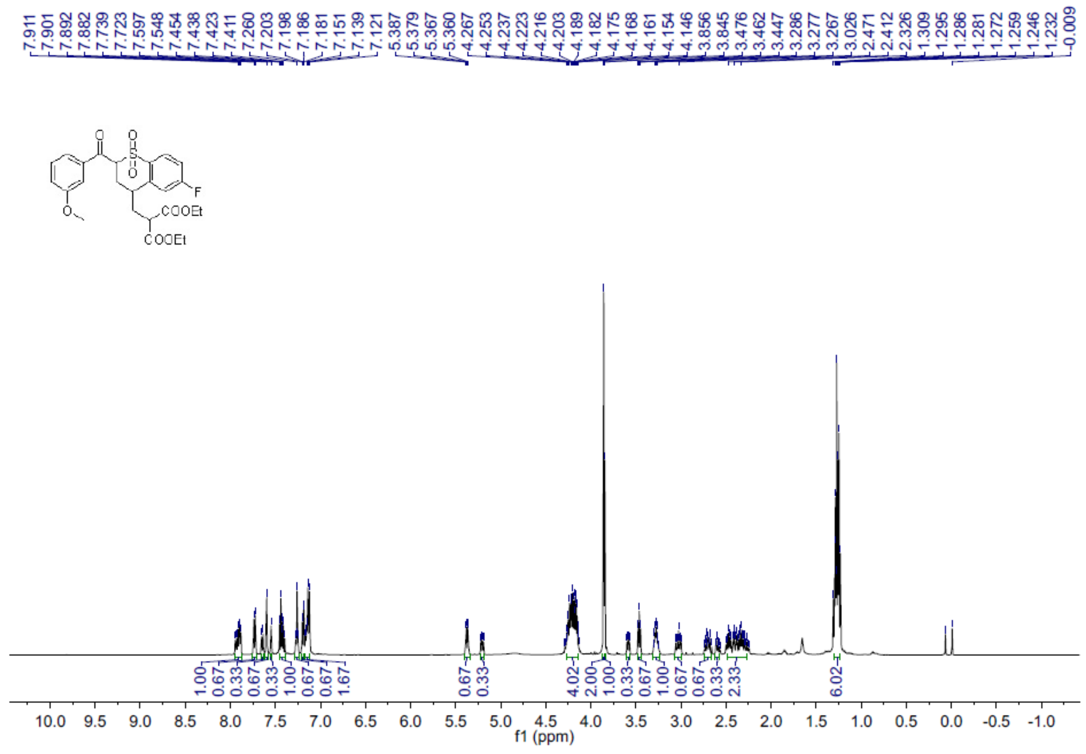

$^{13}C$ {$^{1}H$} NMR (126 MHz, $CDCl_3$) spectrum of compound **4p**

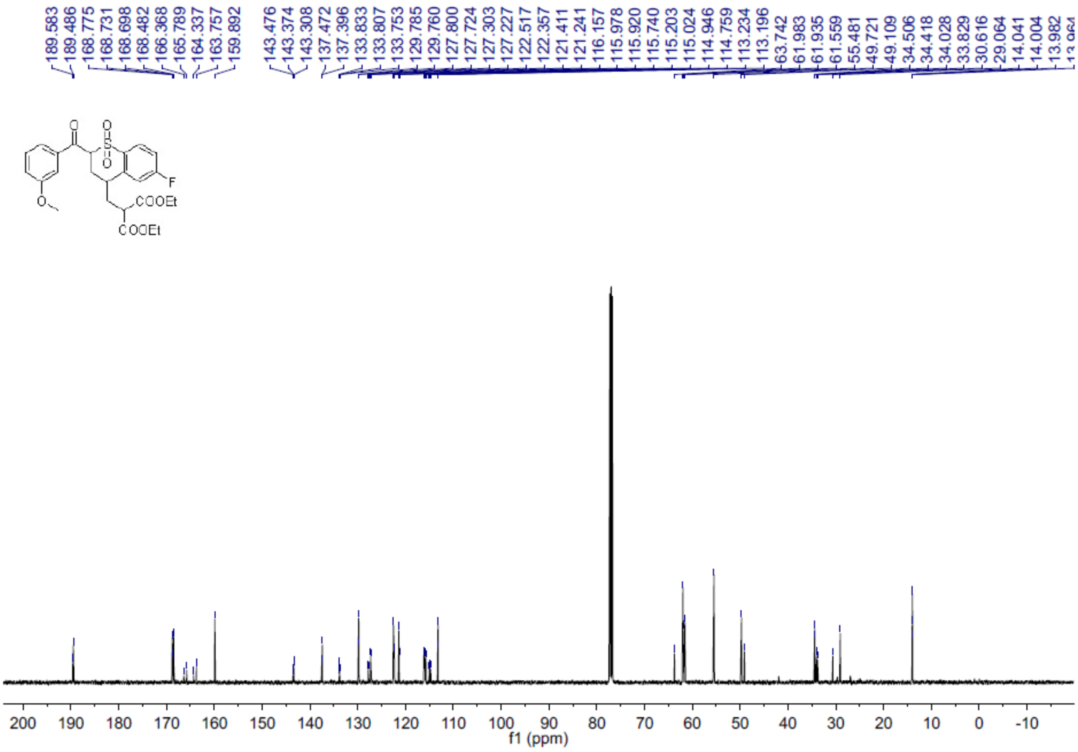

$^{19}F$ NMR (470 MHz, $CDCl_3$) spectrum of compound **4p**

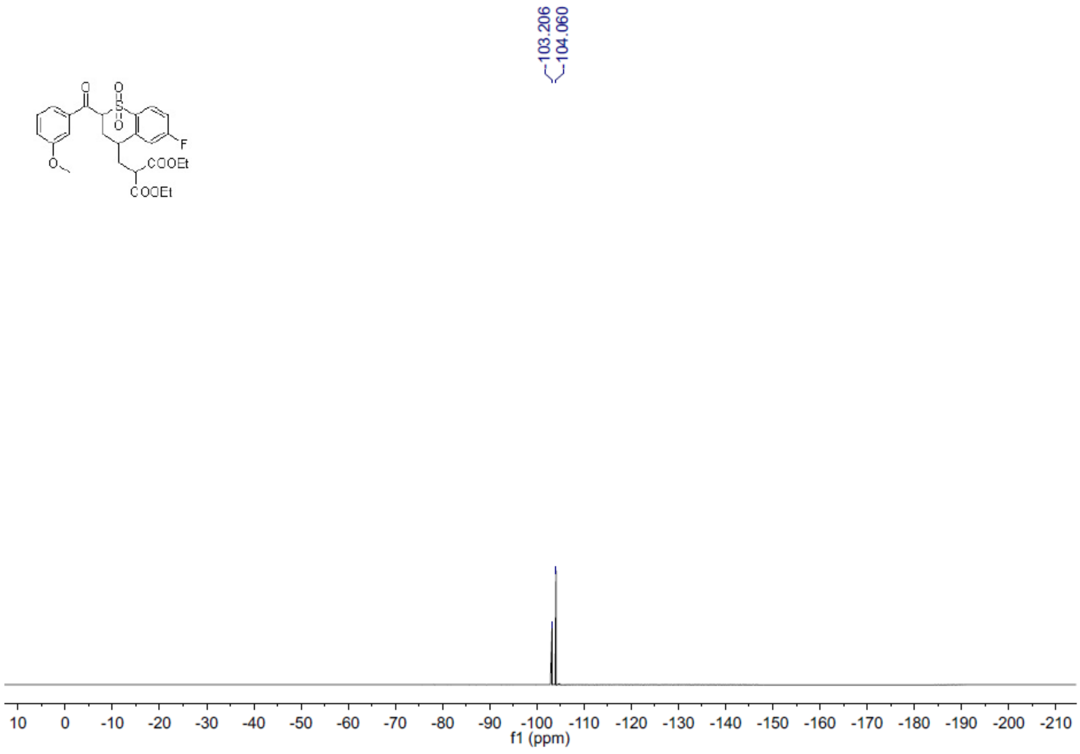

$^{1}$H NMR (500 MHz, $CDCl_3$) spectrum of compound **4q**

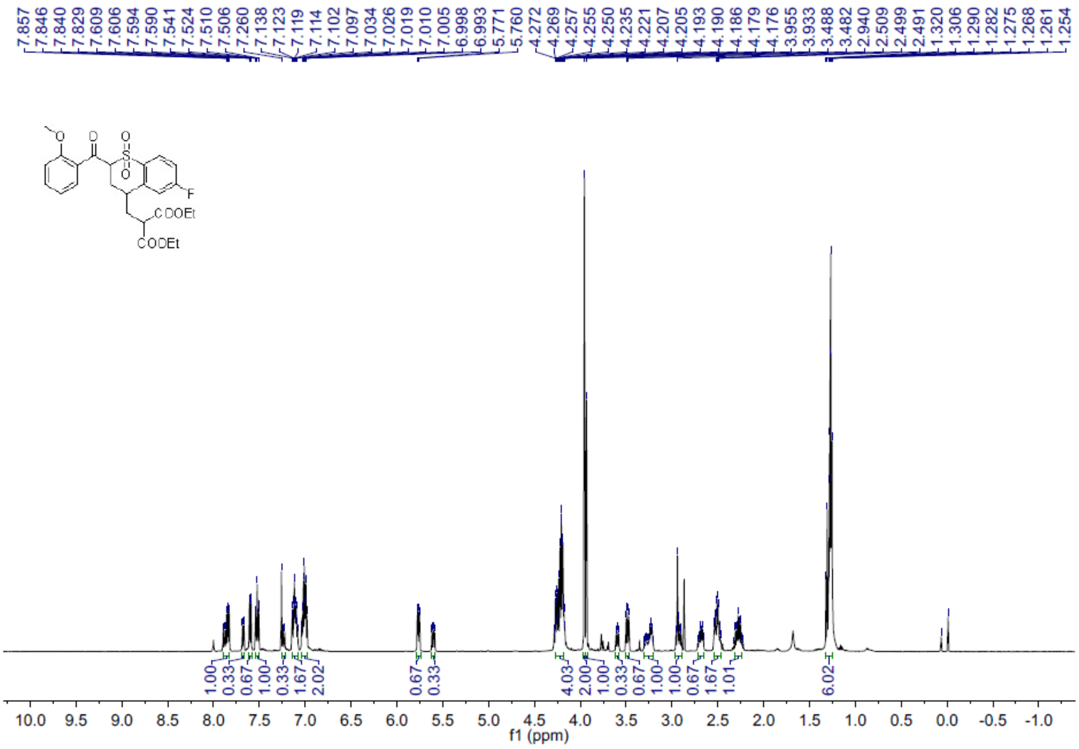


$^{13}$C {$^{1}$H} NMR (126 MHz, $CDCl_3$) spectrum of compound **4q**

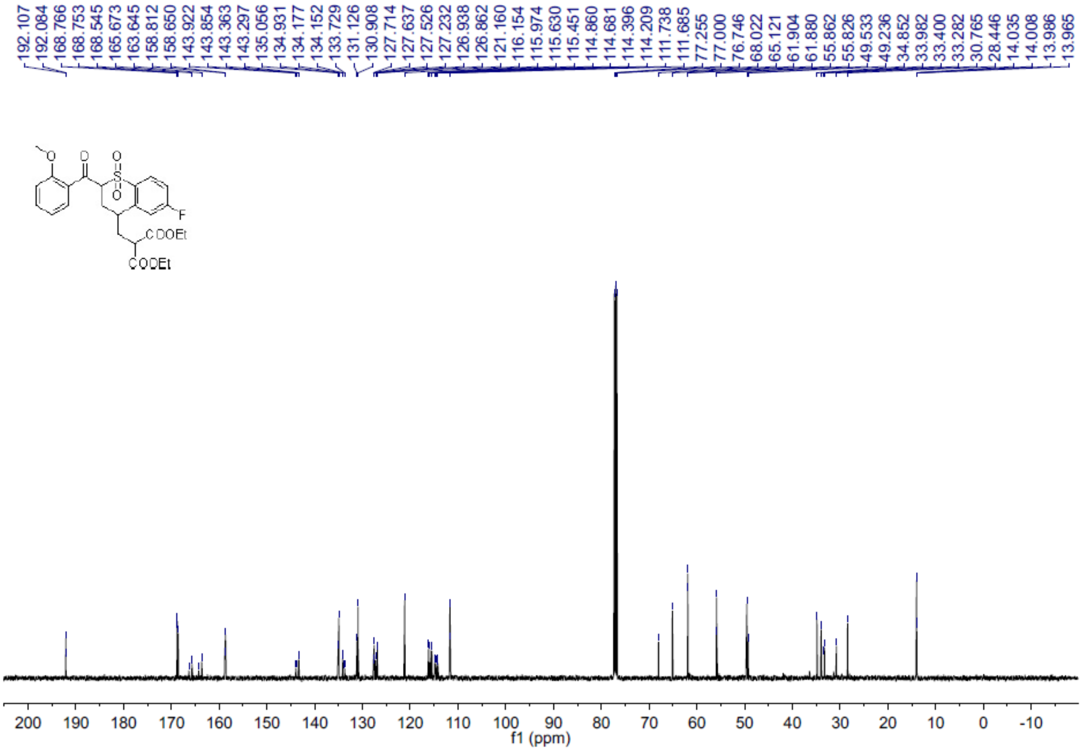

$^{19}F$ NMR (470 MHz, $CDCl_3$) spectrum of compound **4q**

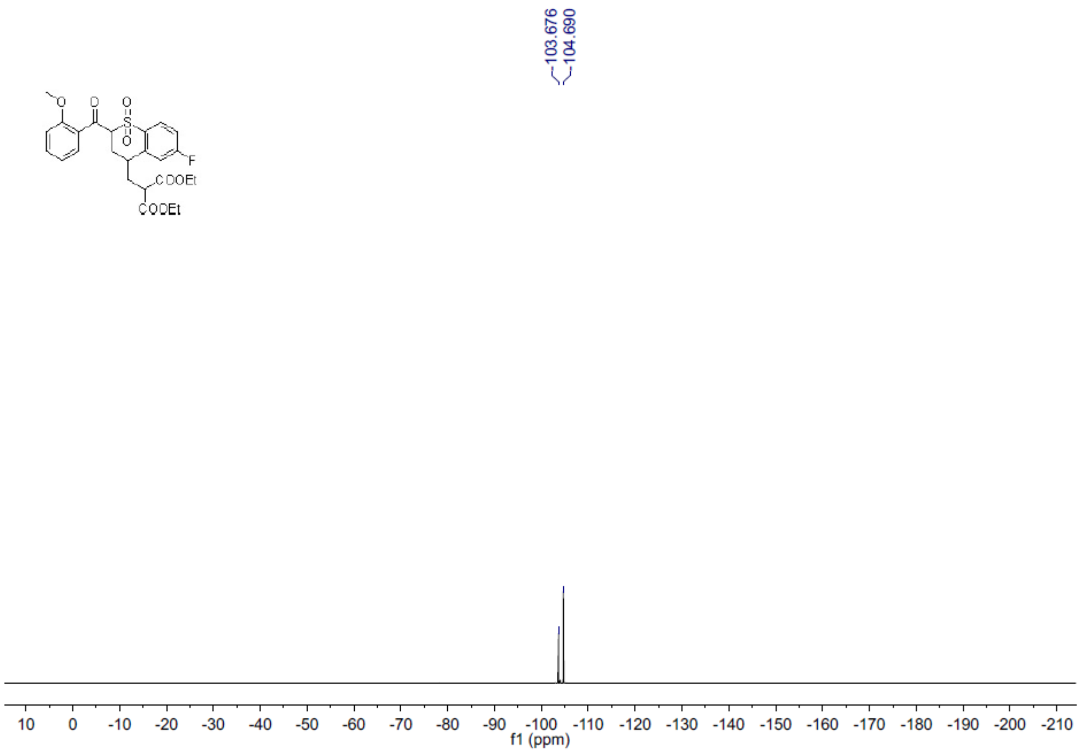


$^{1}H$ NMR (500 MHz, $CDCl_3$) spectrum of compound **5ab**

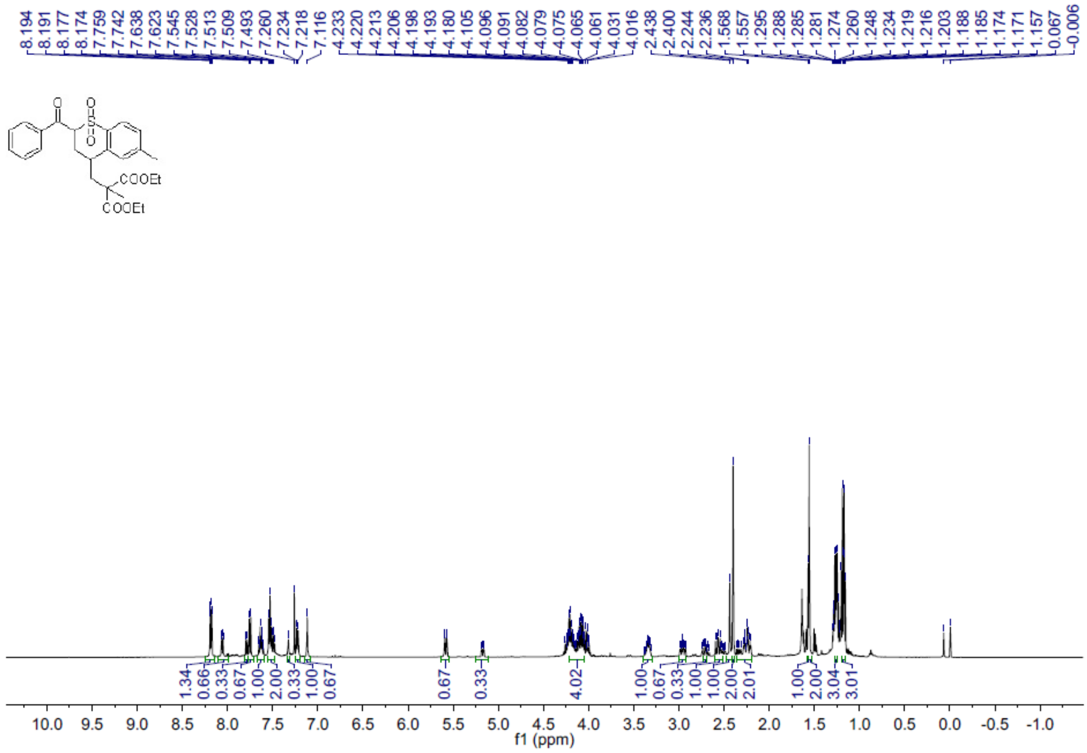

$^{13}C\ \{^{1}H\}$ NMR (126 MHz, $CDCl_3$) spectrum of compound **5ab**

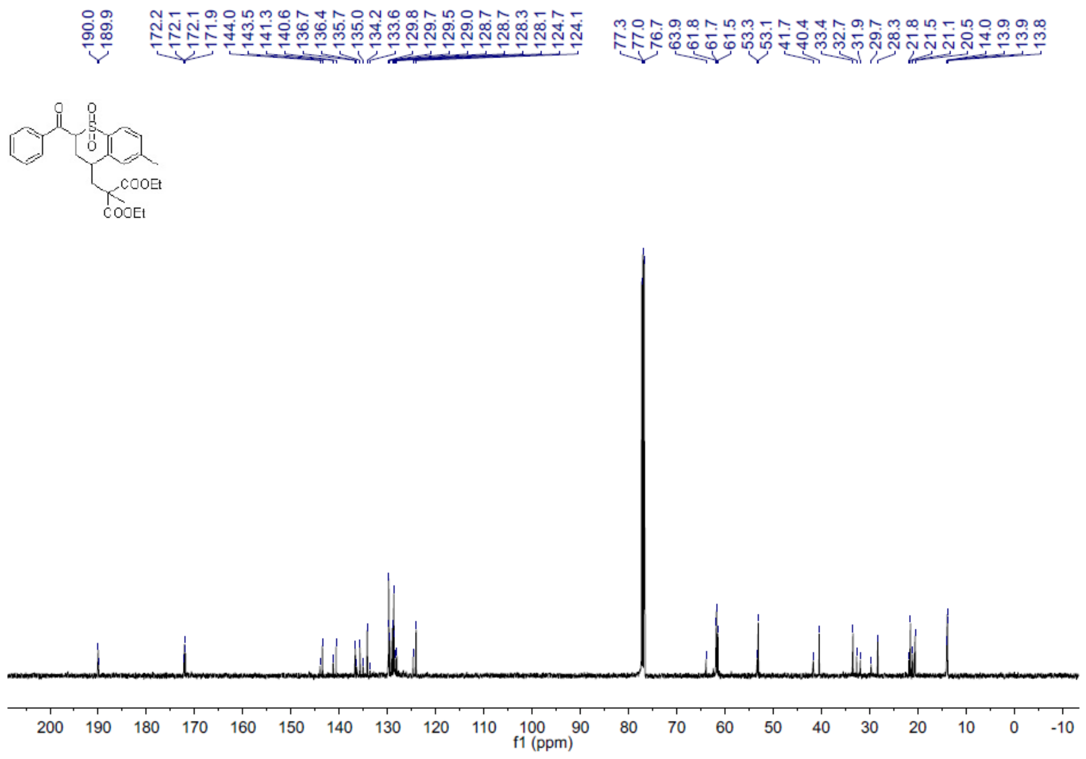

$^{1}H$ NMR (500 MHz, $CDCl_3$) spectrum of compound **5ac**

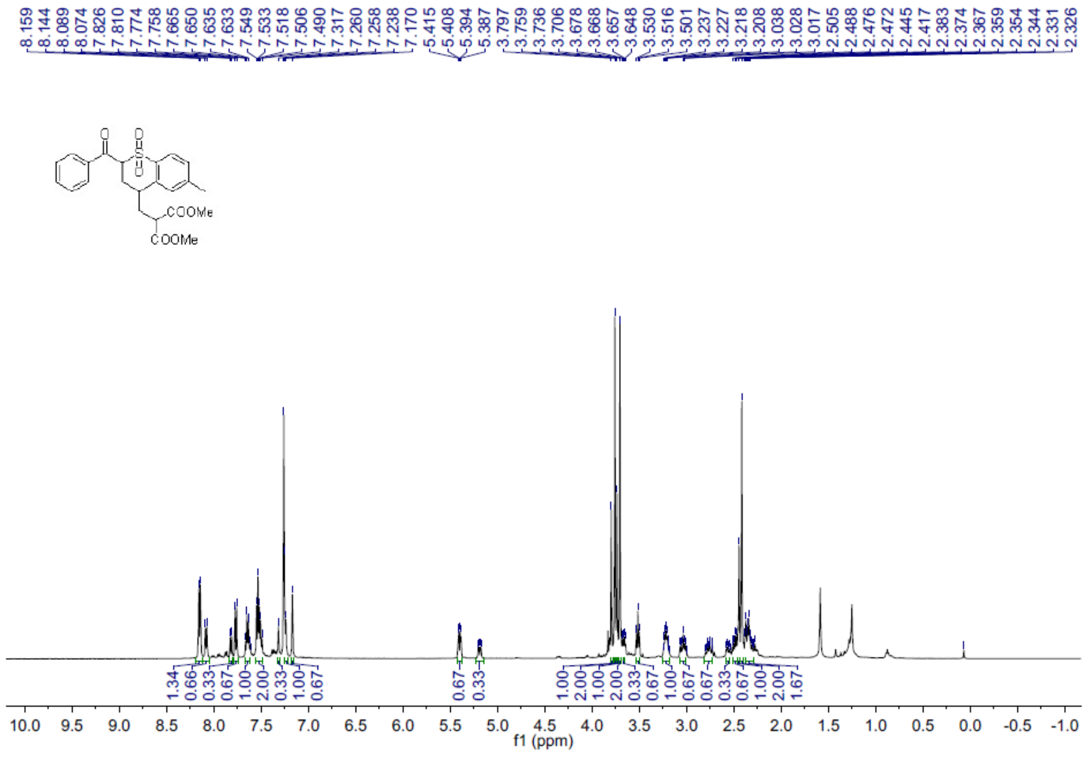

$^{13}C$ {$^{1}H$} NMR (126 MHz, $CDCl_3$) spectrum of compound **5ac**

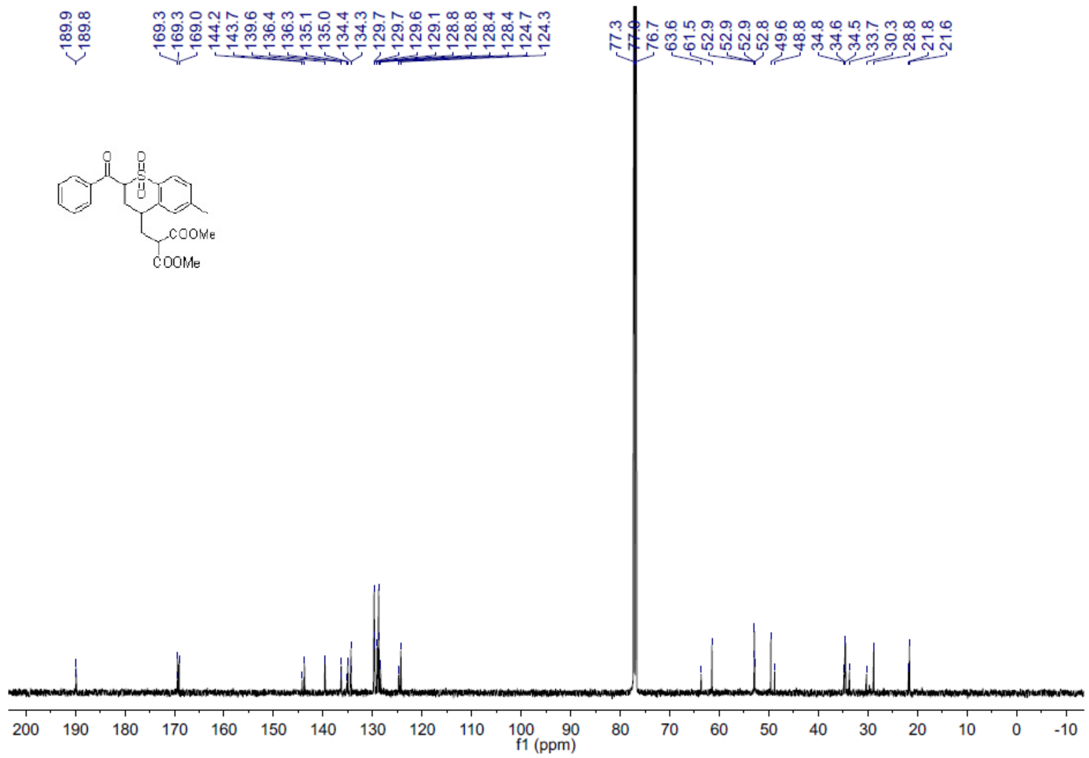

$^{1}H$ NMR (500 MHz, $CDCl_3$) spectrum of compound **5af**

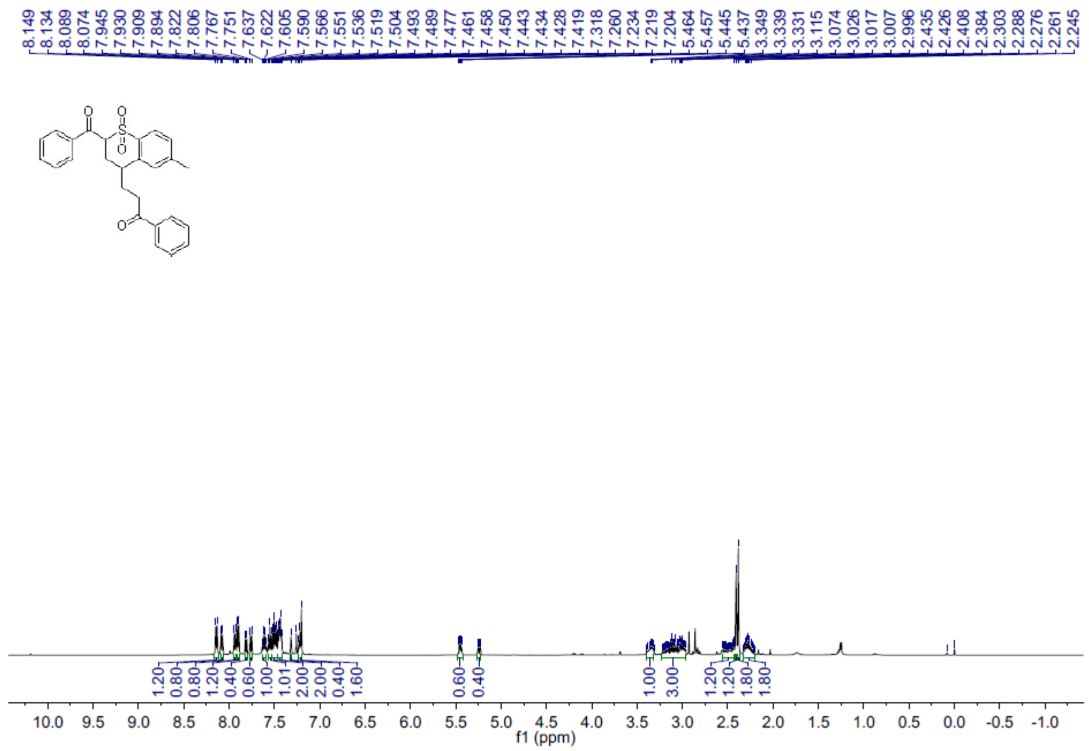

$^{13}C$ {$^{1}H$} NMR (126 MHz, $CDCl_3$) spectrum of compound **5af**

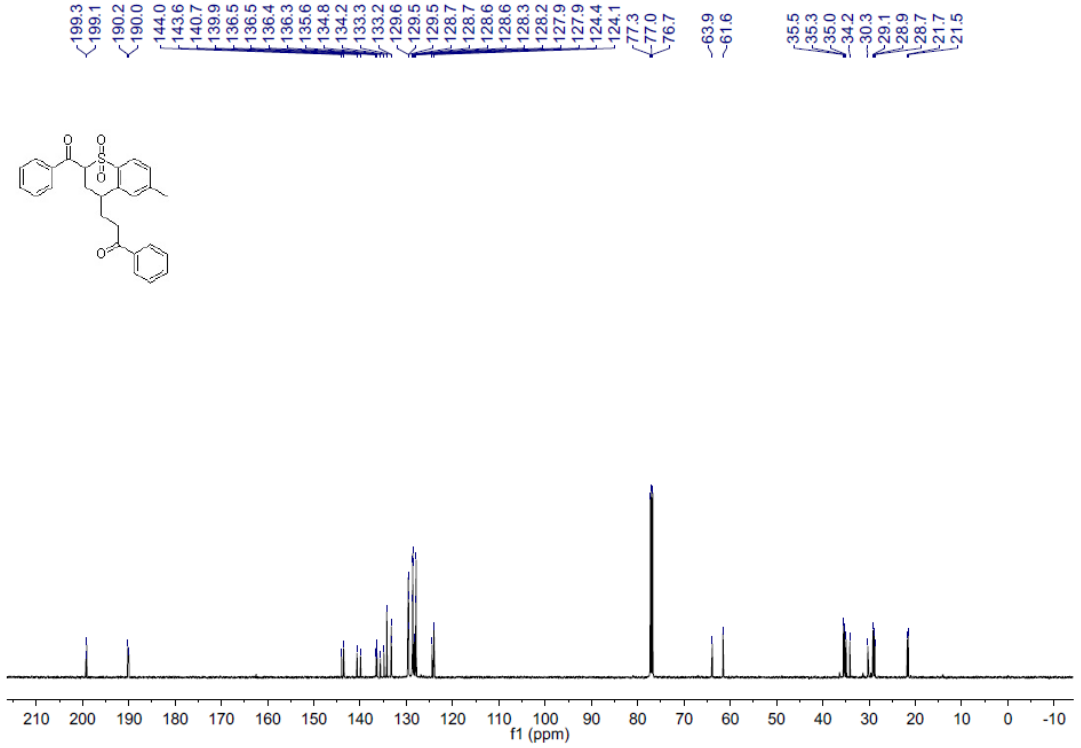


$^{1}H$ NMR (500 MHz, $CDCl_3$) spectrum of compound **5ag**

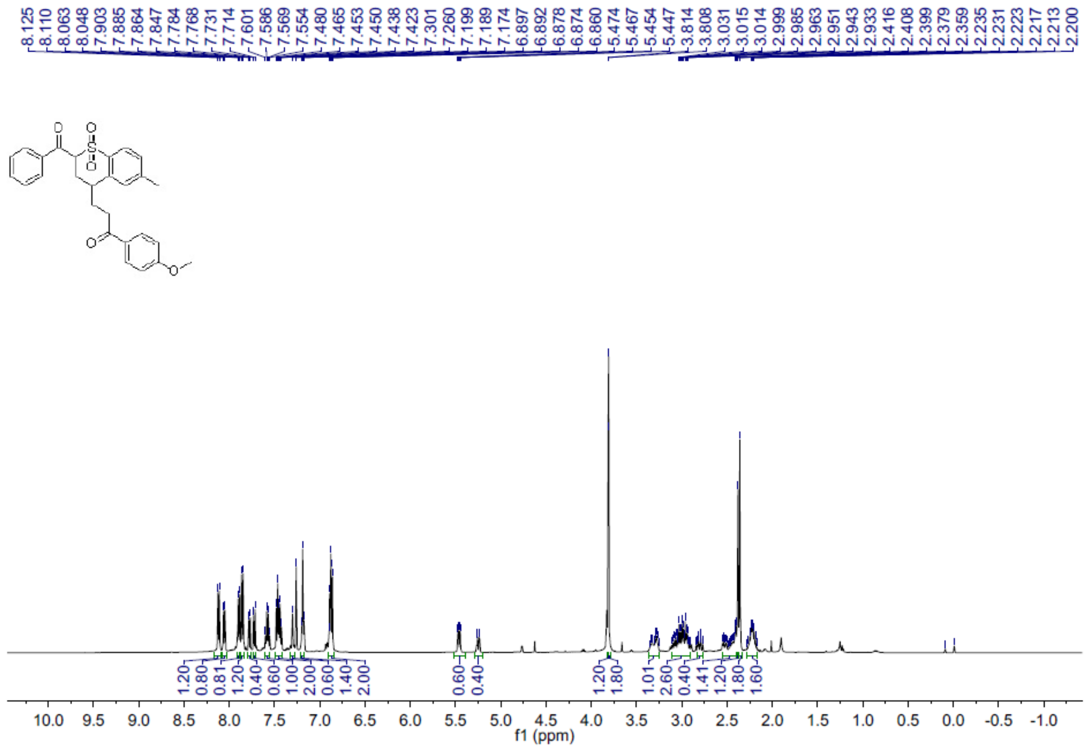

$^{13}$C {$^{1}$H} NMR (126 MHz, $CDCl_3$) spectrum of compound **5ag**

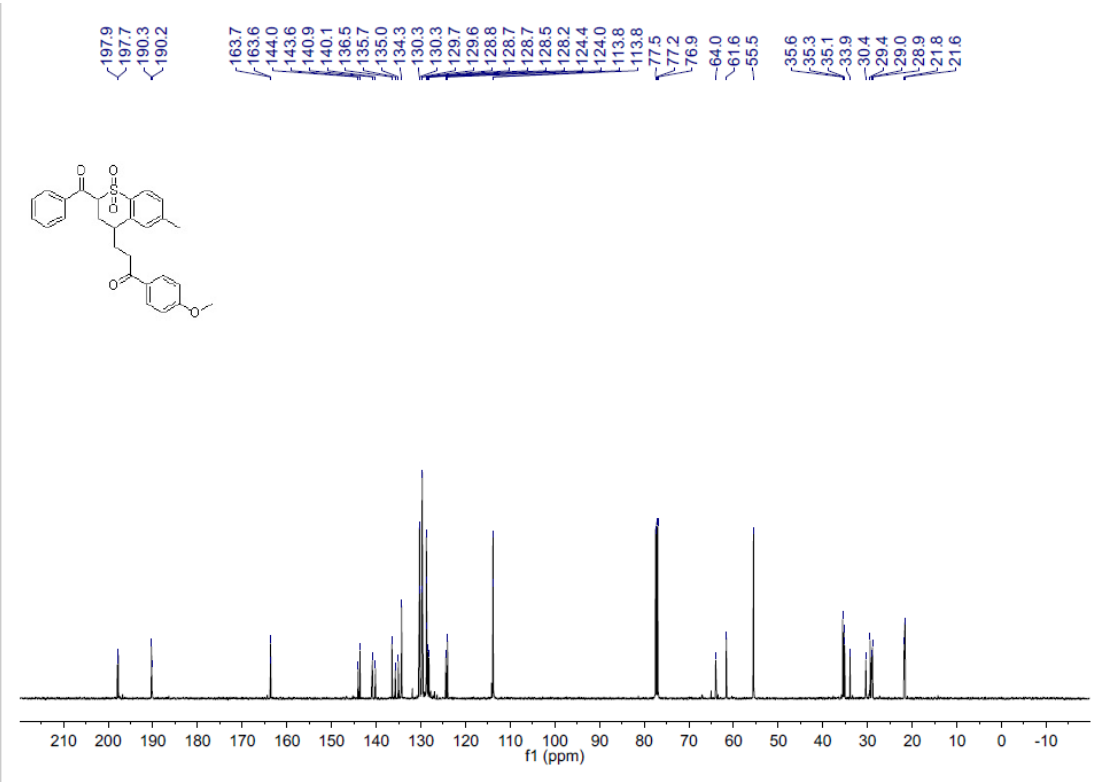


$^{1}$H NMR (500 MHz, $CDCl_3$) spectrum of compound **5ah**

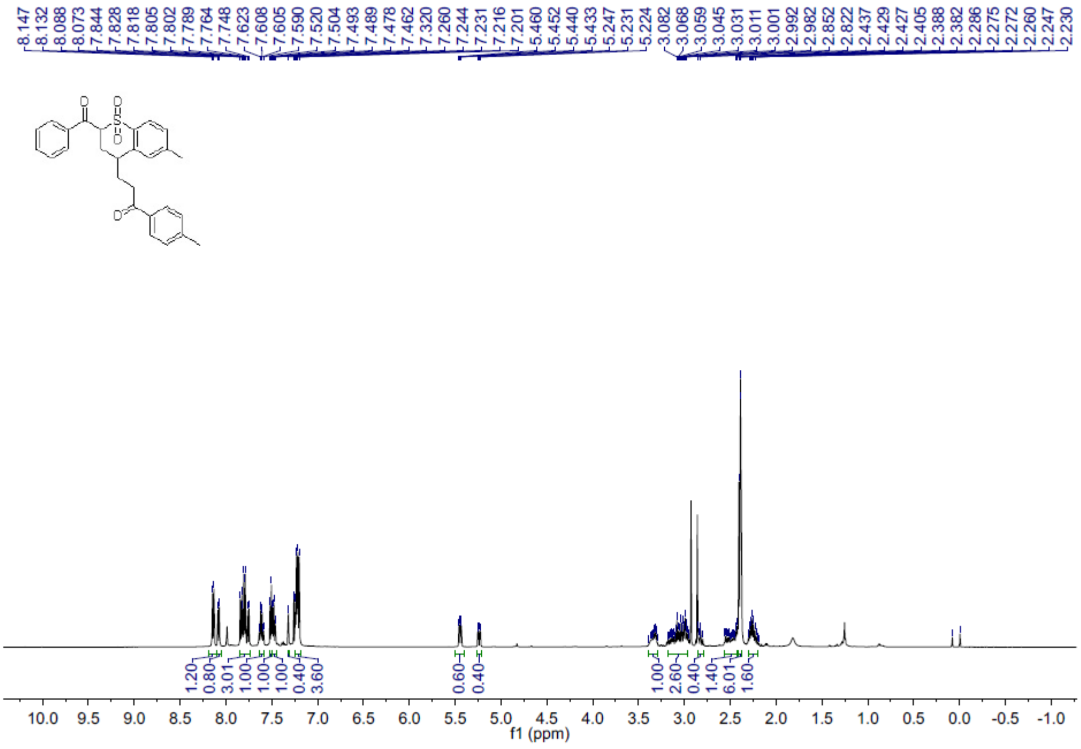

$^{13}C$ {$^{1}H$} NMR (126 MHz, $CDCl_3$) spectrum of compound **5ah**

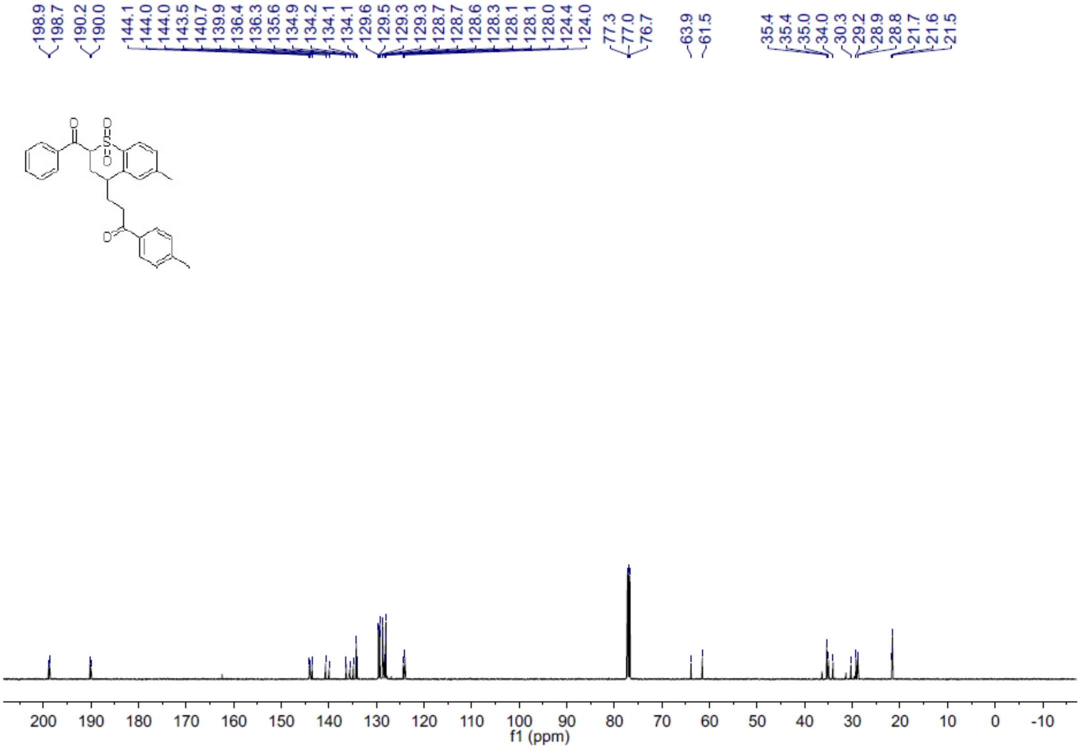


$^{1}H$ NMR (500 MHz, $CDCl_3$) spectrum of compound **5ai**

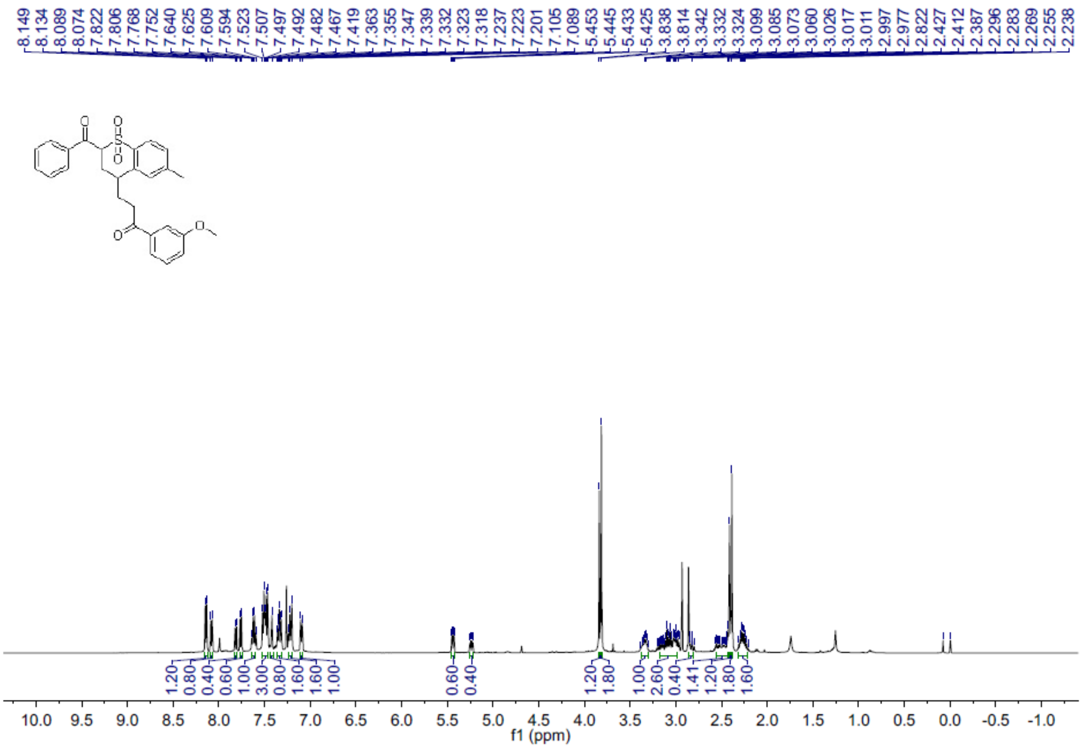

$^{13}C$ {$^{1}H$} NMR (126 MHz, $CDCl_3$) spectrum of compound **5ai**

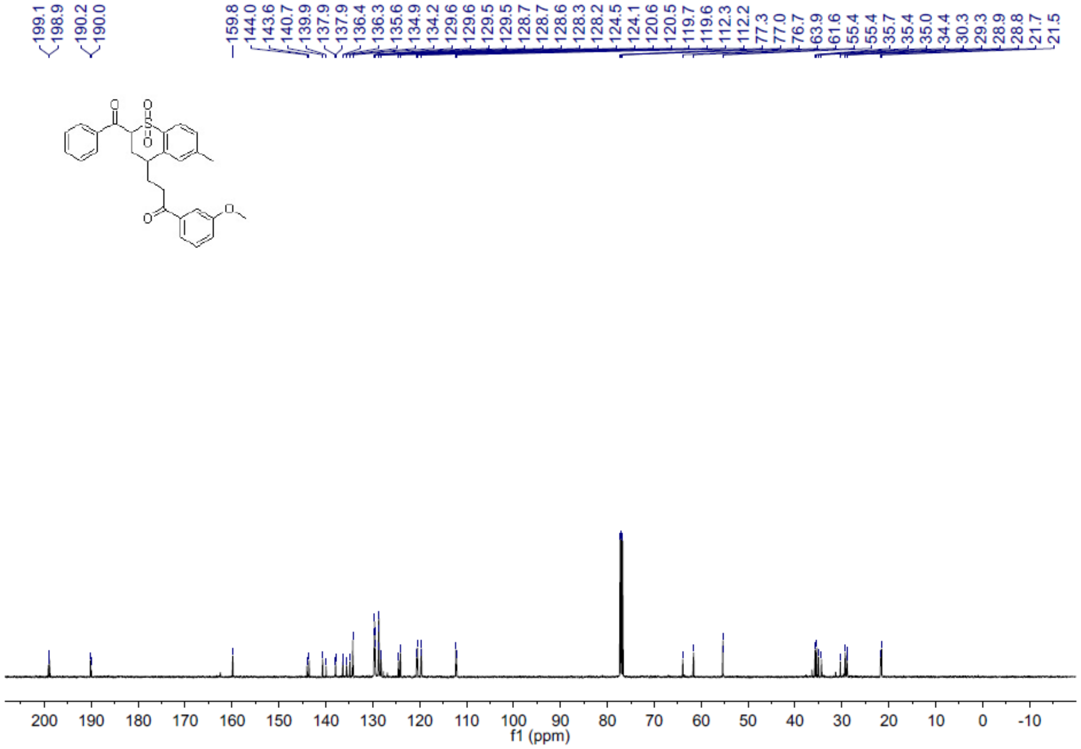


$^{1}H$ NMR (500 MHz, $CDCl_3$) spectrum of compound **5aj**

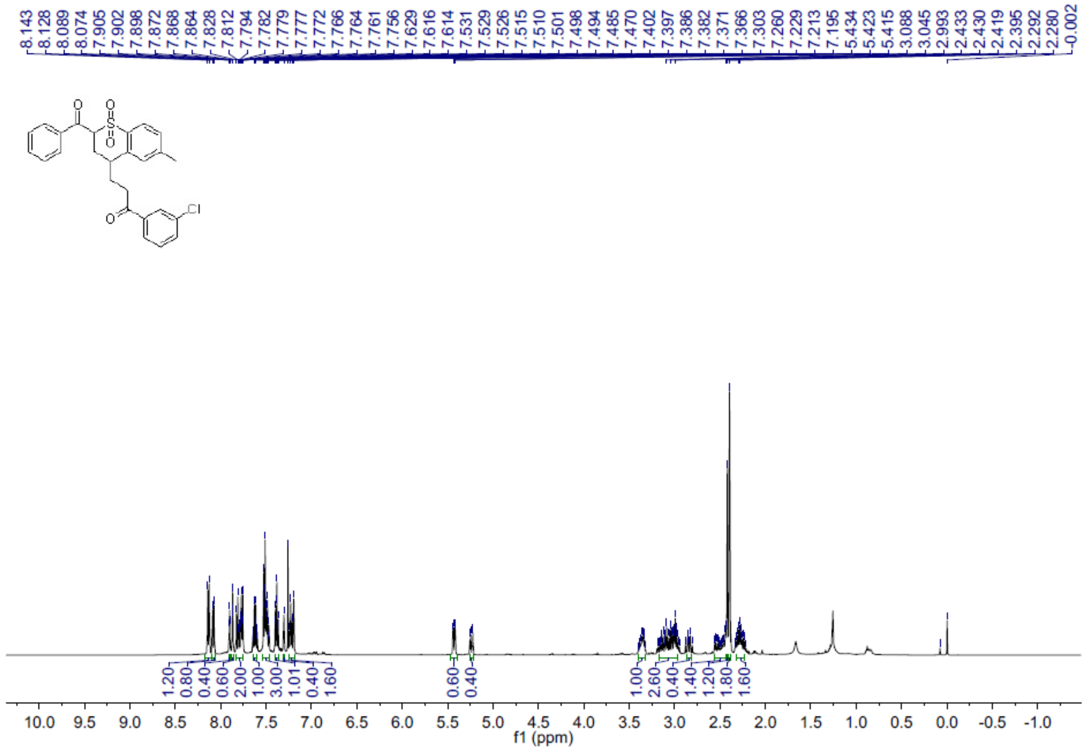

$^{13}C$ {$^{1}H$} NMR (126 MHz, $CDCl_3$) spectrum of compound **5aj**

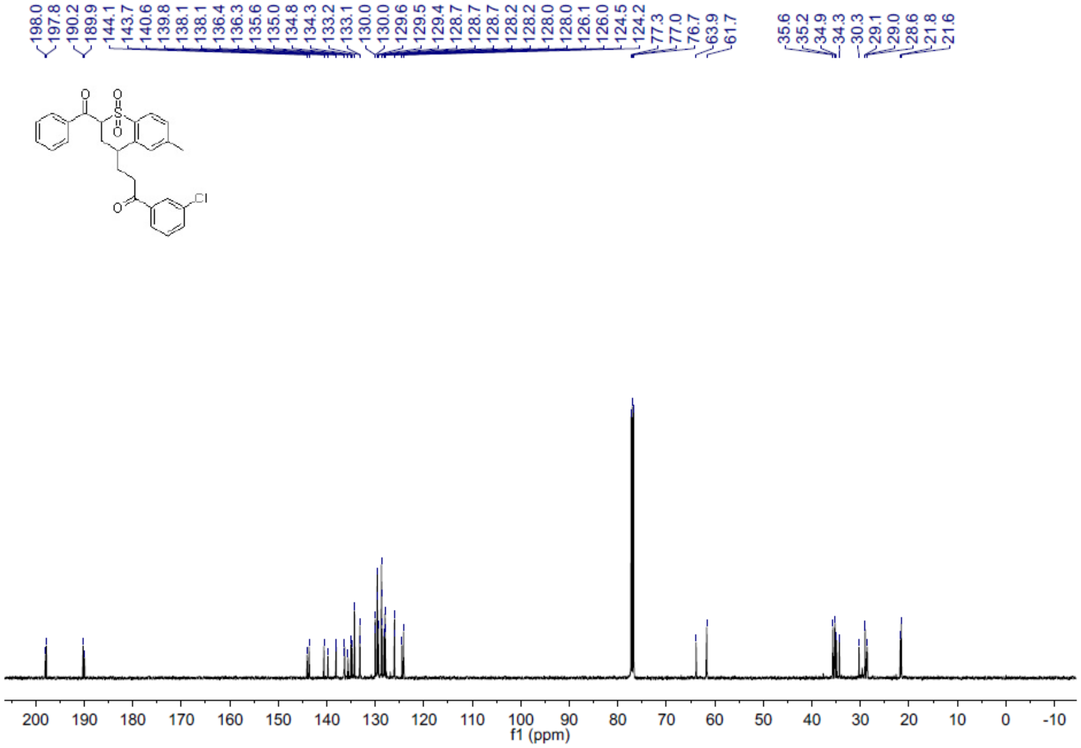


$^{1}H$ NMR (500 MHz, $CDCl_3$) spectrum of compound **5ak**

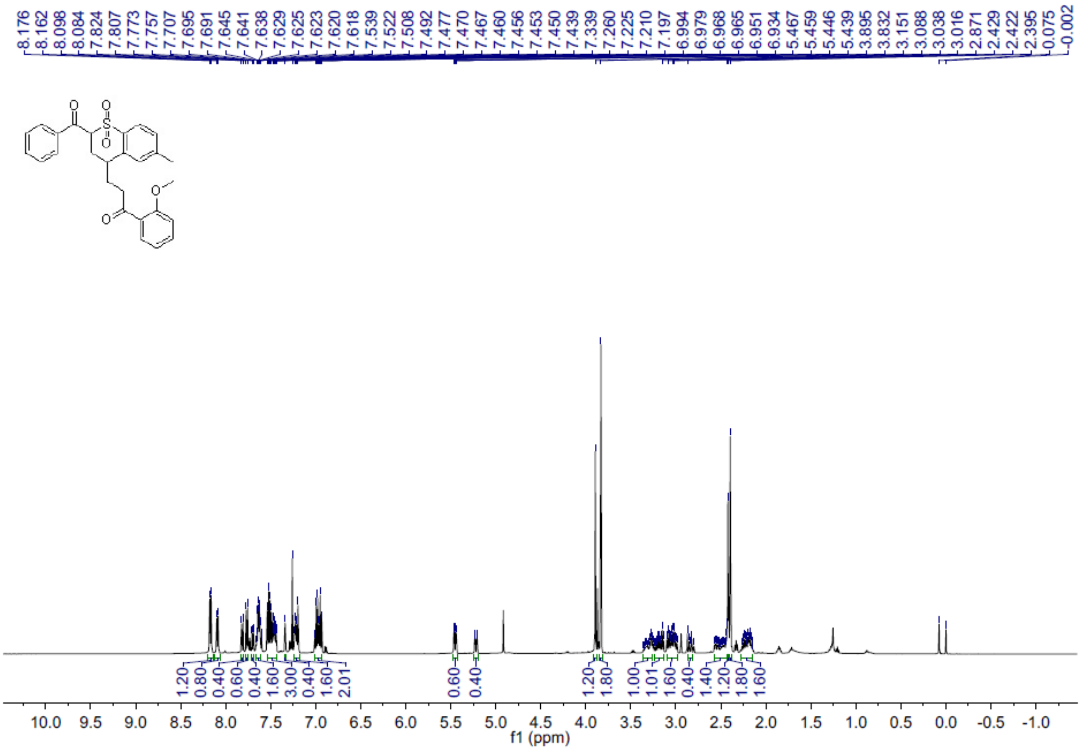

$^{13}C$ {$^{1}H$} NMR (126 MHz, $CDCl_3$) spectrum of compound **5ak**

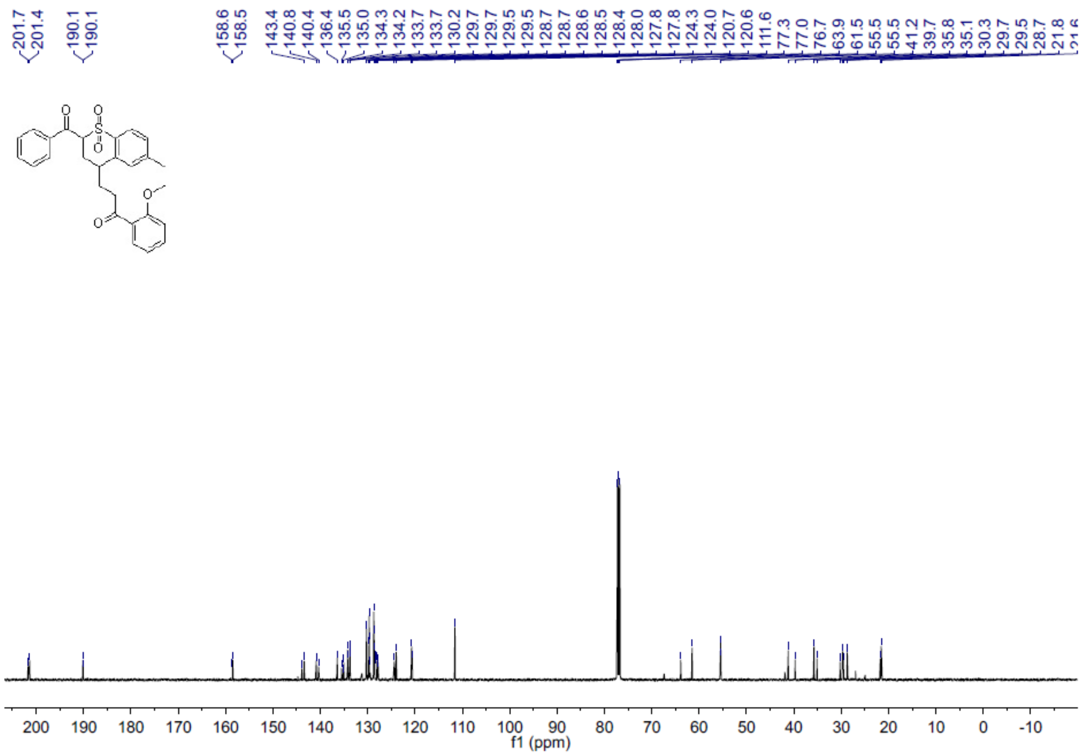


$^{1}H$ NMR (500 MHz, $CDCl_3$) spectrum of compound **5al**

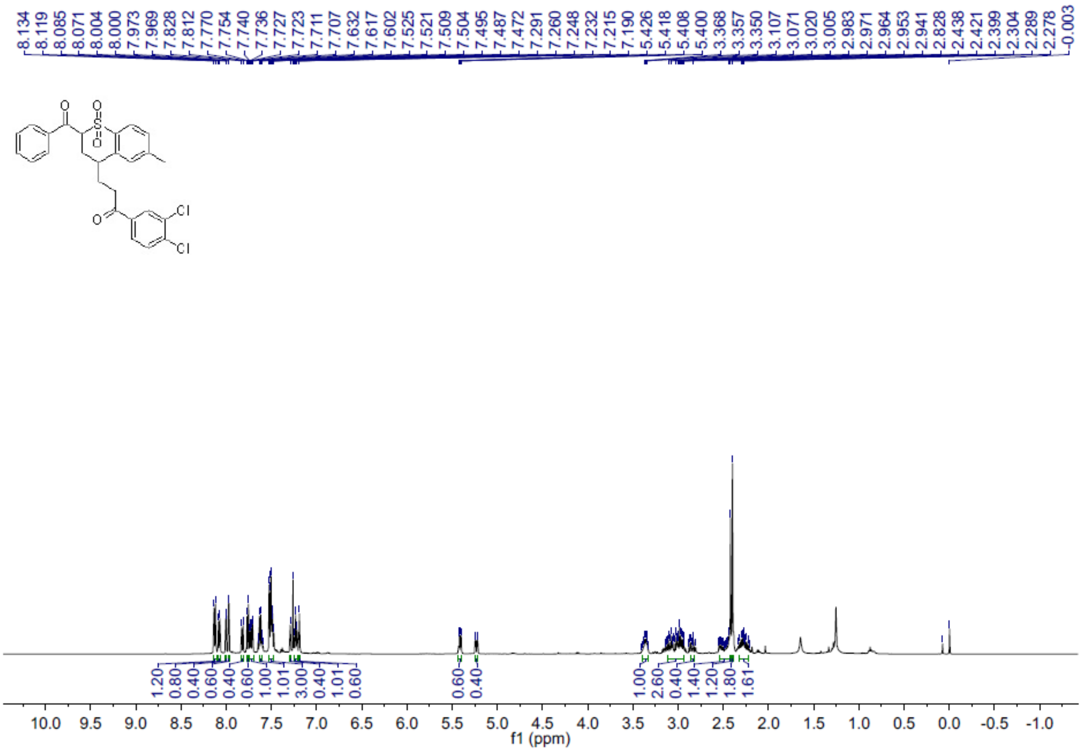

$^{13}$C {$^{1}$H} NMR (126 MHz, $CDCl_3$) spectrum of compound **5al**

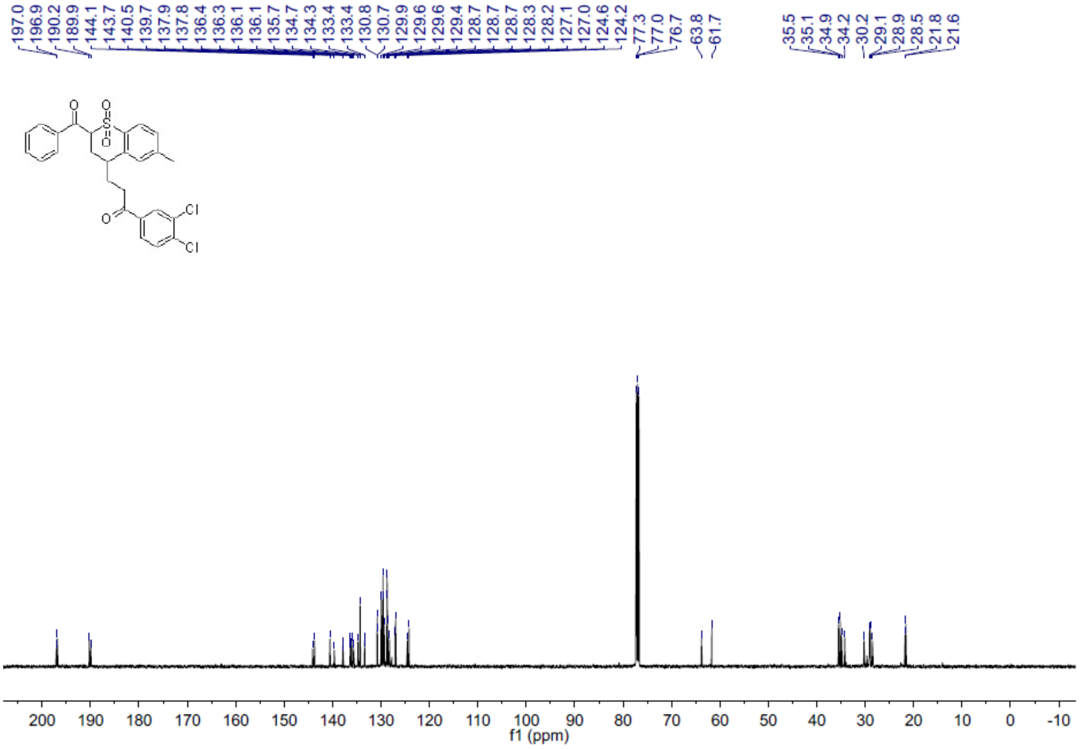


$^{1}$H NMR (500 MHz, $CDCl_3$) spectrum of compound **5am**

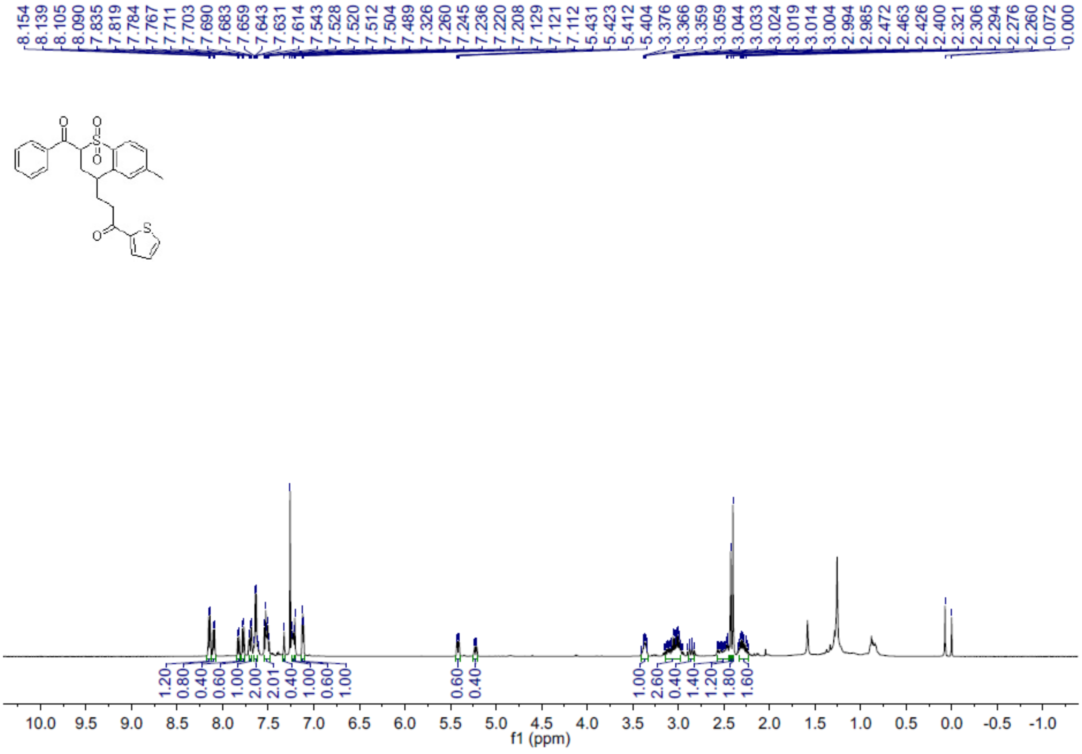

$^{13}C$ {$^{1}H$} NMR (126 MHz, $CDCl_3$) spectrum of compound **5am**

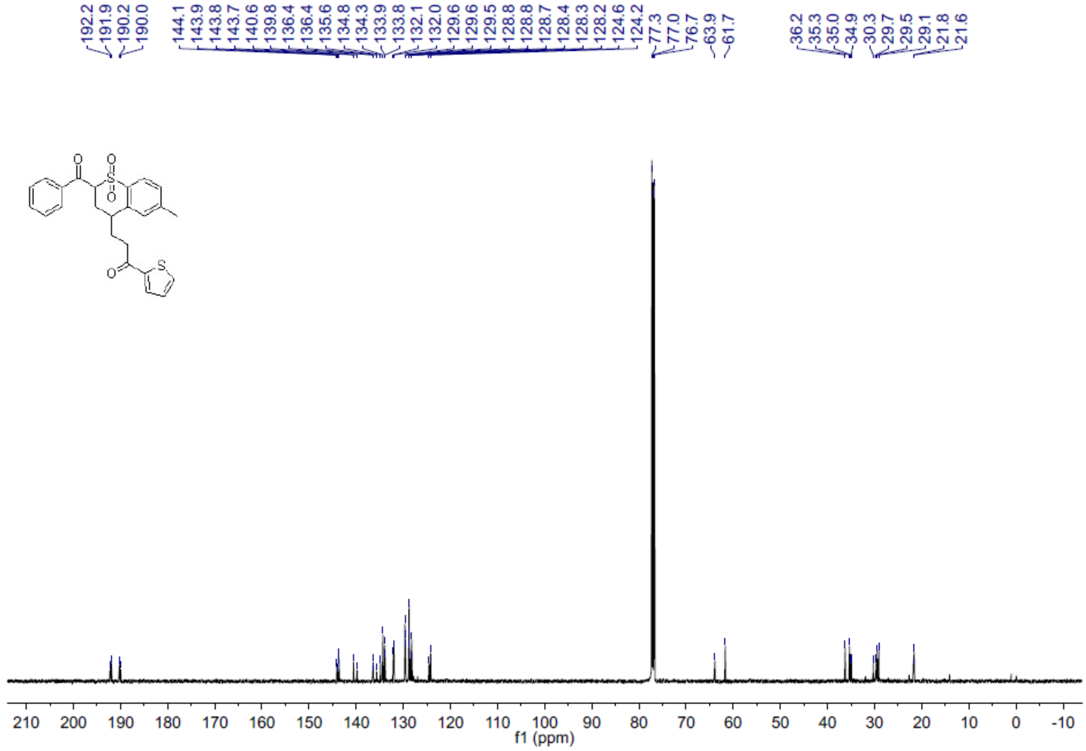


$^{1}H$ NMR (500 MHz, $CDCl_3$) spectrum of compound **5an**

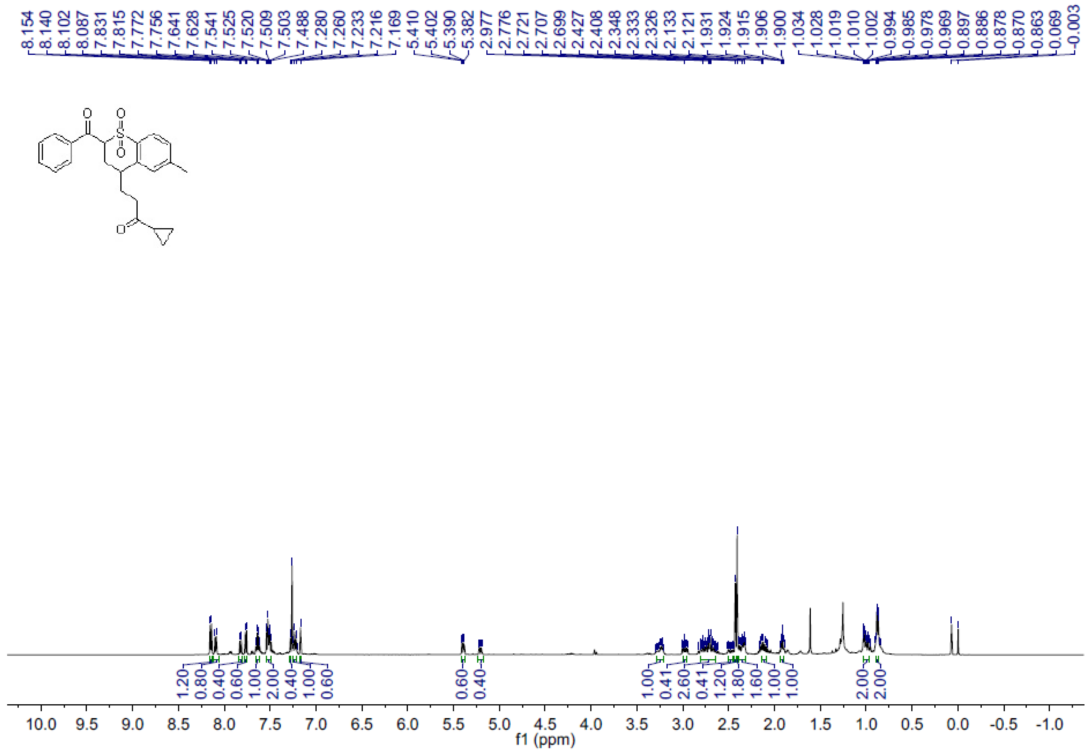

$^{13}C$ {$^{1}H$} NMR (126 MHz, $CDCl_3$) spectrum of compound **5an**

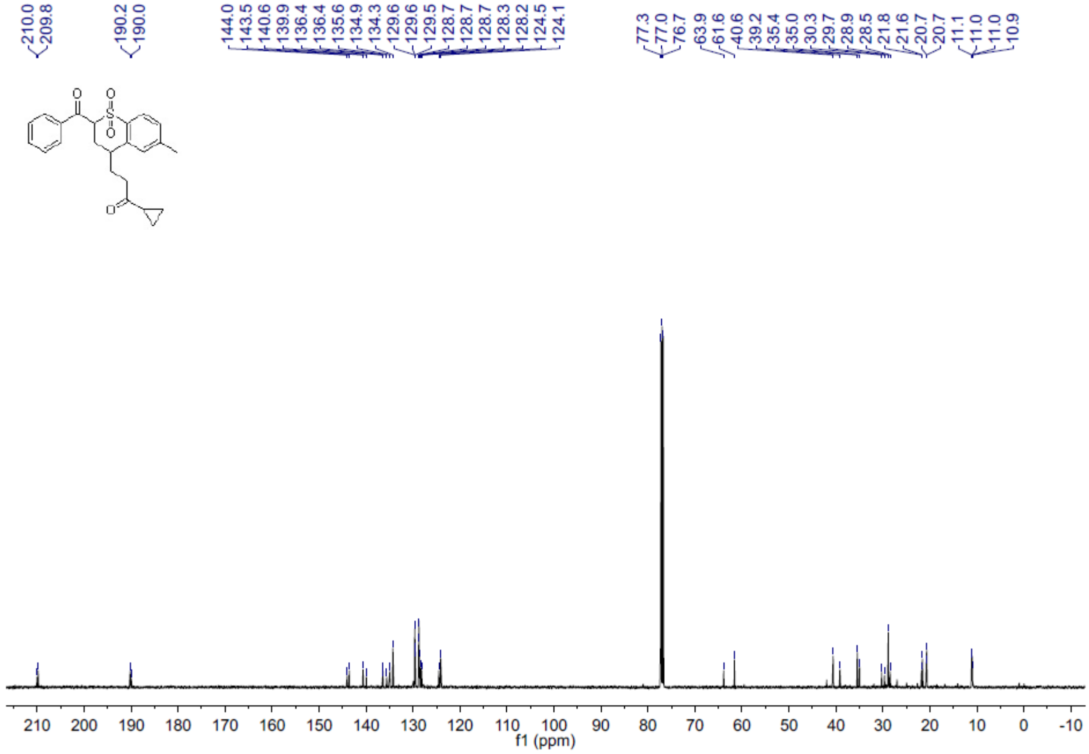

Supplement: Supplementary file 1 [file molecules-29-01971-s001.zip › molecules-2881106-supplementary.pdf]
